# Analysis of the early response to spinal cord injury identified a key role for mTORC1 signaling in the activation of neural stem progenitor cells

**DOI:** 10.1038/s41536-021-00179-3

**Published:** 2021-10-22

**Authors:** Johany Peñailillo, Miriam Palacios, Constanza Mounieres, Rosana Muñoz, Paula G. Slater, Elena De Domenico, Ilya Patrushev, Mike Gilchrist, Juan Larraín

**Affiliations:** 1 Center for Aging and Regeneration, Departamento de Biología Celular y Molecular, Facultad de Ciencias Biológicas, Pontificia Universidad Católica de Chile, Alameda 340, Santiago, Chile; 2 Departamento de Tecnología Médica, Facultad de Medicina, Universidad de Chile, Santiago, Chile; 3 The Francis Crick Institute, 1 Midland Road, London, UK; 4 German Center for Neurodegenerative Diseases (DZNE), PRECISE Platform for Genomics and Epigenomics at DZNE, University of Bonn, Bonn, Germany

**Keywords:** Transcriptomics, Regeneration

## Abstract

*Xenopus laevis* are able to regenerate the spinal cord during larvae stages through the activation of neural stem progenitor cells (NSPCs). Here we use high-resolution expression profiling to characterize the early transcriptome changes induced after spinal cord injury, aiming to identify the signals that trigger NSPC proliferation. The analysis delineates a pathway that starts with a rapid and transitory activation of immediate early genes, followed by migration processes and immune response genes, the pervasive increase of NSPC-specific ribosome biogenesis factors, and genes involved in stem cell proliferation. Western blot and immunofluorescence analysis showed that mTORC1 is rapidly and transiently activated after SCI, and its pharmacological inhibition impairs spinal cord regeneration and proliferation of NSPC through the downregulation of genes involved in the G1/S transition of cell cycle, with a strong effect on PCNA. We propose that the mTOR signaling pathway is a key player in the activation of NPSCs during the early steps of spinal cord regeneration.

## Introduction

Although mammals can regenerate some tissues like the skin, muscle, bones, and liver, they are unable to efficiently regenerate the central nervous system (CNS). Particularly, regeneration of the spinal cord is very deficient, and spinal cord injury (SCI) results in loss of sensory, motor, and autonomic functions altering the autonomy and the life quality of human beings. SCI in mammals not only produces an immediate damage of neurons, axonal circuits, and the tissue in the injured area, which is followed by a second phase that increases the damage and forms a glial scar that limits the tissue loss, but also acts as a barrier for axonal growth^1–5^. In addition, axon regeneration is absent in mammals^6^, and although neural stem progenitor cells (NSPCs) are activated, they are mainly fated to astrocytes, and the formation of new neurons is not observed in vivo^7–9^.

On the contrary, other species, including planarias, fish, amphibians and lampreys, have regenerative mechanisms that allow the anatomical and functional recovery of complex structures, such as organs, eyes, neural tissue, and appendages^10–13^. Amphibians and teleost fish are model organisms widely used to understand the molecular and cellular mechanisms involved in spinal cord regeneration^14,15^. Among these, *Xenopus laevis* can be highlighted because it has great regenerative capabilities during larvae stages (regenerative stages, R-stages), being able to repair severe lesions of the CNS, including the spinal cord. However, this ability is lost during metamorphosis, and thus, froglets and adult frogs (non-regenerative stages, NR-stages) are not able to recover from SCI anymore^16,17^. Previous studies demonstrated that NSPC expressing Sox2 start to proliferate after SCI and are required for spinal cord regeneration in R-stage animals, whereas this is not observed in NR-stage animals, despite having Sox2^+^ cells in their spinal cord^18,19^.

Global studies comparing the transcriptomes deployed after SCI between R- and NR-stages at 1, 2, and 6 days post-transection (dpT) showed a fast and massive transcriptome change at 1 dpT in R-stage^20^. In these animals, enrichment in genes associated with the cell cycle was detected at 1 and 2 dpT, suggesting that many changes occur within the first 24 h after injury, including the activation of a signal to trigger NSPC proliferation. Based on this, we envision that a deep study of the transcriptome changes during the first day after injury should allow the identification of the signaling pathways and mechanism involved in NSPC activation.

Here we use high-resolution expression profiling and Gaussian Process approach^21,22^ to identify the transcriptome changes during the first 21 h after SCI. The gene expression profiles were classified into early, intermediate, and late, according to their kinetics. Early modules show a rapid and transitory activation characteristic of a primary response, with a large component of immediate early genes (IEGs), meanwhile intermediate and late modules have a sustained activation representative of a secondary response. Among the main biological processes present in these phases, we found a large component of genes associated with migration and immune response, ribosome biogenesis, and cell cycle, as well as a downregulation of negative regulators of mechanistic Target of Rapamycin (mTOR) pathway. Western blot and immunofluorescence analysis confirmed that mTORC1 is rapidly and transiently activated after SCI, and its pharmacological inhibition impairs spinal cord regeneration and proliferation of NSPC. This was confirmed through the evaluation of global transcriptional changes after inhibition of mTOR pathways, showing that mTOR targets the expression of genes involved in transition G1/S of the cell cycle, with a strong effect over genes such as proliferating cell nuclear antigen (PCNA). We propose that the mTOR signaling pathway is a key player in the activation of NPSCs during the early steps of spinal cord regeneration in *X. laevis*.

## Results

### mRNA sequencing (mRNA-seq) and data consistency

**Fig. 1:**
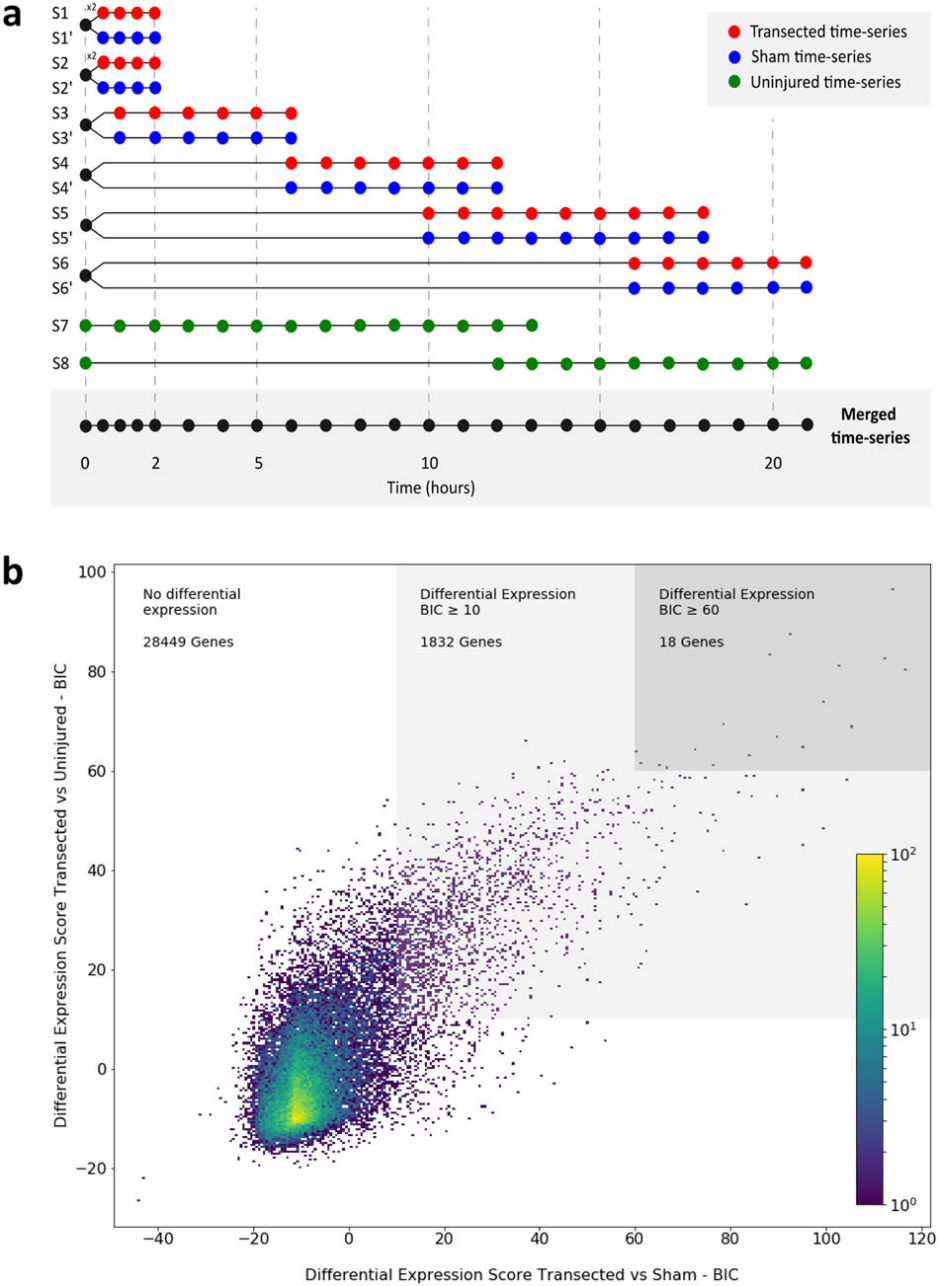
Early transcriptome changes in response to SCI. **a** Time-series samples obtained for mRNA-seq. Spinal cords were isolated in six time series from transected and sham conditions. Between 0 and 2 h, samples were obtained every 30 min and at 1 h intervals between 2 and 21 h. Samples for the uninjured condition were isolated in two time series at 1 h intervals between 0 and 21 h. **b** 2D histogram for differentially expressed genes. The graph depicts differential gene expression comparing transected and uninjured conditions (*y* axis) with transected and sham conditions (*x* axis). The light gray square highlights genes with a BIC ≥ 10 in both comparisons, and those on the dark gray square correspond to genes with a BIC ≥ 60.

To characterize the genes deployed in the spinal cord of *X. laevis* immediately after SCI, high-resolution expression profiling^21^ was used to continually evaluate the global transcriptome changes from 0 to 21 h post-transection (hpT) and were compared to sham and uninjured animals (Fig. 1a). For transected animals, samples were isolated synchronously every 30-min between 0 and 2 hpT and every hour between 2 and 21 hpT. Samples were collected in six series (Fig. 1a): (i) series 1 and 2 (S1 and S2), correspond to animals from the same fertilization used for time points between 0 to 2 hpT, including a biological replicate for 0 hpT, (ii) series 3, 4, 5, and 6 (S3–S6) were obtained from different batches of animals to cover the periods between 0 and 6 hpT, between 6 and 12 hpT; between 10 and 18 hpT, and between 16 and 21 hpT, respectively. These last four series also contained samples at 0 hpT and 1–3 time points overlapping with the subsequent series to facilitate posterior merge and to evaluate continuity between the different time series.

Two sets of control samples were also prepared. One of the controls (“Sham" control, blue in Fig. 1a) corresponded to samples from sham-operated animals, in which only the dorsal skin and muscle were injured at the same level of the rostro-caudal axes as the transected animals, but the spinal cord was left uninjured^17^. This control allowed the exclusion of transcriptional changes in the spinal cord produced as a consequence of damage to tissues surrounding the spinal cord. Sham control samples were obtained in six time series (S1’–S6’), in parallel and from the same batches of animals used to obtain the samples from transected animals, at equivalent time points, and sharing the 0 h time point (Fig. 1a). For a second control (“Uninjured”, green in Fig. 1a), spinal cord tissue was isolated at the same level of the rostro-caudal axes from sham and transected animals. This control samples permitted the elimination of transcriptional changes associated with the normal progress of metamorphosis during the time lapse analyzed. Uninjured time series were collected from the same animal batch in two series at 1-h intervals: one from 0 to 13 h (S7) and the other one from 12 to 21 h (S8), including 0 h, and 12 and 13 h used as overlapping points (Fig. 1a).

Illumina RNA-seq polyA+ libraries were constructed for the 105 samples collected in the three conditions indicated above. An average of 28 million paired-end reads per sample were sequenced with an average of 69% of reads mapped per library (Table S1) and 43,193 genes detected (95.8% of the transcriptome) with a mean of 443 gene counts per library. To avoid noise, only genes having five or more gene counts detected in at least four temporal points in some of the three conditions were considered, resulting in 30,299 genes, which were contemplated for the following analyses. Using Spearman correlation coefficients to check the consistency of the data, we found a high degree of internal consistency for neighboring points within each time series, and although slightly lower the consistency between series was still strong. Because of the variation, the batch effect was corrected, and a better fit was observed between the time series (for more details about data consistency, see the “Methods” section).

In summary, high-quality sets of RNA libraries, at contiguous time points during the first 21 hpT, were generated. The data obtained was very reliable based on the quality of the sequencing, the mapping and gene detection rates, and more importantly the high degree of internal consistency within and among each time series supports that the sampling is representative of the undergoing biological processes.

### Gene co-expression networks present in the early response to SCI

For identification of changes in the transcriptome, the temporal gene expression profiles between transected and uninjured and between transected and sham conditions were compared using Gaussian Processes. To do this, two hypotheses were tested for each comparison, one in which the data of both conditions are fitted to the same temporal profile of gene expression through the time points measured, and a second hypothesis, in which both data sets are fitted to two different expression profiles throughout the time points measured. To choose between both, the logarithm of the marginal likelihoods of the two hypotheses were compared using Bayesian Information Criterion (BIC). A temporal differential gene expression is assigned when a BIC > 0 is obtained. For a better visualization of the changes in the transcriptome after SCI, a two-dimensional histogram was constructed, where the BIC values of the comparison between transected and uninjured conditions are shown on the *y*-axis, and the BIC values of the comparison between transected and sham on the *x*-axis (Fig. 1b).

To select those genes with higher changes in the levels of mRNA between the compared conditions, a BIC ≥ 10 was established as a cut-off, which is considered as a very strong evidence for alternative hypothesis in model selection^23^. The number of genes with a BIC ≥ 10 in the transected against uninjured comparison (4917 genes) was higher than those observed in the transected against sham comparison (2269 genes), suggesting that injury of tissues close to the spinal cord could also elicit a response on the spinal cord, reinforcing the importance of the sham control. Finally, an intersection of 1850 genes, with BIC ≥ 10 in both comparisons, was defined as those genes that change their expression after a direct damage to spinal cord and were used for further analysis.

**Fig. 2:**
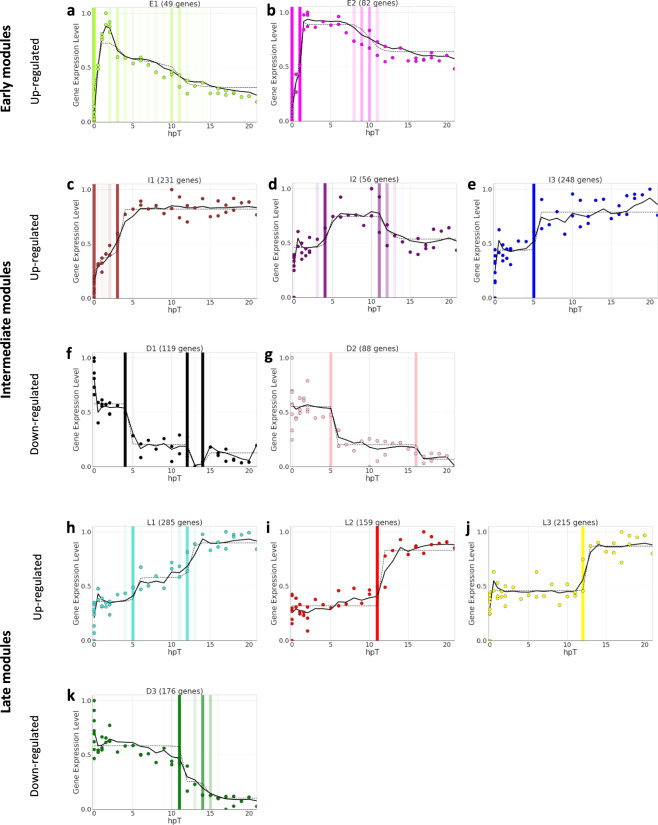
Co-expression modules deployed in response to spinal cord injury. WGCNA analysis clustered genes in 11 co-expression modules. The graphs show the adjusted kinetic profile for each module. Colored dots represent the eigengene modules, continuous lines indicate a regime-switching model used to explore possible change points, discontinuous lines show a change-point model for each module, and vertical color bars highlight temporal points at which we observe a change in the direction of gene expression, with more intense bars indicating a higher probability of change. **a**, **b** Early upregulated modules E1 and E2; **c**–**e** intermediate upregulated modules I1, I2, and I3; **f**, **g** intermediate downregulated modules D1 and D2; and (**h**–**k**) late upregulated L1, L2, and L3 and downregulated module D3.

To identify groups of genes that have similar expression profiles in response to SCI, a weighted gene co-expression network analysis (WGCNA) for the 1850 genes was performed. Using this approach, 92.3% of the 1850 genes were clustered in 11 co-expression modules (Table S2). The differences between co-expression modules are shown in a hierarchical clustering based on the Euclidean distance calculated from the module eigengene of each one (Supplementary Fig. S1). Based on module eigengene and its correlation with the time after lesion, three modules presented downregulated mRNA levels, and the other eight modules showed upregulated levels (Fig. 2). According to their kinetic profiles, the eleven modules were organized in: (i) early upregulated modules (E1, E2), (ii) intermediate modules that were either upregulated (I1, I2, and I3) or downregulated (D1, D2), and (iii) late modules that were also upregulated (L1, L2, and L3) or downregulated (D3).

Genes in modules E1 and E2 showed an upregulation even before 1 hpT and reached their maximum expression levels at around 2 hpT (Fig. 2a, b). In both cases, upregulation was transient, and a decline started around 2 and 8 hpT, but without returning to basal levels. mRNA levels in intermediate modules changed (increase or decrease) between 2 and 5 hpT, reaching stable levels around 4–6 hpT, except for I2 that shows a transitory kinetic falling-down around 11–12 hpT concomitant with the activation of late modules (Fig. 2c–g). Finally, RNA levels in late modules only changed at 11–12 hpT, and the achieved levels were maintained (Fig. 2h–k). Of note, for the L1 module two phases of upregulation were appreciated, one at 4–6 hpT that was coincident with the intermediate modules, followed by a second phase that was more similar to the late modules (Fig. 2h).

Interestingly, later time modules contained a larger number of mRNAs. E1 and E2 modules have an average of 65.5 genes; D1, D2, I1, I2, and I3 include an average of 147.6 genes in each module; and the number of genes on the late modules rises to an average of 206.3 genes. This suggests that changes in mRNA levels were deployed in a sequential manner, adjusting to a cascade effect, which would produce a progressive increase in transcriptome changes during the first 21 h of the regenerative response.

### Gene ontology (GO) enrichment analysis

**Fig. 3:**
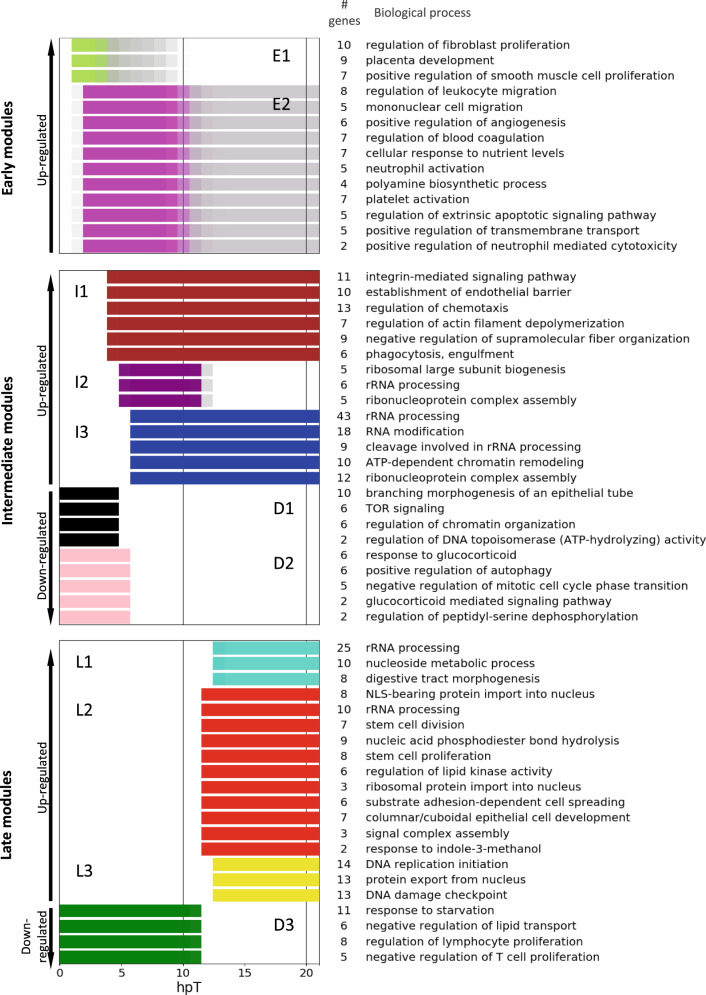
Biological process regulated during the early response to spinal cord injury. Gene ontology enrichment analysis was performed for each co-expression module. The most representative biological processes are described. Color bars associated with each GO term represent the time period when they showed maximum expression. For downregulated modules, the color bars disappear when the expression levels of those genes start to decrease. The number of genes in each GO group is also indicated.

To identify the main biological processes, molecular functions, cellular components, and signaling pathways deployed during the first 21 h after SCI, a GO and Kyoto Encyclopedia of Genes and Genomes (KEGG) pathway enrichment analysis was performed to the 11 co-expression modules. Because several and redundant GO terms were enriched within some modules, an *in-house* clustering method based on semantic similarities between GO terms and genes associated with them was implemented. From this analysis, a representative GO term was chosen for each cluster considering its significance and the differential expression level of the associated genes (Fig. 3 and Supplementary Tables S3–S5). Here we describe the main biological concepts that emerged from an integrated examination of the GO terms and KEGG pathway enrichment analyses.

A detailed examination of the molecular function category from the GO enrichment analysis showed that all groups enriched in module E1 are related to transcriptional components (Supplementary Table S3). In addition, 36.7% of the genes in E1 correspond to transcription factors, a large proportion compared to the other modules in which only an average of 6.5% was detected. The kinetic profile observed for E1 genes is very similar to the behavior described for IEGs, which act as first responders inducing a transcriptome remodeling in response to a stimulus, presenting maximal activation levels around 1 h after activation, and falling very rapidly^24^. Moreover, 48% of the genes in E1 have already been reported as IEGs, among them some widely studied as fos, jun, and myc^25^.

Some of the biological processes enriched in modules E1 and E2 are related to the inflammatory response, including the activation and migration of leukocytes and neutrophils (Fig. 3 and Supplementary Table S4). Furthermore, in the KEGG analysis many of the pathways identified are also related to these processes (Supplementary Table S6). This includes key signaling pathways such as tumor necrosis factor and interleukin (IL)-17, which are known for their pervasive role in triggering an inflammatory response^26,27^, and Toll-like receptor signaling that could activate an innate immune response^28^. The early upregulation reported for these genes, followed by a fast reduction on expression levels, suggest that this immune response is rapid, transient, and tightly controlled.

An enrichment of genes associated with mTOR signaling both in the biological process and KEGG pathway analyses was found in module D1 (Fig. 3 and Supplementary Tables S4 and S6). The mRNA levels for *deptor* (DEP Domain Containing mTOR Interacting Protein), and *tsc2* (TSC Complex Subunit 2), two negative regulators of mTOR, were rapidly reduced after injury, suggesting a possible fast activation of mTOR signaling. Moreover, enrichment of biological processes directly regulated by mTOR pathway such as autophagy, ribosome biogenesis, and starvation response^29^ were found in other intermediate (D2, I2, and I3) and late (D3) modules as well, suggesting that mTOR could play a key role during early response after injury. Related to this, a decrease on mRNA levels of genes involved in positive regulation of autophagy were also enriched on module D2 (Fig. 3 and Supplementary Tables S4).

Ribosome biogenesis represents a very widespread biological process throughout the first 21 h of the response to SCI. In particular, the components needed for ribosome biosynthesis represent almost all GO terms and KEGG pathways enriched in modules I2 and I3 (Fig. 3 and Supplementary Tables S3–S6). This GO include genes involved in: ribosome large subunit biogenesis, rRNA processing, ribonucleoprotein complex assembly, RNA modification and methyltransferase activity, mRNA, transfer RNA and small nucleolar RNA binding, and preribosome. Notably, among these genes, there are several ribosome biogenesis factors (RBFs) that are important and specific for stem cell homeostasis and self-renewal^30^.

Intermediate modules contain GO terms related to chromatin remodeling. Some are upregulated, like ATP-dependent chromatin remodeling, nuclear matrix, methyltransferase complex, SWI/SNF complex, and catalytic activity acting on DNA, that are enriched in module I3 (Fig. 3 and Supplementary Tables S4 and S5). And others enriched in D1 are downregulated, such as regulation of chromatin organization and regulation of DNA topoisomerase activity (Fig. 3 and Supplementary Table S4). From this, it is inferred that an important chromatin remodeling starts at around 6 hpT and could be a key process in the response to SCI^31^.

In agreement with the finding about ribosome biosynthesis, an enrichment of GO terms related to protein synthesis was also observed in modules L1, L2, and L3 (Supplementary Tables S3–S6 and Fig. 3). These groups included GO terms and signaling pathways, such as transferring of glycosyl groups, misfolded protein binding, peptide transporter activity, cytoplasmic ribonucleoprotein granule, endoplasmic reticulum chaperone complex, protein processing in endoplasmic reticulum, N-glycan biosynthesis, and protein export from nucleus.

The GO terms and KEGG pathways DNA replication initiation, DNA replication origin binding, MCM complex, cell cycle, stem cell division, and proliferation and signaling pathways regulating pluripotency of stem cells were enriched in modules L2 and L3 (Fig. 3 and Supplementary Tables S3–S6). Of note, in module L2, a concomitant enrichment in genes linked to columnar/cuboidal epithelial cell development was found, which correspond to the morphological organization of neural progenitor cells in the central canal of spinal cord (Fig. 3). Furthermore, genes linked to neurogenesis, such as nestin and components of the WNT pathway (wnt7b, fzd2, fzd3, and ctnnb1), were found as well. These results, together with the activation of important RBFs involved in stem cell homeostasis and self-renewal, suggest that activation of NSPC is a key process in spinal cord regeneration.

An enrichment of the biological process related to lymphocyte and T cell proliferation is observed in module D3, with a significant reduction of RNA levels starting at 11 hpT (Fig. 3 and Supplementary Table S4). Among the genes included in these GO terms, we found irs4, which inhibits JAK/Stat signaling pathway, modulating the immune response^32^; PDE5A (phosphodiesterase 5A), whose pharmacological inhibition have been associated with anti-inflammatory and neuroprotective effects^33^; and shh (sonic hedgehog), which has been implicated in macrophage polarization after SCI, regulating pro- and anti-inflammatory response^34^. This analysis suggests that there is an active mechanism to control and limit the immune response.

We observe an enrichment in genes associated with “regulation of extrinsic apoptotic signaling pathway” in module E2; however, these genes showed an early and transitory activation (Fig. 3). Moreover, we observe a downregulation of casp3 mRNA and upregulation of bcl2l12 mRNA in late modules D3 and L1, respectively. Casp3 is associated with apoptosis, meanwhile bcl2l12 has an anti-apoptotic role (see Supplementary Information, Table S2). These transcriptional changes suggest a downregulation of apoptotic process during the first stages of spinal cord regeneration.

### Early activation of the mTORC1 signaling pathway in response to SCI

mTOR is a signaling pathway that coordinates cell growth and metabolism and is associated with cancer, regeneration, aging, and other processes^29^. The bioinformatics analysis described above suggests that mTOR is activated because a downregulation of negative regulators, such as *tsc2* and *deptor*, was observed (Fig. S2). In addition, we detected the upregulation of *rps6kb1* and *eif4e*, two key components of the ribosome biogenesis and protein synthesis branches, respectively (Fig. S2).

**Fig. 4:**
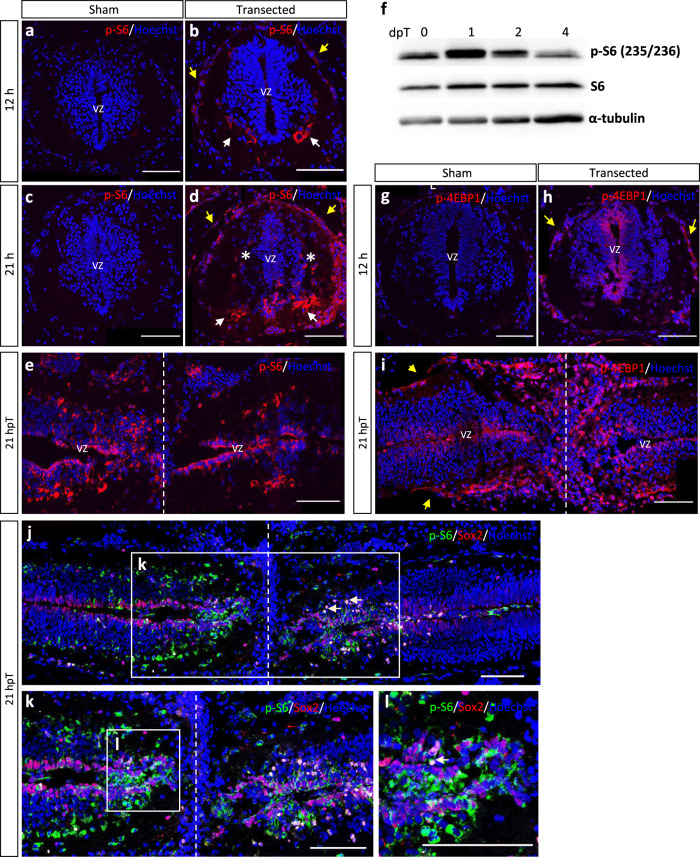
Early activation of mTOR signaling in response to SCI. Activation of mTORC1 was analyzed by immunostaining (**a**–**e**) and immunoblotting (**f**) for p-S6, by immunostaining (**g**–**i**) against p-4EBP1, and (**j**–**l**) double immunostaining for Sox2 and pS6. p-S6 levels were analyzed in transverse cryosections at (**a**, **b**) 12 and (**c**, **d**) 21 h after transection or sham operation and also in longitudinal cryosections at (**e**) 21 hpT. **f** Western blot for S6 and p-S6 in total protein extracts obtained from spinal cords at 0, 1, 2, and 4 dpT for evaluation of mTOR signaling pathway activation after injury. See quantification of five similar blots in Supplementary Fig. S3a. p-4EBP1 levels were analyzed in transversal cryosections at (**g**, **h**) 21 h after transection or sham operation and also in longitudinal cryosections at **i** 21 hpT. Yellow arrow: meningeal cells, white arrow: motoneurons, *: lateral neurons, vz: ventricular zone, dotted line: injury site or ablation gap. Sox2 and p-S6 were analyzed in longitudinal cryosections (**j**), and (**k**, **l**) correspond to magnifications of (**j**, **k**), respectively. White arrows show Sox2^+^ cells with higher levels of p-S6 activation. Immunofluorescences were performed in two or three biological replicates. Scale bar = 100 μm. All the blots included in this figure were derived from the same experiment and were processed in parallel.

To evaluate the activation of ribosome biogenesis and cap-dependent translation, we measured the phosphorylation levels of S6 (p-S6) and 4EBP1 (p-4EBP1), respectively^29^. p-S6 signal was not detected in sham operated, nor in uninjured animals, by immunofluorescence analysis (Fig. 4a, c, and data not shown). At 12 hpT, low levels of p-S6 were detected mainly in meningeal cells and in ventral cells that probably correspond to motoneurons and Mauthner cells (Fig. 4b). This was followed by the detection of higher levels at 21 hpT in similar cells, but now also in more lateral neurons, and the apical pole of ependymal cells in ventricular zone (Fig. 4d). Longitudinal sections in the region surrounding the injury site showed the presence of p-S6 in cells lining the ependymal canal (Fig. 4e) and in cells populating the ablation gap (Fig. 4l). Western blot analysis confirmed peak levels of p-S6 signal at 1 dpT and a decrease at 2 and 4 dpT (Fig. 4f and see Supplementary Fig. S3a).

Similarly, p-4EBP1 signal was absent in sham operated and uninjured animals (Fig. 4g, data not shown), while detected at 12 and 21 hpT, although it was found mainly in the cells of the ependymal layer, and in meningeal cells (Fig. 4h, i). To further evaluate which cells activate mTORC1 signaling, we performed double immunostaining analysis with Sox2, a marker of NSPC. We found that many Sox2^+^ cells in the ablation gap activate phosphorylation of S6, and higher levels of p-S6 were observed closer to the injury site (Fig. 4j–l).

### Early inhibition of mTORC1 signaling impairs spinal cord regeneration

**Fig. 5:**
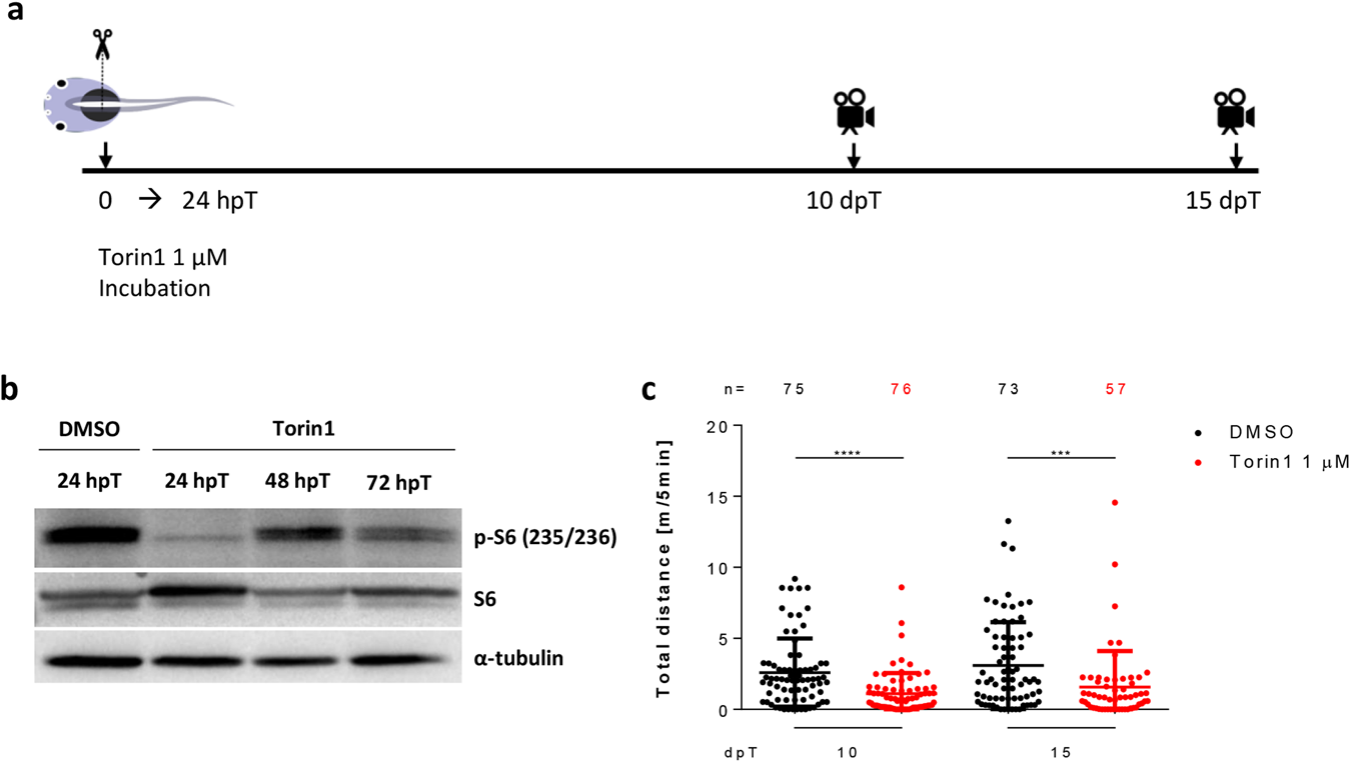
Early inhibition of mTOR signaling impairs swimming recovery. **a** Scheme illustrating the experimental timeline. The mTOR pathway was inhibited incubating animals with Torin1 1 µM during the first 24 hpT, and swimming distance was recorded at 10 and 15 dpT. **b** Western blot for S6 and p-S6 in total protein extracts obtained from spinal cords isolated from *X. laevis* incubated immediately after transection for 24 h with DMSO (vehicle) and Torin1 (drug) and collected at 24 hpT for vehicle treatment and at 24, 48, and 72 hpT for drug treatment. **c** Dotplot of swimming distances of animals treated with vehicle (black dots) and 1 µM Torin1 (red dots), measured at 10 and 15 dpT. Data indicate the mean ± SEM, from three independent experiments. ***P < 0.001, ****P < 0.0001, from a Mann–Whitney *U*-test comparing Torin1-treated animals to control, at each time point. All the blots included in this figure were derived from the same experiment and were processed in parallel.

To test the function of mTORC1 through spinal cord regeneration, we incubated the animals during the first 24 hpT with Torin1 or Rapamycin, two inhibitors of the pathway (Fig. 5a). Western blot analysis against p-S6 and p-4EBP1 showed a strong inhibition at 1 dpT (Fig. 5b and see Supplementary Fig. S3b). Although some activation of the pathway is observed at 2 and 3 dpT, it never attains the levels observed in untreated animals (Fig. 5b). Following the same inhibitory paradigm (Fig. 5a), we found a significant reduction in the recovery of swimming compared to controls at 10 and 15 dpT (Fig. 5c and Supplementary Fig. S3c), suggesting that early mTOR signaling is necessary for proper spinal cord regeneration. No differences were found for sham animals treated with Torin1 or Rapamycin (Supplementary Fig. S3d–f), in agreement with findings showing no effect of Rapamycin in mice swimming abilities in behavioral tests^35,36^.

**Fig. 6:**
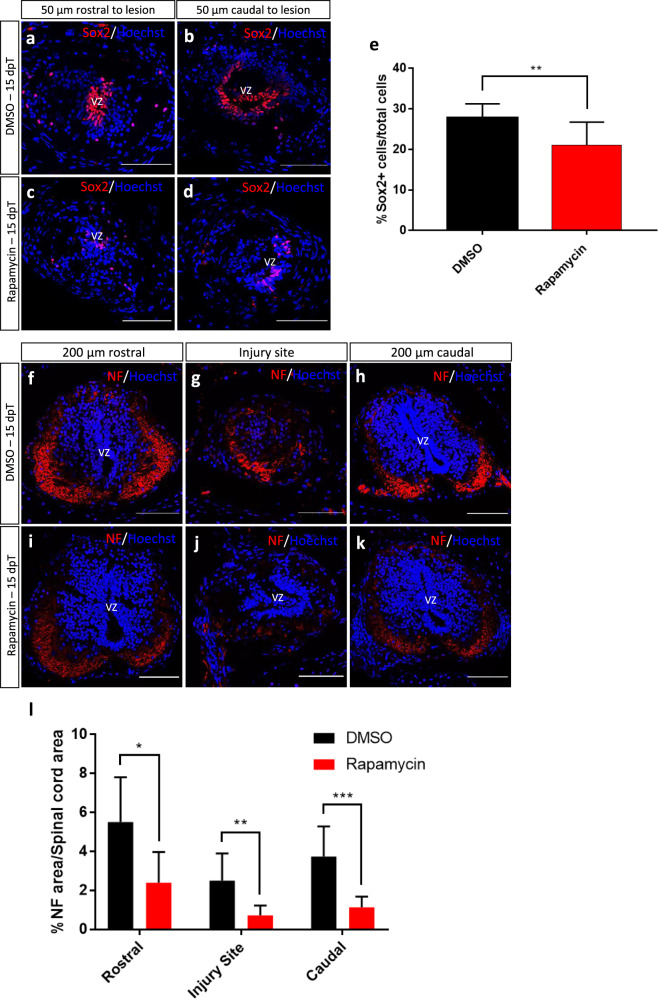
Effects of early inhibition of mTOR pathway in Sox2 and neurofilament levels. **a**–**d** Representative images of transversal cryosections of the spinal cord at 50 µm rostral (left images) and 50 µm caudal (right images) to the injury site. Samples were obtained at 15 dpT from animals treated for 24 h with (**a**, **b**) vehicle or (**c**, **d**) Rapamycin, stained for the NSPC marker Sox2 and Hoechst. **e** Quantification of the percentage of Sox2^+^ cells over total cells. **P < 0.01, from a *t* test comparing Rapamycin to vehicle treatments. *n* = 3 per condition. **f**–**k** Representative images of transversal cryosections of the spinal cord, 200 µm rostral (left images), injury site (middle images), and 200 µm caudal (right images) to the injury site. Samples were obtained at 15 dpT from animals treated for 24 h with (**f**–**h**) vehicle or (**i**–**k**) Rapamycin, stained for the neuronal marker neurofilament (NF) and Hoechst. **l** Quantification of the percentage of spinal cord area positive for NF staining over spinal cord area, in the injury site as well as 200 µm rostral and caudal to the injury site. *t* test. *P < 0.05, **P < 0.01 and ***P < 0.001, from a *t* test comparing Rapamycin to vehicle treatments. *n* = 3 per condition. VZ ventricular zone. Immunofluorescences were performed in two or three biological replicates. Scale bar = 100 μm.

To evaluate a possible mechanism of action to explain the effects of inhibiting the mTOR pathway, we performed immunofluorescence against Sox2 and neurofilament (NF), markers of NSPCs and axons respectively. A clear reduction in the number of Sox2^+^ cells present in the ablation gap was detected at 15 dpT (Fig. 6a–e). In addition, a significant decrease in the numbers of axons in the rostral, caudal, and injury site was observed in treated animals also at 15 dpT (Fig. 6f–l). From these results, we hypothesize that the effects of mTORC1 inhibition in spinal cord regeneration could be explained at different levels, including (i) inhibition of NSPC proliferation, (ii) inhibition of intrinsic programs needed for axon regeneration, (iii) modulation of inflammatory responses to injury, and/or (iv) regulation of cell survival and cell death after injury.

### Activation of the mTORC1 signaling pathway is necessary for proliferation of NSPCs

**Fig. 7:**
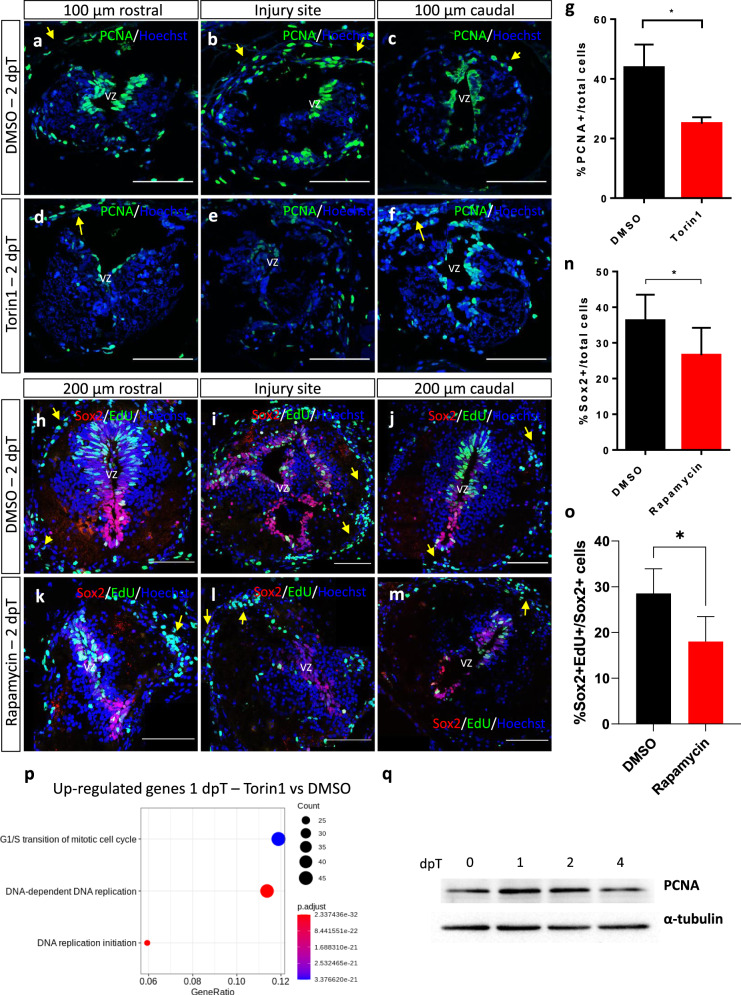
mTOR inhibition reduces cell proliferation and PCNA levels. **a**–**f** Representative images of transversal cryosections of the spinal cord, 100 µm rostral (left images) and 100 µm caudal (right images) to the injury site, obtained at 2 dpT from animals treated with **a**–**c** vehicle or **d**–**f** Torin1, stained for the proliferation marker PCNA and nuclear marker Hoechst. **g** Quantification of the percentage of PCNA^+^ cells over total cells. *P < 0.05, from a *t* test comparing Torin1 to vehicle treatments. *n* = 3 per condition. **h**–**m** Representative images of transversal cryosections of the spinal cord, 200 µm rostral (left images) and 200 µm caudal (right images) to the injury site, obtained at 2 dpT from animals treated with (**h**–**j**) vehicle or (**k**–**m**) Rapamycin, stained for the NSPC marker Sox2, EdU, and Hoechst. **n** Quantification of the percentage of Sox2^+^ cells over total cells. *P < 0.05, from a *t* test comparing Rapamycin to vehicle treatments. *n* = 3 per condition. **o** Quantification of the percentage of EdU^+^/Sox2^+^ cells over Sox2^+^ cells. *P value < 0.05, from a *t* test comparing Rapamycin to vehicle treatments. *n* = 4 per condition. **p** Three principal biological process downregulated at 1 dpT after treatment with Torin1, including a large component of genes related to G1/S transition of cell cycle and DNA replication. **q** Western blot for PCNA in total protein extracts obtained from spinal cords isolated from *X. laevis* at 0, 1, 2, and 4 dpT, α-tubulin was used as a loading control. Yellow arrow: meningeal cells, vz: ventricular zone. Scale bar = 100 μm. All the blots included in this figure were derived from the same experiment and were processed in parallel.

Based on our previous results demonstrating that activation of Sox2^+^ NSPCs are required for spinal cord regeneration in *X. laevis*^19^, we tested the effects of mTOR inhibition in the activation of NSPC. For this, we first studied the expression of PCNA, a marker of DNA replication. Incubation with Torin1 during the first 21 hpT resulted in a strong reduction in the number of cells expressing PCNA at 2 dpT (Fig. 7a–g). This reduction was mainly observed in the cells lining the central canal and the injury zone, but the inhibitory effect was less pronounced in the meningeal cells.

To further study the effects on NSPC proliferation, we provided a pulse of 5-ethynyl-2′-deoxyuridine (EdU) followed by double immunofluorescence against Sox2 and EdU. We found that mTORC1 inhibition significantly decreased the number of Sox2^+^ cells in the lesion site and sections close to it (Fig. 7h–n). In line with that, we also observed a significant decrease in the amount of EdU^+^/Sox2^+^ cell in the same zone (Fig. 7o).

Considering that our GO analysis also identified the regulation of genes involved in apoptosis after injury, we performed terminal deoxynucleotidyl transferase-mediated dUTP-fluorescein nick end labeling (TUNEL) analysis at 1 dpT. We found that inhibition of mTOR resulted in increased levels of TUNEL^+^ cells (see Supplementary Fig. S4). This result is in line with our bioinformatics analysis, suggesting a rapid downregulation of cell death after injury.

These results showed that not only mTORC1 activation is necessary for proliferation of NSPCs but also the decrease of Sox2 cells close to the lesion site suggest a possible role in cell migration and cell death.

### Effect of mTOR inhibition in early gene expression after SCI

It has been described that mTOR pathway not only regulates protein synthesis but that its inhibition also affects gene transcription^37^. Thus, the effect of mTORC1 inhibition in the transcriptome deployed after SCI was evaluated. For this, animals were incubated with Torin1 or vehicle during the first 24 hpT, spinal cords were isolated at 1, 2, and 4 dpT, and mRNA-seq analysis was performed. Differential gene expression was calculated using edgeR; we consider as differentially expressed those genes with a fold change ≥2 (upregulated) or ≤2 (downregulated) and also having a *p* value ≤ 0.01. There were 787, 356, and 212 genes identified as differentially expressed at 1, 2, and 4 dpT, respectively (see Supplementary Fig. S5a–c and Table S7).

We found that a large group of genes related to G1/S transition of mitotic cell cycle and DNA replication initiation were downregulated at 1 dpT in animals treated with Torin1 (Fig. 7p), which is consistent with the results observed for PCNA and EdU^+^/Sox2^+^ (Fig. 7a–o). Of note, a negative fold change of 2.7 and 4.1 for mRNA of both PCNA homeolog genes was observed in the mRNA-seq analysis, while no differential gene expression was observed for sox2 gene (data not shown). This is in agreement with the finding that PCNA protein levels increase at 1 and 2 dpT in animals without mTORC1 inhibition (Fig. 7q and see Supplementary Fig. S5d). Furthermore, we also found a significant upregulation of casp3 mRNA at 1 dpT (fold change of 2.9, see Supplementary Table S7), which is in agreement with the TUNEL analysis described above.

Among the upregulated genes at 1 dpT, we found an enrichment in genes associated with macrophage chemotaxis and positive regulation of IL-4 production, suggesting a strong regulation of the immune response (see Supplementary Information Fig. S5e). At 2 and 4 dpT, an enrichment of genes (upregulated or downregulated) associated with the immune response and other biological processes was also observed, suggesting that an early inhibition of mTORC1 could have a prolonged effect in many process involved in spinal cord regeneration (see Supplementary Information Fig. S5f–i).

All together, these results show that an early and transitory inhibition of mTORC1 have a negative effect on NSPC proliferation. We suggest that these could explain the negative effects in swimming recovery when mTORC1 is inhibited. In addition, this analysis suggests an early regulation of cell death and immune response by mTORC1 that could also contribute to the effects on spinal cord regeneration.

## Discussion

**Fig. 8:**
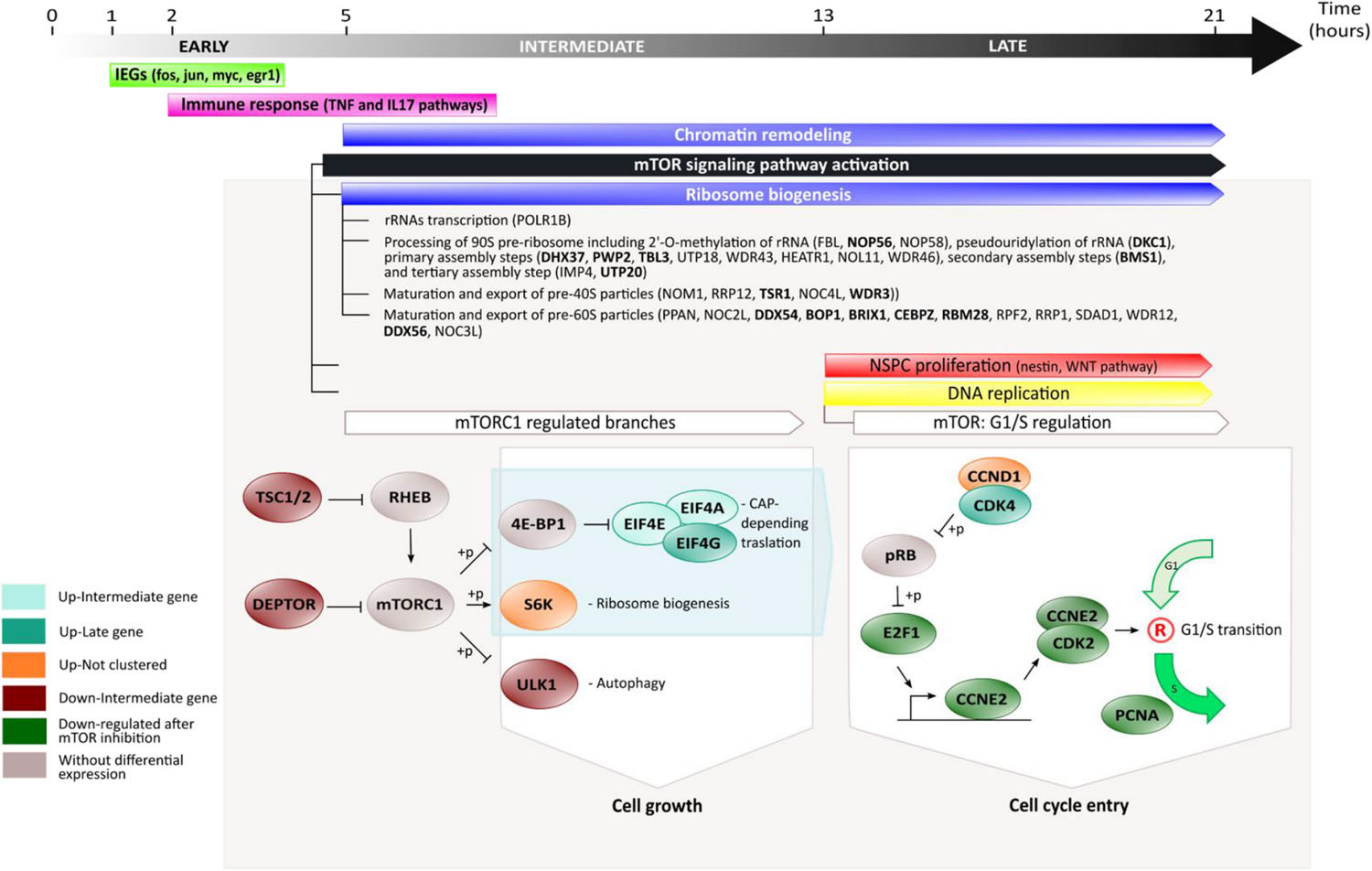
Summary model of early transcriptional response after SCI in *X. laevis*. In early modules, around 2 hpT, occur a rapid and transitory activation of IEGs (module E1, green yellow in Figs. 2a and 3) followed by immune response genes (module E2, magenta in Figs. 2b and 3). Posteriorly, in intermediate modules, around 5 hpT, an increase in genes associated with chromatin remodeling is observed (modules I3, blue in Figs. 2e and 3), as well as regulation of several biological events, including activation of mTORC1 (module D1, black in Figs. 2f and 3). Apparently, mTORC1 would act as key regulator over important cellular processes, such as protein synthesis regulation and ribosome biogenesis (module I3, blue in Figs. 2e and 3) observing a strong upregulation of RBFs linked to self-renewal of neuroblast (highlighted in bold), and NSPC proliferation (module L2, red in Figs. 2i and 3) and DNA replication (module L3, yellow in Figs. 2j and 3) in late modules, regulating the G1/S transition.

Here we performed a high-resolution expression profiling analysis of the first 21 h after spinal cord transection, which led to the identification of the transcriptome changes deployed during this early response, allowing the delineation of the regenerative program deployed after spinal cord transection, particularly those steps involved in the activation of NSPC (Fig. 8), and the identification of the mTORC1 pathway as a key component in the activation of this process.

A first step in the response to any insult is the rapid activation of primary response genes (PRGs)^24^. PRGs are classified into two groups of genes: IEGs, which reach their peaks within 30–60 min after the stimulus, and delayed early genes with their maximum expression at about 120 min after stimulus^38^. The change in expression levels of these genes is characterized by no need of protein synthesis. IEGs correspond mainly to transcription factors, commonly activated in response to different stimulus, and responsible for remodeling the cellular transcriptome, generating the onset of secondary response genes (SRGs), which induce a specific response to detected stimulus^25,39^. Interestingly, almost half of the genes grouped in module E1 correspond to IEGs, including fos, jun, and myc (Fig. 8), suggesting a role for them in the regenerative response. Similar results have been reported in planaria, showing that the activation of IEGs within the first 30 min after wounding is necessary for proper regeneration^40^. Furthermore, the Echeverri group recently showed that Fos can have opposing effects over spinal cord regeneration on axolotl, suggesting that the presence of different dimerization partners can modulate distinct regenerative responses^41^. Regarding SRGs, the intermediate and late modules depicted here have kinetics of expression with patterns similar to those described for SRG, which are usually involved in the maintenance of an effective response to the corresponding stimulus^24,42,43^. Our findings suggest a strong conservation of the kinetic reported for these first responders, even in a complex process such as spinal cord regeneration. Future studies are necessary to confirm that genes in E1 correspond to IEG, specifically validating that they do not need protein synthesis for their activation, as well as to identify their function in the onset of this regenerative program.

Immediately after 2 hpT, an increase in genes associated with immune cell migration was detected, suggesting an early role for innate immune cells. These cells are known to play a role in early stages of wound healing, and are required for proper regeneration principally through cytokine secretion, and favoring the clearance of death cells and deposited material^44^. In agreement with the above, among the intermediate module I1 at around 4 hpT, a big component of genes associated with chemotaxis and immune response was detected, suggesting that innate immune response plays an important and sustained role during the first few hours after SCI (Fig. 8), similar to what has been reported in zebrafish^45^. Furthermore, mRNA-seq assays at 1 dpT using Torin1 reported an increase in genes associated with macrophage migration and positive regulation of IL-4 production (Fig. S5e), suggesting that mTORC1 is also involved in the regulation of early innate immune response.

At 5–6 hpT, we observed an enrichment of components of three interconnected processes: (1) downregulation of negative regulators of mTOR (*tsc2* and *deptor*, Fig. 8), (2) downregulation of positive regulators of autophagy (*ulk1*, Fig. 8), and (3) upregulation of genes associated with ribosome biogenesis (*rps6kb1*, RBFs, and components of EIF4F complex, Fig. 8). These data suggest that the activation of mTOR promote a cell growth stage that is necessary for cell cycle entrance^46^, which in our context could be associated with NSPC activation.

Previously, it has been reported that translational activation is one of the earliest events in transition from quiescence to activation of NSPC^47^ and mTORC1 is a key regulator of ribosome biogenesis and protein translation^48,49^. In intermediate modules, an upregulation of RBFs and protein synthesis was observed, suggesting that this is a process sustained in time and that mTOR activation is maintained, which is supported by high levels of p-S6 observed at 21 hpT (Fig. 4d–h). In this context, the identified RBFs are involved in different steps of ribosome biogenesis and several of them have been reported as necessary for self-renewal in neuroblasts (Fig. 8, highlighted in bold)^30^, which would support in part the association between these processes and NSPC activation in our model.

Even more, at 13 hpT, the time at which L2 and L3 modules reach a peak, we identify an increase in genes associated with stem cell proliferation and neurogenesis like nestin and components of WNT pathway (wnt7b, fzd2, fzd3, and ctnnb1). Also, key genes involved in transition from G1 to S phase as cdk4, e2f1, cdk2, and ccnd1 (Fig. 8) were identified, suggesting a successful progression through cell cycle in NSPC during the first day after lesion. This data fits with our previous finding showing that NSPC start to proliferate between 1 and 2 dpT and are necessary for spinal cord regeneration^18–20^.

In agreement with the model depicted above, we detected an activation of mTORC1 at 12 hpT, attaining a maximal peak at 1 dpT, and decreasing significatively with respect to control at 4 dpT. The pathway is mainly activated in NSPC, meningeal cells, and ventrolateral neurons. This supports a possible activation of mTORC1 leading to an increased rate of protein synthesis in NSPC, which could be necessary for NSPC activation^46^, as well as regulating cap-dependent translation of pro-neurogenic mRNAs^50^. In line with this, we found that early inhibition of the mTOR pathway reduce proliferation of NSPCs.

This is further supported with the finding that mTOR inhibition resulted in the downregulation of genes associated with the cell cycle G1/S transition at 1 dpT, including genes associated with cell cycle checkpoint, *ccne2* and *cdk2*, as well as its transcriptional regulator, *e2f1* (Fig. 8). This is in accordance with a previous report showing that mTORC1 is able to regulate *ccnd* and *ccne*, translationally and transcriptionally, both directly implicated on the G1/S transition^51^. As a consequence of the latest, a downregulation of gene expression levels of PCNA was observed after mTORC1 inhibition at 1 dpT, which is concomitant with reduced levels of cells in proliferative state close to injury site at 2 dpT in animals treated with Torin1 (Fig. 7a–g). It is important to consider that PCNA, a key protein during DNA replication, is significantly induced during the first 2 days of spinal cord regeneration (Fig. 7q). Altogether, our data suggest that mTORC1 plays a key role during early stages of spinal cord regeneration in *X. laevis*, activating NSPC proliferation through translational activation, and later mediating G1/S transition. This role in NSPC activation probably explains in part the disruption of functional recovery.

Many questions remain open regarding the role of mTORC1 in NSPC activation, particularly the identification of the upstream regulators that trigger mTOR signaling after injury, and the specific RNAs and/or proteins regulated by this pathway in order to regulate NSPC proliferation^52^. With respect to upstream regulators for mTOR signaling, gradients of Ca2^+^ and reactive oxygen species are deployed minutes after tail amputation in *Xenopus*^53,54^; both diffusible signals have been previously reported as activators of phosphoinositide-3 kinase (PI3K)/Akt/mTOR pathway^55,56^, which could be correlated with its early activation. In addition, transcriptomic data reported a dual activation of PIK3R3, a regulatory subunit of PI3K that is associated with proliferation^57^. This gene showed an early and transitory activation followed by a late and sustained activation, which was also shared by PI3KR2-like, another regulatory subunit of PI3K. Also a late downregulation of PIK3IP1, a negative regulator of PI3K pathway, was observed. Thus, a possible role of PI3K upstream of mTOR pathway during spinal cord regeneration would be interesting to analyze in future research.

Another question that remains open is about the role of mTORC2 in early stages of spinal cord regeneration. A downregulation of mSin1 (also known as MAPKAP1, mitogen-activated protein kinase-associated protein 1) was observed in our transcriptomic data (see Supplementary Table S2), and an increase in the phosphorylation levels of S6 protein, which is correlated with an activation of S6K, was detected experimentally (see Fig. 4). Both observations have been associated with a reduced activity of mTORC2^58,59^, suggesting low levels of mTORC2 activity during early stages of SCI in *X. laevis*, and consequently that most of the effect of the inhibitors is explained because of the impact on mTORC1.

Finally, with respect to other implications of mTOR, it is known that mTORC1 pathway activation promotes axon regeneration after optic nerve injury^60^. mTORC1 could play a similar role in *Xenopus* spinal cord regeneration, implicating a conserved role of mTOR as a component of the intrinsic mechanism of axon regeneration, and could explain the decrease in the number of axons crossing the injury site observed after mTOR inhibition. A possible role of mTORC1 in axon regeneration could be another explanation for the diminished swimming recovery, prompting to the future testing of the effect of this pathway in axonal regeneration in this model system.

## Methods

### Animal husbandry

*X. laevis* tadpoles were generated by natural mating and cultured as described previously^17^. Animals were grown until Nieuwkoop and Faber stages 49–51 for experiments. Animal procedures were approved by the Scientific Ethics Committee for the Care of Animals and Environment of the Pontificia Universidad Católica de Chile.

### Experimental conditions and surgical procedures

Three experimental conditions were used: transected, sham, and uninjured animals. Transected and sham procedures were performed according to previous publications^17,20^. Briefly, animals were anesthetized for 2 min on 0.02% MS222, followed by a dorsal incision at mid-thoracic level, cutting the skin and muscle to expose the spinal cord for sham animals, and a second step in which the spinal cord was transected at the same anatomical level interrupting all ascending and descending axonal tracts for transected animals. After surgery, animals were left on 0.1× Barth (8.9 mM NaCl; 102 μM KCl; 238.1 μM NaHCO_3_; 1 mM 4-(2-hydroxyethyl)-1-piperazine-ethane sulfonic acid; 81.14 μM MgSO_4_; 33.88 μM Ca(NO_3_)_2_; 40.81 μM CaCl_2_, pH 7.6) with antibiotics (100 μg/ml penicillin and 100 μg/ml streptomycin) until spinal cord isolation, as previously described^17^.

### Spinal cord isolation

For each experimental point, 10–12 spinal cords were isolated to obtain enough material for sequencing. For this, a region of 8–10 mm was isolated from the spinal cord caudal to the injury site from transected animals and a similar region from control animals^17,20^. Proper isolation of the spinal cords, at a precise time, is needed for the success of the time-series experiment. Thus, sampling for transected and sham conditions were composed of three steps: (1) 24 h before surgery, animals were accurately selected, staged to have enough stage 50 tadpoles, and maintained in 0.1× Barth; (2) at surgery time, animals for each condition were split in six different pools, which were composed by 2 + 2 *N* animals each one, where *N* correspond to time points in the time series, and surgery was performed to each pool every 5 min, alternating pools that were transected and sham-operated; (3) once the proper time for each pool was completed, two spinal cord were isolated for each one to complete 10 spinal cords (series S1, S1’, S2, and S2’) or 12 spinal cords (S3, S4, S5, S6, S3’, S4’, S5’, S6’, S7, and S8). These spinal cords were immediately stored in RNAlater. Uninjured samples were obtained through a similar 3-step process, but no previous surgery was performed. For mTOR Inhibition mRNA-seq, animals were selected and maintained in 0.1× Barth with antibiotics. The next day, animals were transected and divided into two groups, one was incubated for 24 h in 0.1× Barth with antibiotics + 1 µM Torin1 and the other in 0.1× Barth with antibiotics + 0.05% dimethyl sulfoxide (DMSO). At 1, 2, and 4 dpT, spinal cords from 15 animals of each group were isolated and stored immediately in RNAlater.

### Library preparation and mRNA-seq

Total RNA extractions were performed using the RNeasy Mini Kit (Qiagen, MD, USA), including a DNase I treatment to avoid genomic DNA contamination^20^. RNA concentration and purity and RNA integrity number were measured using Nanodrop and Bioanalyzer 2100, respectively. PolyA+ RNA-seq libraries were prepared for each time series with the TruSeq RNA Library Prep Kit v2 (Illumina) using between 120 and 500 ng of total RNA as starting material. For mTOR inhibition mRNA-seq, libraries were constructed using 1 µg of total RNA as input. The libraries’ quality was determined using Bioanalyzer 2100 and a mean size of 300 bp was obtained for all libraries. Posteriorly, time-series and mTOR inhibition libraries were sequenced using the HISeq-2000 and HISeq-4000 platforms (Illumina).

### Bioinformatics analysis and data consistency

Sequencing quality was determined using the FASTQC software^61^, with 96% of the bases having a quality score >Q30 (mean quality score Q37.1; Table S1). Libraries were mapped to the model transcriptome v9.1 of *X. laevis* (Xenbase)^62,63^ using Bowtie-RSEM for time-series mRNA-seq^64,65^ and Bowtie2-RSEM for mTOR inhibition mRNA-seq^65,66^, using default parameters established by RSEM. Genes with five or more counts in at least four temporal points were defined as genes detected consistently in time-series mRNA-seq.

Reproducibility among time-series mRNA-seq samples was evaluated by Spearman correlation coefficient over gene counts normalized with variance stabilizing transformation (VST) method of DESeq2^67^. Gene counts normalized with VST was also used as input for batch effect correction using ComBat function included in SVA package, as it has been demonstrated as a suitable adjust for known batches^68^.

To ensure that the sampling kept an adequate tracking of the biological processes within each series, and among the different series, the consistency of the data was evaluated. A high degree of internal consistency for neighboring points within each time series, in the transected condition, was observed using Spearman correlation coefficients (0.99 ± 0.006; Supplementary Fig. S6a). The correlations calculated for neighboring points were very similar to the ones obtained for the biological replicates at 0 hpT, included in series 1 and 2, which were 0.98 and 0.99, respectively (Supplementary Fig. S6a), indicating that neighboring points could function as pseudo-replicates in high-resolution time series^21^. Similar results were observed for correlation coefficient of neighboring points in sham and uninjured conditions, 0.99 ± 0.007 (Supplementary Fig. S7a) and 0.98 ± 0.017 (Supplementary Fig. S8a), respectively.

Furthermore, the correlation coefficient between overlapping points was calculated in different series, and although still strong, they were found to be slightly lower than those calculated for the biological replicates and pseudo-replicates (0.96 ± 0.018; Supplementary Fig. S6b). Something similar was obtained when the 0 hpT time points for each series were calculated (0.95 ± 0.022; Supplementary Fig. S6b, inset). Considering that the expression at 0 hpT corresponds to the basal expression level for each time series, we reasoned that the multiple sampling processes and different batches of animals contributed to generate a batch effect, which could explain the differences on gene expression levels for basal and overlapping points during construction of some gene expression profiles (Supplementary Fig. S9a–d). Combat function of the SVA package was applied to all our data, to correct these batch effects, improving the correlation coefficients of the basal (0 hpT) and overlapping points, 0.99 ± 0.004 and 0.99 ± 0.003, respectively. After these corrections, a better fit was observed between the time series, especially for those genes particularly affected by batch effects (Supplementary Fig. S6c and S9a’–d’).

In case of sham samples, we observed an improvement in pairwise correlation for overlapping points after batch effect correction from 0.97 ± 0.014 (Supplementary Fig. S7b) to 0.99 ± 0.003. While for the uninjured condition, similar results were reported before (0.98 ± 0.012, Supplementary Fig. S8b) and after (0.98 ± 0.005) batch effect correction, probably because this condition was just sampled in two time series, and the batch effect was slightly higher than in the transected condition. Finally, for a better comparison among conditions, a three-dimensional principal component analysis plot is included (Supplementary Fig. S8c), showing a strong difference in differentially expressed genes between the transected and control conditions, which increases with time, thus validating the sampling and data processing.

### Differential expression analysis

For time-series mRNA-seq, differential gene expression analysis was performed using Gaussian Processes, as previously reported^21^, over the list of genes consistently detected. Briefly, for each gene, two possible hypotheses were tested and compared: a null hypothesis in which the gene expression profiles are best fitted by one single Gaussian Process model, non-differential gene expression, and an alternative hypothesis where the gene expression profiles are better fitted by two different Gaussian Process models, temporal differential gene expression. To choose between both hypotheses, the log marginal likelihood of each one was compared using BIC, a BIC > 0 indicates a preference for the alternative hypothesis of two Gaussian Process models. Two different comparisons were done, transected-vs-sham and transected-vs-uninjured, and were selected for further analysis those genes with BIC ≥ 10 for both comparisons because these were considered as strongly deployed in response to SCI. This cut-off has been described previously as a strong evidence for alternative hypothesis in model selection when BIC is used^23^. For mTOR inhibition mRNA-seq, differential expression analysis was performed following a protocol for experiments without replicates using edgeR^69^. Briefly, gene counts were normalized based on library size and later differential gene expression was tested applying the function exactTest, using a biological coefficient of variation of 0.2. To determine differential gene expression, |Log2 (fold change)| ≥ 1 and *p* value ≤ 0.01 were used as cut-off.

### Weighted gene co-expression network analysis

WGCNA^70^ was performed over transected data corresponding to genes differentially expressed with a BIC ≥ 10. Initially, a similarity matrix was calculated for gene expression profiles using biweight midcorrelation (bicor) coefficient, which is less sensible for outliers^71^. Posteriorly, to determine connection strengths between network nodes, a signed hybrid adjacency matrix was calculated, based on correlation measures using a soft-thresholding power equal to 15 (*β* = 15) which was estimated by *pickSoftThreshold* function to satisfied the scale-free topology property of the co-expression network^70^. Then, adjacency matrix was transformed to a topological overlap matrix, and its corresponding dissimilarity matrix was used as input for the hierarchical clustering algorithm to identify co-expression modules on the network^70^. Posteriorly, a change point analysis was performed over representative expression profile of each module using Bayesloop, to determine an approximate time at which gene expression is changing in each co-expression module^72^.

### GO and KEGG pathway enrichment analysis

Protein-coding sequences in the model transcriptome of *X. laevis* were first mapped onto the NCBI non-redundant protein database for mouse, human, zebrafish, chicken, fly, and worm using BlastP (-evalue e-5 -max_target_seqs 5)^73^, and InterProScan was used to identify protein domains^74^. Subsequently, the data were integrated into Blast2GO, and GO terms were assigned to each gene^75^. An enrichment analysis was performed to each module using clusterProfiler^76^, specifically for genes with Module Membership ≥0.6. A *p* value cut-off of 0.01 in Fisher’s Exact Test was chosen for enrichments. To reduce redundancy in the enrichment analysis of time-series mRNA-seq, GO terms that meet at least one of the following criteria were chosen: (1) ≥5 genes in co-expression module are associated with this, (2) at least 15% of genes in co-expression module are associated with this, or (3) at least 15% of genes used as background in enrichment analysis that are associated with this are present in co-expression module. Finally, GO terms were clustered based on semantic similarity and genes associated with them, using DBSCAN algorithm, and representative GO term in each cluster was selected based on the highest *x-*value, which was defined as product of –Log10 (*p* value) calculated for GO term and the sum of BIC for genes associated with this, $x\mbox{-}{\rm{value}}_{{\rm{GO}}\;{\rm{term}}} = - {\rm{Log}}(p\;{\rm{value}})_{{\rm{GO}}\;{\rm{term}}}\times{{{\mathrm{{\Sigma}}}}}{\rm{BIC}}_{{\rm{GO}}\;{\rm{term}}}$. For mTOR inhibition mRNA-seq, three GO terms with the biggest gene ratio in each enrichment were visualized using clusterProfiler.

For KEGG pathway analysis, protein-coding sequences in model transcriptome of *X. laevis* were also mapped onto the NCBI non-redundant database for human proteins using BlastP (-evalue e-5 -max_target_seqs 5). Posteriorly, these annotations were used to calculate an enrichment against human KEGG pathway database using clusterProfiler and Fisher’s Exact Test with false discovery rate cut-off of 0.05^76^.

### Immunofluorescence

Animals were cryosectioned as previously described^17^. Briefly, animals were sacrificed using 0.02% MS222 and fixed with 4% paraformaldehyde (PFA) in phosphate-buffered saline (PBS) at room temperature (RT) for 2 h or at 4 °C overnight (ON). After fixation, excess of PFA was removed, samples were dehydrated in increasing concentration of PBS–sucrose solution (5, 10, and 20%), embedded in optimal cutting temperature compound, and frozen using liquid nitrogen. Later, samples were cryosectioned (10 µm) and tissue sections were permeabilized for 10 min using 0.02% Triton in PBS, blocked for 30 min using bovine serum albumin (BSA) or goat serum; an incubation with methanol for 10 min at −80 °C was optionally performed, in order to reduce tissue auto-fluorescence (Table S8), followed by an ON incubation at 4 °C with primary antibodies. Posteriorly, tissue sections were thoroughly washed with 0.02% Triton in PBS and incubated with Alexa-conjugated secondary antibodies for 2 h at RT. Finally, after washing with 0.02% Triton in PBS, nuclei were stained for 5 min using Hoechst (1:10,000, Thermo Fisher Scientific, Waltham, USA) in 0.02% Triton in PBS. Details of antibodies and concentrations used are reported in Table S8. Immunofluorescence images were captured with a Fluoview FV10i confocal microscope (Olympus). Cell quantification and NF-stained area was performed using the Fiji/ImageJ software. For Sox2, PCNA, and Sox2/EdU analysis, we counted the positive cells over total cell number contained in a defined area of the spinal cord (200 µm rostral and 200 µm caudal to the injury site). For NF analysis, we determined the ratio between pixels stained for NF and pixels in the total area of spinal cord. In both cases, statistical differences were calculated using *t* test.

### TUNEL

Cell death was evaluated in spinal cord longitudinal sections using DeadEnd™ Fluorometric TUNEL System (TUNEL, Promega Corporation, WI, USA), following the manufacturer instructions. Briefly, 24 h after transected animals were incubated with DMSO or 1 µM Torin1, they were sacrificed and fixed with 4% PFA in PBS at 4 °C ON, and samples were dehydrated, frozen, and cryosectioned (10 µm). Spinal cord sections were permeabilized with 20 µg/ml Proteinase K, followed by fixation with 4% PFA, and incubated with the TUNEL mixture for 1 h at 37 °C. Finally, sections were stained with Hoechst 33342 (1:10,000) for 10 min and mounted with vectashield (Vector Laboratories, Burlingame, USA). Images were captured with an Olympus BX51 microscope. The number of TUNEL-positive nuclei were quantified in a defined area of the spinal cord (300 µm rostral and 300 µm caudal to the injury site).

### Western blot

Spinal cords from 12 animals were isolated at the desired time point and manually homogenized in RIPA buffer including protease inhibitor. Extracted proteins were separated by 10% sodium dodecyl sulfate–polyacrylamide gel electrophoresis, and transferred to polyvinylidene difluoride membranes. Primary and secondary antibodies were incubated separately in 3% BSA in TBS-T and revealed with SuperSignal West Pico Chemiluminescent Substrate in the iBright FL-1000 (Invitrogen). Details of antibodies and concentrations used are reported in Table S8. All blots or gels were derived from the same experiment and processed in parallel.

### mTOR inhibition

Transient mTOR pathway inhibition was performed exposing the animals for 24 h to 10 µM Rapamycin (553210, Sigma-Aldrich) or 1 µM Torin1 (ab218606, abcam) in 0.1× Barth with antibiotics, while control animals to 0.1 or 0.05% DMSO in 0.1× Barth, respectively, immediately after transection. After drug exposure, animals were maintained in 0.1× Barth with antibiotics during the course of the experiments.

### Swimming recovery assay

Free swimming distance, in animals treated with mTOR inhibitors, was evaluated by video-tracking at 10 and 15 dpT as previously reported^19^. Briefly, animals were recorded for 5 min with a video camera and the swimming paths were tracked by ANY-maze (Stoelting Co, Wood Dale, IL). Posteriorly, swimming paths were corrected using Resampling Trajectories of traja (Python package), to avoid overestimations induced by animal movements that do not generate displacement, and finally statistic differences between groups were tested using Mann–Whitney *U*-test for non-parametric data.

### Proliferation assay

Cell proliferation was evaluated using a pulse-chase assay with thymidine analog EdU. Thirty-two hpT animals were injected with 1 µL EdU dilution at a concentration of 5 mg/ml in 0.8× PBS and 16 h later were sacrificed, fixed, and processed as previously described^17^.

### Reporting summary

Further information on research design is available in the Nature Research Reporting Summary linked to this article.

## Supplementary information

Supplementary Information

Reporting Summary

## Data Availability

mRNA-seq data are available on NCBI Gene Expression Omnibus (GEO) database repository (accession number: GSE165343; token: ktwxsgagnrcpdkr). Other data or reagents are available upon request from the corresponding author.
