# Supplementary material for: Analysis of the early response to spinal cord injury identified a key role for mTORC1 signaling in the activation of neural stem progenitor cells
Source: NPJ Regen Med. 2021 Oct 22;6:68. doi: 10.1038/s41536-021-00179-3 (PMC8536777; doi:10.1038/s41536-021-00179-3)
Supplement: Supplementary file 1 — Supplementary Information [file 41536_2021_179_MOESM1_ESM.pdf]

Supplementary Figures

Supplementary Figure S1. Representation of module Eigengenes.

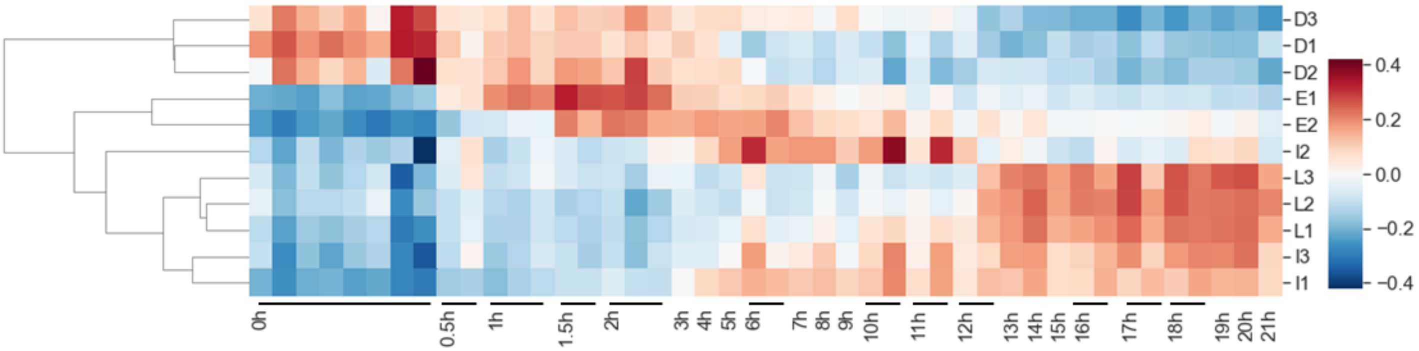

A dendrogram is used to cluster the module Eigengene for the 11 co-expression modules identified in the Weighted Gene Coexpression Network Analysis. Module eigengenes are also depicted using a heatmap for a better comprension of their kinetics.

Supplementary Figure S2. Key components of mTOR signaling pathway are early regulated.

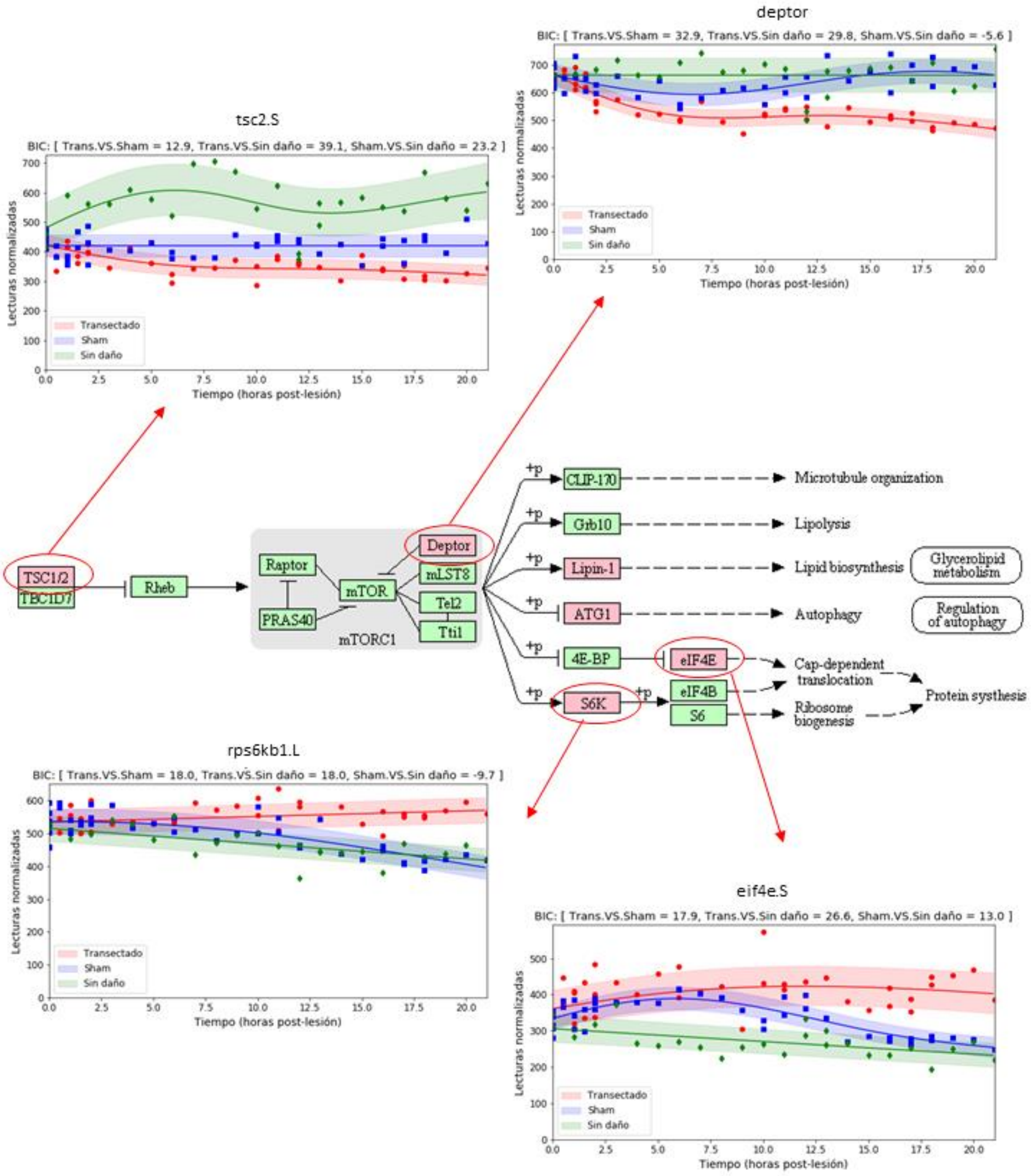

Several components of mTORC1 show early changes in gene expression after SCI. Between these genes we can observe a downregulated after damage in the gene expression profiles of *tsc2* and *deptor*, and an up-regulation in gene expression profiles of *rps6kb1* and *eif4e*, associated to ribosome biogenesis and cap-depending translation, respectively. Both suggest an activation of mTOR pathway with an apparent focus in protein translate regulation.

Supplementary Figure S3. Early inhibition of mTOR signaling pathway using Rapamycin impairs swimming recovery.

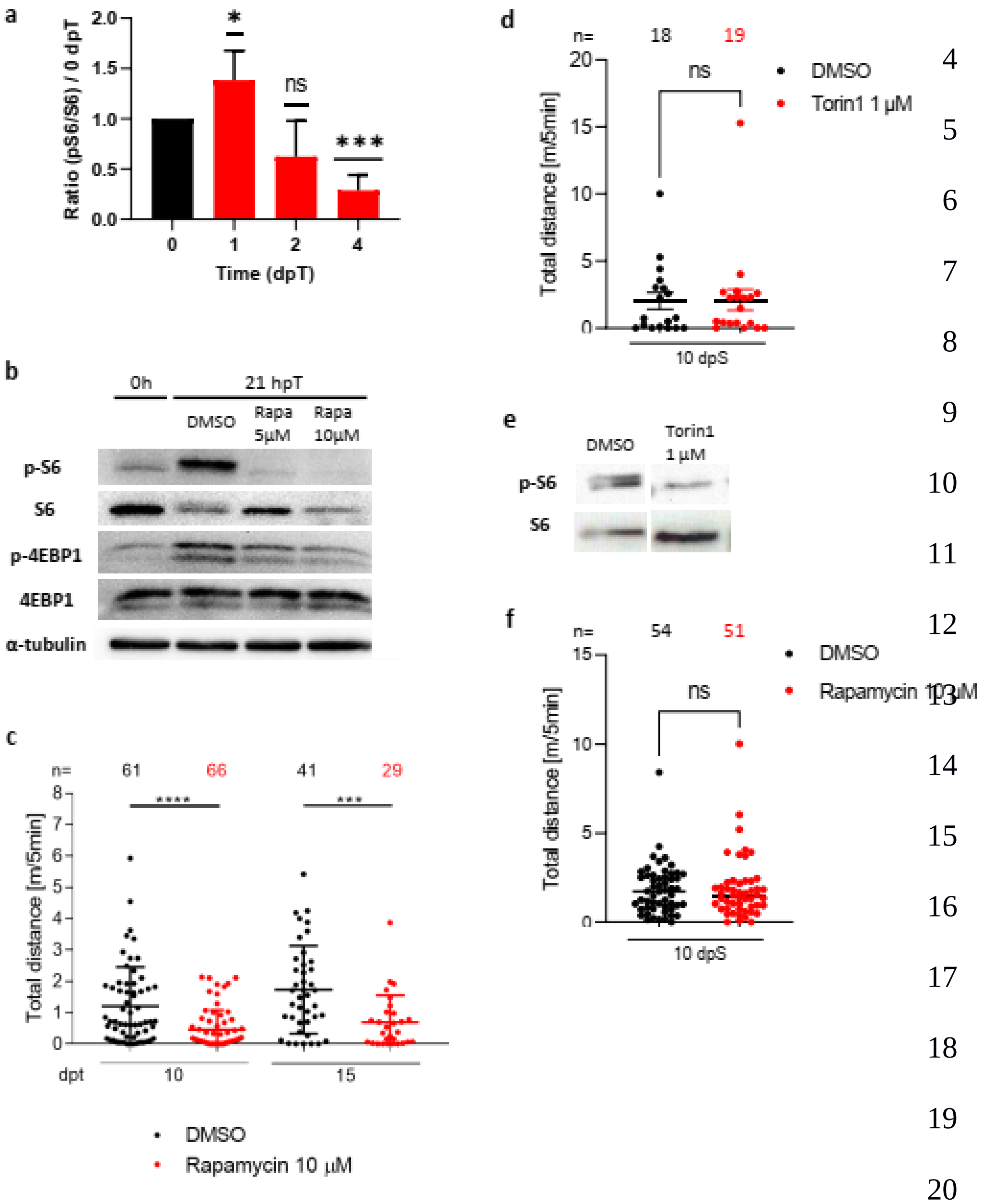

Similar results observed for swimming recovery after Torin1 treatment are reported with Rapamycin treatment. **(a)** Quantification and statistical comparison for immunodetection of ratio pS6/S6 using Western Blot. Data indicate the mean ± SEM, from five independent

experiments. *P <0.05, ***P < 0.001, from a t-test comparing temporal point to control condition at 0 dpT. **(b)** Western Blot for S6, p-S6, 4EBP1 and p-4EBP1 in total protein extracts obtained from spinal cords at 0 hpT and 21 hpT, from *X. laevis* incubated with DMSO (vehicle), Rapamycin 5 µM and 10 µM, for evaluation of mTOR signaling pathway activation. **(c)** Dotplot of swimming distances, of animals treated with vehicle (black dots) and 10 µM Rapamycin (red dots), measured at 10 and 15 dpT. Data indicate the mean ± SEM, from three independent experiments. ***P <0.001, ****P <0.0001, from a Mann Whitney U-test comparing Torin1 treated animals to control, at each time point. **(d)** Dotplot of swimming distances, of animals treated with vehicle (black dots) and Torin1 1 µM (red dots), measured at 10 dpS. Data indicate the mean ± SEM, from one experiment. ns: not significant, from a Mann Whitney U-test comparing Torin1 treated animals to control, at each time point. **(e)** Western Blot for S6 and p-S6 in total protein extracts obtained from spinal cords at 1 dpS, from *X. laevis* incubated with DMSO (vehicle) and Torin1 1 µM, for evaluation of mTOR signaling pathway inhibition in swimming recovery assay. All the blots included in this figure were derived from the same experiment and were processed in parallel. **(f)** Dotplot of swimming distances, of animals treated with vehicle (black dots) and 10 µM Rapamycin (red dots), measured at 10 dpS. Data indicate the mean ± SEM, from two independent experiments. ns: not significant, from a Mann Whitney U-test comparing Rapamycin treated animals to control, at each time point.

Supplementary Figure S4. Early inhibition of mTOR increased the number of TUNEL⁺ cells at 1 dpT.

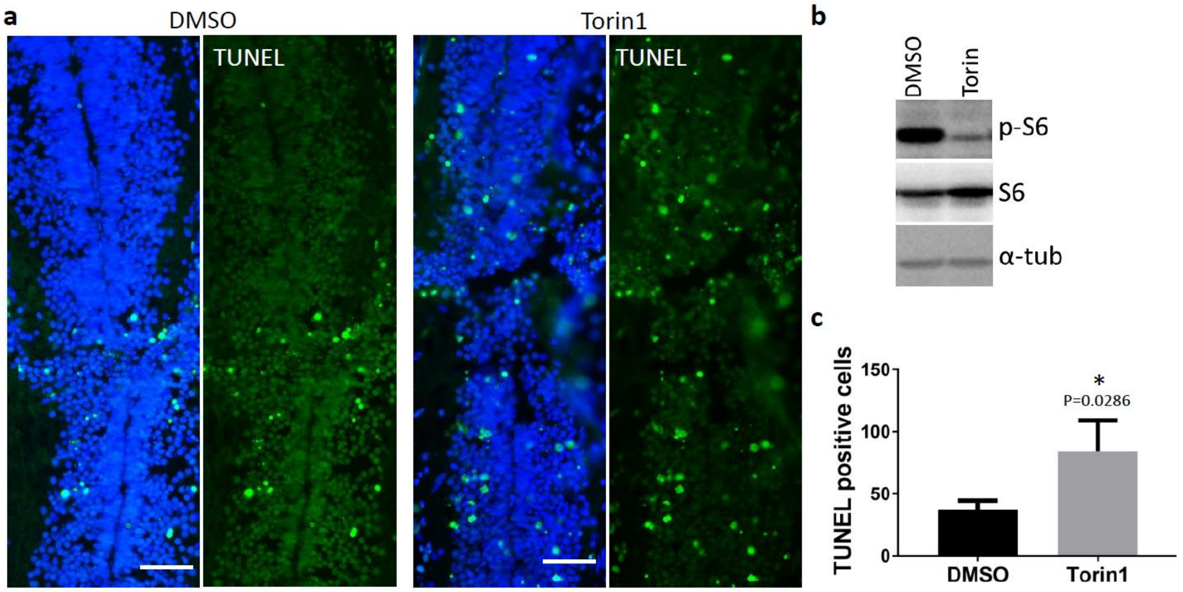

Effect of Torin1 on cell death analyzed by TUNEL. **(a)** Representative images of longitudinal cryosections of spinal cord at 1dpT, 300 µm rostral (upper part) and 300 µm caudal (lower part) to the injury site. TUNEL positive cells (green) and the nuclear staining Hoechst (blue) are shown. Scale bar = 50 µm. **(b)** Western Blot for p-S6, S6 and β-tubulin in total protein extracts obtained from spinal cords at 1 dpT, for evaluation of mTOR signaling pathway inhibition after Torin1 treatment. **(c)** Quantification of TUNEL positive cells in a defined spinal cord area (300 µm rostral and caudal to the injury site). Data indicate the mean ± SEM, from four independent experiments. * $P < 0.05$, from a non-parametric Mann Whitney test comparing Torin1 to vehicle treatments. All the blots included in this figure were derived from the same experiment and were processed in parallel.

Figure S5. Summary of Differential Gene Expression and GO Enrichment analysis for mTOR Inhibition mRNA-seq.

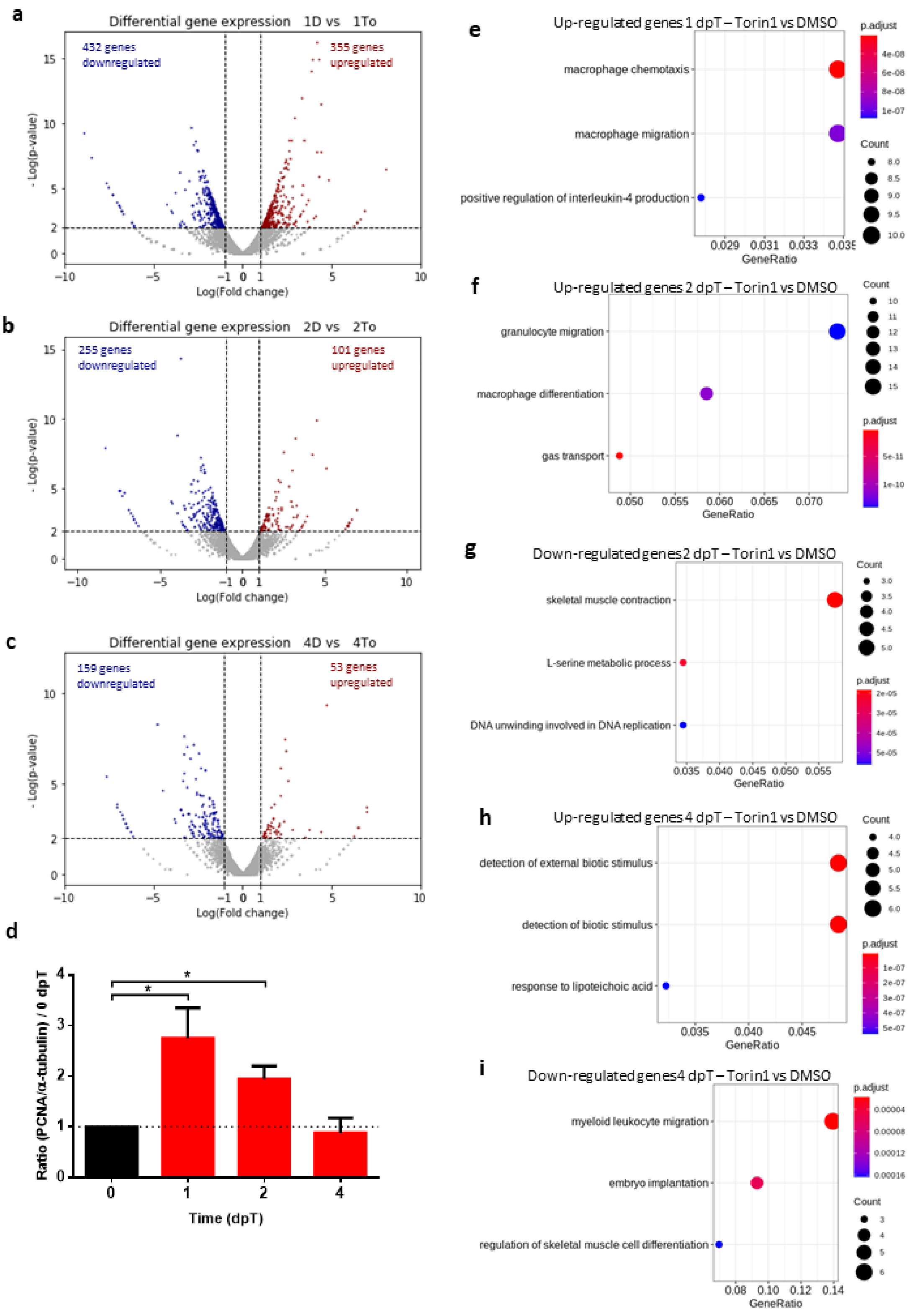

(a-c) Differential gene expression analysis of mTOR Inhibition mRNA-seq at 1 **(a)**, 2 **(b)** and 4 **(c)** dpT are summarized in volcano plots. **(d)** Quantification and statistical comparison for inmmunodetection of PCNA using Western Blot. Data indicate the mean ± SEM, from three independent experiments. *P <0.05, from a t-test comparing temporal point to control condition at 0 dpT. **(e-i)** Three principal biological processes enriched are reported for upregulated genes at 1 **(e)**, 2 **(f)** and 4 **(h)** dpT and for downregulated genes at 2 **(g)** and 4 **(i)** dpT.

Supplementary Figure S6. Batch effect correction improve correlation between transected time-series.

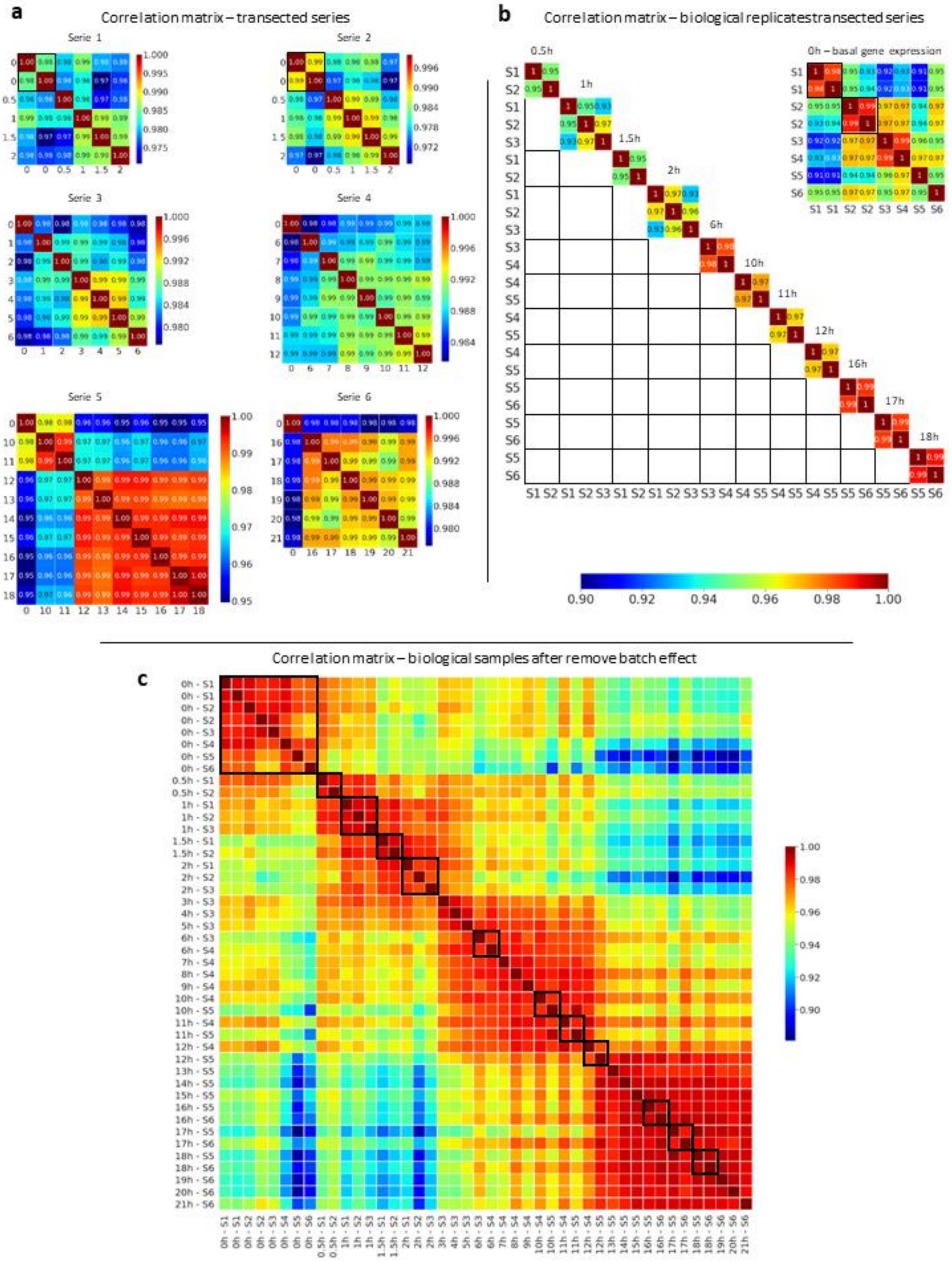

(a) Heatmap show Spearman correlation calculated between temporal points in each time-series before batch effect correction. Correlation coefficient was calculated among all consistently detected genes. Biological replicates at 0 hpT in S1 and S2 are highlighted a black square. Correlation coefficients are indicated in each square and also accord to color bar. **(b)** Heatmap comparing Spearman correlation for samples used as overlapping points between time-series in transected condition before batch effect correction. The inset depicts the correlation for 0 hpT time points included in all time-series. **(c)** Spearman correlation after remove batch effect is illustrated for transected samples. Coefficient correlation was calculated between all genes that showed an effect after transection (BIC > 0). If overlapping point highlighted a black square are compared with B we can to observe an improving in coefficient correlation.

Supplementary Figure S7. Batch effect correction improve correlation between sham time-series.

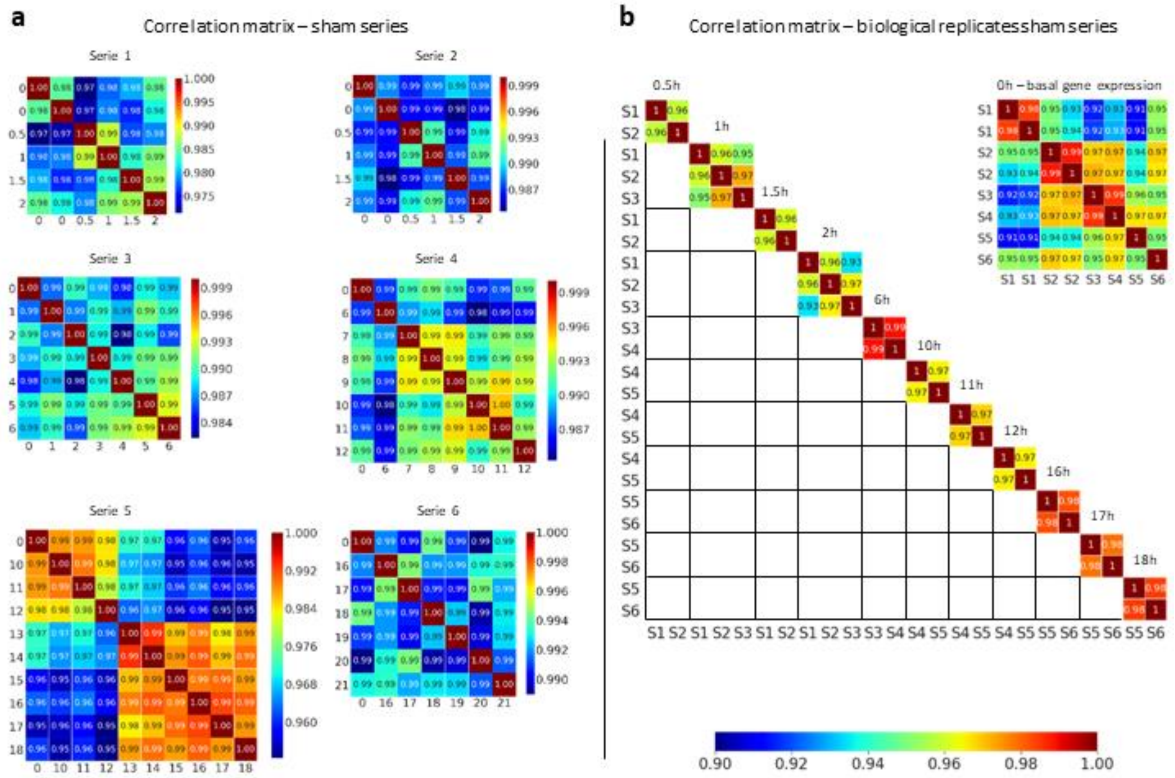

(**a**) Heatmap show Spearman correlation calculated between temporal points in each time-series. Correlation coefficient was calculated among all consistently detected genes. Biological replicates at 0 hpT in S1 and S2 are highlighted a black square. Correlation coefficients are indicated in each square and also accord to color bar. **(b)** Heatmap comparing Spearman correlation for samples used as overlapping points between time-series in sham condition before batch effect correction. The inset depicts the correlation for 0 hpT time points included in all time-series.

Figure S8. Batch effect correction improve correlation between uninjured time-series.

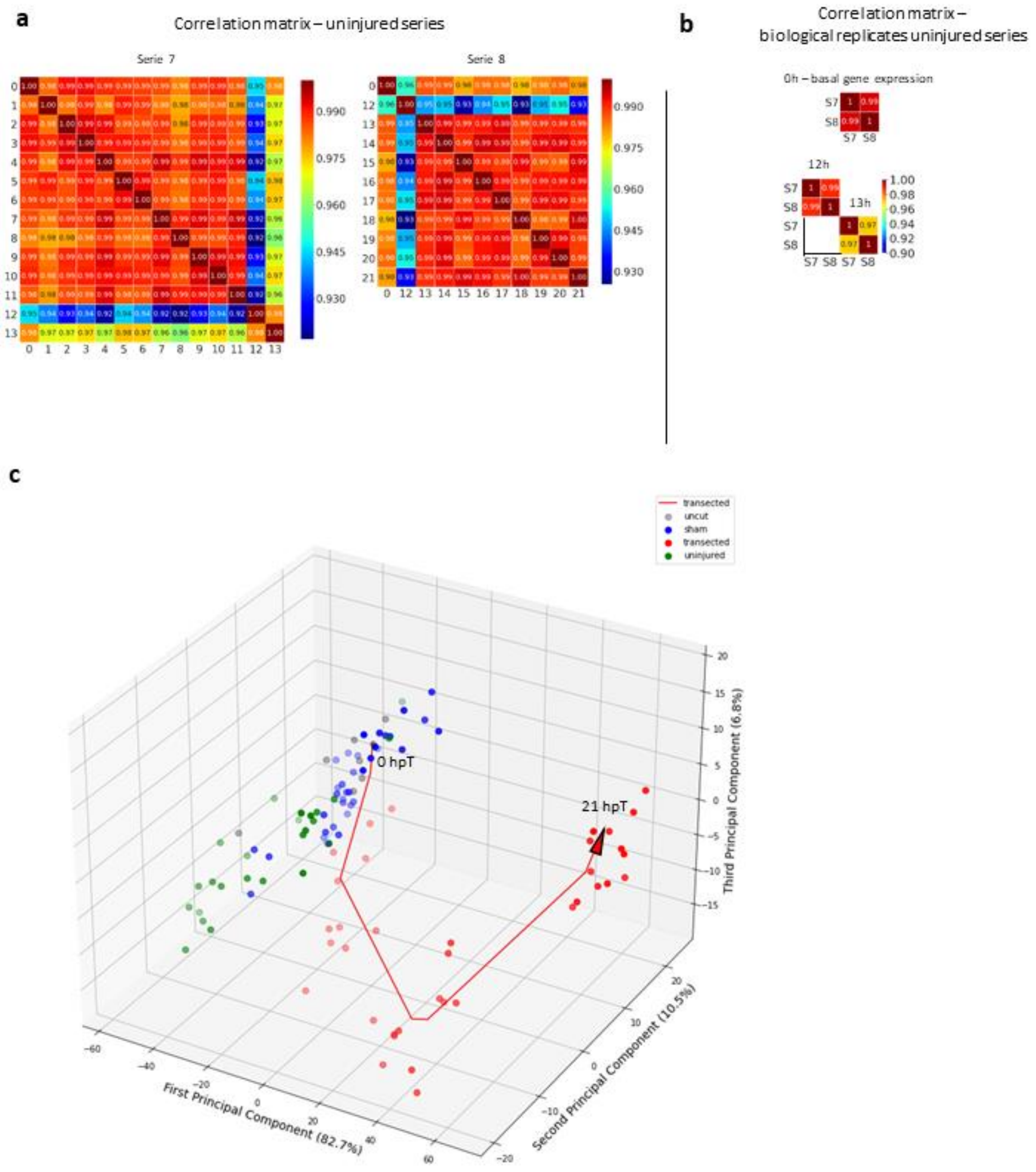

(**a**) Heatmap show Spearman correlation calculated between temporal points in each time-series before batch effect correction. Correlation coefficient was calculated among all consistently detected genes. Biological replicates at 0 hpT in S1 and S2 are highlighted a black square. Correlation coefficients are indicated in each square and also accord to color bar. **(b)** Heatmap comparing Spearman correlation for samples used as overlapping points between time-series in uninjured condition before batch effect correction. The inset depicts the correlation for 0 hpT time points included in all time-series. **(c)** 3D PCA Plot comparing samples of transected (red), sham (blue) and uninjured (green) conditions, including uncut samples used as 0 hpT for transected and sham conditions (grey). PCA was performed over the data of all consistently detected genes. Red line represents a B-Spline interpolation for transected data according to sampling time. Intensity color of dots for transected, sham and uninjured conditions is increasing with sampling time.

Supplementary Figure S9. Individual gene expression profiles are affected by batch effect between time-series.

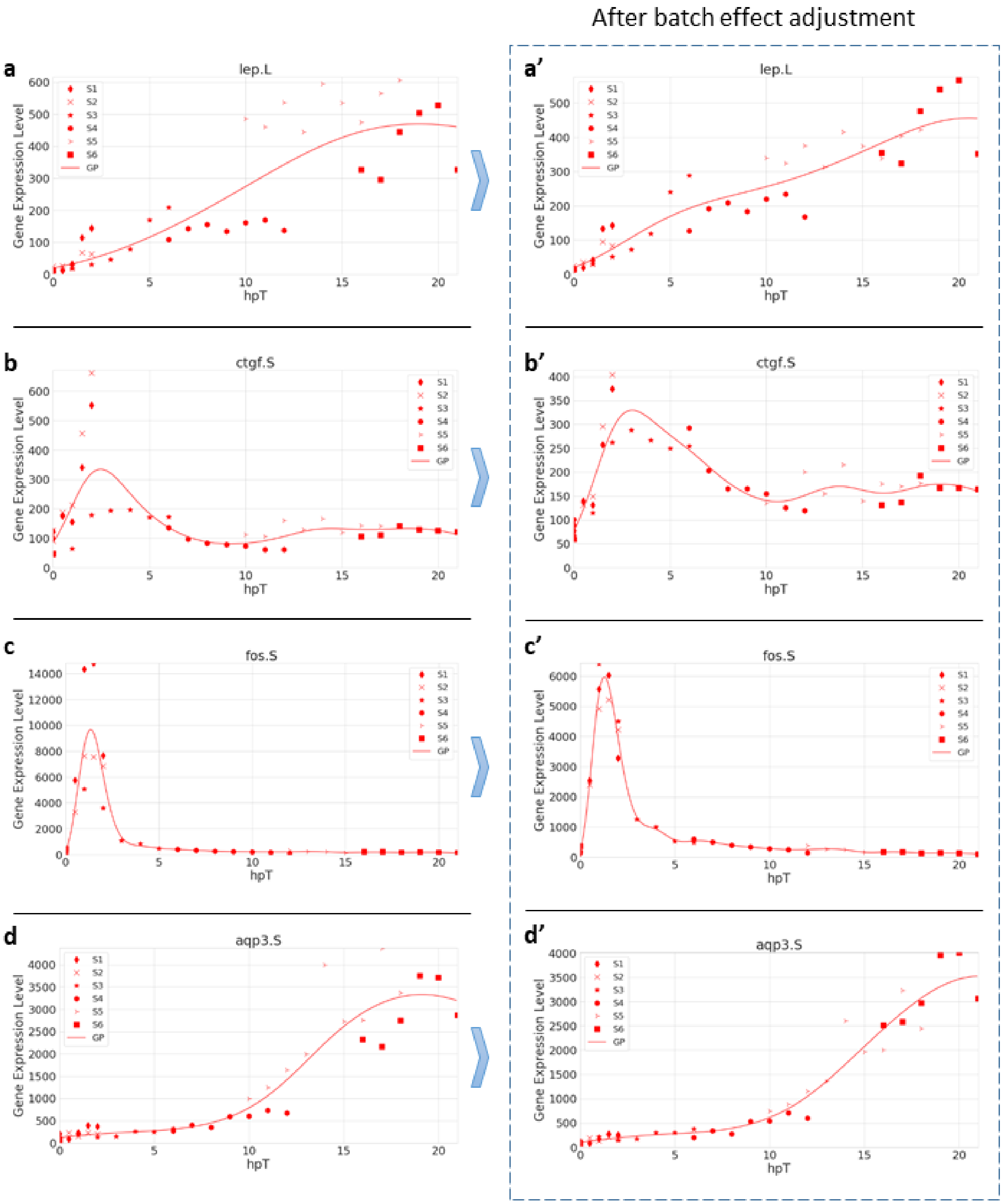

A poor overlapping is observed for some genes when gene expression profiles are constructed after merging all time-series. Graph in panels **a,b,c** and **d** represent four examples of genes affected by batch effect and **a', b',c'** and **d'** depicts the corresponding profiles after batch effect adjustment using the Combat function of the SVA package. Not all genes are affected equally by batch effects which could be observed when **d** is compared with **a**, **b** and **c**.

Supplementary Table S1 - Quality Score and Aligment Rate per Library

Serie	Sample		# Reads	Size	Mean Q-Score	% Aligned Reads
S1,S1'	-	0h (R1)	19,864,452	50	37.8	70.4%
S1,S1'	-	0h (R2)	17,001,050	50	37.8	70.1%
S1'	SHAM	0.5h	16,066,080	50	37.8	69.6%
S1	TRANS	0.5h	18,222,957	50	37.7	68.2%
S1'	SHAM	1h	18,310,893	50	37.7	69.3%
S1	TRANS	1h	17,651,652	50	37.7	70.3%
S1'	SHAM	1.5h	14,699,064	50	37.8	68.3%
S1	TRANS	1.5h	16,044,732	50	38.0	72.0%
S1'	SHAM	2h	16,197,643	50	37.8	69.9%
S1	TRANS	2h	16,573,749	50	37.8	72.0%
S2,S2'	-	0h (R1)	19,392,167	50	37.8	70.0%
S2,S2'	-	0h (R2)	18,882,037	50	37.9	70.3%
S2'	SHAM	0.5h	15,311,616	50	37.8	72.1%
S2	TRANS	0.5h	16,843,042	50	37.8	73.5%
S2'	SHAM	1h	19,569,871	50	37.7	71.9%
S2	TRANS	1h	17,802,141	50	37.7	70.6%
S2'	SHAM	1.5h	20,120,753	50	37.8	70.0%
S2	TRANS	1.5h	18,352,733	50	37.8	72.7%
S2'	SHAM	2h	20,769,534	50	37.8	73.3%
S2	TRANS	2h	15,071,279	50	37.7	71.4%
S3,S3'	-	0h	19,837,973	51	37.6	70.3%
S3'	SHAM	1h	27,537,214	51	36.5	70.8%
S3	TRANS	1h	16,193,233	51	37.6	69.1%
S3'	SHAM	2h	18,092,933	51	37.6	70.6%
S3	TRANS	2h	21,976,557	51	37.8	73.8%
S3'	SHAM	3h	14,747,972	51	37.9	71.8%
S3	TRANS	3h	36,386,417	51	36.6	70.0%
S3'	SHAM	4h	30,142,561	51	37.7	70.4%
S3	TRANS	4h	23,580,012	51	37.7	71.5%
S3'	SHAM	5h	51,862,469	51	36.5	70.9%
S3	TRANS	5h	42,464,794	51	37.7	71.5%
S3'	SHAM	6h	26,593,936	51	38.0	71.6%
S3	TRANS	6h	30,639,623	51	37.7	72.1%
S4,S4'	-	0h	34,293,485	51	37.7	71.2%
S4'	SHAM	6h	25,559,236	51	38.0	68.6%
S4	TRANS	6h	21,267,923	51	37.8	71.3%
S4'	SHAM	7h	23,190,895	51	37.7	72.4%
S4	TRANS	7h	19,264,300	51	37.8	71.3%
S4'	SHAM	8h	23,717,921	51	37.8	71.5%
S4	TRANS	8h	23,346,692	51	38.0	72.5%
S4'	SHAM	9h	50,305,092	51	36.5	71.8%

S4	TRANS	9h	21,458,987	51	37.6	71.6%
S4'	SHAM	10h	28,627,224	51	37.7	71.3%
S4	TRANS	10h	31,972,279	51	37.8	71.1%
S4'	SHAM	11h	49,683,113	51	36.6	72.5%
S4	TRANS	11h	19,180,383	51	37.8	72.8%
S4'	SHAM	12h	24,306,102	51	37.8	70.8%
S4	TRANS	12h	17,132,651	51	37.8	72.8%
S5,S5'	-	0h	26,334,967	51	37.4	69.5%
S5'	SHAM	10h	26,348,795	51	37.3	65.8%
S5	TRANS	10h	25,689,539	51	37.4	69.6%
S5'	SHAM	11h	19,493,335	51	37.5	67.0%
S5	TRANS	11h	31,742,604	51	37.3	68.0%
S5'	SHAM	12h	25,222,408	51	37.2	67.9%
S5	TRANS	12h	35,741,701	51	37.3	70.4%
S5'	SHAM	13h	28,932,056	51	37.4	71.1%
S5	TRANS	13h	32,919,536	51	37.4	73.9%
S5'	SHAM	14h	27,499,234	51	37.3	71.8%
S5	TRANS	14h	30,614,502	51	37.4	70.2%
S5'	SHAM	15h	38,224,480	51	37.2	73.0%
S5	TRANS	15h	37,218,330	51	37.1	70.2%
S5'	SHAM	16h	26,539,788	51	37.1	70.8%
S5	TRANS	16h	21,079,571	51	37.4	70.4%
S5'	SHAM	17h	30,194,620	51	37.1	67.2%
S5	TRANS	17h	39,159,329	51	37.0	70.9%
S5'	SHAM	18h	27,922,262	51	37.1	68.0%
S5	TRANS	18h	108,006,595	51	36.7	71.6%
S6,S6'	-	0h	29,889,602	51	37.3	69.6%
S6'	SHAM	16h	43,043,267	51	35.0	64.3%
S6	TRANS	16h	52,225,695	51	35.1	64.7%
S6'	SHAM	17h	41,853,129	51	34.7	61.4%
S6	TRANS	17h	29,163,192	51	37.4	65.2%
S6'	SHAM	18h	32,193,154	51	37.1	66.7%
S6	TRANS	18h	30,364,293	51	37.2	68.5%
S6'	SHAM	19h	23,766,288	51	37.4	67.8%
S6	TRANS	19h	27,048,993	51	37.3	67.9%
S6'	SHAM	20h	27,592,826	51	37.1	63.2%
S6	TRANS	20h	23,443,149	51	37.3	67.9%
S6'	SHAM	21h	25,636,766	51	37.4	66.5%
S6	TRANS	21h	31,009,574	51	37.5	67.3%
S7	UNINJ	0h	32,204,037	51	36.7	66.2%
S7	UNINJ	1h	30,573,605	51	36.9	64.6%
S7	UNINJ	2h	30,944,504	51	37.3	69.1%
S7	UNINJ	3h	30,922,773	51	37.4	69.6%

S7	UNINJ	4h	34,844,190	51	37.1	66.9%
S7	UNINJ	5h	27,674,143	51	37.0	67.2%
S7	UNINJ	6h	27,516,635	51	36.7	65.9%
S7	UNINJ	7h	31,055,511	51	37.3	65.2%
S7	UNINJ	8h	21,801,494	51	37.1	64.8%
S7	UNINJ	9h	21,228,743	51	36.7	64.6%
S7	UNINJ	10h	28,650,110	51	37.1	69.0%
S7	UNINJ	11h	36,070,048	51	36.8	64.3%
S7	UNINJ	12h	23,071,050	51	36.5	66.6%
S7	UNINJ	13h	28,002,613	51	36.9	66.1%
S8	UNINJ	0h	26,931,801	51	37.0	66.9%
S8	UNINJ	12h	26,482,971	51	36.7	69.5%
S8	UNINJ	13h	33,717,355	51	37.0	66.2%
S8	UNINJ	14h	31,598,901	51	37.0	68.1%
S8	UNINJ	15h	28,371,384	51	37.0	65.4%
S8	UNINJ	16h	33,321,746	51	37.0	68.4%
S8	UNINJ	17h	28,993,749	51	37.3	70.3%
S8	UNINJ	18h	34,612,737	51	37.0	64.6%
S8	UNINJ	19h	32,463,905	51	36.9	66.9%
S8	UNINJ	20h	28,548,009	51	37.2	68.4%
S8	UNINJ	21h	45,561,395	51	37.0	65.6%
mTOR Inhibition	TRANS	24h TORIN1	35,596,842	150	37.9	59.5%
mTOR Inhibition	TRANS	24h DMSO	48,243,669	150	37.9	59.0%
mTOR Inhibition	TRANS	48h TORIN1	48,908,365	150	37.9	59.2%
mTOR Inhibition	TRANS	48h DMSO	50,854,436	150	37.9	60.5%
mTOR Inhibition	TRANS	96h TORIN1	53,050,294	150	37.9	58.0%
mTOR Inhibition	TRANS	96h DMSO	54,484,119	150	37.8	59.7%

Supplementary Table S2 - Differential gene expression and Clustering analysis of time-series mRNA-seq

Module	Gene ID	Gene Symbol	Blastp Human	Transected VS Sham	Transected VS Uninjured	Sham VS Uninjured	MM	p.MM
E1	mi|Xelaev18043661m.g|	socs3.L	SOCS3	68.44	47.52	-15.26	0.84	6.07E-13
E1	mi|Xelaev18034372m.g|	ch25h.L	CH25H	64.36	37.66	-3.95	0.84	1.63E-12
E1	mi|Xelaev18034007m.g|	myc.S	MYC	58.10	53.01	10.02	0.76	1.72E-09
E1	mi|Xelaev18025150m.g|	jun.S	JUN	53.19	46.62	-6.58	0.90	5.13E-17
E1	mi|Xelaev18023019m.g|	jun.L	JUN	52.51	28.30	-5.25	0.85	2.17E-13
E1	mi|Xelaev18039459m.g|	fos.L	FOS	45.79	34.14	-0.22	0.92	1.77E-18
E1	mi|Xelaev18046376m.g|	socs3.S	SOCS3	44.06	16.91	-10.85	0.92	1.71E-18
E1	mi|Xelaev18047800m.g|	rasd1.S	RASD1	43.37	25.53	-15.97	0.95	9.09E-23
E1	mi|Xelaev18004909m.g|	egr4.L	EGR4	43.36	38.43	6.28	0.95	2.04E-22
E1	mi|Xelaev18041369m.g|	fos.S	FOS	38.15	24.38	-16.95	0.89	9.04E-16
E1	mi|Xelaev18043066m.g|	coq10b.L	COQ10B	36.52	31.72	-11.23	0.90	5.89E-17
E1	mi|Xelaev18019082m.g|	junb.L	JUNB	35.20	22.61	-10.66	0.96	1.46E-25
E1	mi|Xelaev18019097m.g|	ier2.L	N/B	33.79	36.50	-4.06	0.94	9.80E-21
E1	mi|Xelaev18014874m.g|	btg2.S	BTG2	30.35	12.91	-16.70	0.85	4.39E-13
E1	mi|Xelaev18027111m.g|	gja1.L	GJA1	27.98	13.08	-8.19	0.71	6.81E-08
E1	mi|Xelaev18039067m.g|	loc100490871-like.L	FOS	27.64	24.96	-13.14	0.90	1.48E-16
E1	mi|Xelaev18019817m.g|	timp3.S	TIMP3	25.19	21.42	-15.74	0.81	2.45E-11
E1	mi|Xelaev18000800m.g|	junb.S	JUNB	25.01	15.11	-9.89	0.96	1.46E-24
E1	mi|Xelaev18042368m.g|	loc100490871-like.S	FOS	24.09	29.24	-8.08	0.91	8.78E-18
E1	mi|Xelaev18028547m.g|	loc101733171.S	HIST1H2BJ	23.22	18.00	-11.27	0.87	1.11E-14
E1	mi|Xelaev18028696m.g|	atf3.S	ATF3	22.09	26.64	-12.80	0.94	1.06E-20
E1	mi|Xelaev18032361m.g|	myc.L	MYC	21.89	29.95	3.72	0.76	2.03E-09
E1	mi|Xelaev18039068m.g|	Xelaev18039068m.g	N/B	20.48	14.14	-13.00	0.90	7.95E-17
E1	mi|Xelaev18039213m.g|	ctgfl.L	WISP1	19.35	24.48	-2.90	0.75	4.72E-

								09
E1	mi|Xelaev18027531m.g|	hes1.L	HES1	18.85	13.28	-11.04	0.75	5.68E-09
E1	mi|Xelaev18012500m.g|	fhl3.L	FHL3	18.83	25.92	-4.12	0.71	5.47E-08
E1	mi|Xelaev18023910m.g|	bhlhe40.L	BHLHE40	18.72	29.58	-8.49	0.94	1.27E-21
E1	mi|Xelaev18043375m.g|	itgb3.L	N/B	17.52	16.23	-12.87	0.78	4.83E-10
E1	mi|Xelaev18005276m.g|	rasl11b.L	RASL11B	17.47	12.01	-0.93	0.86	4.98E-14
E1	mi|Xelaev18023622m.g|	ptgs2.L	PTGS2	17.41	13.78	-6.71	0.86	6.02E-14
E1	mi|Xelaev18033922m.g|	klf10.S	KLF10	17.19	31.67	-5.35	0.79	1.19E-10
E1	mi|Xelaev18032432m.g|	arc.L	ARC	17.03	15.28	-21.60	0.97	6.03E-28
E1	mi|Xelaev18032771m.g|	gjc2.S	GJC1	16.89	42.07	14.79	0.75	4.38E-09
E1	mi|Xelaev18018776m.g|	tnfrsf10b.L	TNFRSF10B	16.29	31.36	1.17	0.91	4.95E-18
E1	mi|Xelaev18027287m.g|	srsf12.L	SRSF12	16.15	21.17	-10.66	0.63	5.32E-06
E1	mi|Xelaev18030118m.g|	rhob.S	RHOB	15.57	40.54	8.35	0.81	3.45E-11
E1	mi|Xelaev18005131m.g|	hs3st1.L	HS3ST1	15.49	16.16	-2.81	0.87	2.70E-14
E1	mi|Xelaev18041423m.g|	loc100497513-like.S	N/B	15.42	13.79	-6.91	0.90	4.78E-17
E1	mi|Xelaev18017353m.g|	egr1.L	EGR1	15.22	11.10	-20.02	0.90	2.09E-16
E1	mi|Xelaev18036625m.g|	dusp5.S	DUSP5	15.20	14.70	-23.09	0.83	3.25E-12
E1	mi|Xelaev18008744m.g|	egr4.S	EGR4	14.92	21.00	0.64	0.93	6.18E-20
E1	mi|Xelaev18005051m.g|	tnip2.L	TNIP2	13.99	23.94	-8.85	0.94	5.65E-21
E1	mi|Xelaev18021648m.g|	Xelaev18021648m.g	PHLDA2	13.34	15.40	-8.36	0.70	9.88E-08
E1	mi|Xelaev18024546m.g|	adm.S	ADM	12.56	28.36	6.36	0.83	1.86E-12
E1	mi|Xelaev18017457m.g|	dusp6.L	DUSP6	11.71	32.59	4.79	0.92	6.53E-19
E1	mi|Xelaev18011436m.g|	ets2.L	ETS2	10.85	22.97	-3.92	0.93	2.89E-20
E1	mi|Xelaev18005395m.g|	vegfc.L	VEGFC	10.79	16.54	-6.84	0.66	1.19E-06
E1	mi|Xelaev18038173m.g|	loc101731931.L	CYR61	10.55	18.97	-5.40	0.78	5.44E-10
E1	mi|Xelaev18039957m.g|	nfkbia.L	NFKBIA	10.41	12.05	-10.14	0.86	1.26E-13

E2	mi|Xelaev18022274m.g|	fosl1.L	FOSL1	71.33	58.45	-9.21	0.97	1.02E-28
E2	mi|Xelaev18001506m.g|	tnfrsf12a.L	TNFRSF12A	67.76	45.69	-10.87	0.89	1.05E-15
E2	mi|Xelaev18024674m.g|	Xelaev18024674m.g	PHLDA2	62.93	55.68	-7.65	0.90	1.11E-16
E2	mi|Xelaev18026209m.g|	atf3.L	ATF3	61.48	61.45	-8.89	0.95	9.28E-23
E2	mi|Xelaev18009501m.g|	Xelaev18009501m.g	N/B	60.20	34.67	-8.68	0.88	5.62E-15
E2	mi|Xelaev18030082m.g|	odc1.S	ODC1	53.09	60.02	19.98	0.87	2.08E-14
E2	mi|Xelaev18040374m.g|	ecm1.L	ECM1	52.43	37.56	-11.52	0.85	1.54E-13
E2	mi|Xelaev18011954m.g|	g0s2.L	G0S2	52.13	59.19	19.21	0.78	3.71E-10
E2	mi|Xelaev18007407m.g|	lif.L	LIF	51.72	36.18	-11.83	0.79	2.28E-10
E2	mi|Xelaev18023601m.g|	selp.L	SELP	51.39	31.73	-8.19	0.84	1.68E-12
E2	mi|Xelaev18039961m.g|	actn1.L	ACTN1	49.51	21.27	-4.36	0.75	4.23E-09
E2	mi|Xelaev18000853m.g|	serpine1.S	N/B	48.68	52.31	3.17	0.95	3.49E-22
E2	mi|Xelaev18003784m.g|	ctgf.S	CTGF	47.70	55.71	17.86	0.91	5.94E-18
E2	mi|Xelaev18024193m.g|	fosl1.S	FOSL1	45.88	36.12	-14.20	0.93	1.97E-19
E2	mi|Xelaev18002248m.g|	Xelaev18002248m.g	N/B	45.74	32.54	-8.41	0.77	8.15E-10
E2	mi|Xelaev18036267m.g|	btg5.L	BTG3	45.25	61.90	5.05	0.98	5.63E-30
E2	mi|Xelaev18040146m.g|	thbs1.L	THBS1	44.59	42.58	-10.89	0.95	4.25E-23
E2	mi|Xelaev18009500m.g|	cxcl8.S	N/B	42.26	28.13	-9.76	0.91	1.38E-17
E2	mi|Xelaev18004505m.g|	pdlim1.S	PDLIM1	41.19	46.90	-8.02	0.72	2.69E-08
E2	mi|Xelaev18026396m.g|	Xelaev18026396m.g	HIST1H2BJ	40.52	36.26	-6.66	0.81	3.62E-11
E2	mi|Xelaev18005127m.g|	wdr1.L	WDR1	39.31	35.52	-0.07	0.73	2.51E-08
E2	mi|Xelaev18019168m.g|	serpine1.L	N/B	39.29	27.86	-15.74	0.92	2.57E-18
E2	mi|Xelaev18019226m.g|	loc100496442.L	N/B	38.68	33.60	-7.63	0.78	4.52E-10
E2	mi|Xelaev18042870m.g|	tmem79.S	TMEM79	37.51	10.15	-3.99	0.77	9.51E-10
E2	mi|Xelaev18005828m.g|	Xelaev18005828m.g	N/B	36.13	39.57	-7.43	0.92	4.90E-19
E2	mi|Xelaev18037813m.g|	loc733735.S	CDC42EP4	35.73	23.86	-11.74	0.77	9.01E-10

E2	mi|Xelaev18006292m.g|	angptl4.L	ANGPTL4	35.68	22.87	-12.15	0.86	6.13E-14
E2	mi|Xelaev18028546m.g|	srf.S	SRF	35.23	25.82	-4.56	0.75	3.32E-09
E2	mi|Xelaev18020909m.g|	pfkfb3.S	PFKFB3	34.30	59.51	30.19	0.90	3.63E-17
E2	mi|Xelaev18025526m.g|	Xelaev18025526m.g	N/B	34.16	18.31	-10.24	0.73	1.53E-08
E2	mi|Xelaev18009822m.g|	angptl4.S	ANGPTL4	33.01	34.90	-6.76	0.74	1.07E-08
E2	mi|Xelaev18016099m.g|	lima1.S	LIMA1	31.62	49.31	0.98	0.95	3.62E-22
E2	mi|Xelaev18012016m.g|	cdkn1a.L	CDKN1A	31.36	29.16	-15.13	0.84	8.68E-13
E2	mi|Xelaev18021488m.g|	irf1.S	IRF1	29.15	12.90	-9.49	0.82	1.26E-11
E2	mi|Xelaev18036485m.g|	Xetrov90019643m.L	N/B	27.32	30.00	-8.50	0.83	2.68E-12
E2	mi|Xelaev18041148m.g|	btbd6.S	BTBD6	27.20	13.72	-11.46	0.78	5.10E-10
E2	mi|Xelaev18046848m.g|	slc16a6.S	SLC16A6	27.15	56.68	27.68	0.93	6.83E-20
E2	mi|Xelaev18014950m.g|	nuak2.S	NUAK2	26.57	37.45	12.80	0.79	2.08E-10
E2	mi|Xelaev18046073m.g|	map3k14.S	MAP3K14	26.38	10.69	-15.84	0.81	3.90E-11
E2	mi|Xelaev18013815m.g|	birc2.L	BIRC2	25.18	33.60	-6.72	0.66	1.25E-06
E2	mi|Xelaev18043136m.g|	Xelaev18043136m.g	ARF3	24.99	13.00	-9.47	0.61	1.09E-05
E2	mi|Xelaev18026400m.g|	Xelaev18026400m.g	HIST1H1D	24.12	32.09	-0.32	0.78	4.87E-10
E2	mi|Xelaev18010901m.g|	Xelaev18010901m.g	F2RL1	24.04	41.72	0.68	0.82	7.84E-12
E2	mi|Xelaev18012102m.g|	fam46b.L	TENT5A	23.07	18.08	-2.46	0.80	4.82E-11
E2	mi|Xelaev18047117m.g|	upp2.S	UPP2	22.22	30.82	1.20	0.84	5.61E-13
E2	mi|Xelaev18033923m.g|	azin1.S	AZIN1	21.86	35.69	-1.65	0.81	2.61E-11
E2	mi|Xelaev18036272m.g|	Xelaev18036272m.g	CMKLR1	20.68	23.21	-4.38	0.69	1.94E-07
E2	mi|Xelaev18014461m.g|	nr1i2.S	NR1I2	20.51	18.60	-11.89	0.80	6.70E-11
E2	mi|Xelaev18047416m.g|	nab1.S	NAB1	20.33	24.32	-12.71	0.79	1.34E-10
E2	mi|Xelaev18043154m.g|	csf3.L	N/B	18.61	31.65	0.05	0.74	1.22E-08
E2	mi|Xelaev18024085m.g|	ppp4r2.L	PPP4R2	18.10	31.38	8.85	0.75	4.06E-09
E2	mi|Xelaev18010243m.g|	rem2.S	REM2	17.43	30.94	5.36	0.79	2.81E-10

E2	mi|Xelaev18013440m.g|	ddit3.L	DDIT3	17.42	22.25	-7.03	0.79	2.29E-10
E2	mi|Xelaev18027688m.g|	rap2b.L	RAP2B	17.35	13.87	-5.96	0.66	1.05E-06
E2	mi|Xelaev18024237m.g|	lrrn4cl.S	LRRN4CL	17.02	12.80	-8.51	0.74	1.12E-08
E2	mi|Xelaev18033486m.g|	stmnd1.S	STMND1	16.76	20.98	2.22	0.64	3.00E-06
E2	mi|Xelaev18015254m.g|	luzp1.S	LUZP1	16.49	21.33	-2.38	0.72	2.93E-08
E2	mi|Xelaev18008815m.g|	smox.S	SMOX	16.36	17.82	-12.97	0.82	1.49E-11
E2	mi|Xelaev18004051m.g|	azin2.S	ODC1	16.02	30.64	-1.32	0.86	4.97E-14
E2	mi|Xelaev18012053m.g|	ptafr.L	PTAFR	16.00	10.95	-13.79	0.76	2.19E-09
E2	mi|Xelaev18035744m.g|	errfi1.L	ERRFI1	15.72	14.31	-12.02	0.87	1.89E-14
E2	mi|Xelaev18010749m.g|	znf474.S	ZNF474	15.20	20.22	-6.67	0.69	1.66E-07
E2	mi|Xelaev18007404m.g|	tbc1d10a.L	TBC1D10A	14.60	16.63	-6.82	0.70	1.13E-07
E2	mi|Xelaev18010195m.g|	gadd45g.S	GADD45G	14.28	15.34	-0.96	0.70	1.35E-07
E2	mi|Xelaev18034426m.g|	Xelaev18034426m.g	LTBR	14.06	11.12	-12.99	0.84	9.15E-13
E2	mi|Xelaev18029955m.g|	slc16a14.S	SLC16A14	13.56	30.71	2.54	0.74	1.03E-08
E2	mi|Xelaev18047474m.g|	abat.S	ABAT	13.34	33.71	4.13	0.68	3.03E-07
E2	mi|Xelaev18038046m.g|	Xelaev18038046m.g	N/B	12.90	20.64	-8.16	0.83	1.86E-12
E2	mi|Xelaev18028697m.g|	ppp2r5a.S	PPP2R5A	12.78	27.01	-0.15	0.69	2.82E-07
E2	mi|Xelaev18039489m.g|	sptlc2.L	SPTLC2	12.76	24.39	5.60	0.77	8.28E-10
E2	mi|Xelaev18014228m.g|	col8a1.S	COL8A1	12.46	28.21	-0.89	0.66	1.11E-06
E2	mi|Xelaev18008178m.g|	Xelaev18008178m.g	F2RL1	12.36	20.70	-8.05	0.89	7.61E-16
E2	mi|Xelaev18016815m.g|	fgf23.1.L	FGF23	12.24	31.91	3.95	0.71	6.52E-08
E2	mi|Xelaev18045117m.g|	abat.L	ABAT	12.12	12.79	-7.15	0.67	6.62E-07
E2	mi|Xelaev18023532m.g|	slc25a38.L	SLC25A38	11.91	12.25	2.00	0.64	2.47E-06
E2	mi|Xelaev18018522m.g|	loc100485269.L	HK2	10.92	31.92	7.10	0.76	1.60E-09
E2	mi|Xelaev18014572m.g|	Xelaev18014572m.g	N/B	10.87	39.32	7.82	0.90	1.02E-16
E2	mi|Xelaev18013467m.g|	cyp27b1.L	CYP27B1	10.51	30.25	-1.46	0.79	1.40E-10

E2	mi|Xelaev18014759m.g|	mid1ip1.S	MID1IP1	10.36	44.37	25.83	0.69	2.74E-07
E2	mi|Xelaev18035648m.g|	ccnl2.L	CCNL2	10.31	29.14	-1.29	0.69	2.10E-07
E2	mi|Xelaev18044244m.g|	slc16a6.L	SLC16A6	10.29	44.35	13.19	0.86	1.40E-13
E2	mi|Xelaev18037971m.g|	epha2.S	EPHA2	10.14	15.81	-9.33	0.77	1.14E-09
I1	mi|Xelaev18038146m.g|	timp-1.L	TIMP1	116.79	80.17	-8.10	0.99	8.93E-36
I1	mi|Xelaev18017925m.g|	lep.L	LEP	113.83	96.57	-1.98	0.95	2.03E-22
I1	mi|Xelaev18044787m.g|	frzb.L	FRZB	104.06	58.28	2.36	0.95	3.24E-22
I1	mi|Xelaev18044618m.g|	itgb6.L	ITGB6	102.42	81.22	1.12	0.92	3.44E-18
I1	mi|Xelaev18030023m.g|	ccl34.S	N/B	99.22	73.62	-9.72	0.96	6.69E-24
I1	mi|Xelaev18046858m.g|	Xelaev18046858m.g	CACNG5	99.18	48.51	13.81	0.91	2.63E-17
I1	mi|Xelaev18038172m.g|	glipr2.L	GLIPR2	95.26	45.11	10.00	0.97	6.39E-29
I1	mi|Xelaev18019016m.g|	s1pr5.L	S1PR1	95.26	64.71	-7.71	0.97	2.09E-27
I1	mi|Xelaev18011464m.g|	runx1.L	RUNX1	94.89	56.24	5.49	0.97	1.19E-27
I1	mi|Xelaev18006245m.g|	cnn1.L	CNN2	92.27	87.20	12.06	0.94	7.58E-21
I1	mi|Xelaev18020863m.g|	lep.S	LEP	89.88	55.08	-8.68	0.91	1.20E-17
I1	mi|Xelaev18042523m.g|	mgc69520.S	GPR4	89.57	66.79	8.72	0.96	1.42E-25
I1	mi|Xelaev18009775m.g|	cnn1.S	CNN2	87.84	83.20	18.72	0.88	5.28E-15
I1	mi|Xelaev18013388m.g|	mmp19.L	MMP19	85.88	42.68	1.26	0.97	1.24E-26
I1	mi|Xelaev18005895m.g|	sgk223.L	PRAG1	84.63	59.02	-11.68	0.95	2.40E-23
I1	mi|Xelaev18003033m.g|	wnt5a.S	WNT5A	84.19	55.02	-5.11	0.90	1.47E-16
I1	mi|Xelaev18025949m.g|	loc100498463.S	N/B	83.81	63.11	-9.61	0.96	1.57E-24
I1	mi|Xelaev18027985m.g|	ccl34.L	N/B	83.20	58.88	-3.64	0.94	1.32E-20
I1	mi|Xelaev18041405m.g|	timp-1.S	TIMP1	83.02	33.26	5.81	0.94	9.58E-22
I1	mi|Xelaev18041771m.g|	fam129b.S	FAM129B	81.77	55.77	-3.31	0.95	9.72E-24
I1	mi|Xelaev18013645m.g|	lima1.L	LIMA1	78.29	69.22	-10.94	0.92	3.15E-19
I1	mi|Xelaev18022186m.g|	rassf7.L	RASSF8	78.26	43.96	-0.77	0.94	5.36E-22

I1	mi|Xelaev18008304m.g|	itga2.L	ITGA2	76.89	50.05	-7.35	0.96	1.03E-24
I1	mi|Xelaev18007696m.g|	cldn5.L	CLDN5	76.34	51.19	-11.20	0.87	3.68E-14
I1	mi|Xelaev18002370m.g|	tnfrsf12a	TNFRSF12A	76.05	61.04	-11.31	0.91	5.97E-18
I1	mi|Xelaev18043629m.g|	tnfrsf6b.L	TNFRSF6B	75.04	47.72	-8.99	0.91	6.87E-18
I1	mi|Xelaev18027421m.g|	chrd.2.L	CHRD	74.80	58.42	-4.76	0.94	1.72E-21
I1	mi|Xelaev18012752m.g|	ccl4.L	CCL4L1	73.84	51.79	-8.96	0.83	2.22E-12
I1	mi|Xelaev18011164m.g|	ccl19.S	CCL19	73.22	35.42	2.86	0.91	1.26E-17
I1	mi|Xelaev18023847m.g|	manf.L	MANF	72.79	64.43	22.34	0.95	4.17E-22
I1	mi|Xelaev18037758m.g|	Xelaev18037758m.g	NECTIN2	69.34	58.80	-0.95	0.95	1.07E-23
I1	mi|Xelaev18007632m.g|	loc100493155.L	C22orf15	65.82	57.83	6.82	0.91	7.80E-18
I1	mi|Xelaev18037390m.g|	tagln.S	TAGLN	65.72	56.71	-7.80	0.89	8.26E-16
I1	mi|Xelaev18027435m.g|	loc100490020.L	N/B	65.55	48.23	-9.24	0.94	6.15E-22
I1	mi|Xelaev18041161m.g|	inf2.S	INF2	65.50	43.64	-4.87	0.92	4.29E-19
I1	mi|Xelaev18043108m.g|	krt17.L	KRT17	65.16	38.35	-7.86	0.91	6.60E-18
I1	mi|Xelaev18037781m.g|	rras.S	RRAS2	64.40	33.33	-4.46	0.88	4.25E-15
I1	mi|Xelaev18029868m.g|	loc100490428-like.S	DBNDD2	64.31	44.89	-8.99	0.92	9.94E-19
I1	mi|Xelaev18018132m.g|	gcnt3.L	GCNT3	63.38	51.82	-5.24	0.88	1.58E-15
I1	mi|Xelaev18041455m.g|	ambp.S	AMBP	63.09	46.61	-4.36	0.89	3.59E-16
I1	mi|Xelaev18036515m.g|	fxyd5.L	N/B	62.30	23.60	6.97	0.86	4.24E-14
I1	mi|Xelaev18002900m.g|	msn.L	MSN	62.27	48.53	-5.96	0.87	1.28E-14
I1	mi|Xelaev18044026m.g|	slc9a3r1.L	SLC9A3R1	62.06	37.97	3.32	0.88	5.23E-15
I1	mi|Xelaev18023924m.g|	lmcd1.L	LMCD1	61.52	21.44	11.87	0.93	8.98E-20
I1	mi|Xelaev18031953m.g|	cdh2.L	CDH2	61.16	28.85	-2.46	0.83	2.34E-12
I1	mi|Xelaev18041034m.g|	actn1.S	ACTN1	60.86	56.46	-8.79	0.87	2.73E-14
I1	mi|Xelaev18030736m.g|	svil.L	SVIL	60.81	20.62	6.68	0.91	2.56E-17
I1	mi|Xelaev18026597m.g|	polr1b.L	POLR1B	60.30	64.04	18.33	0.88	4.65E-15

I1	mi|Xelaev18023809m.g|	mst1.L	N/B	59.48	35.29	-7.78	0.91	7.97E-18
I1	mi|Xelaev18042667m.g|	thbs1.S	THBS1	58.92	31.12	-6.62	0.82	1.57E-11
I1	mi|Xelaev18030727m.g|	itgb1.L	ITGB1	58.84	33.33	-8.16	0.95	5.23E-22
I1	mi|Xelaev18046795m.g|	metrnl.S	METRNL	58.33	52.42	-5.45	0.92	9.23E-19
I1	mi|Xelaev18046913m.g|	Xelaev18046913m.g	PARP14	57.66	36.57	-6.19	0.91	2.81E-17
I1	mi|Xelaev18018790m.g|	plat.L	PLAT	57.03	41.54	-10.63	0.88	7.69E-15
I1	mi|Xelaev18011873m.g|	aifm3-like.L	AIFM3	57.00	44.23	-9.68	0.85	5.39E-13
I1	mi|Xelaev18037885m.g|	myadm.S	MYADM	56.72	34.67	-7.60	0.90	4.27E-17
I1	mi|Xelaev18036474m.g|	kirrel2.L	KIRREL1	56.56	30.74	-5.20	0.89	1.51E-15
I1	mi|Xelaev18023533m.g|	myh9.L	MYH9	56.49	54.54	-5.41	0.92	1.86E-18
I1	mi|Xelaev18042284m.g|	ednrb2.S	EDNRB	55.38	58.02	6.73	0.88	1.81E-15
I1	mi|Xelaev18037564m.g|	errfi1.S	ERRFI1	55.19	51.25	-6.32	0.83	4.95E-12
I1	mi|Xelaev18036047m.g|	rras.L	RRAS2	54.90	35.31	-9.55	0.85	2.50E-13
I1	mi|Xelaev18014318m.g|	rcan1.S	RCAN1	54.61	35.58	-5.51	0.90	1.78E-16
I1	mi|Xelaev18037014m.g|	noc3l.S	NOC3L	53.01	58.18	24.70	0.91	6.32E-18
I1	mi|Xelaev18016257m.g|	mmp13.S	MMP13	52.69	29.73	-7.07	0.91	3.82E-18
I1	mi|Xelaev18033969m.g|	tnfrsf11b.S	TNFRSF11B	52.48	17.52	5.22	0.76	1.72E-09
I1	mi|Xelaev18012754m.g|	ccl42d.L	CCL4L1	51.68	51.28	0.33	0.80	7.14E-11
I1	mi|Xelaev18020453m.g|	chsy1.S	CHSY1	51.66	35.39	-8.06	0.93	2.67E-19
I1	mi|Xelaev18023313m.g|	arhgap29.L	ARHGAP29	51.65	32.88	-14.94	0.88	7.43E-15
I1	mi|Xelaev18033717m.g|	rgs20.S	RGS20	51.49	35.64	-10.20	0.88	2.88E-15
I1	mi|Xelaev18014128m.g|	loc733316.S	CD99	51.45	38.26	-7.06	0.87	2.06E-14
I1	mi|Xelaev18046223m.g|	loc101731830.S	BCAR3	51.40	46.65	-9.69	0.86	1.08E-13
I1	mi|Xelaev18002841m.g|	Xelaev18002841m.g	FLNA	51.38	34.22	-10.72	0.91	2.09E-17
I1	mi|Xelaev18017304m.g|	pdlim7.L	PDLIM7	51.26	37.15	-8.19	0.85	4.11E-13
I1	mi|Xelaev18014321m.g|	runx1.S	RUNX1	51.07	35.51	-8.16	0.88	4.09E-15

I1	mi|Xelaev18042752m.g|	plekho1.S	PLEKHO1	49.85	17.82	-4.07	0.77	1.10E-09
I1	mi|Xelaev18020263m.g|	ddx56.S	DDX56	49.83	49.75	4.64	0.88	1.86E-15
I1	mi|Xelaev18026332m.g|	wdr43.L	WDR43	48.92	30.70	-9.79	0.87	3.41E-14
I1	mi|Xelaev18011753m.g|	MGC115708.L	CD99	48.51	20.44	-0.49	0.91	1.96E-17
I1	mi|Xelaev18027420m.g|	chrd.1.L	CHRD	48.29	38.89	-10.66	0.90	1.76E-16
I1	mi|Xelaev18012947m.g|	il1r2.L	IL1R2	48.11	28.22	-5.59	0.91	2.37E-17
I1	mi|Xelaev18025550m.g|	myh9.S	MYH9	47.56	56.74	4.85	0.89	7.39E-16
I1	mi|Xelaev18026425m.g|	rsph9.L	RSPH9	47.01	33.33	1.01	0.79	1.85E-10
I1	mi|Xelaev18018454m.g|	smad3.L	SMAD3	46.94	25.83	-9.68	0.80	8.19E-11
I1	mi|Xelaev18031194m.g|	inhba.L	INHBA	45.89	26.54	-8.70	0.81	2.55E-11
I1	mi|Xelaev18006910m.g|	gcnt1.L	GCNT1	45.69	27.03	-9.51	0.88	7.20E-15
I1	mi|Xelaev18036965m.g|	pprc1-like.S	PPRC1	44.83	53.89	13.52	0.89	3.55E-16
I1	mi|Xelaev18016263m.g|	mmp1.S	N/B	44.79	39.84	-6.56	0.90	6.37E-17
I1	mi|Xelaev18008990m.g|	tbc1d1.S	TBC1D1	44.59	26.72	-3.72	0.88	7.04E-15
I1	mi|Xelaev18036170m.g|	ccdc63.L	CCDC63	44.57	31.62	-8.08	0.85	1.73E-13
I1	mi|Xelaev18021305m.g|	dram1.S	DRAM1	44.41	39.63	-1.06	0.89	4.16E-16
I1	mi|Xelaev18003274m.g|	chrng.L	CHRNG	44.39	46.44	-7.08	0.89	6.59E-16
I1	mi|Xelaev18030711m.g|	pitrm1.L	PITRM1	44.25	48.28	5.63	0.90	2.54E-16
I1	mi|Xelaev18017422m.g|	nuak1.L	NUAK1	44.13	37.37	-11.63	0.87	1.07E-14
I1	mi|Xelaev18010898m.g|	f2rl2.S	F2RL2	40.86	32.51	-12.28	0.86	8.68E-14
I1	mi|Xelaev18029825m.g|	plod2.S	PLOD2	40.20	38.61	-9.22	0.84	1.27E-12
I1	mi|Xelaev18034677m.g|	pprc1-like.L	PPRC1	39.95	60.69	21.17	0.92	2.02E-18
I1	mi|Xelaev18046673m.g|	slc9a3r1.S	SLC9A3R1	39.65	33.06	1.01	0.89	6.29E-16
I1	mi|Xelaev18038794m.g|	loc100492639-like.L	KCNE5	39.48	26.00	-10.67	0.88	1.61E-15
I1	mi|Xelaev18032266m.g|	fzd6.L	FZD6	39.35	44.36	-6.99	0.92	1.21E-18
I1	mi|Xelaev18016812m.g|	loc100496642-like.L	SLC45A3	39.14	54.31	7.79	0.75	3.74E-09

I1	mi|Xelaev18025380m.g|	arhgap29.S	ARHGAP29	38.89	34.41	-10.70	0.85	2.68E-13
I1	mi|Xelaev18042942m.g|	c2cd4d.S	C2CD4C	38.70	37.49	-9.18	0.91	2.45E-17
I1	mi|Xelaev18026176m.g|	Xelaev18026176m.g	ABCB5	38.50	23.13	-9.79	0.87	2.26E-14
I1	mi|Xelaev18043548m.g|	ctsz.L	CTSZ	38.23	29.89	-10.23	0.83	3.09E-12
I1	mi|Xelaev18019577m.g|	sox15.L	SOX15	38.06	14.81	-4.82	0.82	8.38E-12
I1	mi|Xelaev18047015m.g|	loc100497528.S	TUBA1A	37.99	14.46	-5.90	0.82	1.61E-11
I1	mi|Xelaev18028091m.g|	nt5c1a.L	NT5C1A	37.96	36.31	-5.22	0.73	1.99E-08
I1	mi|Xelaev18013609m.g|	krt18.L	KRT18	37.81	44.93	6.09	0.87	1.12E-14
I1	mi|Xelaev18009561m.g|	sgk223.S	PRAG1	37.79	32.01	-12.54	0.86	1.17E-13
I1	mi|Xelaev18026601m.g|	dzank1.L	DZANK1	37.47	38.22	-4.19	0.88	6.10E-15
I1	mi|Xelaev18016071m.g|	krt18.S	KRT18	37.38	47.54	12.95	0.83	4.51E-12
I1	mi|Xelaev18021801m.g|	adm.L	ADM	36.99	35.90	-9.32	0.87	2.06E-14
I1	mi|Xelaev18020018m.g|	ctnna1.S	CTNNA1	36.86	25.53	-11.99	0.71	8.36E-08
I1	mi|Xelaev18021522m.g|	hbegf.S	HBEGF	36.73	40.98	4.65	0.74	1.32E-08
I1	mi|Xelaev18023312m.g|	f3.L	F3	36.60	37.47	-8.31	0.77	1.30E-09
I1	mi|Xelaev18038898m.g|	utp14a.L	UTP14A	36.27	38.17	6.75	0.78	3.35E-10
I1	mi|Xelaev18008403m.g|	grp.L	GRP	35.99	33.54	-6.64	0.74	1.09E-08
I1	mi|Xelaev18025555m.g|	hmox1.S	HMOX1	35.78	19.28	-8.72	0.91	6.62E-18
I1	mi|Xelaev18045011m.g|	wdr75.L	WDR75	35.41	45.47	12.10	0.86	5.66E-14
I1	mi|Xelaev18043099m.g|	jup.L	JUP	35.32	28.38	-11.02	0.78	4.46E-10
I1	mi|Xelaev18043463m.g|	sall4.L	SALL4	35.08	27.45	-12.23	0.87	1.28E-14
I1	mi|Xelaev18005047m.g|	cfap99.L	CFAP99	34.96	22.60	-6.86	0.82	8.93E-12
I1	mi|Xelaev18014129m.g|	mxra5.S	MXRA5	34.96	21.57	-10.63	0.78	5.99E-10
I1	mi|Xelaev18032888m.g|	armc4.S	ARMC4	34.58	17.83	-8.36	0.79	1.24E-10
I1	mi|Xelaev18020589m.g|	homer2.S	HOMER2	34.44	32.20	-5.72	0.88	6.05E-15
I1	mi|Xelaev18021364m.g|	hk2.S	HK2	34.28	22.75	-10.54	0.75	3.44E-09

I1	mi|Xelaev18013369m.g|	erbb3.L	ERBB3	33.99	18.33	-9.30	0.73	1.98E-08
I1	mi|Xelaev18033773m.g|	c8orf46.S	N/B	33.79	24.02	-9.97	0.65	1.69E-06
I1	mi|Xelaev18017788m.g|	ptpn12.L	N/B	33.66	45.62	8.34	0.86	3.97E-14
I1	mi|Xelaev18008531m.g|	il11ra.L	IL11RA	33.61	17.33	-10.79	0.88	2.93E-15
I1	mi|Xelaev18038628m.g|	loc100145115.L	STARD10	33.13	36.42	2.83	0.80	9.22E-11
I1	mi|Xelaev18038251m.g|	Xelaev18038251m.g	FCN1	33.07	34.28	-4.97	0.77	1.46E-09
I1	mi|Xelaev18028139m.g|	tnfrsf21.L	TNFRSF21	32.77	20.60	-10.93	0.86	5.73E-14
I1	mi|Xelaev18026607m.g|	snx5.L	SNX5	32.76	23.50	-8.05	0.71	5.56E-08
I1	mi|Xelaev18011697m.g|	rab34.L	RAB34	32.59	24.91	-5.72	0.84	1.21E-12
I1	mi|Xelaev18014496m.g|	ptgfrn.S	PTGFRN	32.50	23.81	-8.97	0.79	2.15E-10
I1	mi|Xelaev18005289m.g|	kdr.L	KDR	32.39	27.06	-8.88	0.86	9.40E-14
I1	mi|Xelaev18026428m.g|	srf.L	SRF	31.56	16.93	-7.25	0.65	1.80E-06
I1	mi|Xelaev18020231m.g|	adam28.S	ADAM28	31.05	12.05	-6.33	0.80	7.10E-11
I1	mi|Xelaev18013190m.g|	irg1.L	ACOD1	30.93	31.50	-6.31	0.76	2.12E-09
I1	mi|Xelaev18016349m.g|	prss23.S	PRSS23	30.76	14.48	-9.40	0.79	2.69E-10
I1	mi|Xelaev18014768m.g|	sytl5.S	SYTL5	30.76	15.66	-8.67	0.65	1.69E-06
I1	mi|Xelaev18005548m.g|	ucp1.L	N/B	29.98	11.68	-8.06	0.80	9.46E-11
I1	mi|Xelaev18022156m.g|	ehd1.L	EHD1	29.71	47.91	10.10	0.90	1.00E-16
I1	mi|Xelaev18020683m.g|	Xelaev18020683m.g	MYO1E	29.57	24.11	-9.99	0.83	4.29E-12
I1	mi|Xelaev18044394m.g|	loc100496446.L	MRAS	29.57	38.82	-3.98	0.79	2.36E-10
I1	mi|Xelaev18019841m.g|	etv6.S	ETV6	29.55	15.80	-16.22	0.82	1.45E-11
I1	mi|Xelaev18040535m.g|	crabp2.L	CRABP2	29.46	22.13	-4.16	0.75	3.75E-09
I1	mi|Xelaev18008425m.g|	ccdc68.L	CCDC68	29.30	41.81	4.35	0.86	8.70E-14
I1	mi|Xelaev18011172m.g|	dnajb5-like.S	DNAJB5	29.25	42.69	5.37	0.81	3.71E-11
I1	mi|Xelaev18029887m.g|	sept2.S	37500	29.20	31.03	-3.79	0.82	1.22E-11
I1	mi|Xelaev18046311m.g|	Xelaev18046311m.g	DEPTOR	29.03	23.91	-8.05	0.79	2.45E-10

I1	mi|Xelaev18026141m.g|	Xelaev18026141m.g	RHOQ	28.96	41.62	-5.19	0.81	2.65E-11
I1	mi|Xelaev18021137m.g|	pawr.S	PAWR	28.84	20.85	-10.33	0.85	3.96E-13
I1	mi|Xelaev18038421m.g|	crb2.L	CRB2	28.71	23.32	-4.13	0.82	6.89E-12
I1	mi|Xelaev18009362m.g|	arsj.S	N/B	28.18	26.86	-11.56	0.86	6.82E-14
I1	mi|Xelaev18032744m.g|	ccm2.S	CCM2	28.00	30.83	-7.69	0.78	3.94E-10
I1	mi|Xelaev18031526m.g|	cdh6.L	CDH6	27.79	14.86	-6.53	0.81	4.35E-11
I1	mi|Xelaev18015134m.g|	loc100485272-like.S	ANGPT2	27.77	15.94	-10.59	0.65	2.17E-06
I1	mi|Xelaev18018158m.g|	Xelaev18018158m.g	MYO1E	26.90	14.05	-10.20	0.77	1.29E-09
I1	mi|Xelaev18047027m.g|	ttll4.S	TTLL4	26.72	32.69	-6.20	0.83	3.44E-12
I1	mi|Xelaev18036418m.g|	pqlc2.L	PQLC2	26.57	26.38	-12.31	0.91	1.54E-17
I1	mi|Xelaev18034251m.g|	afap1l2.L	AFAP1L2	26.53	37.74	-3.57	0.86	5.34E-14
I1	mi|Xelaev18004443m.g|	ccdc151.L	CCDC151	26.53	24.25	-6.74	0.80	7.78E-11
I1	mi|Xelaev18012795m.g|	ncf1.L	NCF1C	26.48	25.10	-8.59	0.83	4.71E-12
I1	mi|Xelaev18044527m.g|	pnkd.L	PNKD	26.33	28.01	5.24	0.83	3.48E-12
I1	mi|Xelaev18047501m.g|	gspt1.S	GSPT1	26.15	36.67	7.36	0.80	9.87E-11
I1	mi|Xelaev18010916m.g|	lhfpl2.S	LHFPL2	25.82	29.44	-4.20	0.80	4.70E-11
I1	mi|Xelaev18029097m.g|	tnfaip3.S	TNFAIP3	25.26	18.01	-10.15	0.73	1.92E-08
I1	mi|Xelaev18041763m.g|	Xelaev18041763m.g	FLNA	25.11	17.20	-9.61	0.84	6.02E-13
I1	mi|Xelaev18025818m.g|	slurp1l.S	N/B	25.10	14.81	-8.88	0.76	2.13E-09
I1	mi|Xelaev18036171m.g|	myadm.L	MYADM	24.68	24.52	-11.28	0.76	1.51E-09
I1	mi|Xelaev18028224m.g|	efhc1.L	EFHC1	24.52	20.94	-11.00	0.81	2.51E-11
I1	mi|Xelaev18010899m.g|	f2r.S	F2R	24.49	18.99	-6.50	0.73	1.79E-08
I1	mi|Xelaev18032013m.g|	rgs20.L	RGS20	24.20	13.20	-10.18	0.85	1.62E-13
I1	mi|Xelaev18015457m.g|	egfl6.S	EGFL6	24.02	31.38	-3.05	0.73	2.08E-08
I1	mi|Xelaev18018318m.g|	unc45a.L	UNC45A	23.83	45.18	18.18	0.81	3.80E-11
I1	mi|Xelaev18001387m.g|	Xelaev18001387m.g	ZNF510	23.22	27.24	-4.80	0.74	7.03E-09

I1	mi|Xelaev18022681m.g|	c9orf117.L	CFAP157	23.18	11.97	-5.53	0.77	1.13E-09
I1	mi|Xelaev18029210m.g|	ankrd60-like.S	ANKRD60	22.44	23.74	-7.72	0.79	2.64E-10
I1	mi|Xelaev18027042m.g|	rsph3.L	RSPH3	21.90	10.45	-4.64	0.77	9.67E-10
I1	mi|Xelaev18045253m.g|	il21r.L	IL21R	21.75	13.55	-11.87	0.76	2.41E-09
I1	mi|Xelaev18045172m.g|	gspt1.L	GSPT1	21.23	23.44	2.10	0.77	1.19E-09
I1	mi|Xelaev18027603m.g|	lrrc34.L	LRRC34	21.08	21.26	-8.49	0.80	6.44E-11
I1	mi|Xelaev18004560m.g|	Xelaev18004560m.g	URGCP	20.88	12.54	-11.50	0.77	1.48E-09
I1	mi|Xelaev18044336m.g|	dtx3l-like.L	DTX3L	20.80	22.87	-10.89	0.86	6.08E-14
I1	mi|Xelaev18010354m.g|	lif.S	N/B	20.49	23.98	-7.27	0.78	3.02E-10
I1	mi|Xelaev18033963m.g|	ext1.S	EXT1	20.41	11.92	-11.60	0.72	3.52E-08
I1	mi|Xelaev18021822m.g|	tspan18.L	TSPAN18	20.28	23.39	-6.52	0.75	3.24E-09
I1	mi|Xelaev18041922m.g|	msn.S	MSN	20.22	16.88	-11.29	0.81	3.13E-11
I1	mi|Xelaev18006614m.g|	gatad2a.L	GATAD2A	20.16	23.75	0.89	0.67	7.57E-07
I1	mi|Xelaev18025357m.g|	soat1.S	SOAT1	19.78	25.40	-5.93	0.78	2.87E-10
I1	mi|Xelaev18041868m.g|	loc100493411.S	N/B	19.44	17.99	-11.41	0.70	1.04E-07
I1	mi|Xelaev18039912m.g|	fntb.L	FNTB	19.40	39.87	12.10	0.76	1.54E-09
I1	mi|Xelaev18022773m.g|	loc100494152.L	GPT2	19.02	23.07	-2.49	0.69	2.65E-07
I1	mi|Xelaev18035927m.g|	sphk2.L	SPHK2	18.77	25.27	-6.29	0.83	5.61E-12
I1	mi|Xelaev18012810m.g|	p2rx1.L	P2RX1	18.54	25.36	-7.85	0.81	1.96E-11
I1	mi|Xelaev18033982m.g|	has2.S	HAS2	18.44	11.04	-11.79	0.84	1.03E-12
I1	mi|Xelaev18006264m.g|	prtn3.L	PRTN3	18.31	11.77	-10.92	0.75	3.45E-09
I1	mi|Xelaev18044177m.g|	metrnl.L	METRNL	17.84	19.96	-6.33	0.72	3.45E-08
I1	mi|Xelaev18006266m.g|	ptbp1.L	PTBP1	17.77	32.35	-3.06	0.79	1.69E-10
I1	mi|Xelaev18000573m.g|	vcl.S	VCL	17.77	22.31	-8.35	0.88	7.36E-15
I1	mi|Xelaev18011956m.g|	camk1g.L	CAMK1G	17.75	16.63	-13.86	0.81	1.89E-11
I1	mi|Xelaev18025779m.g|	sema3b.S	SEMA3B	17.69	42.08	11.27	0.78	4.50E-10

I1	mi|Xelaev18012341m.g|	slc16a1.L	SLC16A1	17.62	44.60	23.78	0.81	1.66E-11
I1	mi|Xelaev18017616m.g|	dnajb9.L	DNAJB9	17.32	18.98	-8.84	0.84	1.60E-12
I1	mi|Xelaev18016945m.g|	larp1.L	LARP1	17.11	21.47	-3.51	0.72	3.25E-08
I1	mi|Xelaev18037757m.g|	bcam.S	BCAM	16.96	15.07	-7.35	0.68	3.92E-07
I1	mi|Xelaev18005470m.g|	sfrp2.L	SFRP2	16.64	24.41	-1.16	0.75	3.90E-09
I1	mi|Xelaev18033681m.g|	Xelaev18033681m.g	B4GALT6	15.93	18.23	-7.46	0.72	3.24E-08
I1	mi|Xelaev18013610m.g|	krt8.L	KRT8	15.74	24.75	-5.30	0.81	2.21E-11
I1	mi|Xelaev18009793m.g|	prtn3.S	PRTN3	15.41	11.59	-9.71	0.70	1.19E-07
I1	mi|Xelaev18037759m.g|	Xelaev18037759m.g	N/B	14.65	10.99	-9.49	0.75	4.95E-09
I1	mi|Xelaev18013657m.g|	trim13.L	TRIM13	14.63	27.07	3.41	0.84	8.00E-13
I1	mi|Xelaev18012375m.g|	mid1.L	MID1	14.04	21.54	-6.58	0.71	8.48E-08
I1	mi|Xelaev18047380m.g|	Xelaev18047380m.g	CREB1	13.42	17.68	-7.67	0.61	1.27E-05
I1	mi|Xelaev18001915m.g|	slc7a5.S	SLC7A5	13.19	28.10	-0.65	0.77	1.41E-09
I1	mi|Xelaev18037870m.g|	cyth1-like.S	CYTH2	13.07	15.94	-9.86	0.69	1.73E-07
I1	mi|Xelaev18005291m.g|	tmem165.L	TMEM165	13.01	13.54	-2.03	0.76	3.00E-09
I1	mi|Xelaev18046813m.g|	cfap52.S	CFAP52	12.97	15.83	-2.91	0.71	5.12E-08
I1	mi|Xelaev18039467m.g|	ttll5.L	TTLL5	12.62	20.12	-5.04	0.70	1.19E-07
I1	mi|Xelaev18010286m.g|	Xetrov90002579m.S	CRYBB2	12.56	41.67	11.21	0.83	4.23E-12
I1	mi|Xelaev18043951m.g|	rbm12.L	RBM12	12.47	23.73	-0.83	0.63	4.06E-06
I1	mi|Xelaev18007687m.g|	Xelaev18007687m.g	N/B	12.19	12.55	-10.03	0.72	3.73E-08
I1	mi|Xelaev18041277m.g|	ak7.S	AK7	11.87	12.71	0.79	0.71	6.98E-08
I1	mi|Xelaev18031583m.g|	slc22a23.L	SLC22A23	11.73	29.55	3.38	0.75	5.83E-09
I1	mi|Xelaev18001214m.g|	nans.S	NANS	11.64	16.11	-1.93	0.74	1.03E-08
I1	mi|Xelaev18023653m.g|	dnal4.L	DNAL4	11.55	17.37	-6.18	0.82	1.05E-11
I1	mi|Xelaev18011823m.g|	mpo.L	EPX	11.31	10.13	-7.68	0.81	3.25E-11
I1	mi|Xelaev18039676m.g|	pygl.L	N/B	10.77	24.60	0.91	0.70	1.40E-07

I1	mi|Xelaev18020614m.g|	shc4.S	SHC4	10.55	14.92	-16.06	0.56	7.29E-05
I2	mi|Xelaev18021375m.g|	pdlim7.S	PDLIM7	43.66	50.47	-3.03	0.88	4.06E-15
I2	mi|Xelaev18025422m.g|	clca3p-like.S	CLCA4	42.24	10.51	2.24	0.74	1.14E-08
I2	mi|Xelaev18021627m.g|	loc100494743.S	ICAM5	38.42	16.45	-5.75	0.83	5.33E-12
I2	mi|Xelaev18013257m.g|	nufip1.L	NUFIP1	36.15	44.46	5.78	0.90	3.70E-17
I2	mi|Xelaev18018944m.g|	adgre5.L	ADGRE5	33.04	13.18	-2.14	0.88	4.71E-15
I2	mi|Xelaev18021991m.g|	ppp1r32.L	PPP1R32	32.52	24.20	-11.21	0.79	2.26E-10
I2	mi|Xelaev18027763m.g|	wwtr1.L	WWTR1	30.01	10.03	-3.82	0.89	8.05E-16
I2	mi|Xelaev18014304m.g|	ifngr2.2.S	N/B	29.90	22.33	-12.75	0.89	3.00E-16
I2	mi|Xelaev18018455m.g|	Xelaev18018455m.g	N/B	29.57	20.56	-10.86	0.60	1.74E-05
I2	mi|Xelaev18023508m.g|	emp1.L	EMP1	28.18	19.67	-12.11	0.76	2.11E-09
I2	mi|Xelaev18034459m.g|	dnttip2.L	DNTTIP2	27.33	42.82	11.61	0.92	3.40E-18
I2	mi|Xelaev18008376m.g|	rai14.L	RAI14	27.27	40.38	4.52	0.78	4.23E-10
I2	mi|Xelaev18046166m.g|	snai1.S	SNAI2	26.82	23.82	-11.76	0.66	9.71E-07
I2	mi|Xelaev18047243m.g|	frzb.S	FRZB	25.98	35.19	4.45	0.81	3.36E-11
I2	mi|Xelaev18044665m.g|	ccdc173.L	CCDC173	25.90	32.40	-5.11	0.93	2.44E-19
I2	mi|Xelaev18038343m.g|	loc100487399.L	PHF19	25.03	26.40	-11.09	0.80	4.88E-11
I2	mi|Xelaev18005059m.g|	nop14.L	NOP14	24.02	27.39	-4.46	0.82	9.98E-12
I2	mi|Xelaev18008154m.g|	utp15.L	UTP15	23.02	22.29	-6.10	0.89	1.34E-15
I2	mi|Xelaev18035579m.g|	noc2l.L	NOC2L	22.54	39.19	5.28	0.94	5.62E-21
I2	mi|Xelaev18006390m.g|	tpm4.L	TPM4	22.50	25.92	-7.25	0.86	4.54E-14
I2	mi|Xelaev18023668m.g|	rps19bp1.L	RPS19BP1	22.40	20.92	-4.31	0.64	3.36E-06
I2	mi|Xelaev18023969m.g|	ccdc37.L	CFAP100	20.96	16.40	-10.08	0.80	4.88E-11
I2	mi|Xelaev18003217m.g|	srsf10.S	SRSF10	20.61	31.47	-1.49	0.84	7.74E-13
I2	mi|Xelaev18002851m.g|	fam129b.L	FAM129B	20.46	10.76	-14.05	0.75	5.05E-09
I2	mi|Xelaev18005436m.g|	naf1.L	NAF1	20.43	26.53	-5.55	0.90	4.46E-17

I2	mi|Xelaev18026073m.g|	trmt6.L	TRMT6	18.98	32.28	9.59	0.82	9.34E-12
I2	mi|Xelaev18009894m.g|	tpm4.S	TPM4	18.17	16.73	-7.65	0.84	8.81E-13
I2	mi|Xelaev18021622m.g|	ppan.S	PPAN	18.08	26.01	-3.17	0.91	1.97E-17
I2	mi|Xelaev18010555m.g|	ranbp1.S	RANBP1	17.92	24.94	9.21	0.84	8.83E-13
I2	mi|Xelaev18009441m.g|	eif4e.S	EIF4E	17.91	26.59	12.96	0.73	1.51E-08
I2	mi|Xelaev18026236m.g|	rrp15.L	RRP15	17.74	22.43	-3.04	0.86	1.18E-13
I2	mi|Xelaev18007593m.g|	rab36.L	RAB36	17.70	26.80	3.20	0.85	1.76E-13
I2	mi|Xelaev18041226m.g|	bmp4.S	BMP4	17.66	17.07	-6.54	0.78	3.41E-10
I2	mi|Xelaev18044115m.g|	sept9.L	40057	17.49	12.81	-9.00	0.85	2.94E-13
I2	mi|Xelaev18020823m.g|	sec11a.S	SEC11A	17.28	20.36	11.63	0.74	6.66E-09
I2	mi|Xelaev18030656m.g|	wdr86.L	WDR86	17.14	24.22	-8.51	0.74	9.09E-09
I2	mi|Xelaev18046460m.g|	ccdc137.S	CCDC137	17.02	26.58	6.80	0.82	1.19E-11
I2	mi|Xelaev18021628m.g|	Xelaev18021628m.g	N/B	16.97	27.80	-5.25	0.82	9.09E-12
I2	mi|Xelaev18033370m.g|	znf622.S	ZNF622	16.95	22.45	11.91	0.78	4.17E-10
I2	mi|Xelaev18003402m.g|	Xelaev18003402m.g	N/B	16.37	25.59	5.55	0.70	1.48E-07
I2	mi|Xelaev18030680m.g|	nom1.L	NOM1	16.09	26.09	-6.93	0.83	5.29E-12
I2	mi|Xelaev18047406m.g|	wdr75.S	WDR75	15.92	36.11	7.59	0.83	4.26E-12
I2	mi|Xelaev18045794m.g|	Xelaev18045794m.g	N/B	15.71	20.32	-9.28	0.81	3.07E-11
I2	mi|Xelaev18031827m.g|	tubb6.L	TUBB6	15.44	30.83	-0.78	0.82	7.25E-12
I2	mi|Xelaev18007432m.g|	ddx54.L	DDX54	14.65	23.14	-4.39	0.83	2.33E-12
I2	mi|Xelaev18012601m.g|	clic5.L	CLIC4	14.61	21.13	-8.55	0.83	3.67E-12
I2	mi|Xelaev18021486m.g|	pdlim4.S	PDLIM4	13.90	30.57	6.05	0.76	2.70E-09
I2	mi|Xelaev18005070m.g|	lrpap1.L	LRPAP1	13.86	15.79	-5.07	0.69	2.44E-07
I2	mi|Xelaev18027150m.g|	dse.L	DSE	13.50	22.34	-5.87	0.83	2.73E-12
I2	mi|Xelaev18003586m.g|	aprt.L	APRT	13.12	14.46	2.05	0.84	7.11E-13
I2	mi|Xelaev18015116m.g|	snrnp40.S	SNRNP40	13.00	11.44	-7.13	0.79	1.79E-10

I2	mi|Xelaev18020405m.g|	eif3j.S	EIF3J	12.99	26.39	12.51	0.91	5.55E-18
I2	mi|Xelaev18018994m.g|	ppan.L	PPAN	11.92	18.06	-9.72	0.86	7.58E-14
I2	mi|Xelaev18025709m.g|	nol12.S	NOL12	11.49	25.52	5.14	0.84	1.23E-12
I2	mi|Xelaev18017584m.g|	rpap3.L	RPAP3	10.85	15.18	3.23	0.68	4.20E-07
I2	mi|Xelaev18016649m.g|	arfgap3.L	ARFGAP3	10.77	11.47	-8.76	0.70	1.55E-07
I3	mi|Xelaev18038249m.g|	Xelaev18038249m.g	FCN1	78.36	43.62	-4.83	0.86	6.44E-14
I3	mi|Xelaev18047360m.g|	nop58.S	NOP58	67.94	56.45	20.50	0.97	1.08E-27
I3	mi|Xelaev18010203m.g|	ror2.S	ROR2	66.03	40.55	-7.94	0.86	1.23E-13
I3	mi|Xelaev18044939m.g|	nop58.L	NOP58	63.74	58.19	17.21	0.98	3.64E-30
I3	mi|Xelaev18037983m.g|	mrto4.S	MRTO4	61.77	51.65	10.34	0.94	4.96E-21
I3	mi|Xelaev18011719m.g|	tsr1.L	TSR1	59.88	49.37	13.21	0.96	6.43E-24
I3	mi|Xelaev18047541m.g|	lfng.S	LFNG	58.69	38.63	-10.55	0.85	4.10E-13
I3	mi|Xelaev18034422m.g|	nop2.L	NOP2	56.58	53.25	9.23	0.96	1.84E-25
I3	mi|Xelaev18012031m.g|	stpg1.L	STPG1	55.39	48.55	-2.87	0.87	8.77E-15
I3	mi|Xelaev18009119m.g|	lrp2bp.S	LRP2BP	54.70	47.52	5.30	0.89	7.46E-16
I3	mi|Xelaev18047992m.g|	loc398467.S	VKORC1L1	54.58	51.61	8.28	0.93	2.73E-20
I3	mi|Xelaev18003168m.g|	abcf2.L	ABCF2	53.23	52.09	8.44	0.94	2.59E-21
I3	mi|Xelaev18045175m.g|	rsl1d1.L	RSL1D1	52.81	47.53	8.95	0.96	3.46E-25
I3	mi|Xelaev18036956m.g|	npm3.S	NPM3	52.21	56.22	16.59	0.95	3.53E-23
I3	mi|Xelaev18001058m.g|	eif4a1.S	EIF4A1	51.82	53.70	17.65	0.92	5.49E-19
I3	mi|Xelaev18019581m.g|	eif4a1.L	EIF4A1	51.28	56.07	17.71	0.96	2.72E-24
I3	mi|Xelaev18012935m.g|	pdcl3.L	PDCL3	50.85	56.12	24.38	0.91	1.29E-17
I3	mi|Xelaev18030282m.g|	c2orf70.S	C2orf70	50.27	27.36	-7.23	0.80	5.15E-11
I3	mi|Xelaev18022633m.g|	utp4.L	UTP4	50.17	51.85	17.49	0.90	1.85E-16
I3	mi|Xelaev18025900m.g|	ruvbl1.S	RUVBL1	49.97	43.98	12.11	0.93	7.17E-20
I3	mi|Xelaev18005098m.g|	wfs1.L	WFS1	49.95	45.61	-10.02	0.86	6.92E-14

I3	mi|Xelaev18045528m.g|	tbl3.L	TBL3	49.70	49.81	1.54	0.92	8.55E-19
I3	mi|Xelaev18029944m.g|	daw1.S	DAW1	48.99	37.51	1.98	0.93	1.48E-19
I3	mi|Xelaev18019976m.g|	g3bp1.S	G3BP1	48.12	49.10	12.83	0.95	7.57E-23
I3	mi|Xelaev18047504m.g|	rsl1d1.S	RSL1D1	48.11	48.85	15.85	0.94	2.97E-21
I3	mi|Xelaev18022848m.g|	katnb1.L	KATNB1	48.11	29.47	-7.15	0.84	7.55E-13
I3	mi|Xelaev18021116m.g|	twf1.S	TWF1	46.93	53.59	17.39	0.88	7.97E-15
I3	mi|Xelaev18047357m.g|	wdr12.S	WDR12	46.63	57.40	9.78	0.95	1.63E-22
I3	mi|Xelaev18007911m.g|	kremen1.L	KREMEN1	46.25	36.47	-10.66	0.84	6.26E-13
I3	mi|Xelaev18003341m.g|	Xelaev18003341m.g	HMCN2	45.81	23.97	-2.84	0.81	3.08E-11
I3	mi|Xelaev18032267m.g|	cthrc1.L	CTHRC1	45.27	30.12	-11.50	0.89	5.48E-16
I3	mi|Xelaev18003681m.g|	nop56.L	NOP56	44.93	22.90	6.31	0.92	2.47E-18
I3	mi|Xelaev18029408m.g|	syncrip.S	SYNCRIP	44.88	39.63	8.72	0.91	5.13E-18
I3	mi|Xelaev18017339m.g|	nhp2.L	NHP2	44.70	38.10	1.60	0.90	8.31E-17
I3	mi|Xelaev18012172m.g|	tuba3c.L	TUBA1C	44.48	39.07	8.95	0.90	1.94E-16
I3	mi|Xelaev18043830m.g|	psme3.L	PSME3	44.43	57.51	23.25	0.95	5.50E-23
I3	mi|Xelaev18010570m.g|	pus1.S	PUS1	44.22	53.87	21.03	0.93	5.63E-20
I3	mi|Xelaev18015006m.g|	tuba3c.S	TUBA3C	44.18	36.62	4.94	0.90	1.69E-16
I3	mi|Xelaev18036567m.g|	fbl.L	FBL	43.91	39.70	6.51	0.89	1.23E-15
I3	mi|Xelaev18026809m.g|	synj2.L	SYNJ2	43.59	30.13	-8.18	0.83	4.41E-12
I3	mi|Xelaev18036978m.g|	rrp12.S	RRP12	43.58	43.06	-5.31	0.88	5.79E-15
I3	mi|Xelaev18019137m.g|	ddx27.L	DDX27	43.57	41.73	1.63	0.89	1.01E-15
I3	mi|Xelaev18032072m.g|	mtfr1.L	MTFR1	43.15	47.44	4.91	0.88	1.59E-15
I3	mi|Xelaev18011548m.g|	mina.L	RIOX2	42.81	53.09	11.47	0.89	3.00E-16
I3	mi|Xelaev18021204m.g|	xpot.S	XPOT	42.62	61.45	23.13	0.94	7.55E-21
I3	mi|Xelaev18026185m.g|	diexf.L	UTP25	42.02	57.66	26.69	0.91	4.36E-18
I3	mi|Xelaev18038293m.g|	surf4.2.L	SURF4	41.99	41.07	8.79	0.93	8.88E-20

I3	mi|Xelaev18028344m.g|	loc100494572.L	CASR	41.92	42.08	-4.61	0.82	6.00E-12
I3	mi|Xelaev18006244m.g|	loc101734868.L	N/B	41.69	46.78	9.15	0.77	8.80E-10
I3	mi|Xelaev18023640m.g|	polr3h.L	POLR3H	41.53	47.86	20.35	0.86	7.42E-14
I3	mi|Xelaev18027871m.g|	ankmy1.L	ANKMY1	41.27	23.66	-3.92	0.82	6.70E-12
I3	mi|Xelaev18016836m.g|	g3bp1.L	G3BP1	40.98	46.44	14.35	0.95	1.65E-22
I3	mi|Xelaev18047242m.g|	dnajc10.S	DNAJC10	40.83	51.38	13.57	0.93	3.05E-20
I3	mi|Xelaev18018366m.g|	aen.L	AEN	40.67	49.98	22.19	0.88	3.56E-15
I3	mi|Xelaev18012537m.g|	hdac1.L	HDAC1	40.43	34.10	5.97	0.87	1.17E-14
I3	mi|Xelaev18009660m.g|	plin3.S	PLIN3	40.20	33.43	-10.89	0.84	1.21E-12
I3	mi|Xelaev18008374m.g|	brix1.L	BRIX1	40.17	29.77	0.21	0.87	2.60E-14
I3	mi|Xelaev18016116m.g|	kpna3.S	KPNA3	39.80	50.46	25.85	0.92	3.40E-18
I3	mi|Xelaev18036415m.g|	mrto4.L	MRTO4	39.74	44.10	5.67	0.87	2.18E-14
I3	mi|Xelaev18043784m.g|	utp18.L	UTP18	39.58	51.98	27.12	0.85	2.04E-13
I3	mi|Xelaev18032080m.g|	rrs1.L	RRS1	39.49	52.42	21.44	0.94	6.60E-22
I3	mi|Xelaev18023834m.g|	ifrd2.L	IFRD2	39.42	47.10	13.77	0.90	8.79E-17
I3	mi|Xelaev18013330m.g|	tuba1c.L	TUBA1B	39.40	34.67	-3.85	0.88	3.59E-15
I3	mi|Xelaev18045643m.g|	dctpp1.L	DCTPP1	38.97	47.65	6.92	0.91	1.98E-17
I3	mi|Xelaev18038451m.g|	hspa5.L	N/B	38.82	49.65	18.14	0.89	1.30E-15
I3	mi|Xelaev18030954m.g|	twistnb.L	TWISTNB	38.78	42.08	8.99	0.83	3.55E-12
I3	mi|Xelaev18003092m.g|	lpin3.L	LPIN3	38.71	28.03	-12.56	0.86	4.66E-14
I3	mi|Xelaev18011221m.g|	rrp1b.L	RRP1B	38.53	20.80	1.22	0.78	3.80E-10
I3	mi|Xelaev18047430m.g|	Xelaev18047430m.g	ACTB	38.44	40.88	0.18	0.84	1.38E-12
I3	mi|Xelaev18003076m.g|	kiaa1755.L	KIAA1755	38.40	36.87	-14.39	0.78	3.30E-10
I3	mi|Xelaev18024257m.g|	ehd1.S	EHD1	38.16	51.77	14.33	0.85	1.95E-13
I3	mi|Xelaev18008929m.g|	lyar.S	LYAR	38.10	48.58	20.11	0.91	1.68E-17
I3	mi|Xelaev18046924m.g|	nifk.S	NIFK	37.99	35.84	10.16	0.94	9.42E-21

I3	mi|Xelaev18027302m.g|	syncrip.L	SYNCRIP	37.62	39.47	8.43	0.86	1.32E-13
I3	mi|Xelaev18039568m.g|	ddx24.L	DDX24	37.57	48.50	21.74	0.91	3.13E-17
I3	mi|Xelaev18038075m.g|	fbl.S	FBL	37.57	37.64	0.47	0.83	3.68E-12
I3	mi|Xelaev18023992m.g|	ruvbl1.L	RUVBL1	37.47	39.59	6.57	0.88	2.60E-15
I3	mi|Xelaev18030562m.g|	loc101732654-like.L	N/B	36.52	22.28	-7.44	0.76	2.12E-09
I3	mi|Xelaev18032567m.g|	hgh1.L	HGH1	36.28	41.96	1.41	0.85	3.16E-13
I3	mi|Xelaev18033356m.g|	nsun2.S	NSUN2	36.26	49.12	22.64	0.93	2.54E-19
I3	mi|Xelaev18014473m.g|	rrp1b.S	RRP1B	36.25	38.06	2.54	0.89	7.24E-16
I3	mi|Xelaev18007760m.g|	dhx37.L	DHX37	35.87	46.76	13.95	0.90	1.13E-16
I3	mi|Xelaev18018333m.g|	c15orf26.L	CFAP161	35.74	29.51	4.33	0.89	8.28E-16
I3	mi|Xelaev18033931m.g|	dcaf13.S	DCAF13	35.31	34.39	-4.02	0.82	1.29E-11
I3	mi|Xelaev18044972m.g|	ccnyl1.L	CCNYL1	35.31	55.11	20.07	0.88	4.70E-15
I3	mi|Xelaev18020970m.g|	dnajc2.S	DNAJC2	35.26	40.10	12.43	0.88	3.91E-15
I3	mi|Xelaev18019057m.g|	farsa.L	FARSA	35.23	40.87	8.82	0.86	3.78E-14
I3	mi|Xelaev18031133m.g|	cdv3.L	CDV3	35.09	44.00	10.57	0.92	1.03E-18
I3	mi|Xelaev18029270m.g|	rpf2.S	RPF2	34.91	38.66	21.80	0.81	3.05E-11
I3	mi|Xelaev18007951m.g|	lmnb1.L	LMNB1	34.61	29.74	5.55	0.82	1.53E-11
I3	mi|Xelaev18015103m.g|	Xelaev18015103m.g	CTPS1	34.31	50.35	25.64	0.84	8.12E-13
I3	mi|Xelaev18003804m.g|	kiaa0020.S	PUM3	34.02	37.32	11.75	0.77	7.47E-10
I3	mi|Xelaev18020003m.g|	etf1.S	N/B	33.97	40.52	17.74	0.86	8.05E-14
I3	mi|Xelaev18047985m.g|	loc100492292.S	RNF112	33.81	14.07	-11.53	0.77	7.17E-10
I3	mi|Xelaev18013673m.g|	nek5.L	NEK5	33.74	37.55	8.53	0.80	1.01E-10
I3	mi|Xelaev18028809m.g|	trmt6.S	TRMT6	33.69	40.07	11.65	0.85	2.47E-13
I3	mi|Xelaev18025386m.g|	tmed5.S	TMED5	33.61	34.70	2.99	0.86	1.14E-13
I3	mi|Xelaev18039122m.g|	slc39a7.L	SLC39A7	33.26	42.92	11.03	0.89	2.92E-16
I3	mi|Xelaev18000775m.g|	farsa.S	FARSA	33.00	46.39	11.90	0.88	3.88E-15

I3	mi|Xelaev18018315m.g|	fes.L	FES	32.73	28.62	-11.42	0.81	2.96E-11
I3	mi|Xelaev18021523m.g|	hnrnph1.S	HNRNPH1	32.58	17.22	-0.16	0.82	9.40E-12
I3	mi|Xelaev18033141m.g|	stt3b.S	STT3B	32.41	37.30	9.10	0.85	1.96E-13
I3	mi|Xelaev18033771m.g|	rrs1.S	RRS1	31.92	42.13	8.44	0.85	3.89E-13
I3	mi|Xelaev18044371m.g|	pdia5.L	PDIA5	31.89	42.90	9.48	0.86	6.21E-14
I3	mi|Xelaev18015101m.g|	rcc1.S	RCC1	31.83	40.96	9.79	0.87	2.21E-14
I3	mi|Xelaev18006547m.g|	nans.L	NANS	31.31	33.71	4.63	0.87	2.79E-14
I3	mi|Xelaev18039103m.g|	mgat1.L	MGAT1	31.14	29.97	-0.09	0.84	1.14E-12
I3	mi|Xelaev18021200m.g|	hmga2.S	N/B	31.09	28.98	-7.65	0.85	4.61E-13
I3	mi|Xelaev18010734m.g|	lmnb1.S	LMNB1	30.89	30.22	3.57	0.84	1.11E-12
I3	mi|Xelaev18034964m.g|	bms1.L	BMS1	30.85	38.70	2.30	0.72	4.67E-08
I3	mi|Xelaev18023430m.g|	slc30a7.L	SLC30A7	30.77	35.00	0.43	0.77	8.94E-10
I3	mi|Xelaev18014885m.g|	bysl.S	BYSL	30.76	35.78	6.80	0.88	2.12E-15
I3	mi|Xelaev18031639m.g|	Xelaev18031639m.g	PAK1IP1	30.49	32.74	6.39	0.84	1.18E-12
I3	mi|Xelaev18009841m.g|	anp32c.S	N/B	30.45	22.11	7.51	0.81	1.91E-11
I3	mi|Xelaev18035020m.g|	ftsj3.L	FTSJ3	30.32	34.63	11.10	0.88	2.60E-15
I3	mi|Xelaev18025469m.g|	slc30a7.S	SLC30A7	30.30	45.59	4.58	0.91	7.07E-18
I3	mi|Xelaev18004698m.g|	gnl3.L	GNL3	29.78	21.72	-1.43	0.81	3.18E-11
I3	mi|Xelaev18017622m.g|	ifrd1.L	IFRD1	29.73	42.23	23.58	0.84	1.39E-12
I3	mi|Xelaev18007038m.g|	ngdn.L	N/B	29.65	45.77	12.41	0.89	7.01E-16
I3	mi|Xelaev18009518m.g|	sdad1.S	SDAD1	29.61	36.19	13.11	0.82	1.52E-11
I3	mi|Xelaev18000122m.g|	smarca5.L	SMARCA5	29.53	37.51	8.97	0.84	1.41E-12
I3	mi|Xelaev18006978m.g|	sass6.L	SASS6	29.15	45.38	10.07	0.84	5.79E-13
I3	mi|Xelaev18031899m.g|	esco1.L	ESCO1	29.05	35.28	-1.71	0.87	1.05E-14
I3	mi|Xelaev18043750m.g|	alyref.L	ALYREF	29.02	28.35	1.55	0.81	3.91E-11
I3	mi|Xelaev18029271m.g|	amd1.S	AMD1	28.98	31.95	13.41	0.84	1.14E-12

I3	mi|Xelaev18033191m.g|	cdv3.S	CDV3	28.95	42.00	17.72	0.92	5.72E-19
I3	mi|Xelaev18011589m.g|	cxadr.L	CXADR	28.62	13.29	-7.51	0.82	1.33E-11
I3	mi|Xelaev18003922m.g|	Xelaev18003922m.g	N/B	28.53	30.21	-3.31	0.82	1.38E-11
I3	mi|Xelaev18031358m.g|	pdcd6.L	N/B	28.32	29.49	-3.09	0.86	6.98E-14
I3	mi|Xelaev18009289m.g|	naa15.S	NAA15	28.25	40.23	10.41	0.79	2.67E-10
I3	mi|Xelaev18044492m.g|	abcb6.L	ABCB6	28.21	15.91	-9.68	0.81	2.66E-11
I3	mi|Xelaev18015210m.g|	hdac1.S	HDAC1	28.16	36.61	11.88	0.76	2.65E-09
I3	mi|Xelaev18014468m.g|	pwp2.S	PWP2	28.05	26.11	-13.47	0.69	2.06E-07
I3	mi|Xelaev18032934m.g|	loc100494509.S	CFAP69	27.98	25.31	-1.41	0.79	1.50E-10
I3	mi|Xelaev18017924m.g|	rbm28.L	RBM28	27.79	26.90	-4.40	0.77	1.01E-09
I3	mi|Xelaev18023257m.g|	arpc5.L	ARPC5	27.71	29.59	2.82	0.83	5.03E-12
I3	mi|Xelaev18038552m.g|	dkc1.L	DKC1	27.68	19.10	-4.49	0.88	4.35E-15
I3	mi|Xelaev18026873m.g|	cebpz.L	CEBPZ	27.51	37.71	17.11	0.85	2.60E-13
I3	mi|Xelaev18042357m.g|	ppp5c.S	PPP5C	27.33	34.53	16.77	0.88	2.71E-15
I3	mi|Xelaev18024120m.g|	loc100491288.L	N/B	27.24	32.10	0.16	0.83	4.88E-12
I3	mi|Xelaev18021719m.g|	cyp2r1.L	N/B	27.06	34.48	6.34	0.71	6.67E-08
I3	mi|Xelaev18005568m.g|	naa15.L	NAA15	26.97	36.78	7.66	0.80	4.71E-11
I3	mi|Xelaev18010244m.g|	prmt5.S	PRMT5	26.83	38.01	10.45	0.86	1.43E-13
I3	mi|Xelaev18017974m.g|	pstpip1.L	PSTPIP1	26.68	12.14	-9.40	0.76	1.95E-09
I3	mi|Xelaev18040010m.g|	prkd1.L	PRKD1	26.63	12.82	-8.33	0.79	1.50E-10
I3	mi|Xelaev18016233m.g|	zdhhc20.S	ZDHHC20	26.56	41.33	5.58	0.83	3.85E-12
I3	mi|Xelaev18034881m.g|	nolc1.L	NOLC1	26.56	32.35	1.71	0.73	2.14E-08
I3	mi|Xelaev18018167m.g|	aqp9.L	AQP9	26.20	32.24	-8.46	0.84	1.13E-12
I3	mi|Xelaev18025414m.g|	pkn2.S	PKN2	26.00	32.27	-3.47	0.80	5.29E-11
I3	mi|Xelaev18025315m.g|	cacybp.S	CACYBP	25.96	39.19	15.78	0.85	3.58E-13
I3	mi|Xelaev18000235m.g|	prss23.1	PRSS23	25.89	24.49	-11.70	0.74	8.88E-09

I3	mi|Xelaev18001521m.g|	ctf1.L	N/B	25.61	12.34	-10.92	0.83	2.47E-12
I3	mi|Xelaev18003211m.g|	akirin1.S	AKIRIN1	25.54	27.86	5.32	0.82	5.80E-12
I3	mi|Xelaev18020457m.g|	cers3.S	CERS2	25.46	23.04	-7.45	0.79	1.59E-10
I3	mi|Xelaev18012579m.g|	zmpste24.L	ZMPSTE24	25.42	38.63	18.32	0.84	6.02E-13
I3	mi|Xelaev18009263m.g|	smarca5.S	SMARCA5	25.10	28.26	4.02	0.73	1.86E-08
I3	mi|Xelaev18012632m.g|	cap1.L	CAP1	25.07	28.42	-2.36	0.68	3.57E-07
I3	mi|Xelaev18038118m.g|	abt1.L	ABT1	24.95	27.36	-1.82	0.80	5.23E-11
I3	mi|Xelaev18019507m.g|	plod3.L	PLOD3	24.93	13.46	-10.83	0.67	7.75E-07
I3	mi|Xelaev18028716m.g|	diexf.S	UTP25	24.90	34.82	5.82	0.73	1.49E-08
I3	mi|Xelaev18009715m.g|	ppp1r3b.S	PPP1R3B	24.75	49.66	12.13	0.85	4.46E-13
I3	mi|Xelaev18023869m.g|	wdr82.L	WDR82	24.72	43.72	24.81	0.82	9.10E-12
I3	mi|Xelaev18011070m.g|	txnl1.S	TXNL1	24.71	26.84	4.35	0.82	6.00E-12
I3	mi|Xelaev18047160m.g|	ssb.S	SSB	24.65	29.59	10.09	0.76	1.97E-09
I3	mi|Xelaev18032270m.g|	dcaf13.L	DCAF13	24.64	16.78	-10.35	0.63	4.92E-06
I3	mi|Xelaev18039592m.g|	vrk1.L	VRK1	24.30	38.63	8.66	0.84	9.87E-13
I3	mi|Xelaev18046501m.g|	psme3.S	PSME3	23.92	51.48	26.72	0.86	9.15E-14
I3	mi|Xelaev18035468m.g|	zpr1.L	ZPR1	23.78	44.54	25.81	0.76	2.06E-09
I3	mi|Xelaev18047503m.g|	thumpd1.S	THUMPD1	23.46	21.42	-1.47	0.74	7.16E-09
I3	mi|Xelaev18041512m.g|	rexo4.S	REXO4	23.40	24.56	-0.89	0.83	1.94E-12
I3	mi|Xelaev18022859m.g|	setd6.L	SETD6	23.09	21.21	0.54	0.79	2.04E-10
I3	mi|Xelaev18026273m.g|	degs1.L	DEGS1	23.03	31.53	-3.48	0.79	1.47E-10
I3	mi|Xelaev18025622m.g|	uck2.S	UCK2	22.97	45.48	15.88	0.83	2.46E-12
I3	mi|Xelaev18013463m.g|	tspan31.L	TSPAN31	22.94	38.44	9.64	0.90	6.88E-17
I3	mi|Xelaev18040653m.g|	nop9.L	NOP9	22.89	16.51	-12.31	0.74	1.14E-08
I3	mi|Xelaev18002566m.g|	susd3.S	SUSD3	22.70	23.02	-5.69	0.82	6.45E-12
I3	mi|Xelaev18018123m.g|	duox1.L	DUOX1	22.47	46.12	11.93	0.76	1.82E-09

I3	mi|Xelaev18018301m.g|	MGC85180.L	SLC39A1	22.32	37.18	-3.27	0.87	3.62E-14
I3	mi|Xelaev18017776m.g|	dnajc2.L	DNAJC2	22.12	38.49	13.76	0.90	1.48E-16
I3	mi|Xelaev18023839m.g|	hyal2.L	HYAL2	21.93	30.65	-1.82	0.69	1.86E-07
I3	mi|Xelaev18032103m.g|	orc5.L	ORC5	21.70	31.64	7.04	0.80	7.03E-11
I3	mi|Xelaev18016390m.g|	acer3.S	ACER3	21.64	34.85	6.49	0.81	1.87E-11
I3	mi|Xelaev18032519m.g|	bop1.L	BOP1	21.55	20.46	-9.57	0.74	9.80E-09
I3	mi|Xelaev18005850m.g|	nup54.L	NUP54	21.52	37.39	9.34	0.85	3.61E-13
I3	mi|Xelaev18046993m.g|	gja7.S	GJC1	21.37	11.44	-5.60	0.79	1.17E-10
I3	mi|Xelaev18025436m.g|	mettl13.S	EEF1AKNMT	21.30	16.86	-7.37	0.78	3.96E-10
I3	mi|Xelaev18038092m.g|	slc35a2.L	SLC35A2	21.22	40.08	9.75	0.78	3.78E-10
I3	mi|Xelaev18039668m.g|	c14orf166.L	RTRAF	21.02	26.93	13.66	0.81	2.82E-11
I3	mi|Xelaev18039079m.g|	rsph6a.L	RSPH4A	20.79	23.06	-5.89	0.84	1.13E-12
I3	mi|Xelaev18022608m.g|	dhx38.L	DHX38	20.56	13.84	-9.71	0.72	3.15E-08
I3	mi|Xelaev18043820m.g|	cant1.L	CANT1	19.96	25.01	3.03	0.76	2.10E-09
I3	mi|Xelaev18009899m.g|	elavl1.S	ELAVL1	19.78	24.71	4.33	0.68	3.24E-07
I3	mi|Xelaev18011526m.g|	usp16.L	USP16	19.66	28.83	-5.54	0.83	5.17E-12
I3	mi|Xelaev18025332m.g|	arpc5.S	ARPC5	19.45	29.49	0.10	0.80	1.02E-10
I3	mi|Xelaev18045176m.g|	rrn3.L	RRN3	19.36	26.72	3.55	0.80	7.67E-11
I3	mi|Xelaev18043313m.g|	eftud2.L	EFTUD2	19.27	15.76	-5.99	0.68	4.78E-07
I3	mi|Xelaev18045052m.g|	Xelaev18045052m.g	ACTB	19.20	26.25	-0.65	0.80	4.53E-11
I3	mi|Xelaev18039428m.g|	timm50.L	TIMM50	18.68	40.09	19.72	0.81	2.64E-11
I3	mi|Xelaev18023932m.g|	thumpd3.L	THUMPD3	18.61	23.19	-8.64	0.77	1.25E-09
I3	mi|Xelaev18036612m.g|	itprip.S	ITPRIP	18.51	19.84	-5.40	0.71	6.07E-08
I3	mi|Xelaev18015822m.g|	ccdc65.S	CCDC65	18.50	27.56	0.94	0.79	1.37E-10
I3	mi|Xelaev18040044m.g|	ankrd63.L	ANKRD63	18.43	31.78	3.61	0.62	6.10E-06
I3	mi|Xelaev18040547m.g|	gpatch4.L	GPATCH4	18.42	31.01	6.82	0.73	1.93E-08

I3	mi|Xelaev18033736m.g|	impad1.S	IMPAD1	18.15	41.93	26.07	0.78	6.06E-10
I3	mi|Xelaev18007714m.g|	ranbp1.L	RANBP1	18.12	21.36	3.59	0.88	7.32E-15
I3	mi|Xelaev18026134m.g|	mcfd2.L	N/B	17.95	31.98	4.48	0.64	2.92E-06
I3	mi|Xelaev18027510m.g|	ncbp2.L	NCBP2	17.84	22.83	7.62	0.69	2.14E-07
I3	mi|Xelaev18033057m.g|	mpp6.S	MPP6	17.72	39.98	18.16	0.73	1.38E-08
I3	mi|Xelaev18025907m.g|	rpn1.S	RPN1	17.51	10.56	-6.48	0.66	1.17E-06
I3	mi|Xelaev18027784m.g|	sgpp2.L	SGPP2	17.43	33.37	4.76	0.84	6.31E-13
I3	mi|Xelaev18005443m.g|	ppid.L	PPID	17.36	32.39	12.78	0.83	4.68E-12
I3	mi|Xelaev18029091m.g|	cebpz.S	CEBPZ	17.27	34.36	7.70	0.80	4.73E-11
I3	mi|Xelaev18045048m.g|	hspd1.L	HSPD1	17.17	33.42	20.89	0.78	6.29E-10
I3	mi|Xelaev18010803m.g|	riok2.S	RIOK2	16.66	36.80	18.30	0.87	3.63E-14
I3	mi|Xelaev18043324m.g|	hexim1.L	HEXIM1	16.56	34.55	18.94	0.83	4.90E-12
I3	mi|Xelaev18020212m.g|	mak16.S	MAK16	16.42	18.97	-3.48	0.67	6.64E-07
I3	mi|Xelaev18026559m.g|	acoxl.L	ACOXL	16.28	38.02	4.05	0.82	1.58E-11
I3	mi|Xelaev18030188m.g|	tram2.S	TRAM2	16.19	17.46	-10.78	0.82	1.54E-11
I3	mi|Xelaev18023514m.g|	ttc26.L	TTC26	15.74	27.65	9.87	0.79	1.21E-10
I3	mi|Xelaev18008420m.g|	txnl1.L	TXNL1	15.70	20.86	0.81	0.64	2.91E-06
I3	mi|Xelaev18004598m.g|	hnrnpa0.L	N/B	15.62	10.79	-2.46	0.67	5.54E-07
I3	mi|Xelaev18034647m.g|	c10orf2.L	TWNK	15.53	27.64	-4.97	0.66	1.22E-06
I3	mi|Xelaev18012630m.g|	Xelaev18012630m.g	EEF1A1	15.34	20.28	-9.97	0.76	1.92E-09
I3	mi|Xelaev18011581m.g|	hspa13.L	HSPA13	15.16	29.07	-3.72	0.81	1.69E-11
I3	mi|Xelaev18044198m.g|	wdr16.L	CFAP52	14.83	12.45	-10.70	0.74	1.08E-08
I3	mi|Xelaev18005238m.g|	tmem33.L	TMEM33	14.74	32.01	14.13	0.81	3.31E-11
I3	mi|Xelaev18008665m.g|	ino80b.S	INO80B	14.46	22.68	-3.09	0.71	6.31E-08
I3	mi|Xelaev18017980m.g|	sec11a.L	SEC11A	14.34	26.01	11.59	0.71	7.53E-08
I3	mi|Xelaev18032488m.g|	pycrl.L	PYCR3	13.62	24.31	-0.60	0.70	9.98E-08

I3	mi|Xelaev18015350m.g|	nxn.S	NXN	13.25	38.92	16.25	0.77	1.22E-09
I3	mi|Xelaev18036162m.g|	grwd1.L	N/B	13.08	18.20	-6.07	0.58	3.10E-05
I3	mi|Xelaev18045338m.g|	vwa3a.L	N/B	13.02	31.69	1.73	0.76	1.71E-09
I3	mi|Xelaev18018302m.g|	pdia3.L	PDIA3	12.98	10.95	-6.45	0.72	4.94E-08
I3	mi|Xelaev18026612m.g|	sec23b.L	SEC23B	12.84	24.43	-0.91	0.74	8.59E-09
I3	mi|Xelaev18046351m.g|	tnfrsf6b.S	TNFRSF6B	12.82	13.55	-10.83	0.80	7.83E-11
I3	mi|Xelaev18013725m.g|	hsph1.L	HSPH1	12.81	37.59	15.19	0.83	3.86E-12
I3	mi|Xelaev18039070m.g|	cd3eap-like.L	CD3EAP	12.50	18.92	-3.61	0.80	1.08E-10
I3	mi|Xelaev18011747m.g|	prkx.L	PRKX	11.69	11.12	-6.76	0.61	9.09E-06
I3	mi|Xelaev18019094m.g|	Xelaev18019094m.g	TRMT1	11.66	30.76	-2.23	0.71	6.53E-08
I3	mi|Xelaev18001385m.g|	Xelaev18001385m.g	ZNF568	11.47	24.82	1.66	0.78	4.29E-10
I3	mi|Xelaev18015944m.g|	sp5l.S	SP5	11.35	38.83	8.22	0.83	4.44E-12
I3	mi|Xelaev18012419m.g|	efhc2.L	EFHC2	11.33	30.90	4.06	0.70	1.34E-07
I3	mi|Xelaev18012297m.g|	ppil1.L	PPIL1	11.29	27.99	2.56	0.70	1.53E-07
I3	mi|Xelaev18039148m.g|	tap2.L	N/B	11.10	12.59	-8.18	0.71	8.81E-08
I3	mi|Xelaev18015713m.g|	commd6.S	COMMD6	10.61	31.29	13.89	0.72	4.52E-08
I3	mi|Xelaev18047097m.g|	prpf40a.S	PRPF40A	10.57	14.31	-4.72	0.65	1.57E-06
I3	mi|Xelaev18044753m.g|	prkra-like.L	PRKRA	10.36	10.68	6.04	0.60	1.93E-05
I3	mi|Xelaev18011191m.g|	cbs.L	CBS	10.29	22.62	-2.92	0.62	7.20E-06
D1	mi|Xelaev18035152m.g|	vstm4.L	VSTM4	50.44	53.90	6.47	0.93	3.30E-20
D1	mi|Xelaev18031536m.g|	cdh20.L	CDH20	47.77	49.74	-3.99	0.79	2.57E-10
D1	mi|Xelaev18039333m.g|	loc100145695.L	CYP2C8	47.64	41.48	-5.89	0.91	3.31E-17
D1	mi|Xelaev18020862m.g|	Xelaev18020862m.g	N/B	45.99	39.37	-8.65	0.87	2.62E-14
D1	mi|Xelaev18003091m.g|	emilin3.L	EMILIN3	45.97	38.72	-7.50	0.95	1.80E-23
D1	mi|Xelaev18028060m.g|	lpin1.L	LPIN1	42.36	45.30	15.97	0.92	3.14E-19
D1	mi|Xelaev18001146m.g|	slc25a42.S	SLC25A42	41.85	36.92	-7.27	0.85	3.33E-13

D1	mi|Xelaev18005800m.g|	pkd2.L	PKD2	40.13	39.26	-2.19	0.75	4.71E-09
D1	mi|Xelaev18027475m.g|	loc101732807.L	MASP1	39.58	46.72	2.34	0.86	8.55E-14
D1	mi|Xelaev18003184m.g|	loc100485662.1.L	FBXL14	37.77	45.76	13.38	0.85	5.40E-13
D1	mi|Xelaev18012527m.g|	eva1b.L	EVA1B	37.38	43.17	-4.14	0.82	1.47E-11
D1	mi|Xelaev18031781m.g|	fam69c.L	DIPK1C	37.25	26.59	-9.97	0.78	4.50E-10
D1	mi|Xelaev18012669m.g|	ulk2.L	ULK2	36.49	39.27	14.86	0.86	1.34E-13
D1	mi|Xelaev18024482m.g|	loc100497319-like.S	SPSB3	36.17	47.23	11.76	0.87	2.98E-14
D1	mi|Xelaev18012921m.g|	slc5a7.L	SLC5A7	34.80	41.95	3.25	0.74	7.83E-09
D1	mi|Xelaev18043291m.g|	hdac5.L	HDAC5	34.58	46.90	29.55	0.95	8.31E-23
D1	mi|Xelaev18015850m.g|	loc100493708.S	DPP10	34.36	38.87	4.68	0.92	4.70E-19
D1	mi|Xelaev18031139m.g|	acad11.L	ACAD11	33.77	47.34	13.50	0.92	1.32E-18
D1	mi|Xelaev18030095m.g|	lpin1.S	LPIN1	32.95	35.60	5.88	0.88	3.35E-15
D1	mi|Xelaev18032331m.g|	loc100493830-like.L	DEPTOR	32.93	29.81	-5.57	0.83	3.49E-12
D1	mi|Xelaev18004854m.g|	siglec1.L	SIGLEC1	32.37	26.82	1.21	0.78	3.57E-10
D1	mi|Xelaev18015496m.g|	slc5a7.S	SLC5A7	32.21	22.30	-9.20	0.70	1.15E-07
D1	mi|Xelaev18038985m.g|	wdr44.L	WDR44	32.00	50.60	17.06	0.84	5.58E-13
D1	mi|Xelaev18022382m.g|	nfatc3.L	NFATC3	31.94	45.93	10.06	0.78	3.50E-10
D1	mi|Xelaev18024044m.g|	loc100170549.L	LAMB1	31.76	25.51	-9.62	0.88	3.01E-15
D1	mi|Xelaev18021159m.g|	lgr5.S	LGR5	31.36	23.80	-12.19	0.89	5.26E-16
D1	mi|Xelaev18036835m.g|	fgfr2.S	FGFR2	31.30	19.35	-8.87	0.82	9.72E-12
D1	mi|Xelaev18005236m.g|	limch1.L	LIMCH1	30.42	14.94	-2.08	0.87	3.05E-14
D1	mi|Xelaev18010571m.g|	ulk1.S	ULK1	30.25	39.08	9.18	0.76	1.71E-09
D1	mi|Xelaev18026353m.g|	Xelaev18026353m.g	N/B	30.09	24.18	-10.60	0.85	3.23E-13
D1	mi|Xelaev18008865m.g|	fgfr3.S	N/B	29.20	21.77	3.21	0.81	1.97E-11
D1	mi|Xelaev18010277m.g|	sall2.S	SALL2	29.18	32.88	-5.19	0.74	1.00E-08
D1	mi|Xelaev18033872m.g|	tmem67.S	TMEM67	29.07	45.16	14.76	0.75	4.07E-09

D1	mi|Xelaev18023123m.g|	lrrc41.L	LRRC41	28.29	19.57	-10.41	0.68	4.18E-07
D1	mi|Xelaev18010073m.g|	uhrf2.S	UHRF2	28.24	42.64	10.16	0.83	3.40E-12
D1	mi|Xelaev18014735m.g|	loc100495198.S	MAOA	28.22	31.85	1.22	0.80	9.16E-11
D1	mi|Xelaev18043226m.g|	hoxb4.L	HOXB4	27.51	43.75	1.96	0.87	1.93E-14
D1	mi|Xelaev18039216m.g|	vwa7.L	VWA7	27.11	41.19	3.58	0.70	1.55E-07
D1	mi|Xelaev18047051m.g|	ctdsp1.S	CTDSP1	26.47	38.81	8.71	0.89	5.85E-16
D1	mi|Xelaev18033788m.g|	ncoa2.S	NCOA2	26.05	36.94	16.77	0.87	9.34E-15
D1	mi|Xelaev18006137m.g|	prdm8.L	PRDM8	25.51	45.89	10.63	0.80	1.02E-10
D1	mi|Xelaev18046711m.g|	itgb4.S	ITGB4	24.89	33.22	14.75	0.90	3.51E-17
D1	mi|Xelaev18029373m.g|	ankrd6.S	ANKRD6	24.77	28.62	-5.20	0.82	6.61E-12
D1	mi|Xelaev18016494m.g|	Xelaev18016494m.g	N/B	24.76	34.42	14.13	0.88	2.96E-15
D1	mi|Xelaev18025612m.g|	rxrg.S	RXRG	24.56	43.34	5.44	0.83	5.55E-12
D1	mi|Xelaev18022331m.g|	pc.L	PC	24.54	13.42	-7.66	0.76	1.96E-09
D1	mi|Xelaev18019333m.g|	chrne.L	CHRNE	24.38	21.36	-8.03	0.84	8.80E-13
D1	mi|Xelaev18022002m.g|	b4galnt4.L	B4GALNT4	23.46	34.62	14.81	0.92	1.30E-18
D1	mi|Xelaev18005591m.g|	larp1b.L	LARP1B	22.68	47.92	18.29	0.77	7.22E-10
D1	mi|Xelaev18013971m.g|	loc100498330.L	ARAP1	22.43	36.91	0.49	0.76	1.85E-09
D1	mi|Xelaev18046512m.g|	nbr1.S	NBR1	22.26	36.64	14.86	0.74	8.16E-09
D1	mi|Xelaev18045284m.g|	loc100488925.L	N/B	22.15	44.48	8.75	0.82	7.39E-12
D1	mi|Xelaev18008877m.g|	mxd4.S	MXD4	21.88	33.90	8.76	0.89	8.49E-16
D1	mi|Xelaev18006817m.g|	uhrf2.L	UHRF2	21.74	23.97	-8.69	0.80	7.30E-11
D1	mi|Xelaev18037307m.g|	angptl7.S	ANGPTL7	21.24	14.67	-13.01	0.74	1.29E-08
D1	mi|Xelaev18001815m.g|	Xelaev18001815m.g	SLC6A11	21.00	36.64	11.14	0.83	3.59E-12
D1	mi|Xelaev18029539m.g|	clcn2.S	CLCN2	20.98	44.10	14.89	0.65	2.18E-06
D1	mi|Xelaev18043645m.g|	fam110a.L	FAM110A	20.87	31.80	-2.52	0.67	5.94E-07
D1	mi|Xelaev18043101m.g|	hap1.L	TRAK1	20.80	53.80	29.31	0.88	2.07E-15

D1	mi|Xelaev18012794m.g|	gatsl1.L	CASTOR2	20.69	43.98	24.49	0.80	6.53E-11
D1	mi|Xelaev18042742m.g|	ankrd34a.S	ANKRD34A	19.74	11.29	-2.29	0.81	3.27E-11
D1	mi|Xelaev18005649m.g|	egf.L	EGF	19.61	21.10	-9.48	0.82	1.42E-11
D1	mi|Xelaev18044845m.g|	mreg.L	MREG	19.44	30.49	-2.85	0.65	1.51E-06
D1	mi|Xelaev18009221m.g|	fhdc1.S	FHDC1	19.32	16.95	-8.62	0.83	2.05E-12
D1	mi|Xelaev18025673m.g|	Xelaev18025673m.g	CYP2D6	19.30	32.00	0.75	0.71	5.10E-08
D1	mi|Xelaev18044615m.g|	ly75.L	LY75	19.28	22.91	1.49	0.71	7.13E-08
D1	mi|Xelaev18045690m.g|	Xelaev18045690m.g	ITGAM	19.10	15.41	-9.20	0.79	1.45E-10
D1	mi|Xelaev18017176m.g|	loc101731999.L	RNF14	18.84	16.64	-10.02	0.76	2.15E-09
D1	mi|Xelaev18042302m.g|	wdr44.S	WDR44	18.58	35.61	5.62	0.74	9.21E-09
D1	mi|Xelaev18035792m.g|	hes5_X1.L	HES5	18.56	20.91	2.26	0.75	6.57E-09
D1	mi|Xelaev18039523m.g|	foxn3.L	FOXN3	18.42	36.13	12.25	0.84	9.66E-13
D1	mi|Xelaev18003717m.g|	hhipl1.L	HHIPL1	18.26	20.72	-6.90	0.79	1.53E-10
D1	mi|Xelaev18025768m.g|	rbm5.S	RBM5	17.71	21.19	-9.30	0.80	4.57E-11
D1	mi|Xelaev18043445m.g|	fam65c.L	RIPOR3	17.58	48.99	21.78	0.77	6.91E-10
D1	mi|Xelaev18037638m.g|	Xelaev18037638m.g	CRYAB	17.54	41.19	16.46	0.79	2.37E-10
D1	mi|Xelaev18043292m.g|	g6pc3.1-like.L	G6PC3	17.36	25.52	-1.38	0.71	7.03E-08
D1	mi|Xelaev18038851m.g|	ids.L	IDS	17.10	25.73	-5.63	0.72	2.82E-08
D1	mi|Xelaev18023022m.g|	Xelaev18023022m.g	MYSM1	16.86	18.04	-8.22	0.76	2.64E-09
D1	mi|Xelaev18013854m.g|	sesn3.L	SESN3	16.27	27.87	1.18	0.79	1.28E-10
D1	mi|Xelaev18028057m.g|	greb1.L	GREB1	16.22	28.65	6.52	0.85	5.26E-13
D1	mi|Xelaev18041795m.g|	mapkap1.S	MAPKAP1	16.21	26.83	3.83	0.72	3.55E-08
D1	mi|Xelaev18045179m.g|	c16orf45.L	C16orf45	16.11	20.81	1.54	0.73	1.64E-08
D1	mi|Xelaev18001714m.g|	dlg5.L	DLG5	16.00	30.45	9.48	0.88	6.61E-15
D1	mi|Xelaev18024685m.g|	nfatc3.S	NFATC3	15.71	38.18	23.61	0.88	5.41E-15
D1	mi|Xelaev18015167m.g|	ptpru.S	PTPRU	15.69	25.78	5.98	0.67	5.31E-07

D1	mi|Xelaev18035985m.g|	lipe.L	LIPE	15.68	38.53	14.79	0.74	1.21E-08
D1	mi|Xelaev18016866m.g|	btbd11.L	BTBD11	15.61	16.71	-8.08	0.76	1.72E-09
D1	mi|Xelaev18042916m.g|	kcnn3.S	KCNN3	15.61	21.70	-3.96	0.75	3.46E-09
D1	mi|Xelaev18002658m.g|	pde4d-like.S	PDE4D	15.58	18.77	-4.04	0.61	9.55E-06
D1	mi|Xelaev18031238m.g|	ccdc129.L	ITPRID1	15.52	17.68	-6.96	0.81	2.26E-11
D1	mi|Xelaev18044896m.g|	map3k13.L	MAP3K13	15.41	25.60	3.34	0.71	5.17E-08
D1	mi|Xelaev18018341m.g|	arnt2.L	ARNT2	15.40	16.78	-12.07	0.71	6.77E-08
D1	mi|Xelaev18023628m.g|	loc101734319.L	COLGALT2	15.27	13.60	-10.74	0.77	8.68E-10
D1	mi|Xelaev18045845m.g|	loc100145779.S	BPI	15.24	15.74	-9.11	0.76	2.50E-09
D1	mi|Xelaev18015473m.g|	tmem47.S	TMEM47	15.08	21.44	-5.61	0.71	7.89E-08
D1	mi|Xelaev18005343m.g|	fam149a.L	FAM149A	14.74	20.98	1.16	0.84	6.02E-13
D1	mi|Xelaev18037110m.g|	pald1.S	PALD1	14.72	17.71	-7.49	0.77	9.14E-10
D1	mi|Xelaev18047579m.g|	unkl.S	UNK	14.61	33.95	9.84	0.78	2.87E-10
D1	mi|Xelaev18045873m.g|	hap1.S	TRAK1	14.53	38.27	16.05	0.86	5.12E-14
D1	mi|Xelaev18005528m.g|	Xetrov90000949m.L	N/B	14.45	14.69	-6.52	0.71	5.68E-08
D1	mi|Xelaev18009680m.g|	bmp2k.S	BMP2K	14.41	24.97	-3.07	0.85	2.89E-13
D1	mi|Xelaev18034763m.g|	Xelaev18034763m.g	HECTD2	14.13	21.44	3.53	0.70	9.62E-08
D1	mi|Xelaev18026889m.g|	lyst.L	LYST	13.43	25.70	4.13	0.84	1.17E-12
D1	mi|Xelaev18047744m.g|	tsc2.S	TSC2	12.93	39.06	23.19	0.86	1.04E-13
D1	mi|Xelaev18002572m.g|	Xelaev18002572m.g	SLC6A11	12.50	28.84	16.28	0.82	6.55E-12
D1	mi|Xelaev18021062m.g|	ankrd7.S	ANKRD26	12.08	19.25	-5.09	0.79	2.41E-10
D1	mi|Xelaev18005700m.g|	npnt.L	NPNT	11.94	26.82	-2.05	0.84	1.79E-12
D1	mi|Xelaev18024345m.g|	b4galnt4.S	B4GALNT4	11.89	27.18	12.72	0.89	4.24E-16
D1	mi|Xelaev18047537m.g|	gprc5b.S	GPRC5B	11.49	16.10	-4.90	0.81	4.49E-11
D1	mi|Xelaev18029111m.g|	sash1.S	SASH1	11.43	22.75	9.54	0.82	8.88E-12
D1	mi|Xelaev18039076m.g|	Xelaev18039076m.g	NOVA1	11.40	20.45	-7.19	0.76	2.81E-09

D1	mi|Xelaev18042375m.g|	nova2.S	NOVA2	11.38	28.84	-2.37	0.72	3.32E-08
D1	mi|Xelaev18036183m.g|	loc100497104.L	N/B	11.09	27.91	2.87	0.75	4.02E-09
D1	mi|Xelaev18019338m.g|	tnfsf12.L	N/B	10.93	22.42	-2.64	0.85	4.86E-13
D1	mi|Xelaev18030080m.g|	hpcal1.S	HPCAL1	10.85	27.59	0.09	0.73	1.64E-08
D1	mi|Xelaev18027278m.g|	ankrd6.L	ANKRD6	10.84	21.33	2.94	0.74	1.14E-08
D1	mi|Xelaev18033574m.g|	ldlrad4.S	LDLRAD4	10.49	26.89	7.53	0.68	4.44E-07
D1	mi|Xelaev18002649m.g|	map3k1.S	MAP3K1	10.24	26.65	15.79	0.81	2.44E-11
D1	mi|Xelaev18025291m.g|	lrp8.S	LRP8	10.10	15.73	1.41	0.74	1.27E-08
D2	mi|Xelaev18014320m.g|	Xelaev18014320m.g	N/B	58.46	52.93	13.76	0.83	2.91E-12
D2	mi|Xelaev18025883m.g|	klf15.S	KLF15	42.93	52.43	15.61	0.93	2.04E-19
D2	mi|Xelaev18035885m.g|	loc100135243.L	SBK1	42.00	32.22	-17.42	0.78	5.15E-10
D2	mi|Xelaev18017258m.g|	stk32a.L	STK32A	41.89	30.68	-6.52	0.91	1.29E-17
D2	mi|Xelaev18023970m.g|	klf15.L	KLF15	38.87	44.37	6.72	0.90	3.47E-17
D2	mi|Xelaev18038962m.g|	lpar4.L	LPAR4	38.65	40.51	-0.63	0.84	1.28E-12
D2	mi|Xelaev18008611m.g|	loc101734599.L	B3GALT5	37.72	42.13	4.74	0.88	5.49E-15
D2	mi|Xelaev18005863m.g|	ccng2.L	CCNG2	37.71	42.35	4.32	0.89	1.37E-15
D2	mi|Xelaev18021817m.g|	syt13-like.L	SYT13	37.07	38.45	-10.06	0.79	1.66E-10
D2	mi|Xelaev18039165m.g|	ppt2.L	N/B	36.37	44.57	7.66	0.93	2.70E-19
D2	mi|Xelaev18031231m.g|	dclk3.L	DCLK3	35.66	35.98	-8.65	0.87	1.73E-14
D2	mi|Xelaev18017285m.g|	kiaa1191.L	N/B	34.92	25.24	-4.43	0.79	1.29E-10
D2	mi|Xelaev18017356m.g|	tcp11l2.L	TCP11L2	34.40	58.56	29.37	0.94	7.27E-21
D2	mi|Xelaev18028283m.g|	loc100496183.L	N/B	34.29	17.83	-0.73	0.80	5.63E-11
D2	mi|Xelaev18006161m.g|	lpl.L	LPL	34.18	24.59	-3.70	0.82	7.12E-12
D2	mi|Xelaev18021324m.g|	tcp11l2.S	TCP11L2	33.16	53.79	26.35	0.89	9.19E-16
D2	mi|Xelaev18006882m.g|	klf9.L	KLF9	31.79	41.80	5.49	0.89	9.92E-16
D2	mi|Xelaev18007986m.g|	cdo1.L	CDO1	31.08	35.65	3.78	0.89	2.76E-16

D2	mi|Xelaev18032084m.g|	sgk3.L	SGK3	30.88	54.59	24.09	0.77	8.33E-10
D2	mi|Xelaev18020562m.g|	ppip5k1.S	PPIP5K1	30.57	28.30	-5.16	0.84	1.30E-12
D2	mi|Xelaev18013473m.g|	ctdsp2.L	CTDSP2	30.06	33.16	6.95	0.75	6.26E-09
D2	mi|Xelaev18035807m.g|	uts2.L	N/B	29.91	43.23	7.11	0.80	1.15E-10
D2	mi|Xelaev18005208m.g|	tlr1.L	TLR1	29.43	40.29	-0.36	0.80	4.61E-11
D2	mi|Xelaev18027202m.g|	sesn1.L	SESN1	29.07	41.28	7.63	0.96	2.51E-25
D2	mi|Xelaev18037640m.g|	Xelaev18037640m.g	CRYAB	27.99	53.59	23.01	0.89	1.49E-15
D2	mi|Xelaev18010535m.g|	ypel1.S	YPEL1	27.87	32.49	2.28	0.92	5.05E-19
D2	mi|Xelaev18024163m.g|	ltbp3.S	LTBP3	27.68	53.33	24.56	0.87	1.72E-14
D2	mi|Xelaev18029910m.g|	arhgef4.S	ARHGEF4	26.97	37.90	-4.68	0.79	2.10E-10
D2	mi|Xelaev18023971m.g|	aldh1l1.L	ALDH1L1	26.74	45.13	14.46	0.88	6.41E-15
D2	mi|Xelaev18003101m.g|	sgk2.L	SGK2	26.61	27.03	-3.25	0.77	6.67E-10
D2	mi|Xelaev18016854m.g|	rfx4.L	RFX4	26.35	21.11	-21.18	0.76	1.71E-09
D2	mi|Xelaev18037836m.g|	ppp1r15a.S	PPP1R15B	26.10	23.29	-5.29	0.77	1.10E-09
D2	mi|Xelaev18041740m.g|	pfkfb1.S	PFKFB1	25.65	42.57	19.00	0.85	1.90E-13
D2	mi|Xelaev18038933m.g|	fam46d.L	TENT5C	25.63	23.48	-15.84	0.66	9.78E-07
D2	mi|Xelaev18010714m.g|	slc35e4-like.S	SLC35E4	25.57	23.20	-10.52	0.76	1.64E-09
D2	mi|Xelaev18035650m.g|	mxra8.L	MXRA8	24.82	40.41	14.93	0.83	3.60E-12
D2	mi|Xelaev18024353m.g|	th.S	TH	24.16	29.22	-4.40	0.82	8.68E-12
D2	mi|Xelaev18013761m.g|	rasl11a.L	RASL11A	23.77	24.09	-11.47	0.70	1.60E-07
D2	mi|Xelaev18009835m.g|	loc100494506-like.S	SLC1A6	23.68	21.87	-3.71	0.83	1.98E-12
D2	mi|Xelaev18027866m.g|	klhl24.L	KLHL24	23.47	34.95	8.49	0.87	1.45E-14
D2	mi|Xelaev18029287m.g|	sesn1.S	SESN1	23.25	37.32	8.20	0.91	3.91E-18
D2	mi|Xelaev18015962m.g|	ctdsp2.S	CTDSP2	23.08	21.97	2.90	0.62	6.03E-06
D2	mi|Xelaev18033707m.g|	pcmtd1.S	PCMTD1	22.86	20.03	-7.43	0.77	1.39E-09
D2	mi|Xelaev18044326m.g|	tmem37.L	TMEM37	22.84	40.93	12.45	0.88	6.85E-15

D2	mi|Xelaev18033774m.g|	mybl1.S	MYBL1	21.59	15.43	-5.02	0.83	4.92E-12
D2	mi|Xelaev18010102m.g|	Xelaev18010102m.g	N/B	21.22	20.37	-3.88	0.84	6.03E-13
D2	mi|Xelaev18045133m.g|	emp2.L	EMP2	21.06	40.17	9.12	0.86	1.07E-13
D2	mi|Xelaev18014677m.g|	loc101732226-like.S	ZNF19	20.70	30.49	2.27	0.85	1.59E-13
D2	mi|Xelaev18039614m.g|	loc100189574.L	HEBP2	20.49	17.52	-5.53	0.79	1.78E-10
D2	mi|Xelaev18023755m.g|	MGC75753.L	BTG1	19.51	33.65	6.69	0.73	1.36E-08
D2	mi|Xelaev18027839m.g|	scly.L	SCLY	19.41	19.69	-4.61	0.91	1.29E-17
D2	mi|Xelaev18047120m.g|	dapl1.S	DAPL1	19.09	40.33	21.74	0.87	8.85E-15
D2	mi|Xelaev18008301m.g|	fst.L	FST	19.09	29.13	-4.24	0.73	2.29E-08
D2	mi|Xelaev18026306m.g|	adck3.L	COQ8A	18.94	35.92	8.22	0.69	2.66E-07
D2	mi|Xelaev18026621m.g|	rin2.L	RIN2	18.49	26.49	11.33	0.84	8.22E-13
D2	mi|Xelaev18047408m.g|	slc40a1.S	SLC40A1	18.01	29.54	3.86	0.76	2.63E-09
D2	mi|Xelaev18033912m.g|	tp53inp1.S	TP53INP1	17.08	15.45	5.84	0.77	7.09E-10
D2	mi|Xelaev18011484m.g|	dnajc28.L	DNAJC28	16.95	37.06	14.21	0.81	1.77E-11
D2	mi|Xelaev18036115m.g|	ppp1r15a.L	PPP1R15B	16.47	22.47	-1.91	0.86	1.47E-13
D2	mi|Xelaev18016207m.g|	polr1d.1.S	POLR1D	15.91	23.32	1.37	0.79	1.28E-10
D2	mi|Xelaev18035719m.g|	ms4a1.L	MS4A4A	15.90	32.74	12.81	0.79	1.47E-10
D2	mi|Xelaev18035306m.g|	hepacam.L	HEPACAM	15.83	19.58	-8.00	0.65	2.29E-06
D2	mi|Xelaev18026599m.g|	mertk.L	MERTK	15.78	26.19	3.66	0.76	2.55E-09
D2	mi|Xelaev18037559m.g|	loc100488469.S	N/B	15.63	18.86	-4.08	0.76	1.72E-09
D2	mi|Xelaev18001911m.g|	map1lc3b.S	N/B	15.47	21.25	-0.70	0.83	2.41E-12
D2	mi|Xelaev18009707m.g|	ppp2cb.S	PPP2CB	15.14	11.26	-3.08	0.77	9.93E-10
D2	mi|Xelaev18001838m.g|	Xelaev18001838m.g	YIPF4	15.00	31.28	8.20	0.72	2.70E-08
D2	mi|Xelaev18046608m.g|	map1lc3a.S	MAP1LC3A	14.69	19.00	-0.54	0.85	4.80E-13
D2	mi|Xelaev18044604m.g|	dapl1.L	DAPL1	14.46	28.04	12.99	0.75	6.29E-09
D2	mi|Xelaev18039473m.g|	irf2bpl.L	IRF2BPL	14.08	27.21	-2.36	0.85	1.78E-13

D2	mi|Xelaev18021216m.g|	metap2.S	METAP2	13.83	18.31	-3.10	0.85	2.52E-13
D2	mi|Xelaev18023731m.g|	mpst.L	MPST	13.64	19.45	-3.23	0.85	1.98E-13
D2	mi|Xelaev18030739m.g|	mtpap.L	MTPAP	13.05	23.16	-1.03	0.78	6.54E-10
D2	mi|Xelaev18024188m.g|	npas4.S	NPAS4	13.00	25.05	-14.53	0.72	2.88E-08
D2	mi|Xelaev18004048m.g|	zbtb8a.2.S	ZBTB8A	12.63	37.43	15.39	0.63	3.98E-06
D2	mi|Xelaev18043594m.g|	pcmtd2.L	PCMTD2	12.59	38.26	12.65	0.65	1.76E-06
D2	mi|Xelaev18025682m.g|	tef.S	TEF	12.48	18.88	2.06	0.86	8.09E-14
D2	mi|Xelaev18044077m.g|	trim65.L	TRIM65	12.22	22.16	-0.03	0.83	2.96E-12
D2	mi|Xelaev18024175m.g|	loc100124841.S	N/B	12.17	10.75	-8.68	0.69	2.12E-07
D2	mi|Xelaev18022697m.g|	ca5b.L	CA5B	12.07	33.33	8.88	0.70	1.11E-07
D2	mi|Xelaev18003216m.g|	Xelaev18003216m.g	PNRC2	11.61	13.20	-5.49	0.85	2.86E-13
D2	mi|Xelaev18013866m.g|	slc5a7-like.2.L	SLC5A7	11.21	22.31	-3.92	0.65	1.78E-06
D2	mi|Xelaev18032082m.g|	mybl1.L	MYBL1	11.06	17.34	1.23	0.78	3.14E-10
D2	mi|Xelaev18039014m.g|	mospd1.L	MOSPD1	10.67	32.44	4.88	0.83	2.57E-12
D2	mi|Xelaev18041933m.g|	loc100489885.S	CRADD	10.45	13.17	-5.57	0.73	1.49E-08
D2	mi|Xelaev18036227m.g|	loc100135223.L	N/B	10.21	13.16	-1.49	0.86	6.29E-14
D2	mi|Xelaev18023747m.g|	pfkfb4.L	PFKFB4	10.17	31.73	7.22	0.72	2.73E-08
D2	mi|Xelaev18037799m.g|	tbc1d17.S	TBC1D17	10.05	25.62	7.64	0.73	1.46E-08
L1	mi|Xelaev18011155m.g|	aqp3.S	AQP3	112.09	82.27	-3.25	0.95	3.08E-23
L1	mi|Xelaev18006478m.g|	crlf1.L	CRLF1	105.29	68.95	-8.57	0.96	2.61E-25
L1	mi|Xelaev18042775m.g|	nes.S	NES	70.64	58.47	-0.51	0.87	1.24E-14
L1	mi|Xelaev18023861m.g|	parp3.L	PARP3	68.12	52.88	-9.80	0.94	9.21E-21
L1	mi|Xelaev18025789m.g|	manf.S	MANF	66.57	60.65	18.63	0.98	1.91E-30
L1	mi|Xelaev18028048m.g|	pdia6.L	PDIA6	65.07	61.01	20.99	0.96	6.87E-26
L1	mi|Xelaev18038032m.g|	nphs1.S	NPHS1	62.93	36.60	-5.03	0.91	9.46E-18
L1	mi|Xelaev18019973m.g|	ckap4.S	CKAP4	62.09	51.94	-4.55	0.96	1.82E-24

L1	mi|Xelaev18007684m.g|	sdf2l1.L	SDF2L1	60.72	49.57	11.57	0.94	5.88E-22
L1	mi|Xelaev18030084m.g|	pdia6.S	PDIA6	59.65	51.55	19.36	0.90	7.77E-17
L1	mi|Xelaev18010529m.g|	sdf2l1.S	SDF2L1	58.47	54.81	14.48	0.92	6.00E-19
L1	mi|Xelaev18012803m.g|	mybbp1a.L	MYBBP1A	56.98	51.79	10.57	0.95	4.16E-22
L1	mi|Xelaev18013139m.g|	dnajc3.L	DNAJC3	56.48	49.30	3.33	0.94	7.36E-21
L1	mi|Xelaev18009214m.g|	sfrp2.S	SFRP2	56.32	51.54	-4.74	0.95	4.87E-23
L1	mi|Xelaev18031149m.g|	ctnnb1.L	CTNNB1	55.74	45.56	-6.28	0.95	4.90E-23
L1	mi|Xelaev18010558m.g|	pgam5.S	PGAM5	55.57	53.91	8.59	0.96	6.89E-25
L1	mi|Xelaev18018667m.g|	stc1.L	STC1	55.44	49.01	4.11	0.91	2.99E-17
L1	mi|Xelaev18007461m.g|	Xelaev18007461m.g	CORO1C	55.43	54.02	12.62	0.94	5.38E-22
L1	mi|Xelaev18026393m.g|	tcte1.L	TCTE1	54.16	60.34	15.97	0.96	5.78E-25
L1	mi|Xelaev18021342m.g|	nhp2.S	NHP2	54.16	49.02	14.82	0.95	2.45E-22
L1	mi|Xelaev18034521m.g|	htra1.L	HTRA1	53.01	39.37	-10.82	0.94	3.93E-21
L1	mi|Xelaev18042298m.g|	pls3.S	PLS3	52.44	54.25	2.71	0.94	4.73E-21
L1	mi|Xelaev18012073m.g|	rcc1.L	RCC1	50.17	42.75	4.21	0.90	1.53E-16
L1	mi|Xelaev18025680m.g|	Xelaev18025680m.g	RANGAP1	49.69	52.06	13.09	0.95	4.15E-23
L1	mi|Xelaev18018036m.g|	stra6.L	STRA6	49.18	24.15	-6.53	0.88	7.53E-15
L1	mi|Xelaev18034668m.g|	npm3.L	NPM3	48.85	53.06	15.19	0.96	1.77E-24
L1	mi|Xelaev18017515m.g|	hmga2.L	N/B	48.61	36.90	-9.61	0.91	1.03E-17
L1	mi|Xelaev18000740m.g|	tmed1.S	TMED1	48.43	36.15	-11.08	0.85	1.51E-13
L1	mi|Xelaev18020849m.g|	lmf2.S	LMF2	47.93	48.18	5.76	0.90	9.04E-17
L1	mi|Xelaev18039709m.g|	rcor1.L	N/B	47.28	32.33	-9.35	0.85	5.07E-13
L1	mi|Xelaev18017384m.g|	hsp90b1.L	HSP90B1	47.25	51.72	15.43	0.94	8.75E-21
L1	mi|Xelaev18033843m.g|	e2f5.S	E2F5	46.72	48.24	-0.31	0.94	9.05E-22
L1	mi|Xelaev18007339m.g|	Xelaev18007339m.g	N/B	46.49	45.43	13.12	0.90	3.78E-17
L1	mi|Xelaev18033048m.g|	fam126a.S	FAM126A	46.41	36.54	-6.10	0.87	1.23E-14

L1	mi|Xelaev18028912m.g|	heatr1.S	HEATR1	45.98	45.77	5.91	0.92	3.79E-19
L1	mi|Xelaev18006258m.g|	kiss1r.L	KISS1R	45.90	24.32	-7.27	0.89	3.28E-16
L1	mi|Xelaev18042399m.g|	mgat1.S	MGAT1	45.56	38.68	-7.73	0.92	1.97E-18
L1	mi|Xelaev18021202m.g|	llph.S	N/B	45.05	47.13	22.41	0.95	2.22E-23
L1	mi|Xelaev18027677m.g|	slc33a1.L	SLC33A1	44.85	51.05	11.01	0.96	2.57E-24
L1	mi|Xelaev18017669m.g|	iqub.L	IQUB	44.55	58.54	8.85	0.93	5.07E-20
L1	mi|Xelaev18044935m.g|	wdr12.L	WDR12	44.48	56.83	25.05	0.96	2.79E-24
L1	mi|Xelaev18012647m.g|	vmp1.L	VMP1	44.35	47.62	4.14	0.88	8.12E-15
L1	mi|Xelaev18023679m.g|	Xelaev18023679m.g	RANGAP1	44.13	51.51	14.33	0.96	9.34E-25
L1	mi|Xelaev18022820m.g|	sf3b3.L	SF3B3	44.10	34.83	5.37	0.92	1.04E-18
L1	mi|Xelaev18041802m.g|	hspa5.S	N/B	44.06	55.84	22.73	0.92	1.36E-18
L1	mi|Xelaev18001788m.g|	wnt5a.L	WNT5A	43.83	28.65	-7.27	0.84	5.53E-13
L1	mi|Xelaev18032541m.g|	Xelaev18032541m.g	N/B	42.93	35.79	-10.79	0.88	2.70E-15
L1	mi|Xelaev18047772m.g|	rnf151.S	RNF151	42.86	33.34	-9.04	0.87	2.27E-14
L1	mi|Xelaev18024199m.g|	Xelaev18024199m.g	N/B	42.64	53.40	20.04	0.91	1.98E-17
L1	mi|Xelaev18044358m.g|	umps.L	UMPS	42.59	46.44	12.57	0.91	4.93E-18
L1	mi|Xelaev18006951m.g|	fbp1.L	N/B	42.58	41.24	-5.59	0.90	2.50E-16
L1	mi|Xelaev18005306m.g|	ppat.L	PPAT	42.43	32.23	0.24	0.91	1.17E-17
L1	mi|Xelaev18003918m.g|	Xelaev18003918m.g	PDCD11	42.00	44.38	10.52	0.93	3.54E-20
L1	mi|Xelaev18043858m.g|	loc100494738.L	MMP2	41.33	34.61	-8.12	0.84	1.63E-12
L1	mi|Xelaev18010951m.g|	skiv2l2.S	MTREX	40.77	38.75	8.82	0.91	3.93E-18
L1	mi|Xelaev18011482m.g|	gart.L	GART	40.76	42.69	3.46	0.91	2.10E-17
L1	mi|Xelaev18015282m.g|	vmp1.S	VMP1	40.76	46.26	15.04	0.79	2.72E-10
L1	mi|Xelaev18015590m.g|	f7.S	F7	40.45	19.67	-7.29	0.89	7.24E-16
L1	mi|Xelaev18018815m.g|	ppapdc1b.L	PLPP5	40.05	43.70	-0.27	0.91	7.05E-18
L1	mi|Xelaev18022647m.g|	usp10.L	USP10	39.68	48.50	22.34	0.90	5.40E-17

L1	mi|Xelaev18005008m.g|	polr1a.L	POLR1A	39.45	46.64	1.90	0.90	2.36E-16
L1	mi|Xelaev18017001m.g|	loc100490380-like.L	WDR55	38.57	43.21	20.74	0.85	1.82E-13
L1	mi|Xelaev18040743m.g|	Xelaev18040743m.g	RNF213	38.44	34.94	-9.89	0.88	3.83E-15
L1	mi|Xelaev18026206m.g|	tmem206.L	TMEM206	37.94	38.89	-0.59	0.86	1.07E-13
L1	mi|Xelaev18034106m.g|	bop1.S	BOP1	37.47	25.89	-9.06	0.84	1.02E-12
L1	mi|Xelaev18031726m.g|	mrs2.L	MRS2	37.38	66.02	37.69	0.87	2.69E-14
L1	mi|Xelaev18044297m.g|	utp6.L	UTP6	37.25	33.68	9.70	0.92	3.29E-18
L1	mi|Xelaev18022627m.g|	nip7.L	NIP7	37.15	32.54	10.29	0.91	2.05E-17
L1	mi|Xelaev18044030m.g|	tmem104.L	TMEM104	36.92	38.86	1.19	0.91	9.09E-18
L1	mi|Xelaev18000735m.g|	qtrt1.S	QTRT1	36.81	44.73	7.80	0.92	4.29E-19
L1	mi|Xelaev18010878m.g|	utp15.S	UTP15	36.51	51.15	23.19	0.94	7.86E-21
L1	mi|Xelaev18023800m.g|	usp4.L	USP4	36.04	42.30	2.50	0.93	1.14E-19
L1	mi|Xelaev18017332m.g|	tmed9.L	TMED9	35.87	37.20	12.97	0.90	7.00E-17
L1	mi|Xelaev18026823m.g|	urb2.L	URB2	35.80	49.08	20.94	0.92	7.82E-19
L1	mi|Xelaev18001128m.g|	ddx49.S	DDX49	35.48	39.50	5.24	0.89	1.42E-15
L1	mi|Xelaev18018035m.g|	ccdc33.L	CCDC33	35.23	15.84	-6.34	0.88	2.18E-15
L1	mi|Xelaev18029541m.g|	eif4g3-like.S	EIF4G1	35.10	45.09	7.40	0.91	1.40E-17
L1	mi|Xelaev18022695m.g|	klhdc4.L	KLHDC4	35.08	48.45	18.05	0.74	8.52E-09
L1	mi|Xelaev18038561m.g|	pih1d3.L	PIH1D3	34.62	25.35	-3.14	0.90	9.26E-17
L1	mi|Xelaev18011503m.g|	urb1.L	URB1	34.52	44.16	6.81	0.90	4.52E-17
L1	mi|Xelaev18040474m.g|	loc100488012.L	N/B	33.85	46.93	8.67	0.87	1.37E-14
L1	mi|Xelaev18015057m.g|	ubxn11.S	UBXN11	33.78	47.35	12.19	0.91	4.37E-18
L1	mi|Xelaev18035426m.g|	hyou1.L	HYOU1	33.68	48.92	18.34	0.95	4.75E-22
L1	mi|Xelaev18024088m.g|	shq1.L	SHQ1	32.98	46.04	23.62	0.86	6.93E-14
L1	mi|Xelaev18030974m.g|	nfe2l3.L	NFE2L3	32.92	39.42	3.64	0.88	3.22E-15
L1	mi|Xelaev18016185m.g|	uspl1.S	USPL1	32.66	39.19	1.44	0.77	9.00E-10

L1	mi|Xelaev18039997m.g|	aqr.L	AQR	32.54	38.99	3.85	0.82	7.43E-12
L1	mi|Xelaev18014992m.g|	loc100487263.S	HEATR6	32.53	34.39	-1.73	0.85	2.18E-13
L1	mi|Xelaev18028044m.g|	nol10.L	NOL10	32.36	19.63	-0.86	0.83	3.02E-12
L1	mi|Xelaev18030030m.g|	hs6st1.S	HS6ST1	32.31	33.35	0.65	0.92	2.90E-18
L1	mi|Xelaev18018031m.g|	cd276.L	CD276	32.16	44.29	3.94	0.83	3.38E-12
L1	mi|Xelaev18027786m.g|	farsb.L	FARSB	32.05	47.95	9.76	0.93	2.14E-19
L1	mi|Xelaev18004404m.g|	hyou1.S	HYOU1	31.98	50.83	12.75	0.90	5.23E-17
L1	mi|Xelaev18014052m.g|	serpinh1.L	SERPINH1	31.92	43.69	15.53	0.75	4.84E-09
L1	mi|Xelaev18027122m.g|	nus1.L	NUS1P1	31.65	44.56	18.71	0.89	7.53E-16
L1	mi|Xelaev18036397m.g|	rcc2.L	RCC2	31.52	23.47	-5.71	0.70	1.28E-07
L1	mi|Xelaev18022048m.g|	loc100486192.L	IFITM2	31.47	11.89	-8.69	0.88	2.68E-15
L1	mi|Xelaev18019046m.g|	tmed1.L	TMED1	31.43	37.92	1.35	0.85	1.55E-13
L1	mi|Xelaev18002185m.g|	Xelaev18002185m.g	N/B	31.29	36.65	-3.54	0.88	4.45E-15
L1	mi|Xelaev18026168m.g|	eml4.L	EML4	31.18	20.91	-5.37	0.82	1.07E-11
L1	mi|Xelaev18033061m.g|	nfe2l3.S	NFE2L3	31.18	26.82	-6.81	0.78	2.98E-10
L1	mi|Xelaev18032107m.g|	crispld1.L	CRISPLD1	30.78	44.26	2.85	0.84	1.07E-12
L1	mi|Xelaev18006612m.g|	loc100491278-like.L	EXOSC3	30.77	28.81	7.76	0.88	6.11E-15
L1	mi|Xelaev18028225m.g|	tram2.L	TRAM2	30.66	43.51	-2.88	0.90	1.81E-16
L1	mi|Xelaev18002087m.g|	Xelaev18002087m.g	ANKFN1	30.61	18.00	-9.23	0.81	3.91E-11
L1	mi|Xelaev18022218m.g|	stip1.L	STIP1	30.48	37.57	18.10	0.86	4.17E-14
L1	mi|Xelaev18012860m.g|	nup88.L	NUP88	30.46	24.60	-2.91	0.77	8.29E-10
L1	mi|Xelaev18025804m.g|	rrp9.S	RRP9	29.77	32.86	3.89	0.89	1.30E-15
L1	mi|Xelaev18005211m.g|	fam114a1.L	FAM114A1	29.71	38.56	18.48	0.81	4.47E-11
L1	mi|Xelaev18036988m.g|	loc100493999-like.S	N/B	29.70	29.39	-7.54	0.77	7.96E-10
L1	mi|Xelaev18039639m.g|	Xelaev18039639m.g	ARF6	29.64	35.59	-3.40	0.90	5.60E-17
L1	mi|Xelaev18030229m.g|	efr3b.S	EFR3B	29.54	18.11	-8.89	0.83	4.77E-12

L1	mi|Xelaev18012815m.g|	Xelaev18012815m.g	DERL2	29.42	27.21	2.08	0.88	3.26E-15
L1	mi|Xelaev18021668m.g|	Xelaev18021668m.g	GTF2H1	29.23	32.12	3.78	0.89	1.38E-15
L1	mi|Xelaev18040932m.g|	thbs3.L	THBS3	29.17	27.14	-6.87	0.82	6.90E-12
L1	mi|Xelaev18023860m.g|	rrp9.L	RRP9	29.00	38.07	9.86	0.90	5.42E-17
L1	mi|Xelaev18016536m.g|	sfrpx.S	SFRP2	28.97	39.04	0.02	0.74	1.09E-08
L1	mi|Xelaev18044872m.g|	nme9.L	NME8	27.56	35.28	8.37	0.80	7.48E-11
L1	mi|Xelaev18017767m.g|	pus7.L	PUS7	27.30	40.31	14.06	0.89	9.88E-16
L1	mi|Xelaev18045335m.g|	polr3e-like.L	N/B	27.10	33.84	-1.33	0.89	5.24E-16
L1	mi|Xelaev18013188m.g|	cln5.L	CLN5	27.04	42.77	4.37	0.93	2.50E-19
L1	mi|Xelaev18022997m.g|	alg6.L	ALG6	26.96	43.26	8.85	0.90	4.78E-17
L1	mi|Xelaev18029500m.g|	lrrc1.S	LRRC1	26.63	41.57	14.33	0.75	4.12E-09
L1	mi|Xelaev18036953m.g|	dpcd.S	DPCD	26.26	23.88	7.22	0.77	1.44E-09
L1	mi|Xelaev18041467m.g|	zmynd19.S	ZMYND19	26.01	27.75	0.66	0.86	7.12E-14
L1	mi|Xelaev18028844m.g|	xrn2.S	XRN2	25.95	30.42	0.57	0.82	1.40E-11
L1	mi|Xelaev18007923m.g|	loc100493526-like.L	N/B	25.79	21.67	-8.27	0.76	3.15E-09
L1	mi|Xelaev18040345m.g|	cers2.L	CERS2	25.66	31.65	9.76	0.72	2.66E-08
L1	mi|Xelaev18007453m.g|	ficd.L	FICD	25.60	21.06	-7.12	0.87	1.53E-14
L1	mi|Xelaev18009796m.g|	ptbp1.S	PTBP1	25.33	42.75	2.48	0.88	4.31E-15
L1	mi|Xelaev18045870m.g|	jup.S	JUP	25.25	18.78	-7.07	0.63	3.85E-06
L1	mi|Xelaev18026710m.g|	Xelaev18026710m.g	N/B	25.24	25.85	-8.83	0.89	3.80E-16
L1	mi|Xelaev18036160m.g|	Xelaev18036160m.g	GPRC5C	25.14	25.72	-2.82	0.90	8.98E-17
L1	mi|Xelaev18021103m.g|	dnajb9.S	DNAJB9	25.08	46.13	17.53	0.94	5.33E-21
L1	mi|Xelaev18047053m.g|	pnkd.S	PNKD	24.79	25.10	-13.07	0.85	3.34E-13
L1	mi|Xelaev18041513m.g|	surf4.2.S	SURF4	24.70	33.05	2.11	0.85	4.44E-13
L1	mi|Xelaev18002761m.g|	mylip.S	MYLIP	24.68	43.63	10.88	0.82	6.60E-12
L1	mi|Xelaev18031586m.g|	tubb2b.L	TUBB2B	24.59	31.84	2.41	0.74	9.68E-09

L1	mi|Xelaev18026477m.g|	plek.L	PLEK	24.46	16.71	-8.39	0.68	4.15E-07
L1	mi|Xelaev18027425m.g|	eif4g3-like.L	EIF4G1	24.43	39.76	7.72	0.87	8.45E-15
L1	mi|Xelaev18026372m.g|	Xelaev18026372m.g	KCNK5	24.43	29.40	3.37	0.58	3.21E-05
L1	mi|Xelaev18024857m.g|	piezo1.S	PIEZO1	24.15	32.85	-10.28	0.90	4.32E-17
L1	mi|Xelaev18041210m.g|	tmx1.S	TMX1	24.12	35.52	4.12	0.67	5.24E-07
L1	mi|Xelaev18022050m.g|	loc101733140.L	IFITM2	24.09	13.09	-9.97	0.80	4.65E-11
L1	mi|Xelaev18013028m.g|	chst7.L	CHST7	23.83	34.19	1.81	0.90	1.57E-16
L1	mi|Xelaev18001389m.g|	Xelaev18001389m.g	ZNF79	23.73	12.64	-6.10	0.70	9.86E-08
L1	mi|Xelaev18000401m.g|	Xelaev18000401m.g	N/B	23.31	31.01	-1.78	0.84	1.60E-12
L1	mi|Xelaev18045147m.g|	txndc11.L	TXNDC11	23.05	25.98	1.55	0.86	5.16E-14
L1	mi|Xelaev18005736m.g|	Xelaev18005736m.g	N/B	22.75	29.30	-3.56	0.80	7.10E-11
L1	mi|Xelaev18021012m.g|	celsr2.S	CELSR1	22.74	16.25	-5.70	0.78	6.54E-10
L1	mi|Xelaev18031292m.g|	cdk13.L	CDK13	22.57	28.48	-4.25	0.81	2.61E-11
L1	mi|Xelaev18031895m.g|	thoc1.L	THOC1	22.32	29.95	2.84	0.79	2.69E-10
L1	mi|Xelaev18028836m.g|	smek2.S	PPP4R3B	22.25	35.19	8.47	0.84	1.48E-12
L1	mi|Xelaev18028984m.g|	loc100488558-like.S	FAM98A	22.22	44.44	16.71	0.80	7.82E-11
L1	mi|Xelaev18029645m.g|	b3gnt5.S	B3GNT5	22.13	38.15	5.24	0.81	2.68E-11
L1	mi|Xelaev18041214m.g|	ptger2.S	PTGER2	21.75	23.47	-10.39	0.80	6.54E-11
L1	mi|Xelaev18023913m.g|	edem1.L	EDEM1	21.74	32.42	4.44	0.76	2.29E-09
L1	mi|Xelaev18007775m.g|	gtf2h3.L	GTF2H3	21.61	30.07	6.31	0.73	2.16E-08
L1	mi|Xelaev18010551m.g|	loc100135405.S	N/B	21.49	16.04	-10.22	0.84	1.41E-12
L1	mi|Xelaev18034423m.g|	chd4.L	CHD4	21.43	20.71	-1.93	0.72	3.18E-08
L1	mi|Xelaev18039542m.g|	loc100485426-like.L	PPP4R3A	21.38	37.06	5.85	0.81	1.81E-11
L1	mi|Xelaev18041432m.g|	lmo4.2.S	LMO4	21.30	16.72	-7.60	0.73	1.74E-08
L1	mi|Xelaev18034723m.g|	entpd7.L	ENTPD7	21.25	45.55	15.60	0.90	1.88E-16
L1	mi|Xelaev18039504m.g|	sel1l.L	SEL1L	21.04	15.19	-7.28	0.68	4.16E-07

L1	mi|Xelaev18003334m.g|	dolpp1.L	DOLPP1	20.94	49.04	24.30	0.75	6.47E-09
L1	mi|Xelaev18018517m.g|	mob1a.L	MOB1A	20.93	23.48	-6.64	0.71	6.28E-08
L1	mi|Xelaev18029123m.g|	lats1.S	LATS1	20.88	35.94	6.11	0.88	2.04E-15
L1	mi|Xelaev18026376m.g|	naglu.L	NAGLU	20.83	30.89	-0.46	0.62	8.19E-06
L1	mi|Xelaev18015644m.g|	zic5.S	ZIC5	20.72	24.33	-6.67	0.82	6.64E-12
L1	mi|Xelaev18028835m.g|	pnpt1.S	PNPT1	20.71	40.67	10.05	0.90	4.35E-17
L1	mi|Xelaev18040741m.g|	Xelaev18040741m.g	RNF213	20.67	28.40	-10.77	0.83	2.75E-12
L1	mi|Xelaev18009347m.g|	gar1.S	GAR1	20.60	34.11	8.64	0.87	2.18E-14
L1	mi|Xelaev18013117m.g|	zic5.L	ZIC5	20.41	23.42	-7.42	0.75	4.60E-09
L1	mi|Xelaev18026540m.g|	smek2.L	PPP4R3B	20.40	30.21	7.66	0.78	3.91E-10
L1	mi|Xelaev18035192m.g|	exosc10.L	EXOSC10	20.24	22.29	0.61	0.88	4.45E-15
L1	mi|Xelaev18047570m.g|	c7orf50.S	C7orf50	20.19	11.49	-11.06	0.56	7.67E-05
L1	mi|Xelaev18039464m.g|	flvcr2.L	FLVCR2	19.99	21.11	-10.44	0.84	7.51E-13
L1	mi|Xelaev18033858m.g|	osgin2.S	OSGIN2	19.88	28.97	7.22	0.83	3.10E-12
L1	mi|Xelaev18041545m.g|	c9orf116.S	C9orf116	19.77	18.43	-1.78	0.65	2.21E-06
L1	mi|Xelaev18000838m.g|	loc100492804.S	N/B	19.52	17.61	-6.26	0.62	6.13E-06
L1	mi|Xelaev18013990m.g|	hpx.L	HPX	19.42	28.21	-2.45	0.83	2.00E-12
L1	mi|Xelaev18004340m.g|	Xetrov90029205m.L	N/B	19.40	25.14	-8.77	0.73	1.43E-08
L1	mi|Xelaev18045563m.g|	shmt1.L	SHMT1	19.39	28.84	-2.67	0.64	2.48E-06
L1	mi|Xelaev18024901m.g|	dhx38.S	DHX38	19.26	27.48	7.23	0.65	1.62E-06
L1	mi|Xelaev18014525m.g|	baz1b.S	BAZ1B	19.08	16.59	-5.09	0.78	5.66E-10
L1	mi|Xelaev18018415m.g|	chsy1.L	CHSY1	18.95	16.82	-10.10	0.75	6.21E-09
L1	mi|Xelaev18012140m.g|	slc30a2.L	SLC30A2	18.91	24.78	-2.19	0.64	2.35E-06
L1	mi|Xelaev18017211m.g|	lars.L	LARS	18.67	38.67	11.97	0.79	1.62E-10
L1	mi|Xelaev18018122m.g|	duoxa1.L	DUOXA1	18.62	44.90	21.18	0.68	4.48E-07
L1	mi|Xelaev18006698m.g|	lpar1.L	LPAR1	18.62	40.62	14.56	0.82	1.28E-11

L1	mi|Xelaev18041464m.g|	nsmf.S	NSMF	18.53	29.39	-2.90	0.67	8.10E-07
L1	mi|Xelaev18024126m.g|	slc25a26.L	SLC25A26	18.42	12.46	-10.63	0.86	4.14E-14
L1	mi|Xelaev18016352m.g|	eed.S	N/B	18.39	27.53	4.35	0.71	9.17E-08
L1	mi|Xelaev18034050m.g|	chrac1.S	CHRAC1	18.34	21.89	-2.33	0.71	6.18E-08
L1	mi|Xelaev18044524m.g|	slc11a1.L	SLC11A1	18.30	15.86	-7.43	0.71	5.38E-08
L1	mi|Xelaev18017543m.g|	slc35e3.L	SLC35E3	18.18	42.23	13.65	0.74	7.39E-09
L1	mi|Xelaev18014150m.g|	sdf2.S	SDF2	18.11	33.44	3.67	0.90	5.42E-17
L1	mi|Xelaev18008122m.g|	rad17.L	RAD17	18.05	19.90	-0.78	0.67	7.07E-07
L1	mi|Xelaev18031880m.g|	myl12b.L	MYL12A	17.81	13.15	-9.08	0.87	2.49E-14
L1	mi|Xelaev18027676m.g|	gmps.L	GMPS	17.69	18.87	-0.32	0.87	1.34E-14
L1	mi|Xelaev18014492m.g|	Xetrov90025900m.S	N/B	17.65	28.74	-2.27	0.75	5.01E-09
L1	mi|Xelaev18008068m.g|	slf1.L	SLF1	17.64	33.68	19.72	0.75	4.43E-09
L1	mi|Xelaev18039147m.g|	tap1.L	TAP1	17.59	26.97	0.57	0.80	6.41E-11
L1	mi|Xelaev18008447m.g|	me2.L	ME2	17.49	25.56	-3.72	0.79	2.25E-10
L1	mi|Xelaev18023179m.g|	prpf38a.L	N/B	17.44	11.86	-0.53	0.67	5.98E-07
L1	mi|Xelaev18016912m.g|	gemin5.L	GEMIN5	17.26	22.09	-6.28	0.74	7.57E-09
L1	mi|Xelaev18022217m.g|	fermt3.L	FERMT3	17.24	18.61	-9.45	0.64	3.06E-06
L1	mi|Xelaev18008050m.g|	riok2.L	RIOK2	17.22	24.36	9.55	0.66	9.77E-07
L1	mi|Xelaev18038866m.g|	cxorf57.L	RADX	17.21	13.70	-9.95	0.83	2.96E-12
L1	mi|Xelaev18027350m.g|	lrrc1.L	LRRC1	17.17	30.19	-3.18	0.81	3.32E-11
L1	mi|Xelaev18010143m.g|	tle1.S	TLE1	17.09	19.41	-7.42	0.68	4.52E-07
L1	mi|Xelaev18015989m.g|	Xelaev18015989m.g	GTF2H4	17.02	24.52	5.14	0.74	1.13E-08
L1	mi|Xelaev18029775m.g|	slc33a1.S	SLC33A1	16.86	31.28	6.64	0.76	2.14E-09
L1	mi|Xelaev18030513m.g|	Xelaev18030513m.g	N/B	16.81	11.59	-5.25	0.69	2.27E-07
L1	mi|Xelaev18017110m.g|	nme5.L	NME5	16.72	30.19	12.31	0.86	3.89E-14
L1	mi|Xelaev18020009m.g|	larp1.S	LARP1	16.71	11.46	-6.64	0.66	1.16E-06

L1	mi|Xelaev18047232m.g|	cwc22.S	CWC22	16.33	20.64	0.07	0.75	5.99E-09
L1	mi|Xelaev18031366m.g|	ropn1l.L	ROPN1L	15.77	18.57	-9.01	0.78	6.41E-10
L1	mi|Xelaev18021840m.g|	fjx1.L	FJX1	15.71	14.75	-9.35	0.64	3.03E-06
L1	mi|Xelaev18041376m.g|	fcf1.S	FCF1	15.64	32.65	7.13	0.68	3.62E-07
L1	mi|Xelaev18038244m.g|	Xelaev18038244m.g	FCN1	15.63	12.21	-11.96	0.66	1.08E-06
L1	mi|Xelaev18024056m.g|	loc100158544.L	TTLL3	15.59	13.29	-11.60	0.71	5.46E-08
L1	mi|Xelaev18046938m.g|	eaf2.S	EAF2	15.53	12.47	-6.91	0.75	6.36E-09
L1	mi|Xelaev18008384m.g|	tars.L	TARS	15.26	27.37	1.57	0.72	2.98E-08
L1	mi|Xelaev18041863m.g|	pdcl.S	PDCL	15.17	26.25	12.68	0.76	2.61E-09
L1	mi|Xelaev18034885m.g|	sec24d-like.2.L	SEC24C	14.93	18.49	-7.35	0.82	1.57E-11
L1	mi|Xelaev18009192m.g|	ppid.S	PPID	14.88	28.19	11.49	0.66	9.37E-07
L1	mi|Xelaev18033985m.g|	derl1.S	DERL1	14.78	12.91	-10.27	0.66	9.66E-07
L1	mi|Xelaev18018637m.g|	ddx56.L	DDX56	14.77	25.99	-1.23	0.74	9.34E-09
L1	mi|Xelaev18017314m.g|	znf346.L	ZNF346	14.55	35.30	9.36	0.86	5.65E-14
L1	mi|Xelaev18032226m.g|	pop1-like.L	POP1	14.47	11.49	-11.25	0.60	1.74E-05
L1	mi|Xelaev18011076m.g|	loc100485773.S	N/B	14.42	13.99	-9.68	0.70	1.02E-07
L1	mi|Xelaev18025038m.g|	znhit6.S	ZNHIT6	14.27	19.05	-3.61	0.83	5.00E-12
L1	mi|Xelaev18021021m.g|	creld2.S	CRELD2	14.27	26.33	-3.35	0.66	1.02E-06
L1	mi|Xelaev18014718m.g|	loc100489793.S	SLC19A2	14.21	27.33	4.27	0.79	1.25E-10
L1	mi|Xelaev18012002m.g|	nras.L	NRAS	14.00	42.05	16.91	0.74	1.26E-08
L1	mi|Xelaev18031308m.g|	alg2.L	ALG2	13.96	10.53	-7.04	0.73	1.69E-08
L1	mi|Xelaev18000497m.g|	wdr3.S	WDR3	13.93	25.13	7.99	0.82	8.10E-12
L1	mi|Xelaev18033482m.g|	nup153.S	NUP153	13.91	11.15	-4.90	0.74	1.30E-08
L1	mi|Xelaev18024984m.g|	katnb1.S	KATNB1	13.84	22.38	-4.73	0.81	2.45E-11
L1	mi|Xelaev18045065m.g|	ptcd1.L	PTCD1	13.78	29.11	12.31	0.73	2.31E-08
L1	mi|Xelaev18028379m.g|	ccdc25.L	CCDC25	13.64	20.90	5.20	0.61	1.26E-05

L1	mi|Xelaev18008242m.g|	dimt1.L	DIMT1	13.55	18.08	4.77	0.69	2.64E-07
L1	mi|Xelaev18011462m.g|	setd4.L	SETD4	13.54	21.17	-3.87	0.62	8.40E-06
L1	mi|Xelaev18012614m.g|	zbtb8a.1.L	ZBTB8A	13.48	35.38	8.61	0.76	2.66E-09
L1	mi|Xelaev18003356m.g|	Xelaev18003356m.g	HMCN2	13.00	26.11	1.31	0.78	4.39E-10
L1	mi|Xelaev18024915m.g|	utp4.S	UTP4	12.67	13.88	-9.17	0.68	4.58E-07
L1	mi|Xelaev18014935m.g|	pigw.S	PIGW	12.63	31.12	8.86	0.88	3.87E-15
L1	mi|Xelaev18008627m.g|	loc100490559.L	ZNF329	12.57	32.11	9.93	0.86	8.68E-14
L1	mi|Xelaev18047317m.g|	adprh.S	ADPRH	12.50	25.99	-2.79	0.78	3.76E-10
L1	mi|Xelaev18036059m.g|	bcl2l12.L	BCL2L12	12.36	12.53	-10.24	0.54	1.35E-04
L1	mi|Xelaev18038479m.g|	lhx6.L	LHX6	12.31	37.86	12.85	0.85	2.16E-13
L1	mi|Xelaev18014199m.g|	tbl2.S	TBL2	12.31	12.04	-6.08	0.75	5.45E-09
L1	mi|Xelaev18044061m.g|	tsen54.L	TSEN54	12.29	27.91	2.61	0.81	2.07E-11
L1	mi|Xelaev18012036m.g|	akirin1.L	AKIRIN1	12.21	29.10	8.06	0.86	6.37E-14
L1	mi|Xelaev18038719m.g|	c1galt1c1.L	C1GALT1C1	12.14	38.89	12.03	0.78	3.74E-10
L1	mi|Xelaev18031220m.g|	c7orf72.L	SPATA48	12.13	14.00	-7.49	0.70	1.43E-07
L1	mi|Xelaev18028790m.g|	chac2.S	CHAC2	12.12	27.45	0.66	0.75	3.54E-09
L1	mi|Xelaev18022850m.g|	ccdc135.L	DRC7	12.11	22.15	-4.05	0.84	9.65E-13
L1	mi|Xelaev18030828m.g|	slc25a40.L	SLC25A40	12.09	37.87	9.62	0.81	4.45E-11
L1	mi|Xelaev18044423m.g|	cxcr2.L	CXCR1	12.00	14.36	-7.84	0.67	7.83E-07
L1	mi|Xelaev18015219m.g|	kpna6.S	KPNA6	11.97	29.17	-0.82	0.76	2.65E-09
L1	mi|Xelaev18046900m.g|	loc100492872.S	MARCO	11.88	13.09	-9.37	0.56	6.55E-05
L1	mi|Xelaev18038219m.g|	dph7.L	DPH7	11.76	10.95	-10.79	0.71	6.76E-08
L1	mi|Xelaev18012070m.g|	Xelaev18012070m.g	CTPS1	11.74	16.80	-7.59	0.84	8.03E-13
L1	mi|Xelaev18018051m.g|	snupn.L	SNUPN	11.69	16.98	-5.20	0.66	8.55E-07
L1	mi|Xelaev18009070m.g|	tmem165.S	TMEM165	11.68	30.76	3.92	0.86	7.50E-14
L1	mi|Xelaev18029785m.g|	p2ry1.S	P2RY1	11.65	33.24	9.50	0.69	2.05E-07

L1	mi|Xelaev18038250m.g|	Xelaev18038250m.g	FCN1	11.43	14.18	-5.54	0.62	7.16E-06
L1	mi|Xelaev18047585m.g|	dnaaf5.S	DNAAF5	11.43	28.74	-2.72	0.73	2.20E-08
L1	mi|Xelaev18046995m.g|	loc100495096.S	MYO10	11.24	18.06	-5.04	0.64	2.64E-06
L1	mi|Xelaev18024803m.g|	dnaaf1.S	DNAAF1	11.23	16.04	-9.89	0.66	1.06E-06
L1	mi|Xelaev18013968m.g|	Xelaev18013968m.g	N/B	11.13	32.44	4.32	0.76	2.10E-09
L1	mi|Xelaev18032016m.g|	lypla1.L	N/B	11.07	20.73	-0.84	0.79	1.36E-10
L1	mi|Xelaev18026468m.g|	snrpb2.L	SNRPB2	10.94	11.83	-4.60	0.68	4.55E-07
L1	mi|Xelaev18010783m.g|	man2a1.S	MAN2A1	10.89	19.77	-10.84	0.82	1.21E-11
L1	mi|Xelaev18007728m.g|	noc4l.L	NOC4L	10.71	11.65	-5.53	0.70	1.45E-07
L1	mi|Xelaev18043168m.g|	stard3.L	STARD3	10.65	19.14	-2.75	0.78	4.80E-10
L1	mi|Xelaev18023258m.g|	ncf2.L	NCF2	10.61	11.14	-9.89	0.68	4.46E-07
L1	mi|Xelaev18021942m.g|	agbl2.L	AGBL2	10.55	19.10	-5.35	0.85	2.91E-13
L1	mi|Xelaev18041480m.g|	Xelaev18041480m.g	FCN1	10.47	19.43	-0.68	0.76	1.77E-09
L1	mi|Xelaev18038245m.g|	Xelaev18038245m.g	FCN1	10.37	17.42	-5.44	0.59	2.79E-05
L1	mi|Xelaev18046162m.g|	tmem189.S	N/B	10.27	29.76	14.25	0.63	5.57E-06
L1	mi|Xelaev18015491m.g|	ranbp2.S	RANBP2	10.16	22.58	-0.34	0.74	6.99E-09
L1	mi|Xelaev18011918m.g|	cluh.L	CLUH	10.08	29.40	8.35	0.76	1.53E-09
L2	mi|Xelaev18040537m.g|	nes.L	NES	52.65	40.40	-1.94	0.89	9.13E-16
L2	mi|Xelaev18007012m.g|	ipo4.L	IPO4	51.95	53.80	19.51	0.97	5.19E-28
L2	mi|Xelaev18016936m.g|	ctnna1.L	CTNNA1	47.21	59.19	22.46	0.95	2.85E-22
L2	mi|Xelaev18026661m.g|	mtr.L	MTR	47.00	59.25	22.16	0.88	6.17E-15
L2	mi|Xelaev18016659m.g|	wnt7b.L	WNT7B	46.48	36.47	-7.07	0.95	4.35E-22
L2	mi|Xelaev18027711m.g|	serp2.L	SERP1	46.15	38.90	11.83	0.92	1.06E-18
L2	mi|Xelaev18046040m.g|	fzd2.S	FZD2	45.83	38.42	-8.83	0.85	5.31E-13
L2	mi|Xelaev18044267m.g|	kpna2.L	KPNA2	45.79	23.11	-3.89	0.91	9.96E-18
L2	mi|Xelaev18023833m.g|	sema3b.L	SEMA3B	45.00	50.18	2.14	0.93	2.67E-20

L2	mi|Xelaev18017940m.g|	lmf2.L	LMF2	44.03	53.38	15.98	0.96	1.19E-24
L2	mi|Xelaev18031847m.g|	apcdd1.L	APCDD1	43.74	51.69	22.87	0.95	3.75E-23
L2	mi|Xelaev18022815m.g|	rarg-like.2.L	EXOSC6	42.82	47.09	17.00	0.97	2.61E-26
L2	mi|Xelaev18032592m.g|	abcf2.S	ABCF2	42.39	45.17	6.46	0.96	2.59E-25
L2	mi|Xelaev18010467m.g|	mlec.S	MLEC	42.09	38.66	5.30	0.90	8.95E-17
L2	mi|Xelaev18003611m.g|	tram1.L	TRAM1	41.81	54.31	13.13	0.94	3.95E-21
L2	mi|Xelaev18008012m.g|	wdr36.L	WDR36	40.56	46.10	17.42	0.96	1.68E-25
L2	mi|Xelaev18043274m.g|	fzd2.L	FZD2	38.57	35.80	-7.58	0.90	8.00E-17
L2	mi|Xelaev18043969m.g|	rbl1.L	RBL1	37.81	32.83	-1.67	0.85	2.51E-13
L2	mi|Xelaev18017912m.g|	flnc.L	FLNC	36.63	19.21	-5.90	0.90	4.53E-17
L2	mi|Xelaev18021625m.g|	dnmt1.S	DNMT1	36.55	42.59	10.31	0.95	3.13E-22
L2	mi|Xelaev18006078m.g|	mfsd12.L	MFSD12	36.45	31.78	-12.79	0.95	3.41E-22
L2	mi|Xelaev18012403m.g|	ddx3x.L	DDX3X	36.36	55.67	28.56	0.91	4.04E-18
L2	mi|Xelaev18037693m.g|	cd37.S	CD37	36.25	36.83	-7.01	0.92	3.15E-19
L2	mi|Xelaev18026709m.g|	mthfd1l.L	MTHFD1L	36.24	36.94	-1.81	0.91	1.38E-17
L2	mi|Xelaev18028539m.g|	slc35b2.S	SLC35B2	35.96	27.81	-8.92	0.97	1.78E-26
L2	mi|Xelaev18037434m.g|	timm8b.S	TIMM8B	35.88	42.85	21.40	0.92	3.46E-19
L2	mi|Xelaev18010452m.g|	pxn.S	PXN	34.45	38.39	-8.56	0.92	4.73E-19
L2	mi|Xelaev18039126m.g|	wdr46.L	WDR46	34.22	36.73	13.23	0.91	4.34E-18
L2	mi|Xelaev18021623m.g|	p2ry11.S	P2RY1	32.79	21.95	1.44	0.90	1.95E-16
L2	mi|Xelaev18045954m.g|	kpnb1.S	KPNB1	32.37	33.89	-1.77	0.98	1.09E-29
L2	mi|Xelaev18039478m.g|	pomt2.L	N/B	31.25	43.67	15.17	0.92	5.01E-19
L2	mi|Xelaev18005668m.g|	arsj.L	ARSJ	30.58	11.04	-7.05	0.75	6.11E-09
L2	mi|Xelaev18008328m.g|	fbxo4.L	FBXO4	29.39	36.40	-1.93	0.88	3.42E-15
L2	mi|Xelaev18018975m.g|	atp13a1.L	ATP13A1	28.94	29.89	-5.37	0.95	7.68E-23
L2	mi|Xelaev18041183m.g|	rcor1.S	N/B	27.93	14.46	-9.43	0.85	2.67E-13

L2	mi|Xelaev18026274m.g|	nvl.L	NVL	27.63	30.55	-0.06	0.80	9.17E-11
L2	mi|Xelaev18023843m.g|	zmynd10.L	ZMYND10	27.14	21.92	-7.62	0.87	2.15E-14
L2	mi|Xelaev18000054m.g|	prpf19.L	PRPF19	26.84	43.05	14.25	0.97	3.48E-27
L2	mi|Xelaev18011090m.g|	mex3c.S	MEX3C	26.41	34.44	4.17	0.88	1.84E-15
L2	mi|Xelaev18046582m.g|	src.S	SRC	26.39	43.91	3.94	0.91	2.12E-17
L2	mi|Xelaev18043196m.g|	kpnb1.L	KPNB1	26.11	28.63	0.97	0.95	7.42E-23
L2	mi|Xelaev18012547m.g|	kpna6.L	KPNA6	25.91	20.15	-4.52	0.90	3.53E-17
L2	mi|Xelaev18013148m.g|	sox21.L	SOX21	25.24	17.51	-2.82	0.72	3.93E-08
L2	mi|Xelaev18027990m.g|	uggt1-like.L	UGGT1	24.52	19.41	-4.72	0.88	7.37E-15
L2	mi|Xelaev18014599m.g|	gla.S	N/B	24.15	19.00	-13.61	0.90	5.21E-17
L2	mi|Xelaev18026413m.g|	slc35b2.L	SLC35B2	23.96	27.17	-0.96	0.92	6.66E-19
L2	mi|Xelaev18016734m.g|	Xelaev18016734m.g	TULP3	23.71	14.44	-10.21	0.85	5.30E-13
L2	mi|Xelaev18044377m.g|	slc15a2.L	SLC15A2	23.66	42.55	5.31	0.89	4.78E-16
L2	mi|Xelaev18029741m.g|	kpna4.S	KPNA4	23.41	30.39	5.60	0.96	1.69E-25
L2	mi|Xelaev18006924m.g|	tle1.L	TLE1	23.22	30.34	5.09	0.93	1.89E-19
L2	mi|Xelaev18035353m.g|	srpra.L	SRPRA	22.54	45.11	10.74	0.92	6.68E-19
L2	mi|Xelaev18012685m.g|	por.L	POR	22.34	25.40	4.85	0.83	5.06E-12
L2	mi|Xelaev18015148m.g|	ncdn.S	NCDN	22.18	37.70	10.55	0.91	1.32E-17
L2	mi|Xelaev18009299m.g|	pcdh18.S	PCDH18	21.76	16.41	-9.25	0.77	1.33E-09
L2	mi|Xelaev18024951m.g|	emc8.S	EMC8	21.75	39.99	12.55	0.96	2.70E-24
L2	mi|Xelaev18017372m.g|	gnptab.L	GNPTAB	21.56	35.69	6.62	0.89	2.70E-16
L2	mi|Xelaev18016359m.g|	alg8.S	ALG8	21.55	33.91	4.42	0.95	1.44E-22
L2	mi|Xelaev18027277m.g|	mdn1.L	MDN1	21.44	30.57	-3.95	0.80	9.50E-11
L2	mi|Xelaev18029563m.g|	Xelaev18029563m.g	RNPEPL1	21.41	37.40	6.27	0.95	1.41E-22
L2	mi|Xelaev18044105m.g|	jmjd6.L	JMJD6	21.38	33.34	1.03	0.87	2.94E-14
L2	mi|Xelaev18045593m.g|	rhot2.L	RHOT2	21.11	28.51	0.96	0.91	2.01E-17

L2	mi|Xelaev18004729m.g|	rcl1.S	RCL1	21.07	15.06	-9.63	0.88	7.82E-15
L2	mi|Xelaev18003086m.g|	src.L	SRC	20.82	29.68	4.70	0.95	3.86E-22
L2	mi|Xelaev18046864m.g|	nol11.S	NOL11	20.72	31.18	4.48	0.94	1.14E-21
L2	mi|Xelaev18001143m.g|	ncln.S	NCLN	20.69	23.27	1.14	0.89	3.01E-16
L2	mi|Xelaev18020782m.g|	cd276.S	CD276	20.66	38.32	1.15	0.90	7.61E-17
L2	mi|Xelaev18046718m.g|	srp68.S	SRP68	20.50	26.68	6.90	0.92	6.82E-19
L2	mi|Xelaev18035213m.g|	hspa8.L	HSPA8	20.42	19.43	1.97	0.91	5.96E-18
L2	mi|Xelaev18017705m.g|	alg12.L	ALG12	20.31	26.30	-0.67	0.95	2.54E-22
L2	mi|Xelaev18017829m.g|	hspa14.L	HSPA14	20.18	35.15	21.20	0.88	4.86E-15
L2	mi|Xelaev18034087m.g|	exosc4.S	EXOSC4	20.09	18.71	-3.14	0.85	1.72E-13
L2	mi|Xelaev18034362m.g|	atad1.L	ATAD1	19.72	28.15	-3.65	0.91	5.59E-18
L2	mi|Xelaev18011800m.g|	baz1b.L	BAZ1B	19.24	18.36	-4.47	0.83	5.05E-12
L2	mi|Xelaev18023764m.g|	nol8.L	NOL8	19.13	42.59	15.82	0.85	4.06E-13
L2	mi|Xelaev18014742m.g|	ddx3x.S	DDX3X	18.95	45.03	17.39	0.83	1.97E-12
L2	mi|Xelaev18009334m.g|	sec24b.S	SEC24B	18.92	30.82	-1.13	0.82	1.42E-11
L2	mi|Xelaev18039548m.g|	cpsf2.L	CPSF2	18.81	21.58	0.89	0.87	9.00E-15
L2	mi|Xelaev18033621m.g|	myl12b.S	MYL12A	18.54	24.06	-4.07	0.85	3.07E-13
L2	mi|Xelaev18016464m.g|	cdk4.S	N/B	18.37	24.21	-9.74	0.87	1.80E-14
L2	mi|Xelaev18022567m.g|	cenpn.L	CENPN	18.36	30.11	3.29	0.74	9.54E-09
L2	mi|Xelaev18045107m.g|	eef2kmt.L	EEF2KMT	18.29	13.61	-8.51	0.74	1.21E-08
L2	mi|Xelaev18015669m.g|	sox21.S	SOX21	18.27	12.91	-9.96	0.77	1.19E-09
L2	mi|Xelaev18017286m.g|	wdr96.L	CFAP43	18.24	32.90	-4.39	0.86	9.23E-14
L2	mi|Xelaev18025289m.g|	cpt2.S	CPT2	18.17	37.93	17.76	0.86	7.43E-14
L2	mi|Xelaev18028448m.g|	cad.L	CAD	18.13	15.55	-7.65	0.88	4.54E-15
L2	mi|Xelaev18044280m.g|	ern1.L	ERN1	18.04	32.50	2.26	0.88	2.80E-15
L2	mi|Xelaev18005523m.g|	smad1.L	SMAD9	17.95	19.76	-12.54	0.90	1.64E-16

L2	mi|Xelaev18038759m.g|	uprt.L	UPRT	17.69	37.00	15.25	0.94	3.87E-21
L2	mi|Xelaev18044791m.g|	nup35.L	NUP35	17.66	22.19	-5.20	0.91	1.23E-17
L2	mi|Xelaev18002192m.g|	Xelaev18002192m.g	CDKL4	17.40	16.81	-9.45	0.81	3.79E-11
L2	mi|Xelaev18024927m.g|	usp10.S	USP10	17.16	36.01	8.09	0.93	1.54E-19
L2	mi|Xelaev18028255m.g|	fzd3.L	FZD3	17.14	19.65	0.14	0.87	2.33E-14
L2	mi|Xelaev18036882m.g|	edrf1.S	EDRF1	17.04	39.13	11.10	0.91	9.35E-18
L2	mi|Xelaev18026086m.g|	mtif2.L	MTIF2	17.04	26.87	1.25	0.80	8.76E-11
L2	mi|Xelaev18029823m.g|	c3orf58.S	DIPK2A	16.84	27.35	1.92	0.77	1.39E-09
L2	mi|Xelaev18026418m.g|	mrpl14.L	MRPL14	16.76	18.99	5.27	0.75	4.85E-09
L2	mi|Xelaev18044931m.g|	slc39a10.L	SLC39A10	16.59	25.77	-0.07	0.90	1.09E-16
L2	mi|Xelaev18013640m.g|	smarcd1.L	SMARCD1	16.56	15.69	-6.52	0.75	4.39E-09
L2	mi|Xelaev18025004m.g|	sf3b3.S	SF3B3	16.51	28.54	-1.65	0.94	4.65E-21
L2	mi|Xelaev18031081m.g|	stt3b.L	STT3B	16.49	28.80	5.11	0.94	7.01E-21
L2	mi|Xelaev18028561m.g|	xpo5.S	XPO5	16.47	10.65	-2.51	0.80	5.19E-11
L2	mi|Xelaev18009632m.g|	Xelaev18009632m.g	N/B	16.32	27.54	5.76	0.84	9.70E-13
L2	mi|Xelaev18037707m.g|	ntn1.S	NTN1	15.58	12.66	-11.66	0.82	6.36E-12
L2	mi|Xelaev18041985m.g|	rhog-like.1.S	RHOG	15.56	38.63	7.08	0.92	1.76E-18
L2	mi|Xelaev18044663m.g|	fastkd1.L	FASTKD1	15.52	33.82	5.62	0.94	1.75E-21
L2	mi|Xelaev18042120m.g|	Xelaev18042120m.g	AMMECR1	15.51	24.37	1.72	0.87	1.55E-14
L2	mi|Xelaev18007864m.g|	naa25.L	NAA25	15.32	27.51	-0.13	0.90	2.35E-16
L2	mi|Xelaev18026644m.g|	loc100216148.L	ADPGK	15.24	42.25	13.01	0.80	5.64E-11
L2	mi|Xelaev18034541m.g|	bub3.L	BUB3	15.06	30.98	10.88	0.91	4.80E-18
L2	mi|Xelaev18041731m.g|	plxnb3.S	PLXNB3	15.04	17.07	-13.25	0.60	1.46E-05
L2	mi|Xelaev18018533m.g|	tia1.L	TIA1	14.68	22.60	7.53	0.93	5.85E-20
L2	mi|Xelaev18047703m.g|	xpo6.S	XPO6	14.56	21.27	-4.93	0.88	4.05E-15
L2	mi|Xelaev18023999m.g|	rpn1.L	RPN1	14.41	18.31	1.12	0.87	2.46E-14

L2	mi|Xelaev18017367m.g|	utp20.L	UTP20	14.36	29.13	1.54	0.92	1.81E-18
L2	mi|Xelaev18015395m.g|	ube2g1.S	UBE2G1	14.20	31.67	7.62	0.90	9.61E-17
L2	mi|Xelaev18038114m.g|	suv39h1.L	SUV39H1	14.19	21.27	-0.65	0.86	7.27E-14
L2	mi|Xelaev18022477m.g|	prmt7.L	PRMT7	14.16	39.63	13.11	0.87	2.33E-14
L2	mi|Xelaev18045674m.g|	pelo.L	PELO	14.11	27.76	8.94	0.79	1.24E-10
L2	mi|Xelaev18029875m.g|	dgkd-like.S	DGKD	14.05	20.09	-6.90	0.80	7.86E-11
L2	mi|Xelaev18039130m.g|	tapbp.L	TAPBP	14.00	15.35	-8.58	0.85	3.15E-13
L2	mi|Xelaev18010671m.g|	naa25.S	NAA25	13.98	34.55	3.65	0.92	3.17E-18
L2	mi|Xelaev18007546m.g|	gcn1.L	GCN1	13.87	18.20	-2.25	0.89	5.11E-16
L2	mi|Xelaev18017510m.g|	n6amt1-like.L	N6AMT1	13.83	32.78	14.84	0.83	4.22E-12
L2	mi|Xelaev18046067m.g|	eftud2.S	EFTUD2	13.35	12.56	-6.38	0.75	5.28E-09
L2	mi|Xelaev18022733m.g|	slc7a10.L	SLC7A10	13.22	34.54	9.20	0.92	5.60E-19
L2	mi|Xelaev18035637m.g|	atad3a.L	ATAD3A	12.86	37.35	18.18	0.91	3.22E-17
L2	mi|Xelaev18029543m.g|	psmd2.S	PSMD2	12.83	16.92	1.97	0.90	9.22E-17
L2	mi|Xelaev18025423m.g|	klhl20.S	KLHL20	12.80	14.78	-4.20	0.87	3.60E-14
L2	mi|Xelaev18027431m.g|	loc100490363-like.L	TTC16	12.80	29.81	6.57	0.87	2.10E-14
L2	mi|Xelaev18023666m.g|	mief1.L	MIEF1	12.61	31.62	9.18	0.94	2.16E-21
L2	mi|Xelaev18040984m.g|	slc35a2.S	SLC35A2	12.52	27.75	1.98	0.75	6.09E-09
L2	mi|Xelaev18041192m.g|	dync1h1.S	DYNC1H1	12.43	20.51	0.58	0.86	8.15E-14
L2	mi|Xelaev18009883m.g|	pik3r2-like.S	PIK3R2	12.31	22.44	1.62	0.70	1.35E-07
L2	mi|Xelaev18002793m.g|	pkn3.L	PKN2	12.23	11.04	-10.35	0.80	1.04E-10
L2	mi|Xelaev18009954m.g|	kxd1.S	KXD1	12.09	32.67	8.20	0.88	6.05E-15
L2	mi|Xelaev18046631m.g|	rbl1.S	RBL1	12.09	25.01	1.15	0.80	6.82E-11
L2	mi|Xelaev18017801m.g|	sema3c.L	SEMA3C	11.90	11.76	-15.06	0.82	6.35E-12
L2	mi|Xelaev18028930m.g|	fez2.S	FEZ2	11.88	39.38	8.41	0.92	4.93E-19
L2	mi|Xelaev18038538m.g|	timm8a.L	TIMM8A	11.82	31.37	16.99	0.90	8.72E-17

L2	mi|Xelaev18014276m.g|	usp16.S	USP16	11.77	34.55	6.37	0.86	7.62E-14
L2	mi|Xelaev18047090m.g|	rif1.S	RIF1	11.67	22.21	3.64	0.85	2.65E-13
L2	mi|Xelaev18020274m.g|	loc101734133.S	MCHR1	11.64	23.03	-6.27	0.90	1.35E-16
L2	mi|Xelaev18010449m.g|	gcn1.S	GCN1	11.59	20.24	-4.01	0.91	8.42E-18
L2	mi|Xelaev18005442m.g|	fnip2.L	FNIP2	11.50	27.42	1.11	0.91	2.72E-17
L2	mi|Xelaev18008284m.g|	dhx29.L	DHX29	11.30	19.11	-6.78	0.86	9.62E-14
L2	mi|Xelaev18044054m.g|	hn1.L	JPT1	11.09	17.30	0.57	0.87	1.80E-14
L2	mi|Xelaev18036841m.g|	bub3.S	BUB3	11.05	12.10	-6.89	0.81	4.00E-11
L2	mi|Xelaev18023003m.g|	dock6-like.L	DOCK7	11.01	11.48	-2.25	0.85	1.74E-13
L2	mi|Xelaev18018025m.g|	adpgk.L	ADPGK	11.01	24.38	-0.88	0.79	1.62E-10
L2	mi|Xelaev18027641m.g|	kpna4.L	KPNA4	10.89	28.48	11.27	0.91	8.15E-18
L2	mi|Xelaev18007688m.g|	top3b.L	TOP3B	10.85	13.44	-6.98	0.79	1.21E-10
L2	mi|Xelaev18027456m.g|	tfrc.L	TFRC	10.84	27.44	2.16	0.79	1.33E-10
L2	mi|Xelaev18015288m.g|	ints2.S	INTS2	10.50	25.56	2.44	0.91	1.71E-17
L2	mi|Xelaev18032872m.g|	kif5b.S	KIF5B	10.48	28.72	3.12	0.86	5.24E-14
L2	mi|Xelaev18024911m.g|	pdf.S	PDF	10.42	23.03	-3.94	0.90	2.32E-16
L2	mi|Xelaev18009925m.g|	mier2.S	N/B	10.35	33.38	11.46	0.78	4.35E-10
L2	mi|Xelaev18005749m.g|	metap1.L	METAP1	10.29	35.19	11.87	0.86	6.11E-14
L2	mi|Xelaev18015376m.g|	tyw1.S	TYW1	10.28	31.28	14.29	0.88	1.94E-15
L2	mi|Xelaev18033202m.g|	ctnnb1.S	CTNNB1	10.16	17.43	-3.14	0.94	6.95E-21
L3	mi|Xelaev18021302m.g|	hsp90b1.S	HSP90B1	47.04	47.41	13.08	0.96	8.40E-24
L3	mi|Xelaev18017704m.g|	creld2.L	CRELD2	43.83	45.63	10.02	0.93	4.43E-20
L3	mi|Xelaev18047055m.g|	mcm6.2.S	MCM6	43.50	46.86	11.84	0.96	8.67E-24
L3	mi|Xelaev18028222m.g|	zmcm3.L	MCM3	40.34	39.43	11.41	0.93	1.01E-19
L3	mi|Xelaev18012617m.g|	ak2.L	AK2	39.61	42.04	9.45	0.94	1.17E-20
L3	mi|Xelaev18039725m.g|	trmt61a.L	TRMT61A	39.04	42.53	9.31	0.92	4.66E-19

L3	mi|Xelaev18024200m.g|	lbhd1.S	C11orf98	37.69	45.82	11.86	0.95	9.23E-24
L3	mi|Xelaev18025556m.g|	mcm5.S	MCM5	37.68	36.52	5.92	0.92	9.54E-19
L3	mi|Xelaev18031319m.g|	pdia4.L	PDIA4	37.02	43.46	10.72	0.93	1.67E-20
L3	mi|Xelaev18025893m.g|	mcm2.S	MCM2	36.90	35.23	14.43	0.87	9.75E-15
L3	mi|Xelaev18033587m.g|	apcdd1.S	APCDD1	36.86	47.16	12.26	0.93	1.25E-19
L3	mi|Xelaev18014500m.g|	wdr4.S	WDR4	36.79	39.77	7.71	0.92	8.10E-19
L3	mi|Xelaev18023987m.g|	mcm2.L	MCM2	36.64	38.05	8.80	0.91	1.58E-17
L3	mi|Xelaev18016343m.g|	ctsc.S	CTSC	36.58	19.54	-3.35	0.88	2.88E-15
L3	mi|Xelaev18023040m.g|	ebna1bp2.L	EBNA1BP2	36.17	40.87	8.33	0.90	4.70E-17
L3	mi|Xelaev18031991m.g|	mcm4.L	MCM4	35.42	33.50	10.14	0.92	2.96E-18
L3	mi|Xelaev18047177m.g|	cdca7.S	CDCA7	34.72	39.31	10.07	0.91	5.07E-18
L3	mi|Xelaev18006725m.g|	plin2.L	PLIN2	34.70	20.66	-9.26	0.84	9.01E-13
L3	mi|Xelaev18015852m.g|	cnpy2.S	CNPY2	34.01	33.06	9.72	0.92	1.12E-18
L3	mi|Xelaev18014570m.g|	wdr77.S	WDR77	33.70	27.85	7.00	0.93	9.34E-20
L3	mi|Xelaev18044530m.g|	mcm6.2.L	MCM6	33.59	35.74	13.05	0.90	4.04E-17
L3	mi|Xelaev18033539m.g|	zadh2.S	ZADH2	32.78	32.01	3.05	0.90	3.63E-17
L3	mi|Xelaev18013320m.g|	Xelaev18013320m.g	PRKAG1	31.99	44.10	19.74	0.94	1.83E-21
L3	mi|Xelaev18007841m.g|	gpn3.L	GPN3	31.72	45.48	21.58	0.89	5.58E-16
L3	mi|Xelaev18019056m.g|	Xelaev18019056m.g	CALR	31.51	34.73	4.33	0.91	3.00E-17
L3	mi|Xelaev18044707m.g|	cdca7.L	CDCA7	30.89	36.82	8.90	0.86	8.17E-14
L3	mi|Xelaev18026651m.g|	dhx57.L	DHX57	30.74	39.26	9.79	0.88	2.00E-15
L3	mi|Xelaev18039773m.g|	mthfd1.L	MTHFD1	30.09	26.73	8.48	0.86	4.83E-14
L3	mi|Xelaev18035296m.g|	stt3a.L	STT3A	29.91	34.13	5.10	0.98	2.39E-29
L3	mi|Xelaev18033701m.g|	mcm4.S	MCM4	29.62	34.05	14.19	0.88	4.60E-15
L3	mi|Xelaev18006123m.g|	aptx.L	APTX	29.58	47.36	18.37	0.89	1.13E-15
L3	mi|Xelaev18007565m.g|	mlec.L	MLEC	29.20	39.75	10.90	0.93	1.24E-19

L3	mi|Xelaev18028581m.g|	ccdc167.S	CCDC167	29.10	37.08	6.44	0.92	4.31E-19
L3	mi|Xelaev18027197m.g|	cd164.L	CD164	28.92	38.73	12.82	0.86	5.70E-14
L3	mi|Xelaev18044264m.g|	nol11.L	NOL11	28.48	45.93	11.88	0.94	7.78E-22
L3	mi|Xelaev18025763m.g|	gmppb.S	GMPPB	28.43	14.99	-7.10	0.81	2.51E-11
L3	mi|Xelaev18023542m.g|	mcm5.L	MCM5	28.05	27.36	1.83	0.92	1.09E-18
L3	mi|Xelaev18020556m.g|	pdia3.S	PDIA3	27.97	28.12	1.41	0.88	6.88E-15
L3	mi|Xelaev18007442m.g|	rbm19.L	RBM19	27.48	33.77	9.87	0.75	4.72E-09
L3	mi|Xelaev18037790m.g|	Xelaev18037790m.g	PRMT1	27.37	35.90	9.82	0.96	4.68E-24
L3	mi|Xelaev18041315m.g|	cpsf2.S	CPSF2	26.92	39.88	9.89	0.87	1.40E-14
L3	mi|Xelaev18018758m.g|	bmp1.L	BMP1	26.86	15.00	-8.91	0.84	7.11E-13
L3	mi|Xelaev18047021m.g|	Xelaev18047021m.g	TUBA1A	26.63	27.81	-3.67	0.75	4.28E-09
L3	mi|Xelaev18026702m.g|	desi2.L	DESI2	26.54	33.00	1.78	0.85	1.63E-13
L3	mi|Xelaev18034801m.g|	hells.L	HELLS	25.95	33.75	8.49	0.88	6.99E-15
L3	mi|Xelaev18015757m.g|	Xelaev18015757m.g	N/B	25.00	33.87	9.45	0.82	1.03E-11
L3	mi|Xelaev18025879m.g|	chchd4.S	CHCHD4	24.89	31.71	0.74	0.89	4.34E-16
L3	mi|Xelaev18006829m.g|	rcl1.L	RCL1	24.86	35.15	9.74	0.89	1.16E-15
L3	mi|Xelaev18045328m.g|	kdelr1.L	KDELR2	24.66	33.90	7.41	0.94	7.44E-22
L3	mi|Xelaev18023178m.g|	orc1.L	ORC1	24.49	33.77	9.70	0.84	1.38E-12
L3	mi|Xelaev18047548m.g|	nudt1.S	NUDT1	24.05	40.36	8.02	0.93	1.46E-19
L3	mi|Xelaev18007548m.g|	pxn.L	PXN	23.67	37.46	8.43	0.79	1.69E-10
L3	mi|Xelaev18022464m.g|	nup93.L	NUP93	23.63	27.84	10.14	0.89	7.70E-16
L3	mi|Xelaev18007810m.g|	b3gnt4.L	B3GNT4	23.61	30.29	13.25	0.82	1.60E-11
L3	mi|Xelaev18043267m.g|	slc35b1.L	SLC35B1	23.56	33.25	5.54	0.91	2.44E-17
L3	mi|Xelaev18022257m.g|	ganab.L	GANAB	23.51	37.44	6.32	0.94	9.92E-21
L3	mi|Xelaev18043753m.g|	p4hb.L	P4HB	23.17	28.22	2.31	0.96	2.72E-24
L3	mi|Xelaev18033397m.g|	txndc5.S	TXNDC5	23.11	33.82	5.32	0.95	1.04E-23

L3	mi|Xelaev18017756m.g|	Xelaev18017756m.g	SYPL1	23.08	33.67	1.74	0.91	1.43E-17
L3	mi|Xelaev18034687m.g|	exosc1.L	EXOSC1	22.76	32.75	11.22	0.93	9.56E-20
L3	mi|Xelaev18030542m.g|	ngfr.L	NGFR	22.64	20.74	-7.53	0.69	2.78E-07
L3	mi|Xelaev18007055m.g|	prmt5.L	PRMT5	22.23	36.34	9.26	0.97	8.82E-27
L3	mi|Xelaev18028917m.g|	srsf7.S	SRSF7	22.17	26.42	0.63	0.72	4.95E-08
L3	mi|Xelaev18011705m.g|	sdf2.L	SDF2	21.92	36.30	7.98	0.96	1.52E-24
L3	mi|Xelaev18013439m.g|	Xetrov90006328m.L	MARS	21.87	33.92	3.66	0.89	8.24E-16
L3	mi|Xelaev18004046m.g|	rbbp4.S	RBBP4	21.69	22.18	1.42	0.86	9.04E-14
L3	mi|Xelaev18011675m.g|	blmh.L	BLMH	21.41	40.20	14.90	0.90	1.16E-16
L3	mi|Xelaev18007579m.g|	drg1.L	DRG1	21.38	32.81	10.06	0.89	5.43E-16
L3	mi|Xelaev18019201m.g|	mcm7.L	MCM7	21.32	21.75	0.67	0.92	1.71E-18
L3	mi|Xelaev18046741m.g|	mfsd11.S	N/B	21.31	31.43	-0.17	0.86	1.06E-13
L3	mi|Xelaev18012612m.g|	rbbp4.L	RBBP4	21.29	25.66	2.29	0.85	2.37E-13
L3	mi|Xelaev18022556m.g|	Xelaev18022556m.g	GCSH	21.28	34.37	14.25	0.86	1.32E-13
L3	mi|Xelaev18046010m.g|	eif6.S	EIF6	21.26	17.61	-3.83	0.93	3.09E-20
L3	mi|Xelaev18031769m.g|	zadh2.L	ZADH2	21.22	33.46	-0.79	0.95	4.00E-22
L3	mi|Xelaev18011098m.g|	lipg.S	LIPG	21.11	32.70	27.04	0.43	3.48E-03
L3	mi|Xelaev18008451m.g|	lipg.L	LIPG	20.99	33.18	6.17	0.56	6.57E-05
L3	mi|Xelaev18041732m.g|	Xelaev18041732m.g	ABCD1	20.90	24.03	-4.11	0.79	1.85E-10
L3	mi|Xelaev18000877m.g|	mcm7.S	MCM7	20.52	15.25	-0.15	0.75	3.46E-09
L3	mi|Xelaev18024906m.g|	nob1.S	NOB1	20.33	32.26	9.53	0.87	1.25E-14
L3	mi|Xelaev18036061m.g|	Xelaev18036061m.g	PRMT1	20.31	31.25	2.38	0.96	3.33E-25
L3	mi|Xelaev18000035m.g|	plp2.L	PLP2	20.15	16.91	-9.95	0.83	4.23E-12
L3	mi|Xelaev18020198m.g|	pbdc1.S	PBDC1	20.05	30.82	9.27	0.82	1.10E-11
L3	mi|Xelaev18046413m.g|	loc100497487.S	PTRH1	19.93	41.70	26.29	0.88	5.59E-15
L3	mi|Xelaev18022097m.g|	Xelaev18022097m.g	POLR2L	19.84	20.70	-1.37	0.91	1.36E-17

L3	mi|Xelaev18018585m.g|	lman2l.L	LMAN2L	19.76	37.61	8.91	0.91	2.00E-17
L3	mi|Xelaev18027417m.g|	polr2h.L	POLR2H	19.66	29.02	12.42	0.92	5.83E-19
L3	mi|Xelaev18006181m.g|	dohh.L	DOHH	19.49	27.38	11.86	0.93	4.74E-20
L3	mi|Xelaev18036139m.g|	hspbp1.L	HSPBP1	19.36	31.14	11.94	0.81	1.94E-11
L3	mi|Xelaev18022454m.g|	heatr3.L	HEATR3	19.32	28.41	0.11	0.86	1.26E-13
L3	mi|Xelaev18010780m.g|	wdr36.S	WDR36	19.27	31.39	5.27	0.92	3.49E-19
L3	mi|Xelaev18013779m.g|	mipep.L	MIPEP	19.18	41.76	12.88	0.89	1.29E-15
L3	mi|Xelaev18013700m.g|	alg5.L	N/B	19.12	15.98	-5.07	0.86	4.42E-14
L3	mi|Xelaev18016354m.g|	Xelaev18016354m.g	CLNS1A	18.99	19.26	6.69	0.94	1.30E-20
L3	mi|Xelaev18032746m.g|	ngfr.S	NGFR	18.93	32.52	1.12	0.89	6.60E-16
L3	mi|Xelaev18023851m.g|	hemk1.L	HEMK1	18.91	45.12	20.56	0.92	1.62E-18
L3	mi|Xelaev18032338m.g|	derl1.L	DERL1	18.68	13.87	-3.92	0.83	4.54E-12
L3	mi|Xelaev18037352m.g|	stt3a.S	STT3A	18.59	29.56	4.51	0.97	1.46E-28
L3	mi|Xelaev18008214m.g|	slc30a5.L	SLC30A5	18.49	31.54	6.34	0.93	1.75E-19
L3	mi|Xelaev18025282m.g|	gpx7.S	GPX7	18.37	38.27	17.71	0.88	4.93E-15
L3	mi|Xelaev18024592m.g|	prmt3.S	N/B	17.96	25.38	1.92	0.88	4.11E-15
L3	mi|Xelaev18026725m.g|	ttc27.L	TTC27	17.95	35.63	14.01	0.87	2.10E-14
L3	mi|Xelaev18047701m.g|	nme4.S	N/B	17.93	26.56	2.95	0.74	6.91E-09
L3	mi|Xelaev18044062m.g|	llgl2.L	LLGL2	17.85	25.60	-4.90	0.81	2.54E-11
L3	mi|Xelaev18032559m.g|	slc52a1.L	SLC52A1	17.82	24.33	-1.23	0.88	2.18E-15
L3	mi|Xelaev18039051m.g|	ppp5c.L	N/B	17.61	31.33	4.23	0.80	7.39E-11
L3	mi|Xelaev18041645m.g|	ntmt1.S	NTMT1	17.50	29.93	-1.20	0.87	8.93E-15
L3	mi|Xelaev18030892m.g|	tac1.L	TAC1	17.43	14.79	-7.36	0.50	5.42E-04
L3	mi|Xelaev18027656m.g|	gfm1.L	GFM1	17.40	42.27	25.06	0.93	2.03E-19
L3	mi|Xelaev18004050m.g|	ak2.S	N/B	17.21	37.67	15.61	0.89	3.21E-16
L3	mi|Xelaev18025521m.g|	nhp2l1.S	N/B	17.20	18.57	-3.94	0.91	2.62E-17

L3	mi|Xelaev18031742m.g|	bloc1s4.L	BLOC1S4	17.15	18.55	-3.96	0.85	4.47E-13
L3	mi|Xelaev18045047m.g|	tra2b.L	TRA2B	17.10	27.25	3.20	0.77	7.80E-10
L3	mi|Xelaev18004506m.g|	hells.S	HELLS	17.08	25.01	10.29	0.85	5.06E-13
L3	mi|Xelaev18009750m.g|	sgta.S	SGTA	17.00	28.52	13.59	0.92	1.71E-18
L3	mi|Xelaev18027671m.g|	ssr3.L	SSR3	16.98	16.99	-2.51	0.94	5.65E-21
L3	mi|Xelaev18024458m.g|	Xelaev18024458m.g	N/B	16.93	19.83	9.19	0.82	1.20E-11
L3	mi|Xelaev18009082m.g|	ppat.S	PPAT	16.77	29.97	3.07	0.88	2.61E-15
L3	mi|Xelaev18033246m.g|	psma2.S	PSMA2	16.76	27.28	15.63	0.89	6.29E-16
L3	mi|Xelaev18025251m.g|	cmpk1.S	CMPK1	16.68	29.47	11.39	0.92	3.24E-19
L3	mi|Xelaev18003710m.g|	mogs.L	MOGS	16.60	31.36	10.66	0.85	3.45E-13
L3	mi|Xelaev18022120m.g|	capn1.L	CAPN1	16.52	11.01	-8.67	0.76	2.57E-09
L3	mi|Xelaev18035316m.g|	rexo2.L	N/B	16.21	28.70	9.20	0.90	2.37E-16
L3	mi|Xelaev18025924m.g|	slc25a20.2.S	SLC25A20	16.05	25.88	-1.79	0.89	6.44E-16
L3	mi|Xelaev18020417m.g|	cln6.S	CLN6	16.04	33.29	7.84	0.87	2.63E-14
L3	mi|Xelaev18008550m.g|	stoml2.L	STOML2	15.82	17.32	-4.01	0.71	8.00E-08
L3	mi|Xelaev18026522m.g|	xpo1.L	XPO1	15.60	33.51	10.16	0.83	3.69E-12
L3	mi|Xelaev18017754m.g|	ccdc71l.L	CCDC71L	15.52	28.31	2.56	0.80	5.96E-11
L3	mi|Xelaev18015874m.g|	cdk2.S	N/B	15.50	22.23	-1.32	0.80	8.38E-11
L3	mi|Xelaev18015587m.g|	pcid2.S	PCID2	15.38	24.11	6.34	0.85	2.42E-13
L3	mi|Xelaev18012233m.g|	znhit3.L	ZNHIT3	15.32	23.13	12.17	0.83	5.61E-12
L3	mi|Xelaev18020981m.g|	Xelaev18020981m.g	SYPL1	15.16	28.24	-2.35	0.84	7.91E-13
L3	mi|Xelaev18022560m.g|	Xelaev18022560m.g	GCSH	15.11	30.01	5.87	0.77	1.02E-09
L3	mi|Xelaev18042325m.g|	ddx26b.S	N/B	15.05	25.34	-5.41	0.81	2.21E-11
L3	mi|Xelaev18009860m.g|	mob3a.S	MOB3A	14.86	31.36	2.54	0.84	1.78E-12
L3	mi|Xelaev18023962m.g|	chchd4.L	N/B	14.84	38.18	20.01	0.94	5.45E-22
L3	mi|Xelaev18024234m.g|	Xelaev18024234m.g	TRMT112	14.83	19.50	1.59	0.94	4.65E-21

L3	mi|Xelaev18024202m.g|	ganab.S	N/B	14.76	27.06	-0.52	0.90	1.79E-16
L3	mi|Xelaev18023937m.g|	sec13.L	SEC13	14.75	21.60	-0.12	0.92	2.40E-18
L3	mi|Xelaev18027925m.g|	gk5.L	GK5	14.71	27.23	-3.60	0.85	4.85E-13
L3	mi|Xelaev18037591m.g|	hes3.S	HES3	14.69	19.50	-3.87	0.76	2.23E-09
L3	mi|Xelaev18005042m.g|	haus3.L	N/B	14.58	13.64	-4.91	0.81	3.33E-11
L3	mi|Xelaev18045844m.g|	hm13.S	HM13	14.57	24.73	1.84	0.95	1.15E-22
L3	mi|Xelaev18022459m.g|	adcy7.L	ADCY7	14.51	20.31	-9.41	0.70	1.38E-07
L3	mi|Xelaev18018952m.g|	gipc1.L	GIPC1	14.50	19.25	-4.21	0.83	2.82E-12
L3	mi|Xelaev18039674m.g|	tmx1.L	TMX1	14.49	33.91	7.26	0.89	2.63E-16
L3	mi|Xelaev18043986m.g|	e2f1.L	E2F1	14.44	23.88	6.05	0.73	2.34E-08
L3	mi|Xelaev18027557m.g|	Xelaev18027557m.g	ACTL6A	14.44	27.22	4.65	0.90	7.41E-17
L3	mi|Xelaev18023326m.g|	tmed5.L	TMED5	14.30	17.49	4.10	0.78	4.37E-10
L3	mi|Xelaev18039446m.g|	fcf1.L	FCF1	13.98	17.75	-2.44	0.75	4.75E-09
L3	mi|Xelaev18046644m.g|	e2f1.S	E2F1	13.93	20.85	-4.69	0.68	4.26E-07
L3	mi|Xelaev18013470m.g|	mettl21b.L	N/B	13.92	28.26	-0.03	0.82	1.19E-11
L3	mi|Xelaev18027146m.g|	fam26d.L	CALHM4	13.87	23.27	6.70	0.88	4.10E-15
L3	mi|Xelaev18006236m.g|	cirbp.L	N/B	13.73	26.95	17.63	0.93	6.83E-20
L3	mi|Xelaev18025133m.g|	alg6.S	ALG6	13.69	39.21	18.79	0.89	5.52E-16
L3	mi|Xelaev18014175m.g|	blmh.S	BLMH	13.64	33.79	14.66	0.84	9.52E-13
L3	mi|Xelaev18042168m.g|	tspan6.S	TSPAN6	13.60	32.17	9.33	0.90	2.05E-16
L3	mi|Xelaev18013138m.g|	uggt2.L	UGGT2	13.52	30.38	3.46	0.83	4.04E-12
L3	mi|Xelaev18029561m.g|	eif2b5.S	EIF2B5	13.47	40.74	17.51	0.88	2.90E-15
L3	mi|Xelaev18025187m.g|	nasp.S	N/B	13.46	30.01	15.41	0.69	2.20E-07
L3	mi|Xelaev18013387m.g|	tmem198.L	TMEM198	13.43	12.56	-9.03	0.57	5.57E-05
L3	mi|Xelaev18043403m.g|	cse1l.L	CSE1L	13.30	15.92	-1.90	0.82	1.32E-11
L3	mi|Xelaev18025273m.g|	nrdc.S	NRDC	13.30	33.89	7.84	0.91	2.47E-17

L3	mi|Xelaev18024639m.g|	Xelaev18024639m.g	N/B	13.26	23.55	0.00	0.63	4.38E-06
L3	mi|Xelaev18024347m.g|	pkp3.S	PKP3	13.11	22.12	-7.79	0.72	3.89E-08
L3	mi|Xelaev18031629m.g|	eef1e1.L	N/B	13.03	25.17	5.22	0.94	1.05E-20
L3	mi|Xelaev18024332m.g|	fen1.S	FEN1	13.00	29.15	9.97	0.93	2.90E-19
L3	mi|Xelaev18037583m.g|	loc779604.S	C1orf174	13.00	20.24	0.91	0.80	8.68E-11
L3	mi|Xelaev18046031m.g|	slc35b1.S	SLC35B1	12.74	25.13	2.75	0.93	1.92E-19
L3	mi|Xelaev18022776m.g|	orc6.L	ORC6	12.68	21.51	9.08	0.75	5.22E-09
L3	mi|Xelaev18029551m.g|	alg3.S	ALG3	12.68	30.57	3.83	0.90	4.68E-17
L3	mi|Xelaev18003084m.g|	ctnnbl1.L	N/B	12.61	17.07	-1.43	0.73	2.01E-08
L3	mi|Xelaev18044630m.g|	gca.L	GCA	12.50	30.98	2.86	0.85	2.25E-13
L3	mi|Xelaev18012773m.g|	mrps17.L	MRPS17	12.48	25.20	11.52	0.80	5.32E-11
L3	mi|Xelaev18025691m.g|	polr2f.S	POLR2F	12.43	25.66	8.11	0.88	3.79E-15
L3	mi|Xelaev18000134m.g|	ppp1r3b.L	PPP1R3B	12.40	40.68	15.40	0.62	7.82E-06
L3	mi|Xelaev18028947m.g|	tcp1.S	TCP1	12.38	28.99	8.72	0.96	2.10E-24
L3	mi|Xelaev18025085m.g|	lrriq3.S	LRRIQ3	12.12	14.88	-9.31	0.77	1.43E-09
L3	mi|Xelaev18017652m.g|	naa38-like.L	LSM8	12.11	19.36	5.16	0.90	2.22E-16
L3	mi|Xelaev18039667m.g|	nid2.L	NID2	11.97	12.68	-9.53	0.75	5.52E-09
L3	mi|Xelaev18035169m.g|	mtg1.L	MTG1	11.88	25.60	8.13	0.74	6.85E-09
L3	mi|Xelaev18035689m.g|	lepre1.L	P3H1	11.87	33.42	12.72	0.73	1.99E-08
L3	mi|Xelaev18023002m.g|	atg4c.L	ATG4C	11.73	21.91	-1.94	0.92	2.39E-18
L3	mi|Xelaev18010463m.g|	Xelaev18010463m.g	SRSF9	11.70	25.35	1.55	0.93	7.44E-20
L3	mi|Xelaev18008924m.g|	msx1.S	N/B	11.67	15.77	-4.35	0.71	6.06E-08
L3	mi|Xelaev18008749m.g|	cct7.S	CCT7	11.47	19.03	-9.73	0.90	6.44E-17
L3	mi|Xelaev18043048m.g|	mapre1.L	MAPRE1	11.45	19.26	1.19	0.95	4.35E-22
L3	mi|Xelaev18028276m.g|	stmn4.L	STMN4	11.38	16.68	-3.16	0.76	2.42E-09
L3	mi|Xelaev18038688m.g|	magt1-like.L	MAGT1	11.34	27.14	3.85	0.85	1.64E-13

L3	mi|Xelaev18026449m.g|	mkks.L	MKKS	11.31	32.51	14.41	0.92	2.16E-18
L3	mi|Xelaev18039217m.g|	vars.L	VARS	11.28	28.55	10.31	0.90	6.16E-17
L3	mi|Xelaev18015126m.g|	rpa2.S	RPA2	11.21	18.97	10.40	0.76	2.13E-09
L3	mi|Xelaev18025437m.g|	itpa-like.S	N/B	11.17	30.74	12.25	0.91	1.09E-17
L3	mi|Xelaev18030142m.g|	mrpl19.S	MRPL19	11.04	17.72	14.41	0.81	1.73E-11
L3	mi|Xelaev18010930m.g|	slc30a5.S	SLC30A5	11.02	25.73	4.13	0.89	8.24E-16
L3	mi|Xelaev18047428m.g|	hspd1.S	HSPD1	10.93	30.67	18.44	0.85	1.64E-13
L3	mi|Xelaev18021349m.g|	tmed9.S	TMED9	10.91	36.10	14.46	0.97	2.87E-27
L3	mi|Xelaev18013024m.g|	nipa2.L	NIPA2	10.86	24.93	8.22	0.88	6.36E-15
L3	mi|Xelaev18045227m.g|	lfng.L	LFNG	10.82	20.06	-5.09	0.81	4.04E-11
L3	mi|Xelaev18014498m.g|	pknox1.S	PKNOX1	10.82	14.11	-3.97	0.69	2.28E-07
L3	mi|Xelaev18013468m.g|	mettl1.L	METTL1	10.81	21.69	-1.31	0.84	6.84E-13
L3	mi|Xelaev18007704m.g|	comt.L	N/B	10.80	21.57	2.94	0.89	5.70E-16
L3	mi|Xelaev18018997m.g|	dnmt1.L	DNMT1	10.79	14.57	0.43	0.79	1.63E-10
L3	mi|Xelaev18016398m.g|	wnt11.S	WNT11	10.68	17.93	-2.63	0.72	4.37E-08
L3	mi|Xelaev18025755m.g|	usp4.S	USP4	10.67	13.87	-4.35	0.74	8.65E-09
L3	mi|Xelaev18044106m.g|	srsf2.L	N/B	10.61	12.11	-1.67	0.89	6.39E-16
L3	mi|Xelaev18009768m.g|	cirbp.S	N/B	10.46	30.85	21.82	0.90	2.16E-16
L3	mi|Xelaev18018480m.g|	eif3j-like.L	EIF3J	10.45	26.29	3.44	0.91	8.43E-18
L3	mi|Xelaev18009261m.g|	abce1.S	ABCE1	10.38	32.77	11.62	0.97	1.32E-27
L3	mi|Xelaev18025861m.g|	sec13.S	SEC13	10.30	25.53	12.63	0.85	4.69E-13
L3	mi|Xelaev18022292m.g|	slc29a2.L	SLC29A2	10.28	33.27	12.08	0.90	4.20E-17
L3	mi|Xelaev18027501m.g|	b3gnt5.L	B3GNT5	10.27	31.05	4.73	0.70	1.51E-07
L3	mi|Xelaev18044357m.g|	itgb5.L	ITGB5	10.26	33.50	5.91	0.84	9.03E-13
L3	mi|Xelaev18037879m.g|	Xelaev18037879m.g	KDELR1	10.22	22.98	7.31	0.87	3.14E-14
L3	mi|Xelaev18042470m.g|	vars.S	VARS	10.10	29.26	6.12	0.82	1.47E-11

L3	mi|Xelaev18012826m.g|	alcam.L	ALCAM	10.04	15.46	-12.26	0.88	2.04E-15
D3	mi|Xelaev18025339m.g|	rgs8.S	RGS8	44.84	48.40	4.47	0.89	1.26E-15
D3	mi|Xelaev18041431m.g|	loc101731931.S	CYR61	36.15	53.05	25.96	0.87	8.24E-15
D3	mi|Xelaev18010763m.g|	cdo1.S	CDO1	35.71	37.77	-2.43	0.91	1.02E-17
D3	mi|Xelaev18026738m.g|	vip.L	VIP	35.68	23.10	-7.48	0.86	1.15E-13
D3	mi|Xelaev18017751m.g|	hbp1.L	HBP1	34.99	52.52	21.83	0.94	2.56E-21
D3	mi|Xelaev18008055m.g|	pcsk1.L	PCSK1	34.07	45.70	-1.34	0.79	1.63E-10
D3	mi|Xelaev18036661m.g|	slc18a2-like.2.S	SLC18A2	33.64	39.99	15.00	0.85	3.16E-13
D3	mi|Xelaev18021718m.g|	calca.L	N/B	32.89	54.07	13.29	0.65	2.27E-06
D3	mi|Xelaev18020629m.g|	scg3.S	SCG3	32.43	39.47	-6.94	0.90	1.39E-16
D3	mi|Xelaev18031724m.g|	nrsn1.L	NRSN1	31.82	30.08	-6.98	0.79	1.44E-10
D3	mi|Xelaev18030959m.g|	hdac9.L	HDAC9	31.12	39.88	7.86	0.88	3.05E-15
D3	mi|Xelaev18015657m.g|	loc100486181.S	APOBEC3A	30.72	41.06	16.67	0.91	1.32E-17
D3	mi|Xelaev18043846m.g|	nbr1.L	NBR1	30.36	48.80	19.93	0.94	1.01E-21
D3	mi|Xelaev18012367m.g|	tbl1x.L	TBL1X	29.73	31.75	-6.75	0.94	9.33E-21
D3	mi|Xelaev18041772m.g|	emd.S	EMD	28.77	49.36	18.71	0.94	4.79E-21
D3	mi|Xelaev18025749m.g|	loc100487499.S	N/B	27.70	42.29	18.57	0.85	4.36E-13
D3	mi|Xelaev18045353m.g|	acsm3.L	ACSM3	27.57	32.04	-1.38	0.85	5.00E-13
D3	mi|Xelaev18017836m.g|	upf2.L	UPF2	27.18	45.24	10.38	0.89	3.45E-16
D3	mi|Xelaev18044101m.g|	st6galnac2.L	ST6GALNAC2	27.04	16.31	-9.00	0.87	2.29E-14
D3	mi|Xelaev18007692m.g|	ypel1.L	YPEL1	26.37	46.79	13.95	0.93	1.42E-19
D3	mi|Xelaev18022109m.g|	Xelaev18022109m.g	N/B	26.07	11.42	-7.66	0.82	6.62E-12
D3	mi|Xelaev18030882m.g|	pdk4.L	N/B	25.96	28.86	-4.79	0.89	8.47E-16
D3	mi|Xelaev18013970m.g|	stard10.L	STARD10	25.49	34.70	-5.78	0.84	1.18E-12
D3	mi|Xelaev18039005m.g|	loc100127779.L	CCDC160	25.08	35.95	11.84	0.94	1.47E-20
D3	mi|Xelaev18027093m.g|	eya4-like.L	EYA4	25.07	26.23	-5.51	0.83	2.31E-12

D3	mi|Xelaev18038781m.g|	smarca1.L	SMARCA1	24.67	31.46	-4.58	0.87	1.02E-14
D3	mi|Xelaev18020469m.g|	arrdc4.S	ARRDC4	24.24	23.97	11.26	0.88	2.50E-15
D3	mi|Xelaev18020261m.g|	loc100496891.S	N/B	23.77	33.94	8.67	0.82	1.61E-11
D3	mi|Xelaev18038791m.g|	irs4.L	IRS2	23.71	35.31	6.77	0.91	4.20E-18
D3	mi|Xelaev18020988m.g|	hbp1.S	HBP1	23.63	46.69	19.71	0.89	3.17E-16
D3	mi|Xelaev18011592m.g|	col8a1.L	COL8A1	23.46	13.62	-8.88	0.72	3.66E-08
D3	mi|Xelaev18038396m.g|	tsc1.L	TSC1	23.43	42.94	25.52	0.90	2.42E-16
D3	mi|Xelaev18043626m.g|	Xelaev18043626m.g	ZNF704	23.36	46.66	26.05	0.86	4.38E-14
D3	mi|Xelaev18044241m.g|	wipi1.L	WIPI1	23.03	43.33	16.54	0.95	8.93E-23
D3	mi|Xelaev18046347m.g|	Xelaev18046347m.g	ZNF704	23.02	49.25	30.07	0.91	1.15E-17
D3	mi|Xelaev18005409m.g|	sap30.L	SAP30	22.93	57.28	24.76	0.79	1.98E-10
D3	mi|Xelaev18018045m.g|	hmg20a.L	HMG20A	22.88	34.75	3.96	0.94	1.02E-20
D3	mi|Xelaev18045263m.g|	gpr146.L	GPR146	22.68	32.28	1.66	0.81	4.15E-11
D3	mi|Xelaev18018052m.g|	loc100158581.L	PTPN9	22.45	49.49	11.59	0.89	1.48E-15
D3	mi|Xelaev18032982m.g|	pdk4.S	N/B	22.42	17.93	-5.73	0.90	1.36E-16
D3	mi|Xelaev18002495m.g|	Xelaev18002495m.g	N/B	22.29	35.36	6.43	0.90	1.54E-16
D3	mi|Xelaev18023830m.g|	slc38a3.L	SLC38A3	21.85	17.45	-7.38	0.84	7.42E-13
D3	mi|Xelaev18025657m.g|	nptxr.S	NPTXR	21.51	27.15	-11.25	0.76	1.66E-09
D3	mi|Xelaev18022688m.g|	kiaa0513.L	KIAA0513	21.50	30.12	-6.80	0.85	2.24E-13
D3	mi|Xelaev18031888m.g|	adcyap1.L	ADCYAP1	21.39	31.18	-2.51	0.80	4.56E-11
D3	mi|Xelaev18044167m.g|	foxk2.L	FOXK2	21.28	38.47	22.31	0.90	1.44E-16
D3	mi|Xelaev18039311m.g|	loc100487262.L	N/B	21.15	26.45	0.15	0.83	3.04E-12
D3	mi|Xelaev18018365m.g|	acan.L	ACAN	21.05	34.93	-1.70	0.74	1.29E-08
D3	mi|Xelaev18037914m.g|	cdknx.S	CDKN1B	20.62	39.79	14.10	0.88	1.79E-15
D3	mi|Xelaev18003642m.g|	htr1a.S	HTR1A	20.24	26.59	-8.77	0.84	1.38E-12
D3	mi|Xelaev18014688m.g|	csde1.S	CSDE1	20.16	12.74	-10.16	0.80	8.18E-11

D3	mi|Xelaev18013763m.g|	mtif3.L	MTIF3	20.07	23.45	2.77	0.90	2.02E-16
D3	mi|Xelaev18033616m.g|	zbtb14.S	ZBTB14	20.03	30.84	1.22	0.72	3.34E-08
D3	mi|Xelaev18005685m.g|	pde5a.L	N/B	19.97	10.97	-4.45	0.82	1.38E-11
D3	mi|Xelaev18038639m.g|	acrc.L	GCNA	19.90	25.66	-1.42	0.78	4.45E-10
D3	mi|Xelaev18012702m.g|	syap1.L	SYAP1	19.90	18.03	3.76	0.80	6.47E-11
D3	mi|Xelaev18041751m.g|	itih6.S	ITIH6	19.55	40.20	14.43	0.88	1.67E-15
D3	mi|Xelaev18022580m.g|	nudt7.L	NUDT7	19.30	30.33	-3.87	0.72	3.24E-08
D3	mi|Xelaev18045205m.g|	syt17.L	SYT17	19.26	29.63	3.80	0.90	1.75E-16
D3	mi|Xelaev18025281m.g|	zcchc11.S	TUT4	19.25	20.76	1.49	0.87	1.83E-14
D3	mi|Xelaev18025743m.g|	MGC75753.S	BTG1	18.96	42.23	15.99	0.83	2.09E-12
D3	mi|Xelaev18018799m.g|	vps41.L	VPS41	18.80	13.59	2.30	0.75	4.03E-09
D3	mi|Xelaev18028490m.g|	loc100491791.S	N/B	18.62	12.40	-6.18	0.82	6.00E-12
D3	mi|Xelaev18028620m.g|	adck3.S	COQ8A	18.61	30.05	2.78	0.77	1.24E-09
D3	mi|Xelaev18007577m.g|	pik3ip1.L	PIK3IP1	18.60	14.48	-0.28	0.87	1.54E-14
D3	mi|Xelaev18014789m.g|	tbl1x.S	TBL1X	18.59	18.20	-9.98	0.88	4.83E-15
D3	mi|Xelaev18018209m.g|	scg3.L	SCG3	18.53	21.50	-9.08	0.87	1.08E-14
D3	mi|Xelaev18033741m.g|	tox.S	TOX	18.34	30.57	0.02	0.85	3.91E-13
D3	mi|Xelaev18003535m.g|	mecp2.L	MECP2	18.33	29.49	10.11	0.87	1.85E-14
D3	mi|Xelaev18041885m.g|	Xelaev18041885m.g	NT5DC2	18.14	45.21	25.51	0.87	9.94E-15
D3	mi|Xelaev18020639m.g|	fam214a.S	FAM214A	17.98	34.75	5.80	0.81	3.32E-11
D3	mi|Xelaev18034748m.g|	Xelaev18034748m.g	IFIT5	17.75	27.43	10.55	0.83	3.01E-12
D3	mi|Xelaev18013532m.g|	rapgef3.L	RAPGEF3	17.02	29.17	15.90	0.79	1.25E-10
D3	mi|Xelaev18046827m.g|	ndel1.S	NDEL1	16.96	31.19	-1.39	0.85	2.38E-13
D3	mi|Xelaev18038289m.g|	dbh.L	DBH	16.84	32.85	2.93	0.86	4.71E-14
D3	mi|Xelaev18003720m.g|	mgc108213.L	CYP46A1	16.62	34.77	13.38	0.78	3.09E-10
D3	mi|Xelaev18040384m.g|	Xelaev18040384m.g	N/B	16.59	49.20	17.41	0.96	2.60E-24

D3	mi|Xelaev18037641m.g|	Xelaev18037641m.g	CRYAB	16.25	33.37	3.97	0.82	1.38E-11
D3	mi|Xelaev18014071m.g|	loc100496869.L	NEU3	16.01	25.11	-0.17	0.90	6.70E-17
D3	mi|Xelaev18000174m.g|	irx5.1.L	IRX5	15.98	46.23	12.07	0.92	2.85E-18
D3	mi|Xelaev18025667m.g|	tob2.S	N/B	15.72	31.21	-0.45	0.91	1.05E-17
D3	mi|Xelaev18014618m.g|	hip1.S	HIP1	15.71	46.77	20.23	0.73	1.81E-08
D3	mi|Xelaev18037639m.g|	Xelaev18037639m.g	CRYAB	15.57	53.12	30.15	0.90	2.49E-16
D3	mi|Xelaev18013073m.g|	loc100498409-like.L	IRS2	15.52	29.58	10.86	0.94	1.01E-20
D3	mi|Xelaev18026142m.g|	epas1.L	EPAS1	15.27	42.19	23.28	0.84	7.58E-13
D3	mi|Xelaev18011170m.g|	loc101732034.S	N/B	15.23	24.27	-3.12	0.72	4.36E-08
D3	mi|Xelaev18024417m.g|	Xelaev18024417m.g	CSTF3	15.22	38.09	19.48	0.72	3.98E-08
D3	mi|Xelaev18019886m.g|	slc6a12.S	SLC6A12	15.16	25.33	1.03	0.71	9.03E-08
D3	mi|Xelaev18005367m.g|	casp3.L	N/B	15.15	35.18	15.91	0.88	2.51E-15
D3	mi|Xelaev18015346m.g|	rph3al.S	RPH3AL	15.09	29.42	1.91	0.64	3.51E-06
D3	mi|Xelaev18047038m.g|	cdk5r2.S	CDK5R2	15.02	18.10	-13.31	0.63	5.41E-06
D3	mi|Xelaev18002260m.g|	sgf29.S	SGF29	14.98	35.74	8.56	0.81	1.67E-11
D3	mi|Xelaev18039949m.g|	cfl2.L	CFL2	14.94	34.01	12.41	0.89	6.51E-16
D3	mi|Xelaev18041618m.g|	uck1.S	UCK1	14.80	30.13	2.05	0.91	1.72E-17
D3	mi|Xelaev18036619m.g|	pdcd4.S	PDCD4	14.80	34.94	16.15	0.93	9.62E-20
D3	mi|Xelaev18003908m.g|	nt5c2.L	NT5C2	14.68	30.08	1.85	0.74	1.25E-08
D3	mi|Xelaev18028669m.g|	eprs-like.S	EPRS	14.64	24.65	1.34	0.82	1.03E-11
D3	mi|Xelaev18032944m.g|	nmt1.S	NMT2	14.61	23.54	-1.87	0.71	7.91E-08
D3	mi|Xelaev18016009m.g|	znf385a.S	ZNF385A	14.33	37.39	13.74	0.72	4.90E-08
D3	mi|Xelaev18005853m.g|	Xelaev18005853m.g	CCDC158	13.85	10.11	-10.23	0.76	1.59E-09
D3	mi|Xelaev18020110m.g|	map2k7.S	MAP2K7	13.82	32.53	8.83	0.77	8.40E-10
D3	mi|Xelaev18024931m.g|	atp2c2.S	ATP2C2	13.78	12.26	-15.17	0.78	4.07E-10
D3	mi|Xelaev18024130m.g|	magi1.L	N/B	13.75	19.86	-5.52	0.74	1.21E-08

D3	mi|Xelaev18032047m.g|	tox.L	TOX	13.49	34.00	2.13	0.92	6.95E-19
D3	mi|Xelaev18018185m.g|	ccpg1.L	CCPG1	13.22	25.91	6.91	0.86	9.25E-14
D3	mi|Xelaev18035404m.g|	foxr1.L	TTC36	13.20	43.62	10.65	0.94	6.49E-21
D3	mi|Xelaev18012516m.g|	meaf6.L	MEAF6	13.18	26.29	-0.26	0.83	4.09E-12
D3	mi|Xelaev18031585m.g|	loc100145507.L	ACD	13.14	34.80	3.08	0.85	4.81E-13
D3	mi|Xelaev18017257m.g|	dpysl3.L	DPYSL3	13.11	13.86	-11.82	0.79	1.99E-10
D3	mi|Xelaev18047017m.g|	ptprn.S	PTPRN	13.11	26.07	-2.01	0.62	8.04E-06
D3	mi|Xelaev18040156m.g|	max.L	MAX	13.10	33.22	13.89	0.84	1.12E-12
D3	mi|Xelaev18030676m.g|	shh.L	SHH	13.08	16.51	-14.79	0.86	5.65E-14
D3	mi|Xelaev18004576m.g|	loc100486709.1.L	ZNF71	13.06	29.83	2.36	0.74	7.96E-09
D3	mi|Xelaev18024114m.g|	tmf1.L	TMF1	13.05	46.91	20.09	0.90	5.87E-17
D3	mi|Xelaev18024949m.g|	irf8.S	IRF8	13.02	25.02	0.04	0.82	8.01E-12
D3	mi|Xelaev18009309m.g|	larp1b.S	LARP1B	12.98	22.52	0.30	0.80	4.64E-11
D3	mi|Xelaev18009937m.g|	hcn2.S	HCN2	12.96	20.20	-3.44	0.85	4.20E-13
D3	mi|Xelaev18043235m.g|	calcoco2.L	CALCOCO2	12.94	32.74	7.40	0.87	2.69E-14
D3	mi|Xelaev18011934m.g|	hip1.L	HIP1	12.92	24.73	-3.99	0.71	5.96E-08
D3	mi|Xelaev18008037m.g|	pam.L	PAM	12.87	34.05	4.87	0.75	3.21E-09
D3	mi|Xelaev18039731m.g|	zfyve21.L	ZFYVE21	12.84	30.48	0.70	0.83	3.83E-12
D3	mi|Xelaev18021408m.g|	dpysl3.S	DPYSL3	12.78	24.74	-4.41	0.90	1.27E-16
D3	mi|Xelaev18008363m.g|	slc1a3.L	SLC1A3	12.64	16.55	-11.63	0.86	7.06E-14
D3	mi|Xelaev18026445m.g|	Xelaev18026445m.g	GNMT	12.63	28.03	3.32	0.89	1.50E-15
D3	mi|Xelaev18040152m.g|	gphn.L	GPHN	12.51	16.79	-9.36	0.73	1.97E-08
D3	mi|Xelaev18036996m.g|	rufy2.S	RUFY2	12.48	22.01	-4.65	0.82	1.57E-11
D3	mi|Xelaev18008873m.g|	nelfa.S	NELFA	12.43	37.28	13.45	0.90	5.35E-17
D3	mi|Xelaev18046783m.g|	foxk2.S	FOXK2	12.43	48.43	28.03	0.90	7.61E-17
D3	mi|Xelaev18027812m.g|	atg4b.L	ATG4B	12.36	27.46	2.53	0.77	1.48E-09

D3	mi|Xelaev18018816m.g|	ddhd2.L	DDHD2	12.35	32.94	6.34	0.73	2.10E-08
D3	mi|Xelaev18015612m.g|	loc100498409-like.S	IRS2	12.32	26.27	17.61	0.87	2.91E-14
D3	mi|Xelaev18041778m.g|	mecp2.S	MECP2	12.23	38.90	13.27	0.93	4.39E-20
D3	mi|Xelaev18025898m.g|	Xelaev18025898m.g	MGLL	12.08	20.05	-6.33	0.84	7.39E-13
D3	mi|Xelaev18041485m.g|	Xelaev18041485m.g	GLUL	11.99	27.32	4.51	0.83	3.92E-12
D3	mi|Xelaev18021194m.g|	gns.S	GNS	11.98	27.30	1.03	0.73	1.47E-08
D3	mi|Xelaev18005777m.g|	ccser1.L	CCSER1	11.95	18.25	-8.40	0.85	2.16E-13
D3	mi|Xelaev18012818m.g|	cep97.L	CEP97	11.93	18.16	-6.29	0.77	1.10E-09
D3	mi|Xelaev18022000m.g|	ifitm1.L	IFITM3	11.92	32.02	12.66	0.87	2.32E-14
D3	mi|Xelaev18044773m.g|	ube2e3.L	UBE2E3	11.90	23.19	1.81	0.83	2.45E-12
D3	mi|Xelaev18003631m.g|	stau2.L	STAU2	11.88	15.08	-6.07	0.84	1.51E-12
D3	mi|Xelaev18042526m.g|	loc100487262.S	N/B	11.81	23.31	-2.23	0.77	1.04E-09
D3	mi|Xelaev18037002m.g|	herc4.S	HERC4	11.80	29.99	3.89	0.88	5.38E-15
D3	mi|Xelaev18011288m.g|	loc100490688.L	N/B	11.80	30.81	1.04	0.89	3.44E-16
D3	mi|Xelaev18031822m.g|	spire1.L	SPIRE1	11.48	10.19	-10.61	0.74	1.32E-08
D3	mi|Xelaev18002750m.g|	ranbp9.S	RANBP9	11.33	33.58	6.66	0.84	7.39E-13
D3	mi|Xelaev18035297m.g|	ei24.L	EI24	11.27	22.73	-0.62	0.84	1.09E-12
D3	mi|Xelaev18039269m.g|	unnamed.L	INAFM2	11.24	15.17	-4.23	0.85	1.50E-13
D3	mi|Xelaev18031909m.g|	cables1.L	CABLES1	11.17	25.74	1.05	0.83	1.99E-12
D3	mi|Xelaev18022689m.g|	fbxo31.L	FBXO31	11.15	23.69	-4.31	0.69	1.92E-07
D3	mi|Xelaev18009157m.g|	sap30.S	SAP30	11.09	22.20	-5.94	0.72	3.37E-08
D3	mi|Xelaev18038264m.g|	Xelaev18038264m.g	GLUL	11.05	23.37	7.52	0.87	2.88E-14
D3	mi|Xelaev18045282m.g|	Xelaev18045282m.g	PRKAR1B	11.05	22.66	5.77	0.80	1.16E-10
D3	mi|Xelaev18015145m.g|	c1orf216.S	C1orf216	11.03	23.72	0.13	0.93	1.52E-19
D3	mi|Xelaev18039066m.g|	rtn2.L	RTN2	11.02	26.95	-0.61	0.77	9.79E-10
D3	mi|Xelaev18009202m.g|	gucy1b3.S	GUCY1B1	11.01	11.25	-8.61	0.91	9.85E-18

D3	mi|Xelaev18044963m.g|	loc200726.L	FAM237A	10.96	12.04	-10.17	0.75	3.60E-09
D3	mi|Xelaev18018391m.g|	arrdc4.L	ARRDC4	10.91	15.03	-3.44	0.84	8.66E-13
D3	mi|Xelaev18006303m.g|	ranbp3.L	RANBP3	10.88	12.50	-14.69	0.82	1.39E-11
D3	mi|Xelaev18034012m.g|	loc549366.S	ADCY8	10.79	16.31	-7.36	0.91	1.71E-17
D3	mi|Xelaev18038329m.g|	col5a1-like.L	COL5A1	10.78	19.74	4.74	0.73	2.10E-08
D3	mi|Xelaev18012348m.g|	st7l.L	ST7	10.74	17.59	-8.52	0.66	9.27E-07
D3	mi|Xelaev18044582m.g|	galnt13.L	GALNT13	10.72	10.71	-12.17	0.81	2.35E-11
D3	mi|Xelaev18019772m.g|	mkrn1.S	MKRN1	10.70	31.65	11.76	0.79	2.43E-10
D3	mi|Xelaev18030834m.g|	olah.L	OLAH	10.69	15.90	-11.31	0.83	3.24E-12
D3	mi|Xelaev18022646m.g|	crispld2.L	CRISPLD2	10.68	12.51	-11.79	0.76	2.62E-09
D3	mi|Xelaev18021032m.g|	mapk11.S	MAPK11	10.60	17.76	-6.45	0.74	1.26E-08
D3	mi|Xelaev18016996m.g|	apbb3.L	APBB3	10.54	10.42	-10.73	0.77	1.26E-09
D3	mi|Xelaev18042435m.g|	agpat1.S	AGPAT1	10.51	23.51	-4.95	0.91	1.62E-17
D3	mi|Xelaev18034639m.g|	calhm2.L	CALHM2	10.44	15.00	-7.98	0.70	1.59E-07
D3	mi|Xelaev18018604m.g|	vamp5.L	N/B	10.42	15.34	-6.91	0.81	1.94E-11
D3	mi|Xelaev18029866m.g|	scg2.S	SCG2	10.41	21.15	-5.78	0.78	3.83E-10
D3	mi|Xelaev18035814m.g|	tprg1l.L	TPRG1L	10.29	16.32	-3.52	0.81	3.83E-11
D3	mi|Xelaev18036859m.g|	bnip3.S	BNIP3	10.27	37.21	13.96	0.82	6.69E-12
D3	mi|Xelaev18024381m.g|	ccdc34.S	CCDC34	10.19	41.47	14.92	0.84	1.01E-12
D3	mi|Xelaev18005303m.g|	aasdh.L	AASDH	10.18	12.43	-9.87	0.71	5.89E-08
D3	mi|Xelaev18030216m.g|	pnoc.S	PNOC	10.01	26.21	1.77	0.70	9.57E-08
N/C	mi|Xelaev18037618m.g|	uts2.S	N/B	34.97	37.09	-6.82	0.69	1.75E-07
N/C	mi|Xelaev18033631m.g|	adcyap1.S	N/B	34.14	39.12	-6.50	0.36	1.66E-02
N/C	mi|Xelaev18000856m.g|	vgf.S	SPRR3	33.74	52.38	8.88	0.68	4.28E-07
N/C	mi|Xelaev18044679m.g|	gad1.1.L	GAD1	32.38	16.99	-10.61	0.59	3.00E-05
N/C	mi|Xelaev18021983m.g|	bdnf.L	BDNF	31.66	51.25	0.35	0.29	5.67E-02

N/C	mi|Xelaev18019171m.g|	vgf.L	SPRR3	30.33	52.84	5.22	0.53	2.01E-04
N/C	mi|Xelaev18041279m.g|	atg2b.S	ATG2B	29.88	30.97	10.73	0.86	9.00E-14
N/C	mi|Xelaev18014203m.g|	tax1bp3.S	TAX1BP3	28.29	22.81	-7.68	-0.64	3.34E-06
N/C	mi|Xelaev18026850m.g|	Xelaev18026850m.g	N/B	27.61	30.71	-6.56	0.60	1.79E-05
N/C	mi|Xelaev18024853m.g|	rnf166.S	RNF166	27.22	34.24	8.89	0.79	1.28E-10
N/C	mi|Xelaev18025274m.g|	rab3b.S	RAB3C	27.09	17.94	-11.25	0.40	6.88E-03
N/C	mi|Xelaev18031550m.g|	kdsr.L	KDSR	26.87	23.44	4.79	0.69	2.60E-07
N/C	mi|Xelaev18029077m.g|	pde10a.S	PDE10A	26.62	38.02	3.50	0.44	2.76E-03
N/C	mi|Xelaev18027491m.g|	uts2b.L	UTS2B	26.21	18.26	-3.91	0.50	5.45E-04
N/C	mi|Xelaev18042546m.g|	tgfb1.S	TGFB1	26.04	21.86	-9.96	-0.60	1.93E-05
N/C	mi|Xelaev18010396m.g|	ube3b.S	UBE3B	25.44	38.83	14.12	0.89	1.30E-15
N/C	mi|Xelaev18027794m.g|	scg2.L	SCG2	25.31	27.36	-11.66	0.60	1.53E-05
N/C	mi|Xelaev18041090m.g|	ltk.S	ALK	25.08	33.55	-11.84	0.55	9.46E-05
N/C	mi|Xelaev18012481m.g|	ptpru.L	N/B	23.89	32.56	1.23	0.81	1.97E-11
N/C	mi|Xelaev18026851m.g|	pde10a.L	PDE10A	23.78	30.27	-1.54	0.56	9.16E-05
N/C	mi|Xelaev18017782m.g|	lrrc17.L	LRRC17	22.99	35.67	-2.70	0.07	6.31E-01
N/C	mi|Xelaev18009174m.g|	loc100488610.S	TRIM63	22.67	15.07	-9.53	0.61	1.19E-05
N/C	mi|Xelaev18045869m.g|	fkbp10.S	FKBP10	22.59	30.22	-0.62	-0.81	3.49E-11
N/C	mi|Xelaev18045012m.g|	slc40a1.L	N/B	22.44	28.86	0.92	0.70	1.54E-07
N/C	mi|Xelaev18024627m.g|	calca.S	N/B	22.28	36.02	0.96	0.42	4.23E-03
N/C	mi|Xelaev18024895m.g|	tat.S	TAT	22.14	53.86	16.01	0.17	2.70E-01
N/C	mi|Xelaev18010810m.g|	pcsk1.S	PCSK1	21.68	29.00	-3.01	0.82	1.15E-11
N/C	mi|Xelaev18012734m.g|	fam101b.L	RFLNB	21.62	41.30	-1.67	-0.57	6.35E-05
N/C	mi|Xelaev18047821m.g|	rhbdl1.S	RHBDL1	21.37	19.92	-5.51	0.76	1.54E-09
N/C	mi|Xelaev18025674m.g|	Xelaev18025674m.g	CYP2D6	20.94	41.38	7.05	0.50	5.46E-04
N/C	mi|Xelaev18025227m.g|	pik3r3.S	PIK3R3	20.90	29.25	-5.82	-0.38	1.02E-02

N/C	mi|Xelaev18039619m.g|	l2hgdh.L	L2HGDH	20.57	23.77	-7.67	0.63	4.90E-06
N/C	mi|Xelaev18033026m.g|	hdac9.S	HDAC9	20.45	33.97	-5.97	0.49	7.07E-04
N/C	mi|Xelaev18019340m.g|	Xelaev18019340m.g	TNFSF13	20.32	19.13	-5.73	0.73	2.34E-08
N/C	mi|Xelaev18046721m.g|	rnf157.S	RNF157	20.14	24.78	-3.65	0.55	1.08E-04
N/C	mi|Xelaev18012753m.g|	ccl42c.L	CCL4L1	19.87	18.37	-9.46	-0.44	2.71E-03
N/C	mi|Xelaev18043779m.g|	Xelaev18043779m.g	N/B	19.80	28.86	-0.38	0.66	8.65E-07
N/C	mi|Xelaev18017537m.g|	mdm1.L	N/B	19.53	30.99	-1.13	0.66	8.75E-07
N/C	mi|Xelaev18047374m.g|	gpr1.S	GPR1	19.53	17.00	0.00	0.51	4.36E-04
N/C	mi|Xelaev18035721m.g|	loc100490200.L	MS4A4A	19.50	33.09	15.16	0.45	2.45E-03
N/C	mi|Xelaev18004059m.g|	gale.S	GALE	19.40	22.88	-0.02	-0.59	2.41E-05
N/C	mi|Xelaev18005353m.g|	cfap97.L	CFAP97	19.24	29.60	-3.24	0.67	7.17E-07
N/C	mi|Xelaev18023083m.g|	tesk2.L	TESK2	19.18	13.61	2.36	0.56	8.38E-05
N/C	mi|Xelaev18029914m.g|	klhl24.S	KLHL24	18.88	35.60	10.42	0.82	9.76E-12
N/C	mi|Xelaev18031024m.g|	galnt15.L	GALNT15	18.73	27.79	-1.70	0.45	2.28E-03
N/C	mi|Xelaev18044419m.g|	Xelaev18044419m.g	N/B	18.56	15.58	-10.81	0.09	5.65E-01
N/C	mi|Xelaev18021912m.g|	abtb2.L	ABTB2	18.44	34.07	-7.45	0.51	3.54E-04
N/C	mi|Xelaev18025320m.g|	pappa2.S	PAPPA2	18.16	10.37	-8.89	0.68	3.35E-07
N/C	mi|Xelaev18015389m.g|	gtf2ird1.S	GTF2IRD1	18.12	23.10	2.48	0.75	6.63E-09
N/C	mi|Xelaev18017943m.g|	tymp.L	TYMP	18.09	10.69	-10.25	0.73	1.87E-08
N/C	mi|Xelaev18012650m.g|	rps6kb1.L	RPS6KB1	17.97	18.00	-9.67	-0.65	1.65E-06
N/C	mi|Xelaev18039744m.g|	inf2.L	INF2	17.63	14.00	-10.88	-0.66	1.23E-06
N/C	mi|Xelaev18019513m.g|	loc100485200.L	SPAG7	17.60	28.75	1.13	-0.67	6.74E-07
N/C	mi|Xelaev18030764m.g|	gad2.L	GAD2	17.48	10.01	-7.01	0.53	2.23E-04
N/C	mi|Xelaev18017207m.g|	prelid2.L	PRELID2	17.41	40.53	18.38	-0.65	1.84E-06
N/C	mi|Xelaev18035643m.g|	ankrd65.L	ANK3	17.02	11.77	-12.21	-0.49	7.81E-04
N/C	mi|Xelaev18025232m.g|	lrrc41.S	LRRC41	16.74	14.85	0.92	0.48	9.18E-04

N/C	mi|Xelaev18021270m.g|	mon2.S	MON2	16.52	37.33	12.17	0.82	1.16E-11
N/C	mi|Xelaev18009688m.g|	prdm8.S	PRDM8	16.15	29.41	-7.43	0.69	2.00E-07
N/C	mi|Xelaev18010938m.g|	srek1.S	SREK1	16.15	27.17	-2.32	0.69	2.38E-07
N/C	mi|Xelaev18006943m.g|	isca1.L	ISCA1	16.10	16.49	-6.03	0.61	9.71E-06
N/C	mi|Xelaev18046744m.g|	Xelaev18046744m.g	SEC14L1	15.94	23.97	2.01	0.72	4.28E-08
N/C	mi|Xelaev18045380m.g|	ears2.L	EARS2	15.83	22.89	-10.05	-0.39	9.16E-03
N/C	mi|Xelaev18044295m.g|	crlf3.L	N/B	15.65	13.72	-11.94	-0.66	1.29E-06
N/C	mi|Xelaev18047571m.g|	gpr146.S	GPR146	15.65	29.97	1.55	0.63	3.83E-06
N/C	mi|Xelaev18007536m.g|	prodh.L	PRODH	15.50	10.21	-7.01	0.49	7.31E-04
N/C	mi|Xelaev18029196m.g|	gja1.S	GJA1	15.34	16.74	-8.80	0.56	8.91E-05
N/C	mi|Xelaev18023116m.g|	pik3r3.L	PIK3R3	15.32	29.52	4.10	-0.38	1.02E-02
N/C	mi|Xelaev18005623m.g|	cyp2u1.L	CYP2U1	15.32	40.66	15.36	0.81	3.77E-11
N/C	mi|Xelaev18044478m.g|	ptprn.L	PTPRN	15.23	28.27	-3.70	0.56	7.50E-05
N/C	mi|Xelaev18009061m.g|	pdgfra.S	PDGFRA	15.16	42.21	15.77	0.29	5.67E-02
N/C	mi|Xelaev18033468m.g|	cdh20.S	CDH20	15.15	22.96	-9.35	0.71	8.94E-08
N/C	mi|Xelaev18044420m.g|	igfbp5.L	IGFBP5	15.05	19.18	-9.07	0.55	9.66E-05
N/C	mi|Xelaev18022519m.g|	cyld.L	CYLD	14.79	18.88	-9.17	0.48	8.53E-04
N/C	mi|Xelaev18028179m.g|	loc100490476.L	CYP2E1	14.50	25.22	3.55	0.51	4.34E-04
N/C	mi|Xelaev18042490m.g|	agbl5.S	AGBL5	14.48	40.55	16.09	0.69	2.89E-07
N/C	mi|Xelaev18039589m.g|	atg2b.L	ATG2B	14.42	31.84	11.39	0.76	2.47E-09
N/C	mi|Xelaev18013574m.g|	calcoco1.L	CALCOCO1	14.28	24.50	4.65	0.72	4.63E-08
N/C	mi|Xelaev18023821m.g|	rbm5.L	RBM5	14.28	12.85	-12.52	0.64	2.91E-06
N/C	mi|Xelaev18025679m.g|	sgsm3.S	SGSM3	13.98	23.92	-12.23	0.55	1.16E-04
N/C	mi|Xelaev18007949m.g|	megf10.L	MEGF10	13.88	12.57	-10.72	0.74	8.61E-09
N/C	mi|Xelaev18006473m.g|	ell.L	ELL	13.85	22.57	-6.63	0.76	3.15E-09
N/C	mi|Xelaev18000112m.g|	srsf10.L	SRSF10	13.69	40.52	11.38	-0.65	1.79E-06

N/C	mi|Xelaev18047074m.g|	acvr2a.S	ACVR2A	13.59	31.71	5.60	0.61	9.14E-06
N/C	mi|Xelaev18012160m.g|	srsf1.L	SRSF1	13.46	15.46	-9.18	-0.69	1.71E-07
N/C	mi|Xelaev18038608m.g|	brinp1.L	BRINP1	13.44	34.14	10.24	0.75	5.72E-09
N/C	mi|Xelaev18035487m.g|	sik2.L	SIK2	13.41	18.42	-13.30	0.31	4.38E-02
N/C	mi|Xelaev18036794m.g|	slc16a9.S	SLC16A9	13.41	28.88	4.49	-0.39	8.19E-03
N/C	mi|Xelaev18020935m.g|	bend7.S	BEND7	13.37	24.92	-3.56	0.68	4.57E-07
N/C	mi|Xelaev18040411m.g|	anp32e.L	ANP32E	13.08	21.05	1.37	-0.67	7.99E-07
N/C	mi|Xelaev18037778m.g|	nosip.S	NOSIP	13.04	21.11	-2.53	0.64	2.64E-06
N/C	mi|Xelaev18029630m.g|	tnk2.S	TNK2	12.88	10.65	-11.86	0.72	3.55E-08
N/C	mi|Xelaev18025082m.g|	Xelaev18025082m.g	N/B	12.86	10.30	-12.34	0.43	3.76E-03
N/C	mi|Xelaev18044491m.g|	fam134a.L	RETREG2	12.83	10.72	3.13	0.56	8.25E-05
N/C	mi|Xelaev18008583m.g|	kiaa1161.L	MYORG	12.75	10.11	-7.60	0.71	8.83E-08
N/C	mi|Xelaev18021962m.g|	tcp11l1.L	TCP11L1	12.75	40.01	7.12	0.65	1.65E-06
N/C	mi|Xelaev18020738m.g|	cpeb1.S	CPEB1	12.68	14.44	-5.41	0.50	4.75E-04
N/C	mi|Xelaev18034804m.g|	sorbs1-like.L	SORBS1	12.55	13.70	-8.53	0.57	5.38E-05
N/C	mi|Xelaev18020780m.g|	loxl1.S	LOXL1	12.33	25.81	-2.01	0.70	1.32E-07
N/C	mi|Xelaev18005717m.g|	slc9b2.L	SLC9B2	12.24	23.15	-2.07	0.60	1.53E-05
N/C	mi|Xelaev18023654m.g|	nptxr.L	NPTXR	12.24	16.19	-5.37	0.63	4.18E-06
N/C	mi|Xelaev18009631m.g|	lmnb2.S	N/B	12.22	21.29	-4.07	-0.64	3.30E-06
N/C	mi|Xelaev18024457m.g|	ccnd1.S	N/B	12.16	15.00	-8.58	-0.42	4.45E-03
N/C	mi|Xelaev18044942m.g|	Xelaev18044942m.g	FZD7	12.16	27.86	-1.85	0.49	7.67E-04
N/C	mi|Xelaev18024383m.g|	bdnf.S	BDNF	12.14	40.50	6.20	0.24	1.17E-01
N/C	mi|Xelaev18026765m.g|	c1orf131.L	C1orf131	12.11	31.92	5.71	-0.59	2.43E-05
N/C	mi|Xelaev18043763m.g|	ccdc57.L	CCDC57	12.00	33.37	17.22	0.62	8.78E-06
N/C	mi|Xelaev18002080m.g|	tmem100.S	TMEM100	11.96	25.99	-4.46	0.29	5.58E-02
N/C	mi|Xelaev18033864m.g|	tmem55a.S	PIP4P2	11.93	24.35	1.65	0.67	7.16E-07

N/C	mi|Xelaev18014958m.g|	klf15.2.S	KLF15	11.85	18.03	-6.15	0.72	4.53E-08
N/C	mi|Xelaev18040965m.g|	kcnj10.L	KCNJ10	11.79	28.48	12.22	-0.61	1.02E-05
N/C	mi|Xelaev18037363m.g|	sik2.S	SIK2	11.69	23.92	0.49	0.27	7.58E-02
N/C	mi|Xelaev18023501m.g|	Xelaev18023501m.g	PMM1	11.63	10.86	-6.77	0.54	1.65E-04
N/C	mi|Xelaev18007479m.g|	loc100493091.L	N/B	11.62	31.73	12.65	0.49	6.42E-04
N/C	mi|Xelaev18015803m.g|	nr4a1.S	NR4A1	11.57	17.90	-9.61	0.12	4.35E-01
N/C	mi|Xelaev18016415m.g|	serpinh1.S	SERPINH1	11.52	37.33	22.24	-0.39	8.12E-03
N/C	mi|Xelaev18011332m.g|	ildr2.L	ILDR2	11.51	19.02	-11.38	0.42	4.45E-03
N/C	mi|Xelaev18032906m.g|	msrb2.S	N/B	11.51	24.47	-1.66	0.59	2.19E-05
N/C	mi|Xelaev18035124m.g|	loc101731425.L	CENPQ	11.50	28.26	13.23	0.43	3.44E-03
N/C	mi|Xelaev18006194m.g|	mex3d.L	MEX3D	11.41	15.32	-0.93	-0.65	1.85E-06
N/C	mi|Xelaev18024009m.g|	slc26a6.L	SLC26A6	11.37	23.83	-1.68	0.60	1.44E-05
N/C	mi|Xelaev18046933m.g|	pdia5.S	PDIA5	11.36	15.78	-4.30	-0.62	5.79E-06
N/C	mi|Xelaev18007376m.g|	parp2.L	PARP2	11.32	12.80	-5.80	0.40	7.89E-03
N/C	mi|Xelaev18012623m.g|	Xelaev18012623m.g	VAV1	11.22	17.10	2.79	-0.48	8.72E-04
N/C	mi|Xelaev18025198m.g|	tesk2.S	TESK2	11.19	10.91	3.05	0.61	1.26E-05
N/C	mi|Xelaev18018232m.g|	shc4.L	SHC4	11.14	39.75	3.18	0.11	4.77E-01
N/C	mi|Xelaev18012198m.g|	dyrk3.L	DYRK3	11.13	23.99	1.10	0.63	5.13E-06
N/C	mi|Xelaev18039468m.g|	ift43.L	IFT43	11.12	16.17	-1.49	0.45	2.00E-03
N/C	mi|Xelaev18022661m.g|	mphosph6.L	MPHOSPH6	11.06	23.95	4.71	-0.63	4.62E-06
N/C	mi|Xelaev18036704m.g|	emg1.S	N/B	10.99	26.23	0.20	-0.72	2.78E-08
N/C	mi|Xelaev18010363m.g|	thoc5.S	THOC5	10.95	13.14	-3.72	0.69	2.01E-07
N/C	mi|Xelaev18017582m.g|	lrig3.L	LRIG3	10.69	10.57	-6.90	0.39	8.61E-03
N/C	mi|Xelaev18016973m.g|	loc100489356-like.L	FBXL3	10.67	12.68	-9.39	0.59	2.70E-05
N/C	mi|Xelaev18042541m.g|	cyp2c18.S	CYP2C8	10.66	22.06	-5.10	0.59	2.70E-05
N/C	mi|Xelaev18017340m.g|	brd8.L	BRD8	10.63	43.52	23.26	0.70	1.32E-07

N/C	mi|Xelaev18018281m.g|	efl1.L	EFL1	10.56	20.42	-3.86	-0.34	2.26E-02
N/C	mi|Xelaev18021645m.g|	cdkn2d.S	CDKN2D	10.55	15.72	-4.06	0.48	1.06E-03
N/C	mi|Xelaev18045261m.g|	c7orf50.L	C7orf50	10.47	19.32	-7.94	-0.54	1.79E-04
N/C	mi|Xelaev18036648m.g|	fam160b1.S	FAM160B1	10.29	29.88	12.86	0.64	2.34E-06
N/C	mi|Xelaev18005843m.g|	slc4a4.L	SLC4A4	10.28	11.36	-9.19	0.41	6.08E-03
N/C	mi|Xelaev18037093m.g|	p4ha1-like.1.S	P4HA1	10.20	24.68	-0.70	-0.51	3.51E-04
N/C	mi|Xelaev18026664m.g|	cdc42ep3.L	CDC42EP3	10.10	20.29	-5.13	-0.56	8.71E-05

MM Module Membership
p.MM statistical significance of MM
N/C non-clustered gene

Supplementary Table S3 - GO Enrichment results, Molecular function

Module	GO ID	GO Description	pvalue	Count	Genes	Gene Module Ratio	Go term Ratio	∑BIC	x-value	GO term cluster
E1	GO:0070412	R-SMAD binding	4.11E-09	6	fos.S, fos.L, loc100490871-like.L, loc100490871-like.S, jun.L, jun.S	0.13	0.09	241.38	2024.25	0
E1	GO:0001046	core promoter sequence-specific DNA binding	9.77E-08	8	fos.S, fos.L, loc100490871-like.L, loc100490871-like.S, egr1.L, myc.S, myc.L, klf10.S	0.17	0.03	248.07	1739.00	1
E1	GO:0001227	transcriptional repressor activity RNA polymerase II transcription regulatory region sequence-specific binding	1.33E-04	6	btg2.S, nfkbia.L, ets2.L, atf3.S, bhlhe40.L, hes1.L	0.13	0.01	111.27	431.22	2
E2	GO:0042979	ornithine decarboxylase regulator activity	4.60E-06	3	odc1.S, azin1.S, azin2.S	0.04	0.27	90.96	485.52	-1
E2	GO:0015057	thrombin-activated receptor activity	5.09E-04	2	Xelaev18010901m.g, Xelaev18008178m.g	0.03	0.18	36.40	119.86	-1
I1	GO:0048018	receptor ligand activity	3.96E-04	13	wnt5a.S, timp-1.L, manf.L, timp-1.S, ccl34.S, metrnl.S, sema3b.S, sfrp2.L, metrnl.L,	0.06	0.03	737.61	2509.35	0

					inhba.L, hbegf.S, grp.L, tnfrsf11b.S					
I1	GO:0005178	integrin binding	3.58E-04	9	itgb6.L, itgb1.L, s1pr5.L, itga2.L, kdr.L, sfrp2.L, egfl6.S, thbs1.S, actn1.S	0.04	0.04	526.24	1813.46	1
I1	GO:0004252	serine-type endopeptidase activity	1.80E-03	7	mmp13.S, prss23.S, mmp19.L, mst1.L, plat.L, loc100493411.S, prtn3.S	0.03	0.04	320.69	880.04	2
I3	GO:0030515	snoRNA binding	1.57E-13	11	nop58.L, nop58.S, diexf.L, tbl3.L, nop56.L, tsr1.L, nhp2.L, diexf.S, dkc1.L, pwp2.S, bms1.L	0.05	0.27	484.40	6201.67	0
I3	GO:0001094	TFIID-class transcription factor binding	3.90E-09	6	fbl.L, nop58.L, nop58.S, ruvbl1.S, ruvbl1.L, fbl.S	0.03	0.40	300.60	2527.55	1
I3	GO:0008173	RNA methyltransferase activity	1.42E-08	10	fbl.L, nop2.L, nsun2.S, ftsj3.L, prmt5.S, setd6.L, fbl.S, mettl13.S, thumpd3.L, Xelaev18019094m.g	0.04	0.11	306.13	2402.56	2
I3	GO:0004386	helicase activity	2.17E-07	13	eif4a1.L, ddx27.L, eif4a1.S, ruvbl1.S, ddx24.L, ruvbl1.L, dhx37.L, g3bp1.L, g3bp1.S, smarca5.L, dhx38.L, smarca5.S,	0.06	0.06	487.38	3248.15	3

					c10orf2.L					
I3	GO:0000049	tRNA binding	2.10E-06	8	Xelaev18012630m.g, xpot.S, farsa.S, nsun2.S, farsa.L, thumpd3.L, Xelaev18019094m.g, ssb.S	0.03	0.09	217.36	1233.98	4
I3	GO:0004004	ATP-dependent RNA helicase activity	2.51E-06	8	eif4a1.L, ddx27.L, eif4a1.S, ddx24.L, dhx37.L, g3bp1.L, g3bp1.S, dhx38.L	0.03	0.09	329.79	1847.19	5
I3	GO:0140101	catalytic activity acting on a tRNA	3.50E-06	11	farsa.S, nsun2.S, ftsj3.L, prmt5.S, pdcl3.L, farsa.L, sgpp2.L, setd6.L, mettl13.S, thumpd3.L, Xelaev18019094m.g	0.05	0.06	304.58	1661.76	6
I3	GO:0016671	oxidoreductase activity acting on a sulfur group of donors disulfide as acceptor	6.92E-05	4	dnajc10.S, txnl1.S, txnl1.L, pdia3.L	0.02	0.17	94.23	391.96	7
I3	GO:0140097	catalytic activity acting on DNA	2.36E-04	11	hmga2.S, ruvbl1.S, ruvbl1.L, ftsj3.L, g3bp1.L, g3bp1.S, dkc1.L, prmt5.S, smarca5.L, smarca5.S, c10orf2.L	0.05	0.04	362.63	1315.14	-1

I3	GO:0003729	mRNA binding	7.72E-04	12	Xelaev18012630m.g, eif4a1.L, eif4a1.S, g3bp1.L, g3bp1.S, nifk.S, syncrip.S, syncrip.L, ncbp2.L, kiaa0020.S, ssb.S, elavl1.S	0.05	0.03	424.33	1320.69	-1
D1	GO:0015081	sodium ion transmembrane transporter activity	4.09E-03	5	Xelaev18001815m.g, Xelaev18002572m.g, slc5a7.S, pkd2.L, slc5a7.L	0.05	0.02	140.65	335.97	0
D1	GO:0031493	nucleosomal histone binding	9.19E-04	2	uhrf2.L, uhrf2.S	0.02	0.20	49.97	151.75	-1
D1	GO:0005007	fibroblast growth factor-activated receptor activity	1.12E-03	2	fgfr3.S, fgfr2.S	0.02	0.18	60.50	178.51	-1
D2	GO:0070513	death domain binding	2.45E-05	3	dapl1.S, loc100489885.S, dapl1.L	0.04	0.18	44.00	202.83	0
D2	GO:0016791	phosphatase activity	1.11E-03	7	pfkfb1.S, ppp1r15a.S, ppip5k1.S, pfkfb4.L, ppp2cb.S, ctdsp2.L, ctdsp2.S	0.09	0.01	160.77	474.97	1
D2	GO:0070728	leucine binding	6.10E-04	2	sesn1.L, sesn1.S	0.02	0.18	52.32	168.19	-1

L1	GO:0051787	misfolded protein binding	4.75E-08	7	sdf2l1.L, dnajb9.S, dnajc3.L, hspa5.S, sdf2l1.S, sdf2.S, edem1.L	0.03	0.22	284.67	2084.69	0
L1	GO:0016757	transferase activity transferring glycosyl groups	2.31E-05	17	parp3.L, sdf2l1.L, qtrt1.S, mgat1.S, ppat.L, alg6.L, sdf2l1.S, sdf2.S, umps.L, Xelaev18039639m.g, man2a1.S, b3gnt5.S, alg2.L, c1galt1c1.L, edem1.L, chsy1.L, pdcl.S	0.06	0.04	544.40	2524.03	1
L1	GO:0044653	dextrin alpha-glucosidase activity	8.66E-03	2	man2a1.S, edem1.L	0.01	0.15	32.64	67.32	2
L1	GO:0008933	lytic transglycosylase activity	8.66E-03	2	man2a1.S, edem1.L	0.01	0.15	32.64	67.32	-1
L2	GO:0015197	peptide transporter activity	6.40E-08	11	kpna4.S, kpna2.L, ipo4.L, kpnb1.S, kpna4.L, slc15a2.L, kpnb1.L, kpna6.L, xpo6.S, xpo5.S, tfrc.L	0.07	0.06	281.95	2028.36	0
L2	GO:0043021	ribonucleoprotein complex binding	5.50E-07	11	srpra.L, ddx3x.L, srp68.S, dhx29.L, ddx3x.S, gcn1.S, gcn1.L, prmt7.L, pelo.L, mtif2.L, nvl.L	0.07	0.05	208.05	1302.34	1

L2	GO:0030331	estrogen receptor binding	4.54E-04	5	ctnnb1.S, src.S, dnmt1.S, src.L, pik3r2-like.S	0.03	0.05	106.24	355.12	2
L3	GO:0140097	catalytic activity acting on DNA	8.70E-09	16	prmt5.L, mcm6.2.S, zmcm3.L, fen1.S, mcm5.S, mcm2.S, aptx.L, mcm2.L, mcm4.L, mcm7.L, mcm4.S, mcm6.2.L, mcm5.L, nme4.S, dnmt1.L, mcm7.S	0.08	0.05	457.11	3684.51	0
L3	GO:0003688	DNA replication origin binding	1.62E-09	6	mcm5.S, mcm2.S, mcm2.L, mcm5.L, hspd1.S, orc6.L	0.03	0.40	162.87	1431.58	1
L3	GO:0008168	methyltransfe rase activity	1.90E-07	18	wdr4.S, ntmt1.S, Xelaev1803779 0m.g, Xelaev1803606 1m.g, prmt5.L, trmt61a.L, hemk1.L, wdr77.S, Xelaev1802423 4m.g, prmt3.S, Xelaev1802255 6m.g, mettl1.L, mettl21b.L, Xelaev1802256 0m.g, rbbp4.L, rbbp4.S, comt.L, dnmt1.L	0.09	0.04	374.31	2516.02	2

D3	GO:0005539	glycosaminoglycan binding	1.44E-04	10	shh.L, acan.L, col5a1-like.L, dpysl3.S, loc101731931.S, unnamed.L, dpysl3.L, pcsk1.L, crispld2.L, gns.S	0.06	0.03	174.92	671.83	0
D3	GO:0047485	protein N-terminus binding	2.26E-03	7	loc549366.S, tbl1x.L, mecp2.S, tbl1x.S, tsc1.L, sgf29.S, mecp2.L	0.04	0.03	128.08	338.88	1
D3	GO:1901981	phosphatidylinositol phosphate binding	9.47E-03	7	wipi1.L, cfl2.L, hcn2.S, unnamed.L, hip1.L, hip1.S, rph3al.S	0.04	0.02	105.89	214.30	2
D3	GO:0032051	clathrin light chain binding	3.01E-03	2	hip1.L, hip1.S	0.01	0.17	28.63	72.17	-1

Supplementary Table S4 - GO Enrichment results, Biological process

Module	GO ID	GO Description	pvalue	Count	Genes	Gene Module Ratio	GO term Ratio	∑BIC	x-value	GO term cluster
E1	GO:0048145	regulation of fibroblast proliferation	1.29E-11	10	fos.S, nfkbia.L, fos.L, loc100490871-like.L, loc100490871-like.S, myc.S, myc.L, itgb3.L, jun.L, jun.S	0.21	0.05	349.29	3803.15	0
E1	GO:0001890	placenta development	4.39E-08	9	ptgs2.L, adm.S, junb.S, junb.L, socs3.L, hes1.L, loc101731931.L, socs3.S, Xelaev18021648m.g	0.19	0.02	245.42	1805.67	1
E1	GO:0048661	positive regulation of smooth muscle cell proliferation	1.57E-07	7	ptgs2.L, egr1.L, myc.S, myc.L, itgb3.L, jun.L, jun.S	0.15	0.03	235.83	1604.50	2
E2	GO:0006596	polyamine biosynthetic process	8.14E-07	4	odc1.S, azin1.S, smox.S, azin2.S	0.05	0.17	107.32	653.55	0
E2	GO:0002685	regulation of leukocyte migration	4.49E-06	8	Xelaev18036272m.g, Xelaev18010901m.g, ptafr.L, ctgf.S, Xelaev18008178m.g, serpine1.L, thbs1.L, Xelaev18009501m.g	0.11	0.03	264.86	1416.42	1

E2	GO:0030193	regulation of blood coagulation	4.07E-06	7	Xelaev18010901m.g, abat.S, Xelaev18008178m.g, fam46b.L, abat.L, serpine1.L, thbs1.L	0.09	0.03	168.81	910.00	2
E2	GO:0042119	neutrophil activation	6.96E-06	5	Xelaev18036272m.g, Xelaev18010901m.g, ptafr.L, Xelaev18008178m.g, Xelaev18009501m.g	0.07	0.06	133.28	687.39	3
E2	GO:0071674	mononuclear cell migration	1.57E-05	5	Xelaev18036272m.g, ctgf.S, serpine1.L, thbs1.L, Xelaev18009501m.g	0.07	0.05	212.46	1020.74	4
E2	GO:0031669	cellular response to nutrient levels	4.61E-04	7	cdkn1a.L, atf3.L, srf.S, cyp27b1.L, angptl4.S, angptl4.L, nuak2.S	0.09	0.02	233.82	780.13	5
E2	GO:0045766	positive regulation of angiogenesis	3.46E-04	6	ecm1.L, angptl4.S, angptl4.L, serpine1.L, thbs1.L, Xelaev18009501m.g	0.08	0.02	265.20	917.99	6
E2	GO:2001236	regulation of extrinsic apoptotic signaling pathway	6.28E-03	5	Xelaev18034426m.g, atf3.L, g0s2.L, serpine1.L, thbs1.L	0.07	0.01	211.54	465.79	7

E2	GO:0034764	positive regulation of transmembrane transport	4.97E-03	5	odc1.S, azin1.S, Xelaev18043136m.g, ptafr.L, azin2.S	0.07	0.01	131.96	303.99	8
E2	GO:0070960	positive regulation of neutrophil mediated cytotoxicity	5.44E-04	2	Xelaev18010901m.g, Xelaev18008178m.g	0.03	0.18	36.40	118.81	9
E2	GO:0030168	platelet activation	1.33E-04	7	rap2b.L, Xelaev18010901m.g, srf.S, abat.S, Xelaev18008178m.g, actn1.L, abat.L	0.09	0.02	163.95	635.46	-1
I1	GO:0061028	establishment of endothelial barrier	6.69E-07	10	s1pr5.L, tnfrsf21.L, f2rl2.S, cldn5.L, jup.L, f2r.S, msn.S, myadm.S, msn.L, myadm.L	0.05	0.07	468.93	2895.51	0
I1	GO:0007229	integrin-mediated signaling pathway	4.61E-06	11	itgb6.L, itgb1.L, itga2.L, timp-1.L, timp-1.S, Xelaev18002841m.g, myh9.S, Xelaev18041763m.g, ccm2.S, myh9.L, ctnna1.S	0.05	0.05	683.37	3646.63	1

I1	GO:0050920	regulation of chemotaxis	1.53E-04	13	wnt5a.S, s1pr5.L, itga2.L, f2rl2.S, camk1g.L, kdr.L, mst1.L, f2r.S, sema3b.S, f3.L, thbs1.S, prtn3.S, smad3.L	0.06	0.03	606.87	2315.79	2
I1	GO:0030834	regulation of actin filament depolymerization	8.57E-05	7	f2rl2.S, myh9.S, f2r.S, msn.S, myh9.L, lima1.L, msn.L	0.03	0.06	330.18	1342.86	3
I1	GO:0006911	phagocytosis engulfment	8.99E-05	6	itga2.L, f2rl2.S, myh9.S, f2r.S, myh9.L, thbs1.S	0.03	0.08	305.20	1234.88	4
I1	GO:1902904	negative regulation of supramolecular fiber organization	1.84E-03	9	s1pr5.L, myh9.S, msn.S, myh9.L, myadm.S, lima1.L, msn.L, mid1.L, myadm.L	0.04	0.03	455.53	1246.21	5
I2	GO:0042273	ribosomal large subunit biogenesis	3.23E-06	5	znf622.S, noc2l.L, rrp15.L, ppan.S, ppan.L	0.11	0.04	87.24	479.02	0
I2	GO:0022618	ribonucleoprotein complex assembly	1.22E-03	5	nufip1.L, ppan.S, eif3j.S, srsf10.S, ppan.L	0.11	0.01	99.75	290.78	1
I2	GO:0006364	rRNA processing	2.44E-04	6	utp15.L, wdr75.S, nop14.L, ddx54.L, naf1.L, rrp15.L	0.13	0.01	115.78	418.23	-1

I3	GO:0006364	rRNA processing	1.56E-29	43	fbl.L, utp4.L, nop58.L, nop58.S, mrto4.S, diexf.L, ddx27.L, tbl3.L, rsl1d1.L, wdr12.S, nop2.L, npm3.S, nop56.L, tsr1.L, aen.L, rsl1d1.S, dhx37.L, rrp1b.L, nhp2.L, ftsj3.L, rrs1.L, diexf.S, dkc1.L, gnl3.L, rrp1b.S, ngdn.L, riok2.S, rpf2.S, utp18.L, dcaf13.S, rexo4.S, mrto4.L, abt1.L, rrs1.S, bysl.S, fbl.S, bop1.L, thumpd1.S, dcaf13.L, pwp2.S, nolc1.L, bms1.L, mak16.S	0.18	0.10	1648.14	########	0
I3	GO:0009451	RNA modification	2.62E-13	18	fbl.L, nop58.L, nop58.S, pus1.S, nop2.L, nop56.L, nsun2.S, nhp2.L, ftsj3.L, syncrip.S, dkc1.L, syncrip.L, trmt6.S, fbl.S, thumpd1.S, thumpd3.L, Xelaev18019094m.g, ssb.S	0.08	0.10	692.41	8711.20	1

I3	GO:0000469	cleavage involved in rRNA processing	1.59E-09	9	npm3.S, tsr1.L, nhp2.L, rrs1.L, gnl3.L, abt1.L, rrs1.S, bop1.L, bms1.L	0.04	0.18	335.32	2950.27	2
I3	GO:0043044	ATP-dependent chromatin remodeling	3.89E-07	10	npm3.S, ruvbl1.S, ruvbl1.L, hdac1.L, Xelaev18047430m.g, Xelaev18045052m.g, smarca5.L, anp32c.S, hdac1.S, smarca5.S	0.04	0.08	350.97	2249.87	3
I3	GO:0022618	ribonucleoprotein complex assembly	8.78E-04	12	mrto4.S, npm3.S, tsr1.L, ruvbl1.S, ruvbl1.L, prmt5.S, brix1.L, rpf2.S, mrto4.L, abt1.L, pwp2.S, bms1.L	0.05	0.03	486.81	1488.02	4
D1	GO:0048754	branching morphogenesis of an epithelial tube	5.96E-05	10	npnt.L, fhdc1.S, egf.L, nfatc3.S, dlg5.L, fgfr2.S, nfatc3.L, pkd2.L, tmem67.S, sall2.S	0.10	0.02	244.20	1031.67	0
D1	GO:0031929	TOR signaling	2.83E-04	6	loc100493830-like.L, tsc2.S, mapkap1.S, sesn3.L, gatsl1.L, larp1b.L	0.06	0.03	121.71	431.87	1

D1	GO:2000371	regulation of DNA topoisomerase (ATP-hydrolyzing) activity	1.03E-03	2	uhrf2.L, uhrf2.S	0.02	0.18	49.97	149.27	2
D1	GO:1902275	regulation of chromatin organization	7.28E-03	6	lpin1.L, hdac5.L, lpin1.S, uhrf2.L, uhrf2.S, pkd2.L	0.06	0.02	199.99	427.54	-1
D2	GO:1902308	regulation of peptidyl-serine dephosphorylation	5.08E-04	2	ppp1r15a.L, ppp1r15a.S	0.03	0.20	42.57	140.21	0
D2	GO:0051384	response to glucocorticoid	5.19E-04	6	klf9.L, pfkfb1.S, cdo1.L, npas4.S, sgk3.L, sgk2.L	0.08	0.02	159.00	522.27	1
D2	GO:0043402	glucocorticoid mediated signaling pathway	6.20E-04	2	sgk3.L, sgk2.L	0.03	0.18	57.49	184.40	2
D2	GO:1901991	negative regulation of mitotic cell cycle phase transition	9.71E-03	5	ccng2.L, loc100489885.S, ctdsp2.L, MGC75753.L, ctdsp2.S	0.06	0.01	120.81	243.17	3
D2	GO:0010508	positive regulation of autophagy	5.04E-03	6	stk32a.L, tp53inp1.S, loc100494506-like.S, map1lc3a.S, ca5b.L, map1lc3b.S	0.08	0.01	124.89	286.95	4

L1	GO:0006364	rRNA processing	2.84E-11	25	Xelaev18003918m.g, heatr1.S, nhp2.S, wdr12.L, skiv2l2.S, utp15.S, npm3.L, rrp9.S, ddx49.S, utp6.L, exosc10.L, loc100491278-like.L, rrp9.L, wdr3.S, loc100490380-like.L, gar1.S, bop1.S, xrn2.S, shq1.L, ddx56.L, utp4.S, dimt1.L, fcf1.S, noc4l.L, riok2.L	0.10	0.06	749.32	7902.79	0
L1	GO:0009116	nucleoside metabolic process	1.89E-04	10	qtrt1.S, ppat.L, nme5.L, gmps.L, umps.L, ptger2.S, Xelaev18012070m.g, entpd7.L, nme9.L, pdcl.S	0.04	0.04	253.72	944.78	1
L1	GO:0048546	digestive tract morphogenesis	2.68E-03	8	stra6.L, ctnnb1.L, sfrp2.S, hsp90b1.L, wnt5a.L, stip1.L, sfrpx.S, jup.S	0.03	0.04	337.01	866.67	2

L2	GO:0006607	NLS-bearing protein import into nucleus	4.78E-10	8	kpna4.S, kpna2.L, ipo4.L, kpnb1.S, kpna4.L, kpnb1.L, nup35.L, kpna6.L	0.05	0.18	234.08	2181.68	0
L2	GO:0006364	rRNA processing	7.27E-04	10	rarg-like.2.L, nol11.S, utp20.L, wdr46.L, wdr36.L, rcl1.S, mdn1.L, nol8.L, exosc4.S, suv39h1.L	0.07	0.02	248.59	780.18	1
L2	GO:0017145	stem cell division	7.56E-05	7	wnt7b.L, fzd2.S, smad1.L, fzd2.L, tia1.L, pelo.L, dock6-like.L	0.05	0.04	188.62	777.36	2
L2	GO:0072089	stem cell proliferation	4.29E-03	8	ctnna1.L, wnt7b.L, nes.L, ctnnb1.S, fzd3.L, dync1h1.S, dock6-like.L, smarcd1.L	0.05	0.02	213.63	505.76	3
L2	GO:0006610	ribosomal protein import into nucleus	1.17E-04	3	ipo4.L, kpnb1.S, kpnb1.L	0.02	0.20	110.43	434.22	4
L2	GO:0007172	signal complex assembly	1.43E-04	3	pxn.S, src.S, src.L	0.02	0.19	81.66	313.92	5
L2	GO:0043550	regulation of lipid kinase activity	1.51E-04	6	rhog-like.1.S, src.S, src.L, rbl1.L, rbl1.S, pik3r2-like.S	0.04	0.05	124.99	477.47	6
L2	GO:0034446	substrate adhesion-dependent cell spreading	5.95E-03	6	rhog-like.1.S, pxn.S, src.S, src.L, fzd2.S, fzd2.L	0.04	0.02	181.62	404.18	7

L2	GO:0071680	response to indole-3-methanol	2.67E-03	2	ctnna1.L, ctnnb1.S	0.01	0.17	57.37	147.60	8
L2	GO:0090305	nucleic acid phosphodiester bond hydrolysis	5.67E-04	9	kpnb1.S, utp20.L, ern1.L, rcl1.S, kpnb1.L, hspa14.L, cpsf2.L, exosc4.S, pelo.L	0.06	0.03	185.13	601.08	-1
L2	GO:0002066	columnar/cuboidal epithelial cell development	3.62E-03	7	rhog-like.1.S, kpnb1.S, smad1.L, kpnb1.L, cdk4.S, dync1h1.S, sec24b.S	0.05	0.02	141.71	345.98	-1
L3	GO:0006270	DNA replication initiation	1.09E-16	14	mcm6.2.S, zmcm3.L, mcm5.S, mcm2.S, mcm2.L, mcm4.L, mcm7.L, mcm4.S, mcm6.2.L, mcm5.L, orc1.L, cdk2.S, mcm7.S, orc6.L	0.07	0.24	416.24	6643.63	0
L3	GO:0006611	protein export from nucleus	8.69E-06	13	chchd4.L, Xelaev18010463m.g, abce1.S, cpsf2.S, srsf2.L, eif6.S, xpo1.L, pcid2.S, Xelaev18019056m.g, chchd4.S, nup93.L, cse1l.L, srsf7.S	0.06	0.04	242.21	1225.75	1

L3	GO:0000077	DNA damage checkpoint	2.73E-05	13	Xelaev18037790m.g, Xelaev18036061m.g, stt3a.L, stt3a.S, polr2h.L, polr2f.S, mcm7.L, Xelaev18015757m.g, cdk2.S, e2f1.L, mcm7.S, e2f1.S, rpa2.S	0.06	0.04	250.17	1141.73	2
D3	GO:0032369	negative regulation of lipid transport	3.88E-06	6	hbp1.L, shh.L, loc100498409-like.L, loc100498409-like.S, irs4.L, hbp1.S	0.04	0.10	123.26	666.94	0
D3	GO:0042594	response to starvation	1.75E-04	11	Xelaev18002495m.g, pdk4.S, pdk4.L, slc38a3.L, slc18a2-like.2.S, Xelaev18038264m.g, Xelaev18026445m.g, Xelaev18041485m.g, max.L, adcyap1.L, atg4b.L	0.07	0.03	208.67	783.97	1
D3	GO:0042130	negative regulation of T cell proliferation	3.55E-04	5	casp3.L, shh.L, pde5a.L, unnamed.L, Xelaev18045282m.g	0.03	0.06	70.49	243.12	2

D3	GO:0050670	regulation of lymphocyte proliferation	2.15E-03	8	casp3.L, shh.L, loc100498409-like.L, loc100498409-like.S, irs4.L, pde5a.L, unnamed.L, Xelaev18045282m.g	0.05	0.02	122.04	325.62	3

Supplementary Table S5 - GO Enrichment results, Cellular Component

Module	GO ID	GO Description	pvalue	Count	Genes	Gene Module Ratio	GO term Ratio	∑BIC	x-value	GO term cluster
I1	GO:0031528	microvillus membrane	4.44E-10	8	itgb6.L, slc9a3r1.L, slc9a3r1.S, jup.L, msn.S, Xelaev18020683m.g, msn.L, Xelaev18018158m.g	0.04	0.24	378.42	3539.36	0
I1	GO:0030863	cortical cytoskeleton	2.47E-09	16	cdh2.L, Xelaev18002841m.g, myh9.S, cldn5.L, vcl.S, Xelaev18041763m.g, msn.S, pdlim7.L, sept2.S, myh9.L, myadm.S, actn1.S, Xelaev18020683m.g, msn.L, Xelaev18018158m.g, myadm.L	0.07	0.06	697.49	6003.55	1
I1	GO:0001726	ruffle	3.88E-05	14	itgb6.L, itgb1.L, rab34.L, myh9.S, msn.S, plekho1.S, pdlim7.L, myh9.L, myadm.S, snx5.L, actn1.S, msn.L, cyth1-like.S, myadm.L	0.06	0.03	669.58	2953.97	2
I1	GO:0005915	zonula adherens	5.70E-06	5	Xelaev18037758m.g, vcl.S, jup.L, actn1.S, ctnna1.S	0.02	0.17	220.16	1154.49	3
I1	GO:0032154	cleavage furrow	1.92E-04	7	itgb1.L, myh9.S, msn.S, sept2.S, myh9.L, lima1.L, msn.L	0.03	0.05	352.87	1311.33	4
I1	GO:0032432	actin filament bundle	1.36E-05	10	Xelaev18002841m.g, myh9.S, vcl.S, Xelaev18041763m.g, pdlim7.L, cnn1.L, myh9.L, cnn1.S, lima1.L, actn1.S	0.05	0.05	568.83	2769.07	5
I1	GO:0005884	actin filament	1.43E-04	10	Xelaev18002841m.g, vcl.S, Xelaev18041763m.g, pawr.S, dnajb9.L, pdlim7.L, actn1.S, Xelaev18020683m.g, Xelaev18018158m.g, Xelaev18026141m.g	0.05	0.04	337.98	1299.05	6

I1	GO:0031091	platelet alpha granule	7.19E-04	6	itgb6.L, timp-1.L, timp-1.S, loc100493411.S, thbs1.S, actn1.S	0.03	0.05	441.45	1387.57	7
I1	GO:0098862	cluster of actin-based cell projections	7.88E-07	15	slc9a3r1.L, homer2.S, slc9a3r1.S, Xelaev18002841m.g, myh9.S, vcl.S, Xelaev18041763m.g, msn.S, myh9.L, lima1.L, soat1.S, actn1.S, Xelaev18020683m.g, msn.L, Xelaev18018158m.g	0.07	0.04	632.36	3859.74	-1
I1	GO:0005604	basement membrane	4.47E-03	7	itgb1.L, timp-1.L, runx1.L, timp-1.S, runx1.S, egfl6.S, thbs1.S	0.03	0.03	487.56	1145.63	-1
I2	GO:0030684	preribosome	2.55E-07	6	znf622.S, nop14.L, noc2l.L, rrp15.L, ppan.S, ppan.L	0.12	0.05	111.26	733.48	0
I2	GO:0001725	stress fiber	4.38E-05	5	pdlim7.S, sept9.L, pdlim4.S, tpm4.S, tpm4.L	0.10	0.03	115.72	504.36	1
I2	GO:0030689	Noc complex	1.98E-04	2	nop14.L, noc2l.L	0.04	0.20	46.56	172.38	-1
I3	GO:0030684	preribosome	7.47E-29	27	fbl.L, nop58.L, nop58.S, mrto4.S, diexf.L, tbl3.L, rsl1d1.L, wdr12.S, nop56.L, tsr1.L, rsl1d1.S, rrp1b.L, ftsj3.L, rrs1.L, diexf.S, rrp1b.S, ngdn.L, utp18.L, dcaf13.S, mrto4.L, rrs1.S, fbl.S, bop1.L, dcaf13.L, pwp2.S, bms1.L, mak16.S	0.12	0.22	1086.21	30551.13	0
I3	GO:0044452	nucleolar part	2.66E-14	16	fbl.L, utp4.L, nop58.L, nop58.S, tbl3.L, wdr12.S, twistnb.L, npm3.S, nop56.L, nhp2.L, dkc1.L, utp18.L, fbl.S, bop1.L, cd3eap-like.L, pwp2.S	0.07	0.14	669.64	9089.94	1

I3	GO:0034708	methyltransferase complex	2.28E-04	9	ruvbl1.S, ruvbl1.L, wdr82.L, hdac1.L, prmt5.S, Xelaev18047430m.g, Xelaev18045052m.g, trmt6.S, hdac1.S	0.04	0.04	298.92	1088.71	2
I3	GO:0016363	nuclear matrix	9.59E-04	9	xpot.S, npm3.S, ruvbl1.S, ruvbl1.L, lmnb1.S, ppid.L, lmnb1.L, anp32c.S, prpf40a.S	0.04	0.04	306.16	924.04	3
I3	GO:1905368	peptidase complex	7.44E-03	6	wfs1.L, psme3.S, psme3.L, txnl1.S, txnl1.L, sec11a.L	0.03	0.03	173.06	368.31	4
I3	GO:1904949	ATPase complex	1.21E-04	9	abcf2.L, ruvbl1.S, ruvbl1.L, abcb6.L, hdac1.L, smarca5.L, hdac1.S, ino80b.S, smarca5.S	0.04	0.05	306.56	1201.25	-1
I3	GO:0070603	SWI/SNF superfamily-type complex	7.05E-04	7	ruvbl1.S, ruvbl1.L, hdac1.L, smarca5.L, hdac1.S, ino80b.S, smarca5.S	0.03	0.05	225.12	709.51	-1
D1	GO:1903349	omegasome membrane	1.12E-03	2	ulk2.L, ulk1.S	0.02	0.18	66.74	196.91	-1
D1	GO:1990462	omegasome	1.12E-03	2	ulk2.L, ulk1.S	0.02	0.18	66.74	196.91	-1
D1	GO:0044853	plasma membrane raft	8.14E-03	5	tsc2.S, lrp8.S, slc5a7.S, slc5a7.L, lipe.L	0.05	0.02	105.73	220.93	-1
L1	GO:0034663	endoplasmic reticulum chaperone complex	2.91E-12	9	sdf2l1.L, pdia6.L, hyou1.L, hspa5.S, hsp90b1.L, sdf2l1.S, pdia6.S, sdf2.S, hyou1.S	0.04	0.36	419.00	4833.44	0
L1	GO:0030684	preribosome	1.03E-07	11	Xelaev18003918m.g, heatr1.S, wdr12.L, nip7.L, rrp9.S, utp6.L, rrp9.L, wdr3.S, bop1.S, fcf1.S, noc4l.L	0.04	0.09	343.38	2399.20	1
L2	GO:0005643	nuclear pore	1.24E-06	8	kpna4.S, kpna2.L, ipo4.L, kpnb1.S, kpna4.L, kpnb1.L, nup35.L, kpna6.L	0.05	0.06	234.08	1382.53	0

L2	GO:0005875	microtubule associated complex	2.00E-03	9	pxn.S, prpf19.L, emc8.S, kpnb1.S, kif5b.S, kpnb1.L, bub3.L, dync1h1.S, bub3.S	0.06	0.02	190.53	514.21	1
L2	GO:0036464	cytoplasmic ribonucleoprotein granule	6.53E-03	7	rhog-like.1.S, mex3c.S, ddx3x.L, kpnb1.S, kpnb1.L, tia1.L, ddx3x.S	0.05	0.02	170.44	372.45	2
L2	GO:0005840	ribosome	1.22E-03	9	serp2.L, ddx3x.L, srp68.S, ddx3x.S, gcn1.S, hspa14.L, mrpl14.L, gcn1.L, metap1.L	0.06	0.02	194.65	567.44	-1
L3	GO:0000781	chromosome telomeric region	3.23E-13	18	stt3a.L, stt3a.S, mcm6.2.S, zmcm3.L, fen1.S, mcm5.S, mcm2.S, mcm2.L, mcm4.L, mcm7.L, mcm4.S, nhp2l1.S, mcm6.2.L, mcm5.L, orc1.L, cdk2.S, mcm7.S, rpa2.S	0.09	0.09	493.47	6163.59	0
L3	GO:0031970	organelle envelope lumen	1.84E-06	10	chchd4.L, ak2.L, ak2.S, ngfr.S, chchd4.S, pdia3.S, rexo2.L, nme4.S, ngfr.L, stoml2.L	0.05	0.06	216.05	1238.88	1
L3	GO:0034708	methyltransferase complex	9.07E-05	9	Xelaev18037790m.g, Xelaev18036061m.g, prmt5.L, trmt61a.L, wdr77.S, Xelaev18016354m.g, mettl1.L, rbbp4.L, rbbp4.S	0.04	0.04	215.42	870.84	2
L3	GO:0005788	endoplasmic reticulum lumen	6.18E-06	13	hsp90b1.S, ganab.L, ctsc.S, pdia4.L, txndc5.S, p4hb.L, gpx7.S, cln6.S, Xelaev18019056m.g, ganab.S, pdia3.S, uggt2.L, lepre1.L	0.06	0.04	324.48	1690.20	3
L3	GO:0030684	preribosome	7.47E-04	6	ebna1bp2.L, wdr36.S, nob1.S, eif6.S, nhp2l1.S, fcf1.L	0.03	0.05	128.23	400.90	4

L3	GO:0042470	melanosome	2.36E-03	7	hsp90b1.S, ganab.L, pdia4.L, p4hb.L, ganab.S, pdia3.S, itgb5.L	0.03	0.03	183.74	482.84	5
L3	GO:0042555	MCM complex	1.84E-19	11	mcm6.2.S, zmcm3.L, mcm5.S, mcm2.S, mcm2.L, mcm4.L, mcm7.L, mcm4.S, mcm6.2.L, mcm5.L, mcm7.S	0.05	0.65	363.57	6811.33	-1
L3	GO:0005657	replication fork	2.82E-03	5	stt3a.L, stt3a.S, zmcm3.L, dnmt1.L, rpa2.S	0.02	0.05	110.84	282.59	-1
D3	GO:0043679	axon terminus	1.10E-04	12	slc18a2-like.2.S, hcn2.S, dpysl3.S, Xelaev18038264m.g, rapgef3.L, dbh.L, dpysl3.L, Xelaev18041485m.g, adcyap1.L, pnoc.S, ptprn.S, calca.L	0.07	0.03	206.80	818.39	0
D3	GO:0000118	histone deacetylase complex	2.22E-03	5	tbl1x.L, tbl1x.S, hdac9.L, sap30.L, sap30.S	0.03	0.04	113.46	301.20	-1

Supplementary Table S6 - KEGG Pathway Enrichment results

Module	Path. ID	Path. Description	FDR	Count	Genes
E1	hsa04668	TNF signaling pathway	4.75E-06	7	JUNB, VEGFC, JUN, PTGS2, FOS, NFKBIA, SOCS3
E1	hsa04380	Osteoclast differentiation	2.81E-03	5	JUNB, JUN, FOS, NFKBIA, SOCS3
E1	hsa04657	IL-17 signaling pathway	5.36E-03	4	JUN, PTGS2, FOS, NFKBIA
E1	hsa05166	Human T-cell leukemia virus 1 infection	5.36E-03	6	ETS2, EGR1, JUN, MYC, FOS, NFKBIA
E1	hsa05140	Leishmaniasis	7.12E-03	4	JUN, PTGS2, FOS, NFKBIA
E1	hsa04010	MAPK signaling pathway	7.12E-03	6	VEGFC, DUSP6, JUN, MYC, DUSP5, FOS
E1	hsa05167	Kaposi sarcoma-associated herpesvirus infection	7.12E-03	5	JUN, PTGS2, MYC, FOS, NFKBIA
E1	hsa04210	Apoptosis	1.42E-02	4	TNFRSF10B, JUN, FOS, NFKBIA
E1	hsa04926	Relaxin signaling pathway	1.42E-02	4	VEGFC, JUN, FOS, NFKBIA
E1	hsa05224	Breast cancer	1.42E-02	4	JUN, HES1, MYC, FOS
E1	hsa04662	B cell receptor signaling pathway	1.96E-02	3	JUN, FOS, NFKBIA
E1	hsa05031	Amphetamine addiction	1.96E-02	3	JUN, ARC, FOS
E1	hsa05161	Hepatitis B	1.96E-02	4	JUN, MYC, FOS, NFKBIA
E1	hsa05210	Colorectal cancer	3.36E-02	3	JUN, MYC, FOS
E1	hsa05222	Small cell lung cancer	3.36E-02	3	PTGS2, MYC, NFKBIA
E1	hsa04620	Toll-like receptor signaling pathway	3.40E-02	3	JUN, FOS, NFKBIA
E1	hsa04625	C-type lectin receptor signaling pathway	3.45E-02	3	JUN, PTGS2, NFKBIA
E1	hsa04660	T cell receptor signaling pathway	3.50E-02	3	JUN, FOS, NFKBIA
E1	hsa05142	Chagas disease (American trypanosomiasis)	3.64E-02	3	JUN, FOS, NFKBIA
E1	hsa05169	Epstein-Barr virus infection	3.64E-02	4	JUN, HES1, MYC, NFKBIA
E1	hsa04933	AGE-RAGE signaling pathway in diabetic complications	3.64E-02	3	VEGFC, EGR1, JUN
E1	hsa04658	Th1 and Th2 cell differentiation	3.64E-02	3	JUN, FOS, NFKBIA
E2	hsa04115	p53 signaling pathway	1.80E-02	4	SERPINE1, GADD45G, CDKN1A, THBS1

E2	hsa04668	TNF signaling pathway	3.91E-02	4	LIF, BIRC2, IRF1, MAP3K14
E2	hsa04066	HIF-1 signaling pathway	3.91E-02	4	SERPINE1, CDKN1A, HK2, LTBR
I1	hsa04060	Cytokine-cytokine receptor interaction	5.87E-03	13	TNFRSF12A, IL11RA, LIF, CCL19, CCL13, CCL3L1, IL1R2, LEP, TNFRSF21, INHBA, TNFRSF11B, TNFRSF6B, IL21R
I1	hsa05205	Proteoglycans in cancer	7.52E-03	11	FLNA, MSN, KDR, ITGA2, ERBB3, HBEGF, ITGB1, FZD6, RRAS2, THBS1, MRAS
I1	hsa05412	Arrhythmogenic right ventricular cardiomyopathy (ARVC)	1.53E-02	6	ITGA2, ITGB1, CDH2, JUP, ITGB6, CACNG5
I1	hsa04530	Tight junction	4.51E-02	8	MSN, CLDN5, RUNX1, MYH9, ITGB1, ACTN1, SLC9A3R1, TUBA1A
I3	hsa03008	Ribosome biogenesis in eukaryotes	5.06E-11	12	NOP56, GNL3, RIOK2, PWP2, RBM28, UTP4, BMS1, FBL, DKC1, UTP18, NOP58, TBL3
D1	hsa04150	mTOR signaling pathway	1.19E-03	7	ULK1, ULK2, CASTOR2, LPIN1, DEPTOR, MAPKAP1, TSC2
D2	hsa04216	Ferroptosis	3.45E-02	3	MAP1LC3B, MAP1LC3A, SLC40A1
L1	hsa03008	Ribosome biogenesis in eukaryotes	2.16E-04	8	WDR3, RIOK2, GAR1, UTP15, UTP4, HEATR1, POP1, UTP6
L1	hsa00510	N-Glycan biosynthesis	1.99E-02	5	DOLPP1, MAN2A1, ALG6, ALG2, MGAT1
L1	hsa04141	Protein processing in endoplasmic reticulum	1.99E-02	9	HYOU1, DNAJC3, HSP90B1, CKAP4, EDEM1, PDIA6, DERL1, SEC24C, SEL1L
L2	hsa04141	Protein processing in endoplasmic reticulum	1.19E-02	8	TRAM1, SEC24B, UBE2G1, RPN1, UGGT1, STT3B, HSPA8, ERN1
L2	hsa04360	Axon guidance	1.19E-02	8	SRC, PIK3R2, SEMA3C, SEMA3B, FZD3, MYL12A, NTN1, PLXNB3
L2	hsa04550	Signaling pathways regulating pluripotency of stem cells	1.19E-02	7	SMAD1, PIK3R2, WNT7B, FZD3, CTNNB1, FZD2, RIF1
L2	hsa05205	Proteoglycans in cancer	2.29E-02	8	SRC, PIK3R2, PXN, WNT7B, FLNC, FZD3, CTNNB1, FZD2
L2	hsa00510	N-Glycan biosynthesis	2.29E-02	4	ALG8, ALG12, RPN1, STT3B
L2	hsa05100	Bacterial invasion of epithelial cells	2.29E-02	5	SRC, PIK3R2, PXN, CTNNB1, RHOG
L2	hsa05217	Basal cell carcinoma	4.72E-02	4	WNT7B, FZD3, CTNNB1, FZD2
L3	hsa03030	DNA replication	2.04E-05	7	MCM7, MCM5, MCM2, FEN1, MCM3, MCM4, MCM6
L3	hsa04141	Protein processing in endoplasmic reticulum	3.01E-05	12	MOGS, UGGT2, CALR, PDIA3, HSP90B1, CAPN1, SEC13, SSR3, PDIA4, DERL1, STT3A, HSPBP1
L3	hsa04110	Cell cycle	6.88E-04	9	MCM7, ORC6, ORC1, MCM5, MCM2, MCM3, MCM4, E2F1, MCM6

L3	hsa03008	Ribosome biogenesis in eukaryotes	2.50E-03	6	WDR36, NOB1, SNU13, XPO1, REXO2, EIF6
L3	hsa00510	N-Glycan biosynthesis	4.20E-03	5	MOGS, ALG5, ALG6, ALG3, STT3A

Supplementary Table S7 - Differential gene expression analysis of mTor Inhibition mRNA-seq

		DMSO vs Torin1 – 1 dpT		DMSO vs Torin1 – 2 dpT		DMSO vs Torin1 – 4 dpT	
**Xenopus ID**	**gene symbol**	**logFC**	**PValue**	**logFC**	**PValue**	**logFC**	**PValue**
mi|Xelaev18038036m.g|	Xelaev18038036m.g	-8.92	***0.00***	-2.66	***0.00***	-2.38	***0.00***
mi|Xelaev18036521m.g|	Xelaev18036521m.g	-8.49	***0.00***	-9.82	***0.00***	-5.58	0.04
mi|Xelaev18011418m.g|	loc101734578.L	-7.65	***0.00***	2.19	***0.01***	-0.37	0.67
mi|Xelaev18007944m.g|	Xelaev18007944m.g	-7.52	***0.00***	-7.18	***0.00***	0.00	1.00
mi|Xelaev18032480m.g|	loc100135371.L	-7.32	***0.00***	-8.35	***0.00***	0.07	1.00
mi|Xelaev18003376m.g|	igfals.1.L	-7.32	***0.00***	1.76	0.05	1.31	0.06
mi|Xelaev18022406m.g|	Xelaev18022406m.g	-7.04	***0.00***	0.85	0.48	-0.91	0.31
mi|Xelaev18013620m.g|	krt75.L	-6.97	***0.00***	-2.23	***0.00***	-2.21	***0.01***
mi|Xelaev18027033m.g|	adat2.L	-6.97	***0.00***	0.02	1.00	-0.50	0.52
mi|Xelaev18010286m.g|	Xetrov90002579m.S	-6.88	***0.00***	-1.34	0.15	-0.22	0.79
mi|Xelaev18027450m.g|	Xelaev18027450m.g	-6.84	***0.00***	-1.07	0.06	-1.39	***0.00***
mi|Xelaev18044444m.g|	slc4a3.L	-6.80	***0.00***	0.59	0.78	0.79	0.75
mi|Xelaev18046054m.g|	loc100488256.S	-6.71	***0.00***	0.02	1.00	-1.28	0.15
mi|Xelaev18035848m.g|	loc100493881.L	-6.26	***0.00***	-1.73	0.22	-2.85	***0.01***
mi|Xelaev18032354m.g|	Xelaev18032354m.g	-6.26	***0.00***	0.02	1.00	-0.24	1.00
mi|Xelaev18000879m.g|	loc100490626.S	-6.13	***0.01***	-1.85	0.05	-0.36	0.64
mi|Xelaev18026637m.g|	mal.L	-6.13	***0.01***	-1.94	0.06	-2.13	0.01
mi|Xelaev18025719m.g|	sstr3.S	-6.13	***0.01***	1.89	0.40	-1.22	0.24
mi|Xelaev18047255m.g|	Xetrov90024133m.S	-5.98	0.01	-2.41	***0.00***	-1.54	0.02
mi|Xelaev18035484m.g|	Xelaev18035484m.g	-5.81	0.02	-7.32	***0.00***	-6.30	***0.00***
mi|Xelaev18009847m.g|	loc100494822.S	-5.62	0.04	-2.69	***0.00***	-2.35	***0.00***
mi|Xelaev18014531m.g|	cldn4.S	-5.40	0.08	-0.07	1.00	-2.08	***0.00***
mi|Xelaev18040728m.g|	Xelaev18040728m.g	-5.30	0.08	-2.15	0.02	-3.00	***0.00***
mi|Xelaev18009818m.g|	Xelaev18009818m.g	-4.84	0.14	-6.70	***0.00***	-1.67	0.22
mi|Xelaev18015036m.g|	Xelaev18015036m.g	-4.84	0.14	-2.15	***0.01***	-1.90	0.02
mi|Xelaev18012142m.g|	Xelaev18012142m.g	-4.84	0.14	-0.75	0.27	-3.04	***0.00***
mi|Xelaev18036100m.g|	loc101731919.L	-4.84	0.14	-0.49	0.68	-4.52	***0.00***
mi|Xelaev18014725m.g|	Xelaev18014725m.g	-4.44	0.26	-3.22	***0.00***	-0.07	1.00
mi|Xelaev18042333m.g|	vgll1.S	-4.44	0.26	-3.63	***0.00***	-2.75	***0.01***
mi|Xelaev18012124m.g|	Xelaev18012124m.g	-4.44	0.26	-0.56	0.33	-2.25	***0.00***
mi|Xelaev18036123m.g|	loc100491292-like.L	-4.44	0.26	-0.15	1.00	-3.85	***0.00***
mi|Xelaev18045876m.g|	krt.S	-4.44	0.26	-5.35	0.08	-2.85	***0.01***
mi|Xelaev18034081m.g|	Xelaev18034081m.g	-4.25	***0.00***	-2.57	***0.00***	-2.92	***0.00***
mi|Xelaev18034496m.g|	Xelaev18034496m.g	-4.04	0.51	-6.81	***0.00***	4.68	0.26
mi|Xelaev18032437m.g|	loc100127564.L	-4.04	***0.00***	-1.24	0.02	-2.20	***0.00***
mi|Xelaev18004896m.g|	loc100487404-like.L	-3.98	***0.00***	-0.10	1.00	-0.27	0.75
mi|Xelaev18006283m.g|	Xelaev18006283m.g	-3.89	0.51	-7.46	***0.00***	0.79	0.75
mi|Xelaev18001560m.g|	Xelaev18001560m.g	-3.89	0.51	1.89	0.40	-6.56	***0.00***

mi|Xelaev18034079m.g|	Xelaev18034079m.g	-3.89	0.51	0.28	1.00	-1.60	***0.01***
mi|Xelaev18045677m.g|	eln2.L	-3.89	0.51	0.98	0.70	-4.77	***0.00***
mi|Xelaev18036933m.g|	Xelaev18036933m.g	-3.89	0.51	0.67	0.57	6.99	***0.00***
mi|Xelaev18035220m.g|	Xelaev18035220m.g	-3.78	***0.00***	-1.89	***0.00***	-2.77	***0.00***
mi|Xelaev18001215m.g|	trim14.S	-3.57	***0.01***	-2.53	***0.00***	-0.82	0.21
mi|Xelaev18028299m.g|	Xelaev18028299m.g	-3.56	***0.00***	1.07	0.06	-0.14	0.87
mi|Xelaev18016560m.g|	arfip2.S	-3.52	***0.01***	1.52	0.17	-0.81	0.38
mi|Xelaev18016847m.g|	ccdc69.L	-3.49	***0.01***	-0.14	0.90	-0.04	1.00
mi|Xelaev18036007m.g|	loc101730286.L	-3.41	1.00	-6.94	***0.00***	-0.31	1.00
mi|Xelaev18039251m.g|	Xelaev18039251m.g	-3.40	***0.00***	0.69	0.43	0.08	1.00
mi|Xelaev18045920m.g|	ikzf3.S	-3.31	***0.01***	-0.80	0.33	-0.65	0.40
mi|Xelaev18032441m.g|	loc101730807.L	-3.23	***0.00***	-0.19	0.73	-0.40	0.51
mi|Xelaev18023707m.g|	Xelaev18023707m.g	-3.21	***0.01***	0.73	0.75	-0.09	1.00
mi|Xelaev18012127m.g|	loc101732121.L	-3.10	0.01	-0.67	0.14	-1.88	***0.00***
mi|Xelaev18032566m.g|	Xelaev18032566m.g	-3.03	***0.00***	0.97	0.05	0.00	1.00
mi|Xelaev18039742m.g|	Xelaev18039742m.g	-2.98	1.00	-6.59	***0.00***	-1.90	***0.01***
mi|Xelaev18015051m.g|	loc101732121.S	-2.98	1.00	-1.42	0.06	-2.16	***0.01***
mi|Xelaev18031621m.g|	loc100488932.L	-2.98	1.00	0.00	1.00	-7.67	***0.00***
mi|Xelaev18019499m.g|	loc100491764.L	-2.98	1.00	1.55	0.33	-2.85	***0.01***
mi|Xelaev18029491m.g|	loc100497103.S	-2.98	1.00	4.10	0.51	-9.03	***0.00***
mi|Xelaev18047055m.g|	mcm6.2.S	-2.88	***0.00***	1.32	***0.00***	0.72	0.09
mi|Xelaev18031299m.g|	ccr3.L	-2.87	0.02	-2.67	***0.00***	-0.95	0.13
mi|Xelaev18018099m.g|	loc100497279-like.L	-2.87	0.02	-0.39	0.46	-3.13	***0.00***
mi|Xelaev18022083m.g|	loc100493937.L	-2.87	0.02	2.98	***0.00***	0.52	0.32
mi|Xelaev18020233m.g|	stc1.S	-2.86	***0.00***	-0.21	0.77	0.25	0.69
mi|Xelaev18036111m.g|	Xelaev18036111m.g	-2.86	***0.00***	-2.06	***0.00***	-2.56	***0.00***
mi|Xelaev18003356m.g|	Xelaev18003356m.g	-2.86	***0.00***	1.18	0.06	-0.50	0.47
mi|Xelaev18040474m.g|	loc100488012.L	-2.82	***0.00***	1.43	0.02	0.70	0.35
mi|Xelaev18011203m.g|	Xelaev18011203m.g	-2.76	***0.00***	-1.38	0.05	-0.67	0.31
mi|Xelaev18012479m.g|	laptm5.L	-2.76	***0.00***	-1.35	0.03	-0.20	0.84
mi|Xelaev18020745m.g|	kiaa0101.S	-2.75	***0.00***	0.48	0.26	0.19	0.66
mi|Xelaev18001333m.g|	Xelaev18001333m.g	-2.71	0.02	-2.69	0.04	-7.04	***0.00***
mi|Xelaev18029514m.g|	agpat5.S	-2.71	***0.00***	-0.20	0.81	0.69	0.34
mi|Xelaev18038344m.g|	phf19.L	-2.68	***0.00***	0.12	0.92	0.55	0.37
mi|Xelaev18044707m.g|	cdca7.L	-2.67	***0.00***	0.90	0.03	0.65	0.12
mi|Xelaev18017994m.g|	loc100496311-like.L	-2.64	0.01	-0.40	0.43	-1.77	***0.00***
mi|Xelaev18018083m.g|	kiaa0101.L	-2.62	***0.00***	0.53	0.21	-0.03	0.95
mi|Xelaev18035512m.g|	Xelaev18035512m.g	-2.58	***0.00***	-1.22	***0.01***	-0.75	0.09
mi|Xelaev18042611m.g|	suv39h1.S	-2.58	***0.00***	0.19	0.80	0.30	0.63
mi|Xelaev18027929m.g|	neu4.L	-2.56	***0.00***	-0.86	0.12	-0.80	0.17
mi|Xelaev18044721m.g|	Xelaev18044721m.g	-2.54	***0.00***	1.52	0.06	3.72	***0.00***
mi|Xelaev18006472m.g|	isyna1.L	-2.52	***0.00***	0.87	0.04	0.48	0.25
mi|Xelaev18023987m.g|	mcm2.L	-2.50	***0.00***	1.16	***0.01***	0.45	0.28

mi|Xelaev18016904m.g|	Xelaev18016904m.g	-2.48	***0.00***	-0.26	0.64	0.51	0.36
mi|Xelaev18047779m.g|	loc101733326.S	-2.47	0.03	-0.92	0.05	-2.34	***0.00***
mi|Xelaev18037685m.g|	loc100494984.S	-2.47	***0.00***	1.01	0.31	-0.05	1.00
mi|Xelaev18045526m.g|	Xelaev18045526m.g	-2.47	***0.00***	0.27	0.72	0.14	0.83
mi|Xelaev18045757m.g|	loc105948426.L	-2.45	***0.00***	-1.37	***0.00***	-0.69	0.11
mi|Xelaev18017570m.g|	e2f7.L	-2.45	***0.00***	0.48	0.29	-0.03	0.98
mi|Xelaev18013921m.g|	loc100491855-like.L	-2.45	***0.00***	0.55	0.44	-0.07	1.00
mi|Xelaev18021143m.g|	e2f7.S	-2.44	***0.00***	1.12	0.01	-0.06	0.95
mi|Xelaev18004500m.g|	pik3ap1.S	-2.43	***0.00***	-0.26	0.64	-1.68	***0.00***
mi|Xelaev18042718m.g|	Xelaev18042718m.g	-2.43	***0.01***	-1.06	0.10	-0.14	0.86
mi|Xelaev18046392m.g|	Xelaev18046392m.g	-2.43	***0.01***	-0.83	0.26	0.36	0.85
mi|Xelaev18047570m.g|	c7orf50.S	-2.41	***0.00***	1.58	***0.00***	-0.28	0.72
mi|Xelaev18028969m.g|	stx11.S	-2.41	***0.00***	0.09	0.95	-1.61	***0.01***
mi|Xelaev18013994m.g|	umod.2.L	-2.39	***0.00***	-0.99	0.02	-1.57	***0.00***
mi|Xelaev18035905m.g|	loc100494984.L	-2.38	***0.00***	0.54	0.25	0.30	0.51
mi|Xelaev18032248m.g|	ccne2.L	-2.38	***0.00***	0.71	0.12	0.43	0.35
mi|Xelaev18031362m.g|	loc100494665.L	-2.37	***0.00***	0.83	0.17	-1.23	0.02
mi|Xelaev18021944m.g|	cyb5r2.L	-2.35	***0.00***	0.48	0.54	0.27	0.89
mi|Xelaev18035483m.g|	loc101732051.L	-2.28	0.06	-3.95	***0.00***	-7.06	***0.00***
mi|Xelaev18040340m.g|	Xelaev18040340m.g	-2.28	0.06	-2.24	***0.00***	-1.49	0.04
mi|Xelaev18012623m.g|	Xelaev18012623m.g	-2.27	***0.00***	-0.70	0.25	0.12	0.90
mi|Xelaev18015126m.g|	rpa2.S	-2.27	***0.00***	0.94	0.03	0.37	0.39
mi|Xelaev18021834m.g|	prr5l.L	-2.26	***0.01***	0.02	1.00	-0.28	0.68
mi|Xelaev18032344m.g|	anxa13.L	-2.26	***0.01***	0.41	0.62	1.28	0.06
mi|Xelaev18038114m.g|	suv39h1.L	-2.24	***0.00***	0.65	0.16	0.12	0.84
mi|Xelaev18047177m.g|	cdca7.S	-2.24	***0.00***	1.00	0.02	0.36	0.40
mi|Xelaev18028222m.g|	zmcm3.L	-2.22	***0.00***	1.00	0.02	0.43	0.30
mi|Xelaev18043120m.g|	krt12.L	-2.22	***0.00***	-1.33	***0.00***	-1.69	***0.00***
mi|Xelaev18011415m.g|	mx2.L	-2.22	***0.00***	0.44	0.38	0.08	0.87
mi|Xelaev18032898m.g|	Xelaev18032898m.g	-2.20	***0.01***	-0.30	0.84	-0.04	1.00
mi|Xelaev18013147m.g|	Xelaev18013147m.g	-2.20	***0.01***	0.95	0.21	0.31	0.72
mi|Xelaev18020044m.g|	cd74.S	-2.20	***0.00***	-1.57	***0.00***	-0.81	0.05
mi|Xelaev18039147m.g|	tap1.L	-2.20	***0.00***	0.75	0.29	-0.43	0.56
mi|Xelaev18024639m.g|	Xelaev18024639m.g	-2.18	***0.00***	0.84	0.10	1.05	0.04
mi|Xelaev18024217m.g|	loc100126636.S	-2.17	***0.00***	0.61	0.15	0.39	0.36
mi|Xelaev18014617m.g|	ccl5.S	-2.15	0.03	-1.28	0.03	-1.66	***0.00***
mi|Xelaev18001554m.g|	Xelaev18001554m.g	-2.15	***0.00***	1.00	0.03	0.25	0.60
mi|Xelaev18013438m.g|	mars.L	-2.15	***0.00***	0.71	0.17	0.85	0.11
mi|Xelaev18034957m.g|	Xetrov90018123m.L	-2.14	***0.00***	-1.00	0.05	-1.45	0.01
mi|Xelaev18028235m.g|	Xelaev18028235m.g	-2.12	***0.00***	0.23	0.62	0.56	0.20
mi|Xelaev18025680m.g|	Xelaev18025680m.g	-2.11	***0.00***	1.09	0.01	0.65	0.12
mi|Xelaev18017634m.g|	tfec.L	-2.09	***0.01***	-1.17	0.03	0.36	0.55
mi|Xelaev18015006m.g|	tuba3c.S	-2.09	***0.00***	0.72	0.08	0.46	0.27

mi|Xelaev18043986m.g|	e2f1.L	-2.08	***0.00***	1.53	***0.00***	0.36	0.45
mi|Xelaev18032996m.g|	rpa3.S	-2.06	***0.00***	0.42	0.36	0.08	0.87
mi|Xelaev18025789m.g|	manf.S	-2.06	***0.00***	1.10	0.01	0.60	0.16
mi|Xelaev18033043m.g|	cdca7l.S	-2.06	***0.00***	0.10	0.94	0.59	0.31
mi|Xelaev18015481m.g|	gk.S	-2.05	***0.00***	0.46	0.33	-0.01	1.00
mi|Xelaev18018522m.g|	loc100485269.L	-2.05	***0.00***	0.96	0.03	0.39	0.36
mi|Xelaev18011207m.g|	slc22a15.1.L	-2.04	***0.00***	0.59	0.24	0.21	0.72
mi|Xelaev18043892m.g|	znfx1.L	-2.02	***0.00***	0.60	0.28	-0.30	0.56
mi|Xelaev18014939m.g|	ube2t.S	-2.02	***0.00***	0.73	0.10	0.08	0.91
mi|Xelaev18020338m.g|	pcna.S	-2.02	***0.00***	0.74	0.08	0.23	0.57
mi|Xelaev18044530m.g|	mcm6.2.L	-2.01	***0.00***	0.94	0.02	0.45	0.29
mi|Xelaev18007315m.g|	Xetrov90002577m.L	-2.01	0.25	-2.99	***0.00***	-0.42	0.56
mi|Xelaev18011199m.g|	Xelaev18011199m.g	-2.01	0.25	-3.15	***0.00***	-0.55	0.65
mi|Xelaev18032204m.g|	esrp1.L	-2.01	0.25	-1.04	0.16	-3.37	***0.00***
mi|Xelaev18009370m.g|	sec24d-like.1.S	-2.00	***0.00***	0.99	0.05	0.26	0.68
mi|Xelaev18012305m.g|	loc101734700.L	-1.99	0.01	-2.76	***0.00***	0.39	0.44
mi|Xelaev18022635m.g|	has3.L	-1.99	***0.00***	0.85	0.11	-0.18	0.74
mi|Xelaev18036044m.g|	rcn3.L	-1.98	***0.00***	0.01	0.99	0.25	0.58
mi|Xelaev18042619m.g|	ebp.S	-1.98	***0.00***	0.42	0.36	0.17	0.70
mi|Xelaev18011472m.g|	smim11a.L	-1.97	***0.00***	0.71	0.14	0.38	0.48
mi|Xelaev18003303m.g|	kdelc2.L	-1.96	***0.00***	0.48	0.28	0.45	0.35
mi|Xelaev18025556m.g|	mcm5.S	-1.96	***0.00***	0.92	0.03	0.77	0.07
mi|Xelaev18036887m.g|	hhex.S	-1.95	***0.00***	-0.61	0.24	0.28	0.65
mi|Xelaev18002538m.g|	usp18.L	-1.95	***0.00***	0.85	0.18	0.32	0.59
mi|Xelaev18015874m.g|	cdk2.S	-1.95	***0.00***	0.78	0.07	0.26	0.55
mi|Xelaev18031417m.g|	Xetrov90016017m.L	-1.95	***0.00***	0.36	0.54	0.17	0.76
mi|Xelaev18006960m.g|	znf367.L	-1.94	***0.00***	1.38	***0.01***	0.31	0.58
mi|Xelaev18001683m.g|	Xelaev18001683m.g	-1.94	0.13	-0.55	0.65	-3.02	***0.00***
mi|Xelaev18009179m.g|	msmo1.S	-1.93	***0.00***	0.37	0.39	0.06	0.90
mi|Xelaev18033586m.g|	loc100492069.S	-1.92	***0.00***	0.70	0.16	0.17	0.72
mi|Xelaev18035389m.g|	c1qtnf5.L	-1.92	***0.00***	1.00	0.06	1.36	0.01
mi|Xelaev18013189m.g|	loc100490530.L	-1.91	***0.00***	0.52	0.23	0.19	0.67
mi|Xelaev18007079m.g|	loc548369.L	-1.91	0.06	-3.12	***0.00***	-3.32	***0.00***
mi|Xelaev18030303m.g|	emilin1.S	-1.90	***0.00***	0.26	0.55	0.36	0.40
mi|Xelaev18021948m.g|	olfml1.L	-1.90	***0.00***	0.16	0.84	-0.63	0.27
mi|Xelaev18023847m.g|	manf.L	-1.90	***0.00***	0.95	0.03	0.46	0.28
mi|Xelaev18015054m.g|	Xelaev18015054m.g	-1.90	0.03	-0.77	0.07	-1.45	***0.00***
mi|Xelaev18044607m.g|	wdsub1.L	-1.89	***0.00***	-0.01	1.00	-0.24	0.64
mi|Xelaev18047780m.g|	Xelaev18047780m.g	-1.89	0.02	-0.61	0.18	-2.49	***0.00***
mi|Xelaev18013377m.g|	cdk2.L	-1.88	***0.00***	0.68	0.10	0.32	0.44
mi|Xelaev18039592m.g|	vrk1.L	-1.87	***0.00***	0.50	0.25	0.35	0.43
mi|Xelaev18014996m.g|	arg2.S	-1.87	***0.00***	0.58	0.17	-0.11	0.79
mi|Xelaev18019057m.g|	farsa.L	-1.87	***0.00***	0.92	0.03	0.59	0.17

mi|Xelaev18011643m.g|	bcl7b.L	-1.86	***0.00***	0.79	0.07	0.14	0.75
mi|Xelaev18010866m.g|	loc733945.S	-1.85	***0.00***	0.72	0.12	0.16	0.83
mi|Xelaev18027985m.g|	ccl34.L	-1.84	***0.00***	1.13	0.03	4.70	***0.00***
mi|Xelaev18017782m.g|	lrrc17.L	-1.84	***0.00***	0.36	0.42	-0.16	0.74
mi|Xelaev18013725m.g|	hsph1.L	-1.84	***0.00***	0.90	0.04	0.52	0.22
mi|Xelaev18039138m.g|	Xelaev18039138m.g	-1.83	***0.00***	-1.11	0.02	-1.23	***0.01***
mi|Xelaev18000877m.g|	mcm7.S	-1.83	***0.00***	0.68	0.10	0.33	0.43
mi|Xelaev18028040m.g|	rrm2.2.L	-1.83	***0.00***	0.33	0.43	-0.04	0.93
mi|Xelaev18009948m.g|	isyna1.S	-1.82	***0.00***	0.50	0.25	0.15	0.72
mi|Xelaev18039633m.g|	lrr1.L	-1.82	***0.00***	0.27	0.57	-0.29	0.56
mi|Xelaev18027728m.g|	pcolce2.L	-1.81	***0.00***	-0.14	0.75	0.43	0.31
mi|Xelaev18034668m.g|	npm3.L	-1.80	***0.00***	1.42	***0.00***	0.59	0.18
mi|Xelaev18047637m.g|	crym.S	-1.80	***0.00***	0.16	0.73	0.45	0.35
mi|Xelaev18036014m.g|	loc100493902.L	-1.80	***0.00***	-0.97	0.04	-0.01	1.00
mi|Xelaev18045102m.g|	hbg1.L	-1.80	0.19	0.59	0.78	-3.37	***0.00***
mi|Xelaev18021917m.g|	rdm1-like.L	-1.79	***0.00***	0.32	0.50	-0.72	0.14
mi|Xelaev18020367m.g|	loc100485269.S	-1.78	***0.00***	0.90	0.04	0.26	0.54
mi|Xelaev18040846m.g|	loc100488697.L	-1.78	***0.01***	-1.58	***0.00***	1.41	0.01
mi|Xelaev18045905m.g|	cdc6.S	-1.78	***0.00***	0.71	0.13	0.23	0.64
mi|Xelaev18027786m.g|	farsb.L	-1.77	***0.00***	1.04	0.01	0.47	0.27
mi|Xelaev18025634m.g|	loc101734319.S	-1.77	***0.00***	0.23	0.62	-0.10	0.86
mi|Xelaev18004056m.g|	Xelaev18004056m.g	-1.77	0.03	-2.14	***0.00***	0.37	0.52
mi|Xelaev18015037m.g|	Xelaev18015037m.g	-1.77	0.01	-0.86	0.04	-1.65	***0.00***
mi|Xelaev18044351m.g|	Xelaev18044351m.g	-1.76	***0.01***	0.74	0.23	0.30	0.63
mi|Xelaev18039025m.g|	vgll1.L	-1.75	0.40	-0.56	0.47	-3.49	***0.00***
mi|Xelaev18030637m.g|	loc101733516-like.L	-1.75	0.40	0.79	0.44	-2.11	***0.00***
mi|Xelaev18030082m.g|	odc1.S	-1.75	***0.00***	0.91	0.03	0.26	0.53
mi|Xelaev18031891m.g|	tyms.L	-1.74	***0.00***	0.58	0.17	0.21	0.63
mi|Xelaev18038825m.g|	tnmd.L	-1.74	***0.01***	-1.88	***0.00***	-0.91	0.19
mi|Xelaev18029627m.g|	Xelaev18029627m.g	-1.74	***0.00***	0.38	0.40	0.11	0.82
mi|Xelaev18013138m.g|	uggt2.L	-1.73	***0.00***	0.93	0.03	0.13	0.78
mi|Xelaev18030897m.g|	rpa3.L	-1.73	***0.00***	0.86	0.04	0.09	0.83
mi|Xelaev18023929m.g|	rad18.L	-1.73	***0.00***	-0.06	0.94	0.08	0.89
mi|Xelaev18039725m.g|	trmt61a.L	-1.73	***0.00***	0.95	0.03	0.88	0.05
mi|Xelaev18040019m.g|	dtd2.L	-1.73	***0.00***	0.17	0.73	0.31	0.52
mi|Xelaev18035692m.g|	Xelaev18035692m.g	-1.72	0.12	2.19	***0.01***	-1.00	0.19
mi|Xelaev18006820m.g|	mlana-like.L	-1.72	0.12	0.40	0.67	-2.08	***0.00***
mi|Xelaev18012770m.g|	psph.L	-1.72	***0.00***	1.27	***0.00***	0.35	0.42
mi|Xelaev18022776m.g|	orc6.L	-1.71	***0.00***	0.63	0.15	0.34	0.45
mi|Xelaev18028268m.g|	ptrhd1.L	-1.71	***0.00***	0.46	0.29	0.30	0.49
mi|Xelaev18007801m.g|	kntc1.L	-1.70	***0.00***	0.41	0.34	0.02	0.98
mi|Xelaev18026654m.g|	eif2ak2.L	-1.70	***0.00***	0.71	0.11	0.69	0.12
mi|Xelaev18033485m.g|	rbm24.S	-1.69	***0.00***	0.46	0.42	-0.42	0.43

mi|Xelaev18031850m.g|	loc100492069.L	-1.69	***0.00***	0.12	0.78	0.49	0.24
mi|Xelaev18034336m.g|	slc2a3.L	-1.69	***0.00***	-0.32	0.53	-0.98	0.04
mi|Xelaev18038451m.g|	hspa5.L	-1.69	***0.00***	1.17	***0.01***	0.44	0.28
mi|Xelaev18041802m.g|	hspa5.S	-1.68	***0.00***	1.24	***0.00***	0.42	0.31
mi|Xelaev18019237m.g|	Xelaev18019237m.g	-1.68	0.01	-1.12	0.04	-1.35	***0.01***
mi|Xelaev18003621m.g|	Xelaev18003621m.g	-1.68	***0.01***	-0.13	0.83	0.43	0.49
mi|Xelaev18003932m.g|	msmo1.L	-1.68	***0.00***	0.48	0.27	0.25	0.55
mi|Xelaev18039046m.g|	slc1a5.L	-1.68	***0.00***	1.46	***0.00***	0.36	0.39
mi|Xelaev18030195m.g|	Xelaev18030195m.g	-1.68	***0.00***	0.92	0.04	0.10	0.81
mi|Xelaev18043312m.g|	higd1b.L	-1.68	***0.01***	-0.52	0.33	-0.75	0.17
mi|Xelaev18030970m.g|	cycs.L	-1.68	***0.00***	0.74	0.08	0.33	0.43
mi|Xelaev18020849m.g|	lmf2.S	-1.67	***0.00***	0.91	0.05	1.01	0.03
mi|Xelaev18022958m.g|	rpe65.L	-1.67	***0.01***	-0.11	0.82	-0.23	0.70
mi|Xelaev18040854m.g|	Xelaev18040854m.g	-1.67	***0.01***	-0.06	0.95	-0.27	0.60
mi|Xelaev18015189m.g|	cdca8.S	-1.67	***0.00***	0.10	0.85	0.00	1.00
mi|Xelaev18017384m.g|	hsp90b1.L	-1.67	***0.00***	1.05	0.01	0.39	0.34
mi|Xelaev18016256m.g|	mmp7.S	-1.66	***0.00***	-1.70	***0.00***	-0.79	0.19
mi|Xelaev18030079m.g|	rrm2.2.S	-1.66	***0.00***	0.44	0.29	-0.10	0.81
mi|Xelaev18047548m.g|	nudt1.S	-1.65	***0.00***	0.86	0.04	0.31	0.47
mi|Xelaev18046644m.g|	e2f1.S	-1.65	***0.00***	0.88	0.05	0.19	0.69
mi|Xelaev18016115m.g|	ebpl.S	-1.65	***0.00***	0.63	0.15	0.03	0.95
mi|Xelaev18019361m.g|	gstp1.L	-1.65	***0.00***	1.40	***0.00***	2.11	***0.00***
mi|Xelaev18025161m.g|	tmem125.S	-1.65	***0.01***	-0.25	0.70	-1.26	0.09
mi|Xelaev18034537m.g|	oat.1.L	-1.64	***0.00***	0.29	0.52	0.11	0.80
mi|Xelaev18032773m.g|	higd1a.S	-1.64	***0.00***	0.64	0.15	0.43	0.32
mi|Xelaev18023851m.g|	hemk1.L	-1.64	***0.00***	0.79	0.08	0.70	0.12
mi|Xelaev18002493m.g|	akr1c2.L	-1.63	***0.00***	-0.02	0.97	1.27	0.01
mi|Xelaev18032592m.g|	abcf2.S	-1.62	***0.00***	0.80	0.06	0.51	0.22
mi|Xelaev18015143m.g|	clspn.S	-1.62	***0.00***	0.85	0.05	0.14	0.74
mi|Xelaev18031991m.g|	mcm4.L	-1.62	***0.00***	1.05	0.01	0.53	0.20
mi|Xelaev18012948m.g|	loc100490253.L	-1.61	0.17	-3.15	***0.00***	-1.13	0.06
mi|Xelaev18013131m.g|	Xetrov90006020m.L	-1.61	0.17	6.34	***0.00***	0.87	0.59
mi|Xelaev18011478m.g|	donson.L	-1.61	***0.00***	0.27	0.56	-0.07	0.89
mi|Xelaev18001975m.g|	Xelaev18001975m.g	-1.60	0.17	3.66	***0.00***	0.48	1.00
mi|Xelaev18023218m.g|	dhcr24.L	-1.60	***0.00***	0.19	0.67	0.33	0.45
mi|Xelaev18005788m.g|	Xelaev18005788m.g	-1.60	***0.00***	0.81	0.06	0.15	0.73
mi|Xelaev18009263m.g|	smarca5.S	-1.60	***0.00***	0.67	0.11	0.25	0.55
mi|Xelaev18012341m.g|	slc16a1.L	-1.60	***0.00***	0.69	0.11	0.38	0.38
mi|Xelaev18027199m.g|	cep57l1.L	-1.59	***0.00***	0.05	0.94	0.02	0.98
mi|Xelaev18037689m.g|	tnnt1.S	-1.58	***0.00***	0.47	0.46	-0.80	0.16
mi|Xelaev18025959m.g|	ccdc3-like.2.S	-1.58	0.06	-2.28	***0.00***	0.30	0.73
mi|Xelaev18046413m.g|	loc100497487.S	-1.57	***0.00***	0.98	0.04	0.63	0.19
mi|Xelaev18032379m.g|	sqle.L	-1.56	***0.00***	0.35	0.46	0.42	0.35

mi|Xelaev18024120m.g|	loc100491288.L	-1.56	***0.00***	0.44	0.35	0.50	0.30
mi|Xelaev18026675m.g|	loc101735110.L	-1.56	0.40	-0.86	0.30	-2.79	***0.00***
mi|Xelaev18008591m.g|	Xelaev18008591m.g	-1.56	0.15	-1.94	***0.00***	0.06	1.00
mi|Xelaev18025227m.g|	pik3r3.S	-1.56	***0.00***	0.63	0.21	0.02	1.00
mi|Xelaev18011675m.g|	blmh.L	-1.55	***0.00***	0.78	0.06	0.21	0.63
mi|Xelaev18008117m.g|	dhfr.L	-1.55	***0.00***	0.45	0.32	0.20	0.66
mi|Xelaev18008734m.g|	sfxn5.S	-1.55	***0.01***	0.58	0.31	0.18	0.78
mi|Xelaev18047992m.g|	loc398467.S	-1.54	***0.00***	0.90	0.04	0.74	0.09
mi|Xelaev18017827m.g|	mcm10.L	-1.54	***0.00***	0.30	0.51	-0.07	0.90
mi|Xelaev18017115m.g|	dck.2.L	-1.54	***0.00***	0.71	0.11	-0.01	0.98
mi|Xelaev18024332m.g|	fen1.S	-1.54	***0.00***	0.78	0.07	0.36	0.40
mi|Xelaev18007557m.g|	gatc-like.L	-1.54	***0.00***	0.59	0.17	0.10	0.84
mi|Xelaev18007632m.g|	loc100493155.L	-1.53	***0.00***	-0.09	0.83	0.24	0.61
mi|Xelaev18025893m.g|	mcm2.S	-1.53	***0.00***	1.11	***0.01***	0.25	0.56
mi|Xelaev18038250m.g|	Xelaev18038250m.g	-1.53	***0.01***	-0.71	0.14	-1.06	0.02
mi|Xelaev18026606m.g|	mgme1.L	-1.53	***0.01***	-0.21	0.70	0.10	0.84
mi|Xelaev18034687m.g|	exosc1.L	-1.52	***0.00***	0.74	0.09	0.44	0.31
mi|Xelaev18033701m.g|	mcm4.S	-1.52	***0.00***	1.04	0.02	0.41	0.34
mi|Xelaev18047799m.g|	med9.S	-1.52	***0.00***	0.11	0.82	0.19	0.70
mi|Xelaev18012154m.g|	Xelaev18012154m.g	-1.51	***0.00***	-0.54	0.26	-0.51	0.31
mi|Xelaev18008853m.g|	spon2.S	-1.51	***0.00***	-0.35	0.45	0.91	0.05
mi|Xelaev18023891m.g|	poc1a.L	-1.50	***0.01***	0.89	0.10	0.49	0.39
mi|Xelaev18010558m.g|	pgam5.S	-1.50	***0.00***	0.73	0.09	0.36	0.41
mi|Xelaev18037790m.g|	Xelaev18037790m.g	-1.50	***0.00***	0.78	0.06	0.28	0.50
mi|Xelaev18012486m.g|	loc100497282.L	-1.50	***0.01***	-0.32	0.52	-0.22	0.70
mi|Xelaev18025187m.g|	nasp.S	-1.50	***0.00***	0.58	0.17	-0.09	0.84
mi|Xelaev18020998m.g|	Xelaev18020998m.g	-1.50	0.24	-2.44	***0.00***	-1.43	0.02
mi|Xelaev18023880m.g|	arhgdib.L	-1.50	***0.00***	-1.97	***0.00***	-0.36	0.39
mi|Xelaev18007810m.g|	b3gnt4.L	-1.49	***0.00***	0.45	0.28	0.17	0.70
mi|Xelaev18016180m.g|	hsph1.S	-1.49	***0.00***	0.91	0.03	0.38	0.36
mi|Xelaev18010191m.g|	cks2.S	-1.49	***0.00***	0.09	0.84	0.08	0.89
mi|Xelaev18007758m.g|	aacs.L	-1.49	***0.00***	0.38	0.36	0.20	0.63
mi|Xelaev18025098m.g|	rpe65.S	-1.48	***0.00***	0.11	0.82	0.08	0.90
mi|Xelaev18044349m.g|	Xelaev18044349m.g	-1.48	***0.00***	0.52	0.27	0.97	0.06
mi|Xelaev18023178m.g|	orc1.L	-1.48	***0.00***	0.71	0.11	0.46	0.29
mi|Xelaev18007012m.g|	ipo4.L	-1.48	***0.00***	0.91	0.03	0.67	0.12
mi|Xelaev18004185m.g|	c1qb.L	-1.48	***0.00***	-1.71	***0.00***	-1.15	***0.01***
mi|Xelaev18028001m.g|	tmem18.L	-1.47	***0.00***	-0.13	0.82	0.25	0.63
mi|Xelaev18044855m.g|	bard1.L	-1.47	***0.00***	0.39	0.40	0.25	0.60
mi|Xelaev18002599m.g|	Xelaev18002599m.g	-1.46	***0.00***	-0.47	0.30	-0.27	0.54
mi|Xelaev18017677m.g|	tmem229a.L	-1.46	***0.00***	0.43	0.36	0.23	0.61
mi|Xelaev18005686m.g|	mad2l1.L	-1.46	***0.00***	0.18	0.70	-0.23	0.66
mi|Xelaev18030853m.g|	cyp51a1.L	-1.45	***0.00***	0.33	0.44	0.47	0.26

mi|Xelaev18005620m.g|	ccna2.L	-1.45	***0.00***	0.30	0.49	0.05	0.91
mi|Xelaev18022668m.g|	chst6.L	-1.45	***0.00***	-0.17	0.76	-0.27	0.58
mi|Xelaev18028467m.g|	Xelaev18028467m.g	-1.44	0.64	-0.07	1.00	-2.19	***0.00***
mi|Xelaev18043014m.g|	muc1.S	-1.44	0.64	-0.47	0.45	-2.57	***0.00***
mi|Xelaev18045538m.g|	Xelaev18045538m.g	-1.44	0.64	-1.38	0.03	-2.03	***0.00***
mi|Xelaev18046397m.g|	kcnj16.S	-1.44	0.64	-0.67	0.47	-2.85	***0.01***
mi|Xelaev18010407m.g|	Xetrov90002799m.S	-1.44	0.64	3.77	***0.00***	-0.84	1.00
mi|Xelaev18027830m.g|	agxt.L	-1.44	0.64	-2.80	***0.00***	0.33	1.00
mi|Xelaev18022567m.g|	cenpn.L	-1.44	***0.00***	-0.02	1.00	0.47	0.35
mi|Xelaev18031900m.g|	abhd3.L	-1.44	***0.00***	-0.11	0.81	0.15	0.73
mi|Xelaev18004404m.g|	hyou1.S	-1.44	***0.00***	0.92	0.03	0.20	0.64
mi|Xelaev18003153m.g|	abcb8.L	-1.43	***0.00***	0.50	0.25	0.34	0.44
mi|Xelaev18000775m.g|	farsa.S	-1.43	***0.00***	0.79	0.06	0.47	0.26
mi|Xelaev18031586m.g|	tubb2b.L	-1.43	***0.00***	0.33	0.43	0.21	0.61
mi|Xelaev18033544m.g|	cndp2.S	-1.43	***0.00***	-0.95	0.02	-0.32	0.44
mi|Xelaev18007818m.g|	hpd-like.1.L	-1.43	***0.00***	0.17	0.67	0.24	0.56
mi|Xelaev18043830m.g|	psme3.L	-1.43	***0.00***	0.71	0.09	0.19	0.64
mi|Xelaev18027784m.g|	sgpp2.L	-1.42	***0.00***	0.97	0.03	0.16	0.72
mi|Xelaev18020619m.g|	Xelaev18020619m.g	-1.42	***0.00***	0.45	0.30	0.33	0.46
mi|Xelaev18034801m.g|	hells.L	-1.42	***0.00***	0.75	0.08	0.45	0.29
mi|Xelaev18016415m.g|	serpinh1.S	-1.42	***0.00***	0.41	0.32	0.10	0.81
mi|Xelaev18005162m.g|	loc100488322.L	-1.42	***0.01***	0.09	0.85	-0.10	0.85
mi|Xelaev18011098m.g|	lipg.S	-1.42	***0.00***	0.47	0.26	0.14	0.74
mi|Xelaev18042873m.g|	loc100485201-like.S	-1.42	***0.01***	0.18	0.71	0.22	0.66
mi|Xelaev18012909m.g|	gk.L	-1.41	***0.00***	0.51	0.27	0.40	0.37
mi|Xelaev18039628m.g|	pole2.L	-1.41	***0.00***	0.36	0.41	0.33	0.45
mi|Xelaev18002995m.g|	slc6a13.L	-1.41	***0.00***	-0.24	0.55	0.47	0.26
mi|Xelaev18018556m.g|	pcna.L	-1.41	***0.00***	0.41	0.32	0.22	0.59
mi|Xelaev18018247m.g|	cdc42se2-like.2.L	-1.41	***0.00***	0.49	0.27	0.19	0.67
mi|Xelaev18032976m.g|	col1a2.S	-1.41	***0.00***	0.17	0.68	0.02	0.97
mi|Xelaev18022034m.g|	fen1.L	-1.41	***0.00***	0.34	0.41	0.31	0.46
mi|Xelaev18011723m.g|	Xelaev18011723m.g	-1.41	***0.01***	0.01	1.00	-0.18	0.71
mi|Xelaev18026477m.g|	plek.L	-1.41	***0.01***	-0.95	0.04	0.16	0.75
mi|Xelaev18033246m.g|	psma2.S	-1.41	***0.00***	0.43	0.30	0.21	0.61
mi|Xelaev18015839m.g|	tuba1c.S	-1.40	***0.00***	0.19	0.65	0.23	0.57
mi|Xelaev18011452m.g|	chaf1b.L	-1.40	***0.00***	0.43	0.33	0.26	0.56
mi|Xelaev18047894m.g|	phgdh.S	-1.40	***0.00***	1.13	***0.01***	0.27	0.52
mi|Xelaev18033497m.g|	e2f3.S	-1.40	***0.00***	0.52	0.24	0.39	0.39
mi|Xelaev18020080m.g|	sec24a.S	-1.40	***0.01***	-0.42	0.40	-0.57	0.25
mi|Xelaev18047351m.g|	plcl1.S	-1.40	***0.01***	0.06	0.90	0.42	0.39
mi|Xelaev18020309m.g|	ncaph.S	-1.40	***0.00***	0.17	0.70	0.46	0.30
mi|Xelaev18013439m.g|	Xetrov90006328m.L	-1.40	***0.00***	0.91	0.03	0.74	0.09
mi|Xelaev18004059m.g|	gale.S	-1.40	***0.00***	0.45	0.32	0.48	0.29

mi|Xelaev18017957m.g|	cttn-like.1.L	-1.39	***0.01***	-1.07	0.02	-0.92	0.04
mi|Xelaev18015084m.g|	fgr.S	-1.39	***0.01***	-1.14	0.02	-0.41	0.39
mi|Xelaev18026213m.g|	flvcr1.L	-1.39	***0.00***	0.51	0.24	0.19	0.65
mi|Xelaev18012625m.g|	gale.L	-1.38	***0.01***	0.32	0.49	0.32	0.53
mi|Xelaev18023164m.g|	cdkn2c.L	-1.38	***0.01***	0.27	0.56	0.45	0.39
mi|Xelaev18033829m.g|	pmp2.S	-1.37	***0.00***	-3.77	***0.00***	0.21	0.63
mi|Xelaev18027677m.g|	slc33a1.L	-1.37	***0.00***	0.49	0.28	0.13	0.81
mi|Xelaev18009215m.g|	tlr2.S	-1.37	0.24	-0.48	0.56	-2.09	***0.00***
mi|Xelaev18009326m.g|	ccna2.S	-1.37	***0.00***	-0.54	0.22	0.40	0.37
mi|Xelaev18019201m.g|	mcm7.L	-1.37	***0.00***	0.69	0.10	0.32	0.44
mi|Xelaev18018358m.g|	rhcg.L	-1.37	0.02	-1.84	***0.00***	-0.51	0.28
mi|Xelaev18026072m.g|	mcm8.L	-1.37	***0.00***	0.29	0.50	0.38	0.39
mi|Xelaev18023640m.g|	polr3h.L	-1.37	***0.00***	0.64	0.15	0.29	0.52
mi|Xelaev18040478m.g|	loc100487362.L	-1.36	***0.00***	0.44	0.33	-0.39	0.38
mi|Xelaev18008929m.g|	lyar.S	-1.36	***0.00***	0.61	0.15	0.40	0.35
mi|Xelaev18007388m.g|	selm.L	-1.35	***0.00***	0.59	0.19	0.27	0.57
mi|Xelaev18024424m.g|	cyb5r2.S	-1.35	***0.00***	-0.35	0.43	0.12	0.78
mi|Xelaev18018413m.g|	aldh1a3-like.L	-1.35	***0.01***	-0.40	0.41	1.04	0.03
mi|Xelaev18033448m.g|	loc548390-like.S	-1.35	***0.01***	-0.68	0.17	0.01	1.00
mi|Xelaev18039466m.g|	c14orf1.L	-1.34	***0.00***	0.34	0.44	0.20	0.65
mi|Xelaev18028226m.g|	xkr5.L	-1.34	***0.00***	0.31	0.51	0.66	0.17
mi|Xelaev18023542m.g|	mcm5.L	-1.34	***0.00***	0.80	0.06	0.61	0.15
mi|Xelaev18017704m.g|	creld2.L	-1.34	***0.00***	1.17	***0.01***	0.51	0.24
mi|Xelaev18041358m.g|	ahsa1.S	-1.34	***0.00***	0.63	0.13	0.06	0.89
mi|Xelaev18031988m.g|	spidr.L	-1.34	***0.00***	0.87	0.06	0.21	0.68
mi|Xelaev18038983m.g|	loc101732341.L	-1.34	***0.00***	0.47	0.30	0.41	0.38
mi|Xelaev18003271m.g|	loc100498108-like.L	-1.33	0.02	-1.32	***0.01***	-0.74	0.11
mi|Xelaev18006123m.g|	aptx.L	-1.33	***0.00***	0.65	0.14	0.25	0.58
mi|Xelaev18034826m.g|	dnajc9.L	-1.33	***0.00***	0.64	0.13	0.14	0.75
mi|Xelaev18019973m.g|	ckap4.S	-1.33	***0.00***	0.38	0.37	0.24	0.59
mi|Xelaev18012297m.g|	ppil1.L	-1.32	***0.00***	0.49	0.25	0.14	0.74
mi|Xelaev18026085m.g|	prorsd1p.L	-1.32	***0.00***	0.55	0.21	0.32	0.49
mi|Xelaev18013694m.g|	ufm1.L	-1.32	***0.00***	0.52	0.23	0.38	0.37
mi|Xelaev18014175m.g|	blmh.S	-1.32	***0.00***	0.55	0.19	0.01	0.98
mi|Xelaev18022663m.g|	Xetrov90011556m.L	-1.31	***0.01***	0.61	0.19	0.35	0.49
mi|Xelaev18021991m.g|	ppp1r32.L	-1.31	***0.00***	0.57	0.19	0.57	0.19
mi|Xelaev18012800m.g|	rfc2.L	-1.31	***0.00***	0.54	0.20	0.19	0.66
mi|Xelaev18003623m.g|	Xelaev18003623m.g	-1.31	***0.00***	-0.07	0.87	0.48	0.30
mi|Xelaev18010174m.g|	znf367.S	-1.30	***0.01***	1.19	***0.01***	0.55	0.25
mi|Xelaev18045649m.g|	Xelaev18045649m.g	-1.30	0.06	-1.96	***0.00***	-0.44	0.45
mi|Xelaev18004506m.g|	hells.S	-1.30	***0.00***	0.66	0.12	0.41	0.34
mi|Xelaev18034855m.g|	lrrc20.L	-1.30	***0.01***	0.79	0.08	0.20	0.67
mi|Xelaev18034026m.g|	sqle.S	-1.30	***0.00***	0.03	0.95	0.24	0.57

mi|Xelaev18035426m.g|	hyou1.L	-1.30	***0.00***	1.02	0.02	0.21	0.62
mi|Xelaev18025879m.g|	chchd4.S	-1.29	***0.00***	0.29	0.50	0.26	0.55
mi|Xelaev18026651m.g|	dhx57.L	-1.29	***0.00***	0.27	0.56	0.49	0.26
mi|Xelaev18039657m.g|	wdhd1.L	-1.29	***0.00***	0.56	0.19	0.21	0.62
mi|Xelaev18013274m.g|	lcp1.L	-1.29	***0.00***	-1.62	***0.00***	-0.36	0.39
mi|Xelaev18013544m.g|	nckap1l.L	-1.29	***0.01***	-1.41	***0.00***	-0.44	0.32
mi|Xelaev18000904m.g|	Xelaev18000904m.g	-1.29	0.04	-2.11	***0.00***	-0.12	0.91
mi|Xelaev18019581m.g|	eif4a1.L	-1.29	***0.00***	0.72	0.08	0.35	0.39
mi|Xelaev18032784m.g|	pomp.S	-1.28	***0.00***	0.29	0.49	0.20	0.63
mi|Xelaev18046056m.g|	col1a1.S	-1.28	***0.00***	-0.45	0.27	0.06	0.88
mi|Xelaev18006589m.g|	haus8.L	-1.28	***0.00***	0.47	0.29	0.16	0.74
mi|Xelaev18017952m.g|	Xetrov90008497m.L	-1.28	***0.00***	-0.01	0.99	-0.03	0.96
mi|Xelaev18013628m.g|	bcdin3d.L	-1.28	***0.01***	0.29	0.53	0.55	0.22
mi|Xelaev18009381m.g|	mad2l1.S	-1.28	***0.00***	0.43	0.32	0.09	0.84
mi|Xelaev18004541m.g|	pycard.S	-1.28	***0.01***	-0.02	0.96	0.35	0.44
mi|Xelaev18005417m.g|	c4orf27.L	-1.27	***0.00***	0.57	0.18	0.07	0.88
mi|Xelaev18043865m.g|	ube2c.L	-1.27	***0.00***	-0.51	0.24	0.10	0.82
mi|Xelaev18030593m.g|	higd1a.L	-1.27	***0.00***	0.57	0.17	0.29	0.49
mi|Xelaev18017944m.g|	odf3.L	-1.27	***0.00***	-0.02	0.97	0.47	0.27
mi|Xelaev18023014m.g|	psma4.L	-1.27	***0.00***	0.20	0.63	0.28	0.49
mi|Xelaev18028391m.g|	pbk.L	-1.26	***0.00***	0.15	0.74	0.15	0.74
mi|Xelaev18028048m.g|	pdia6.L	-1.26	***0.00***	0.89	0.03	0.50	0.23
mi|Xelaev18018914m.g|	Xelaev18018914m.g	-1.26	0.11	-1.84	***0.00***	-1.06	0.03
mi|Xelaev18034677m.g|	pprc1-like.L	-1.26	***0.00***	1.49	***0.00***	0.60	0.16
mi|Xelaev18028884m.g|	acss1.S	-1.26	***0.00***	0.34	0.43	0.20	0.65
mi|Xelaev18015931m.g|	Xetrov90006328m.S	-1.26	***0.00***	0.79	0.06	0.46	0.27
mi|Xelaev18030705m.g|	idi1.L	-1.26	***0.00***	0.09	0.84	0.09	0.83
mi|Xelaev18001058m.g|	eif4a1.S	-1.26	***0.00***	0.96	0.02	0.31	0.46
mi|Xelaev18020003m.g|	etf1.S	-1.26	***0.00***	0.64	0.12	0.28	0.50
mi|Xelaev18015784m.g|	lcp1.S	-1.25	***0.01***	-1.06	0.01	-0.45	0.30
mi|Xelaev18013690m.g|	Xetrov90006631m.L	-1.25	***0.01***	0.08	0.88	0.31	0.53
mi|Xelaev18005792m.g|	Xelaev18005792m.g	-1.25	***0.00***	0.30	0.49	-0.03	0.96
mi|Xelaev18016226m.g|	mipep.S	-1.25	***0.01***	0.35	0.44	0.17	0.71
mi|Xelaev18020255m.g|	aebp1.S	-1.25	***0.00***	-0.58	0.18	0.37	0.39
mi|Xelaev18012172m.g|	tuba3c.L	-1.25	***0.00***	0.35	0.39	0.31	0.45
mi|Xelaev18047015m.g|	loc100497528.S	-1.25	***0.00***	0.10	0.81	0.80	0.05
mi|Xelaev18017233m.g|	pole4.L	-1.25	***0.01***	0.20	0.68	0.34	0.49
mi|Xelaev18039428m.g|	timm50.L	-1.25	***0.00***	0.78	0.07	0.23	0.60
mi|Xelaev18045037m.g|	card11.L	-1.25	0.03	-1.49	***0.00***	-0.45	0.32
mi|Xelaev18004068m.g|	mfsd2a.S	-1.25	***0.00***	0.35	0.42	0.30	0.48
mi|Xelaev18029509m.g|	gsta4.S	-1.24	0.07	-0.43	0.46	-1.76	***0.01***
mi|Xelaev18039483m.g|	ahsa1.L	-1.24	***0.00***	0.40	0.34	0.08	0.85
mi|Xelaev18038658m.g|	gjb1.L	-1.24	***0.01***	0.83	0.07	0.26	0.61

mi|Xelaev18043299m.g|	col1a1.L	-1.24	***0.00***	-0.34	0.41	0.22	0.60
mi|Xelaev18020297m.g|	mat2a.S	-1.24	***0.00***	0.98	0.02	0.21	0.62
mi|Xelaev18032329m.g|	dscc1.L	-1.24	***0.01***	0.37	0.42	0.07	0.89
mi|Xelaev18031168m.g|	ezh2.L	-1.24	***0.00***	0.42	0.31	0.02	0.96
mi|Xelaev18023992m.g|	ruvbl1.L	-1.24	***0.00***	0.59	0.17	0.53	0.21
mi|Xelaev18006316m.g|	myo1f.L	-1.23	0.02	-1.79	***0.00***	-0.74	0.09
mi|Xelaev18029485m.g|	prim2.S	-1.23	***0.01***	0.56	0.19	-0.04	0.94
mi|Xelaev18016145m.g|	ufm1.S	-1.23	***0.01***	0.36	0.41	0.00	1.00
mi|Xelaev18045889m.g|	krt12.S	-1.23	0.02	-1.36	***0.00***	-1.86	***0.00***
mi|Xelaev18010549m.g|	txnrd2.S	-1.22	***0.00***	0.18	0.68	-0.18	0.67
mi|Xelaev18039139m.g|	Xelaev18039139m.g	-1.22	0.01	-1.18	***0.01***	-1.16	***0.01***
mi|Xelaev18045563m.g|	shmt1.L	-1.22	***0.00***	0.75	0.08	0.52	0.21
mi|Xelaev18007339m.g|	Xelaev18007339m.g	-1.22	***0.01***	0.57	0.21	0.03	0.96
mi|Xelaev18012120m.g|	Xelaev18012120m.g	-1.22	0.06	-0.69	0.10	-2.09	***0.00***
mi|Xelaev18023745m.g|	rft1.L	-1.21	***0.01***	0.38	0.40	0.54	0.23
mi|Xelaev18007400m.g|	mtfp1.L	-1.21	***0.01***	0.61	0.17	0.17	0.72
mi|Xelaev18044318m.g|	loc100492872.L	-1.21	0.02	-1.30	***0.01***	-0.93	0.03
mi|Xelaev18015457m.g|	egfl6.S	-1.21	***0.01***	-0.14	0.78	0.50	0.29
mi|Xelaev18021625m.g|	dnmt1.S	-1.21	***0.00***	0.74	0.08	0.44	0.29
mi|Xelaev18003729m.g|	Xelaev18003729m.g	-1.21	0.28	-0.08	1.00	-1.95	***0.00***
mi|Xelaev18006108m.g|	chaf1a.L	-1.21	***0.01***	0.45	0.29	0.32	0.47
mi|Xelaev18045869m.g|	fkbp10.S	-1.20	***0.00***	0.22	0.60	0.60	0.15
mi|Xelaev18021356m.g|	Xelaev18021356m.g	-1.20	***0.00***	-0.81	0.05	-1.52	***0.00***
mi|Xelaev18007695m.g|	cdc45.L	-1.20	***0.01***	0.42	0.33	0.11	0.80
mi|Xelaev18028880m.g|	uqcc2.S	-1.20	***0.01***	0.46	0.29	0.04	0.95
mi|Xelaev18007704m.g|	comt.L	-1.20	***0.00***	0.66	0.11	0.21	0.61
mi|Xelaev18028947m.g|	tcp1.S	-1.20	***0.00***	0.55	0.19	0.20	0.63
mi|Xelaev18045048m.g|	hspd1.L	-1.20	***0.00***	0.91	0.03	0.18	0.66
mi|Xelaev18018941m.g|	Xelaev18018941m.g	-1.20	***0.01***	0.23	0.62	0.02	1.00
mi|Xelaev18027656m.g|	gfm1.L	-1.19	***0.01***	0.64	0.13	0.39	0.36
mi|Xelaev18034050m.g|	chrac1.S	-1.19	***0.01***	0.22	0.61	0.20	0.65
mi|Xelaev18040951m.g|	fdps.L	-1.19	***0.00***	0.36	0.39	0.33	0.43
mi|Xelaev18012382m.g|	mid1ip1.L	-1.19	***0.00***	0.08	0.85	0.07	0.86
mi|Xelaev18010529m.g|	sdf2l1.S	-1.19	***0.01***	0.75	0.08	0.25	0.57
mi|Xelaev18012617m.g|	ak2.L	-1.18	***0.01***	0.70	0.10	0.63	0.14
mi|Xelaev18003164m.g|	loc100496088.1.L	-1.18	***0.01***	-0.14	0.75	0.41	0.35
mi|Xelaev18047429m.g|	hspe1.S	-1.18	***0.01***	0.66	0.11	0.08	0.85
mi|Xelaev18026526m.g|	kiaa1841.L	-1.18	***0.01***	0.34	0.43	0.06	0.89
mi|Xelaev18016770m.g|	Xelaev18016770m.g	-1.18	0.02	-1.43	***0.00***	-0.54	0.22
mi|Xelaev18003168m.g|	abcf2.L	-1.18	***0.01***	0.80	0.05	0.35	0.41
mi|Xelaev18043144m.g|	cdc6.L	-1.18	***0.01***	1.27	***0.00***	0.29	0.52
mi|Xelaev18034070m.g|	Xelaev18034070m.g	-1.18	0.01	-2.36	***0.00***	-1.33	***0.00***
mi|Xelaev18033059m.g|	cycs.S	-1.18	***0.01***	0.34	0.41	0.02	0.97

mi|Xelaev18029931m.g|	hes6.2.S	-1.17	***0.01***	-0.12	0.76	-0.10	0.81
mi|Xelaev18036193m.g|	spib.L	-1.17	0.02	-1.24	***0.01***	-0.82	0.06
mi|Xelaev18012050m.g|	rpa2.L	-1.17	***0.01***	0.61	0.14	0.20	0.63
mi|Xelaev18030833m.g|	acbd7.L	-1.17	***0.01***	-0.34	0.41	-0.13	0.76
mi|Xelaev18033637m.g|	usp14.S	-1.17	***0.01***	0.44	0.29	0.16	0.70
mi|Xelaev18038499m.g|	ercc6l.L	-1.17	***0.01***	0.06	0.90	0.78	0.08
mi|Xelaev18029506m.g|	elovl5.S	-1.17	***0.01***	0.41	0.32	0.09	0.83
mi|Xelaev18047357m.g|	wdr12.S	-1.16	***0.01***	1.00	0.02	0.65	0.13
mi|Xelaev18017941m.g|	ncaph2.L	-1.16	***0.01***	0.49	0.26	0.25	0.59
mi|Xelaev18030084m.g|	pdia6.S	-1.16	***0.01***	0.74	0.08	0.41	0.33
mi|Xelaev18036061m.g|	Xelaev18036061m.g	-1.15	***0.01***	0.53	0.20	0.17	0.69
mi|Xelaev18011029m.g|	rai14.S	-1.15	***0.01***	-0.09	0.84	0.48	0.26
mi|Xelaev18007684m.g|	sdf2l1.L	-1.15	***0.01***	0.99	0.02	0.31	0.47
mi|Xelaev18007938m.g|	adamts19.L	-1.15	0.09	0.38	0.59	-1.97	***0.00***
mi|Xelaev18002012m.g|	hnrnpdl.S	-1.15	***0.01***	0.48	0.25	0.10	0.81
mi|Xelaev18013378m.g|	pmel-like.L	-1.14	0.07	0.18	0.79	-1.64	***0.00***
mi|Xelaev18015113m.g|	fabp3.S	-1.14	***0.01***	-0.24	0.57	-0.19	0.65
mi|Xelaev18022815m.g|	rarg-like.2.L	-1.14	***0.01***	0.23	0.60	0.34	0.43
mi|Xelaev18016520m.g|	rrm1.S	-1.14	***0.01***	0.64	0.12	0.20	0.63
mi|Xelaev18025300m.g|	dhcr24.S	-1.14	***0.01***	-0.08	0.87	0.17	0.69
mi|Xelaev18021242m.g|	lum.S	-1.13	***0.01***	0.06	0.88	-0.27	0.52
mi|Xelaev18024334m.g|	fads2.S	-1.13	***0.01***	-0.11	0.80	0.18	0.67
mi|Xelaev18035637m.g|	atad3a.L	-1.13	***0.01***	0.54	0.20	0.32	0.45
mi|Xelaev18032513m.g|	tonsl.L	-1.13	***0.01***	0.41	0.35	0.39	0.37
mi|Xelaev18012612m.g|	rbbp4.L	-1.13	***0.01***	0.53	0.20	0.37	0.37
mi|Xelaev18009580m.g|	uhrf1.S	-1.12	***0.01***	0.46	0.27	0.02	0.96
mi|Xelaev18020359m.g|	snrpg.S	-1.12	***0.01***	0.30	0.46	0.20	0.63
mi|Xelaev18039699m.g|	hsp90aa1.1.L	-1.12	***0.01***	0.77	0.07	0.04	0.93
mi|Xelaev18007393m.g|	pole.L	-1.12	***0.01***	0.32	0.45	0.22	0.61
mi|Xelaev18008321m.g|	hmgcs1.L	-1.12	***0.01***	0.30	0.47	0.33	0.43
mi|Xelaev18042357m.g|	ppp5c.S	-1.12	***0.01***	0.49	0.24	0.18	0.67
mi|Xelaev18011707m.g|	proca1.L	-1.12	***0.01***	-0.13	0.75	0.39	0.35
mi|Xelaev18025315m.g|	cacybp.S	-1.12	***0.01***	0.75	0.07	0.11	0.78
mi|Xelaev18016836m.g|	g3bp1.L	-1.12	***0.01***	0.68	0.10	0.22	0.59
mi|Xelaev18043758m.g|	mrpl12.L	-1.12	***0.01***	0.79	0.06	0.25	0.55
mi|Xelaev18005732m.g|	h2afz.L	-1.11	***0.01***	0.22	0.60	0.22	0.59
mi|Xelaev18021302m.g|	hsp90b1.S	-1.11	***0.01***	0.98	0.02	0.18	0.67
mi|Xelaev18008420m.g|	txnl1.L	-1.11	***0.01***	0.48	0.26	0.20	0.64
mi|Xelaev18009793m.g|	prtn3.S	-1.11	0.05	-1.75	***0.00***	-0.45	0.37
mi|Xelaev18039051m.g|	ppp5c.L	-1.11	***0.01***	0.54	0.20	0.13	0.77
mi|Xelaev18000122m.g|	smarca5.L	-1.11	***0.01***	0.70	0.09	0.40	0.33
mi|Xelaev18047504m.g|	rsl1d1.S	-1.11	***0.01***	0.96	0.02	0.49	0.24
mi|Xelaev18045050m.g|	hspe1.L	-1.11	***0.01***	0.95	0.02	0.18	0.67

mi|Xelaev18014965m.g|	Xelaev18014965m.g	-1.11	0.11	-1.14	***0.01***	-1.32	***0.00***
mi|Xelaev18035875m.g|	lig1.L	-1.10	***0.01***	0.36	0.40	0.20	0.64
mi|Xelaev18002665m.g|	Xelaev18002665m.g	-1.10	0.21	0.32	0.77	-2.46	***0.01***
mi|Xelaev18022695m.g|	klhdc4.L	-1.10	***0.01***	0.89	0.03	0.18	0.66
mi|Xelaev18024333m.g|	loc101732576.S	-1.10	***0.01***	0.24	0.57	0.22	0.60
mi|Xelaev18036299m.g|	Xelaev18036299m.g	-1.10	0.24	-1.56	***0.01***	-0.55	0.34
mi|Xelaev18028769m.g|	epcam.S	-1.10	0.24	-0.89	0.08	-2.41	***0.00***
mi|Xelaev18034577m.g|	mki67.L	-1.10	***0.01***	0.19	0.65	0.17	0.69
mi|Xelaev18013125m.g|	gpr183.L	-1.10	0.37	-2.67	***0.00***	-0.85	0.26
mi|Xelaev18047428m.g|	hspd1.S	-1.09	***0.01***	0.96	0.02	0.11	0.79
mi|Xelaev18046690m.g|	slc16a5-like.S	-1.09	0.44	-1.96	***0.01***	0.01	1.00
mi|Xelaev18047252m.g|	col6a1.S	-1.09	***0.01***	-1.26	***0.00***	0.61	0.14
mi|Xelaev18009848m.g|	Xelaev18009848m.g	-1.08	0.48	-1.67	***0.00***	-1.60	0.01
mi|Xelaev18024874m.g|	loc100486268-like.S	-1.08	0.48	-2.99	***0.00***	-0.71	0.59
mi|Xelaev18045537m.g|	Xelaev18045537m.g	-1.08	0.48	-1.61	***0.00***	-2.15	***0.00***
mi|Xelaev18047254m.g|	col6a2.S	-1.07	0.01	-1.20	***0.00***	0.55	0.19
mi|Xelaev18040567m.g|	Xelaev18040567m.g	-1.07	0.54	-2.00	***0.00***	-0.83	0.15
mi|Xelaev18003402m.g|	Xelaev18003402m.g	-1.06	0.64	6.35	***0.00***	0.32	1.00
mi|Xelaev18020058m.g|	csf1r.S	-1.06	0.02	-1.12	***0.01***	-0.92	0.03
mi|Xelaev18004984m.g|	loc734128.L	-1.04	0.64	-2.32	***0.00***	-2.87	***0.00***
mi|Xelaev18019189m.g|	unnamed.L	-1.04	0.64	-6.47	***0.00***	-0.91	0.21
mi|Xelaev18038956m.g|	znf185.L	-1.04	0.64	-2.83	0.06	-2.41	***0.00***
mi|Xelaev18017735m.g|	lamb4.L	-1.04	0.64	6.49	***0.00***	-1.40	0.64
mi|Xelaev18019671m.g|	Xelaev18019671m.g	-1.03	0.19	-1.80	***0.01***	0.38	0.63
mi|Xelaev18020340m.g|	arhgap25.S	-1.02	0.21	-2.40	***0.00***	-2.31	***0.00***
mi|Xelaev18018366m.g|	aen.L	-1.02	0.06	1.60	***0.00***	-0.02	0.97
mi|Xelaev18017269m.g|	loc100494767.L	-1.00	0.31	-2.52	***0.01***	0.49	0.65
mi|Xelaev18020200m.g|	dok2.S	-1.00	0.21	-1.81	***0.01***	0.01	1.00
mi|Xelaev18011933m.g|	ccl5.L	-0.99	0.14	-1.79	***0.00***	-1.64	***0.00***
mi|Xelaev18012141m.g|	Xelaev18012141m.g	-0.98	0.24	-0.61	0.15	-1.71	***0.00***
mi|Xelaev18004184m.g|	c1qc.L	-0.98	0.03	-1.52	***0.00***	-1.06	0.01
mi|Xelaev18028932m.g|	loc101735110.S	-0.97	0.48	-0.80	0.19	-2.07	***0.00***
mi|Xelaev18042429m.g|	loc100495183.S	-0.97	0.48	-1.39	0.02	-1.36	***0.01***
mi|Xelaev18012304m.g|	pigr.L	-0.94	0.06	-2.27	***0.00***	-0.35	0.41
mi|Xelaev18018667m.g|	stc1.L	-0.94	0.05	0.77	0.09	1.27	***0.01***
mi|Xelaev18019751m.g|	parvg.S	-0.94	0.13	-1.62	***0.00***	-0.03	1.00
mi|Xelaev18007316m.g|	Xetrov90002578m.L	-0.94	0.16	-2.26	***0.00***	-1.59	***0.00***
mi|Xelaev18020840m.g|	cttn-like.1.S	-0.92	0.09	-1.33	***0.01***	-0.63	0.17
mi|Xelaev18040332m.g|	crp.4.L	-0.91	0.04	-1.34	***0.00***	-0.89	0.03
mi|Xelaev18009842m.g|	myo1f.S	-0.91	0.09	-1.39	***0.00***	-0.36	0.45
mi|Xelaev18023671m.g|	grap2.L	-0.90	0.40	-2.85	***0.00***	-1.39	***0.01***
mi|Xelaev18018187m.g|	rab27a.L	-0.88	0.30	0.25	0.80	-2.16	***0.01***
mi|Xelaev18036965m.g|	pprc1-like.S	-0.85	0.05	1.44	***0.00***	0.74	0.09

mi|Xelaev18029410m.g|	nt5e.S	-0.84	0.48	-3.52	***0.01***	5.65	0.04
mi|Xelaev18019546m.g|	Xelaev18019546m.g	-0.82	0.17	-1.78	***0.00***	-0.49	0.31
mi|Xelaev18042386m.g|	degs3.S	-0.82	0.70	-0.64	0.62	-2.93	***0.00***
mi|Xelaev18042796m.g|	Xelaev18042796m.g	-0.82	0.70	-0.10	1.00	2.76	***0.00***
mi|Xelaev18041773m.g|	MGC75872.S	-0.80	0.30	-1.63	***0.00***	-0.55	0.33
mi|Xelaev18002190m.g|	Xelaev18002190m.g	-0.78	0.08	0.13	0.76	2.16	***0.00***
mi|Xelaev18023723m.g|	rac2.L	-0.77	0.11	-1.60	***0.00***	-0.41	0.35
mi|Xelaev18009890m.g|	gzmh.S	-0.77	0.43	-1.69	***0.00***	0.08	0.93
mi|Xelaev18043055m.g|	hck.L	-0.76	0.13	-1.35	***0.00***	-0.65	0.16
mi|Xelaev18025816m.g|	arhgdib.S	-0.76	0.09	-1.23	***0.00***	-0.79	0.06
mi|Xelaev18045691m.g|	phgdh.L	-0.75	0.08	1.37	***0.00***	0.20	0.64
mi|Xelaev18018108m.g|	ctsl.L	-0.74	0.13	-1.77	***0.00***	-0.48	0.29
mi|Xelaev18045688m.g|	Xelaev18045688m.g	-0.73	0.78	-3.48	***0.00***	-0.40	0.61
mi|Xelaev18044432m.g|	Xelaev18044432m.g	-0.73	0.78	2.94	***0.01***	0.91	0.48
mi|Xelaev18025498m.g|	ptprc.S	-0.73	0.15	-1.25	***0.01***	-0.04	0.95
mi|Xelaev18026393m.g|	tcte1.L	-0.73	0.11	0.62	0.17	1.43	***0.00***
mi|Xelaev18021926m.g|	spi1.L	-0.72	0.12	-1.51	***0.00***	-1.02	0.02
mi|Xelaev18006520m.g|	slc46a2.L	-0.71	0.38	-1.84	***0.01***	-1.20	0.04
mi|Xelaev18025949m.g|	loc100498463.S	-0.69	0.15	0.56	0.23	1.30	***0.01***
mi|Xelaev18036708m.g|	lrrc23.S	-0.67	0.19	0.28	0.58	1.39	***0.01***
mi|Xelaev18004183m.g|	c1qa.L	-0.65	0.12	-1.30	***0.00***	-0.88	0.03
mi|Xelaev18038248m.g|	Xelaev18038248m.g	-0.64	0.18	-1.20	***0.01***	-0.41	0.37
mi|Xelaev18030527m.g|	dhx30.L	-0.64	0.19	0.05	0.92	1.43	***0.00***
mi|Xelaev18011349m.g|	Xelaev18011349m.g	-0.61	0.52	-1.81	***0.01***	-0.24	0.78
mi|Xelaev18040797m.g|	il6r.L	-0.61	0.32	-1.80	***0.00***	-1.00	0.03
mi|Xelaev18018905m.g|	loc101731348.L	-0.60	0.31	-1.26	***0.01***	-1.10	0.01
mi|Xelaev18026073m.g|	trmt6.L	-0.58	0.19	1.18	***0.01***	0.18	0.68
mi|Xelaev18012140m.g|	slc30a2.L	-0.57	0.41	1.87	***0.00***	-0.31	0.58
mi|Xelaev18030893m.g|	asns.L	-0.57	0.18	1.15	***0.01***	0.30	0.48
mi|Xelaev18039168m.g|	Xelaev18039168m.g	-0.55	0.30	-1.69	***0.00***	0.05	1.00
mi|Xelaev18025712m.g|	sh3bp1.S	-0.53	0.46	-0.24	0.71	-1.43	***0.01***
mi|Xelaev18040509m.g|	sh2d2a.L	-0.52	0.62	-2.40	***0.00***	-1.49	0.06
mi|Xelaev18012079m.g|	loc100494491.L	-0.52	0.57	-0.56	0.57	-6.78	***0.00***
mi|Xelaev18003023m.g|	trex2.1	-0.52	0.65	-2.48	***0.00***	-0.26	0.69
mi|Xelaev18025607m.g|	selp.S	-0.52	0.65	3.26	***0.00***	-0.48	1.00
mi|Xelaev18037766m.g|	cxcr3L..S	-0.52	0.80	-3.15	***0.00***	-1.30	0.27
mi|Xelaev18012322m.g|	loc100492643.L	-0.52	1.00	-3.63	***0.00***	-0.91	0.24
mi|Xelaev18027954m.g|	Xelaev18027954m.g	-0.52	1.00	-3.54	***0.01***	-0.89	0.54
mi|Xelaev18011344m.g|	loc100127716.L	-0.52	1.00	-1.44	0.04	-1.90	***0.01***
mi|Xelaev18030636m.g|	loc100497570.L	-0.52	1.00	-0.84	0.14	-2.02	***0.00***
mi|Xelaev18043533m.g|	loc100492690.L	-0.51	1.00	-2.17	***0.00***	-0.54	0.45
mi|Xelaev18009421m.g|	ddit4l.S	-0.51	1.00	-2.90	***0.01***	0.48	1.00
mi|Xelaev18016295m.g|	krt2l.S	-0.51	1.00	2.79	***0.00***	-1.30	0.09

mi|Xelaev18002187m.g|	nmral1.S	-0.51	0.24	0.32	0.44	1.76	***0.00***
mi|Xelaev18005736m.g|	Xelaev18005736m.g	-0.50	0.40	2.15	***0.00***	0.61	0.21
mi|Xelaev18007075m.g|	loc100135349.L	-0.49	1.00	-7.46	***0.00***	0.00	1.00
mi|Xelaev18043879m.g|	loc100486088.L	-0.49	1.00	-2.58	***0.00***	-1.34	0.03
mi|Xelaev18029920m.g|	otos.S	-0.49	1.00	0.53	0.68	-2.09	***0.00***
mi|Xelaev18038657m.g|	MGC69466.L	-0.49	1.00	6.35	***0.00***	0.99	1.00
mi|Xelaev18011458m.g|	cbr1.L	-0.47	0.28	-0.25	0.57	1.39	***0.00***
mi|Xelaev18011935m.g|	epx.L	-0.46	0.29	-1.12	***0.01***	-0.64	0.15
mi|Xelaev18021364m.g|	hk2.S	-0.45	0.65	0.02	1.00	2.02	***0.00***
mi|Xelaev18037761m.g|	apoe.S	-0.45	0.51	-2.54	***0.00***	-2.08	***0.00***
mi|Xelaev18024536m.g|	loc101730972.S	-0.43	0.73	-1.05	0.19	-2.26	***0.00***
mi|Xelaev18042470m.g|	vars.S	-0.43	0.31	1.11	***0.01***	0.35	0.41
mi|Xelaev18011816m.g|	loc100489843-like.L	-0.42	0.70	-4.28	***0.00***	-0.82	0.19
mi|Xelaev18012845m.g|	Xelaev18012845m.g	-0.42	0.47	2.39	***0.00***	1.00	0.08
mi|Xelaev18016264m.g|	mmp13l.S	-0.41	0.43	0.45	0.31	1.37	***0.00***
mi|Xelaev18039707m.g|	ankrd9.L	-0.41	0.40	-1.83	***0.00***	0.65	0.19
mi|Xelaev18022675m.g|	irf8.L	-0.40	0.54	-1.80	***0.00***	-1.01	0.03
mi|Xelaev18012886m.g|	tlr7.L	-0.40	0.59	-1.64	***0.00***	-1.21	***0.01***
mi|Xelaev18020353m.g|	anxa4.S	-0.40	0.46	-1.50	***0.00***	-0.83	0.06
mi|Xelaev18010684m.g|	cmklr1.S	-0.39	0.67	-1.87	***0.00***	-1.18	0.01
mi|Xelaev18000008m.g|	bpi.S	-0.39	0.41	-1.61	***0.00***	-1.23	***0.00***
mi|Xelaev18006430m.g|	Xelaev18006430m.g	-0.39	0.54	0.18	0.84	1.99	***0.01***
mi|Xelaev18040648m.g|	tnfaip8l2.L	-0.38	0.45	-1.29	***0.01***	-0.87	0.06
mi|Xelaev18012283m.g|	csrp1.L	-0.38	0.76	1.77	***0.00***	0.19	0.77
mi|Xelaev18035976m.g|	loc100497009.L	-0.37	0.82	-0.88	0.30	-2.22	***0.00***
mi|Xelaev18033415m.g|	f13a1.S	-0.36	0.55	-1.57	***0.00***	-0.39	0.44
mi|Xelaev18029674m.g|	lamp3-like.S	-0.36	0.44	-1.31	***0.00***	-0.66	0.12
mi|Xelaev18038928m.g|	unnamed.L	-0.36	0.52	-2.56	***0.00***	-1.15	0.01
mi|Xelaev18036988m.g|	loc100493999-like.S	-0.35	0.51	-1.69	***0.00***	-1.19	0.03
mi|Xelaev18012406m.g|	gpr34.L	-0.35	0.49	-1.25	***0.01***	-1.07	0.02
mi|Xelaev18032905m.g|	loc100494322.S	-0.35	0.61	-2.24	***0.00***	0.30	0.63
mi|Xelaev18046887m.g|	Xelaev18046887m.g	-0.35	0.66	-2.96	***0.00***	0.07	1.00
mi|Xelaev18047607m.g|	Xelaev18047607m.g	-0.35	1.00	2.75	***0.00***	-4.67	0.26
mi|Xelaev18044138m.g|	Xelaev18044138m.g	-0.34	0.56	-1.00	0.04	-1.55	***0.00***
mi|Xelaev18012049m.g|	themis2.L	-0.33	0.86	-2.33	***0.00***	-1.09	0.11
mi|Xelaev18015387m.g|	ncf1.S	-0.33	0.86	-0.87	0.10	-1.33	***0.00***
mi|Xelaev18012100m.g|	sytl1.L	-0.33	1.00	-1.76	***0.01***	-0.24	0.84
mi|Xelaev18045690m.g|	Xelaev18045690m.g	-0.33	1.00	-4.34	***0.00***	-0.19	0.85
mi|Xelaev18002907m.g|	zbtb6.1	-0.33	1.00	-0.39	0.82	-2.75	***0.01***
mi|Xelaev18021244m.g|	epyc.S	-0.33	1.00	-0.29	0.65	-1.63	***0.00***
mi|Xelaev18042268m.g|	unnamed.S	-0.33	0.63	-1.31	***0.01***	-0.73	0.11
mi|Xelaev18007013m.g|	loc100486385.L	-0.32	0.77	2.16	***0.00***	0.01	1.00
mi|Xelaev18002547m.g|	Xelaev18002547m.g	-0.32	0.47	1.59	***0.00***	0.51	0.25

mi|Xelaev18027388m.g|	col9a1.L	-0.31	0.48	-0.45	0.30	-1.17	***0.01***
mi|Xelaev18046443m.g|	Xelaev18046443m.g	-0.30	0.60	-0.01	1.00	1.54	***0.00***
mi|Xelaev18039828m.g|	Xelaev18039828m.g	-0.30	0.84	3.02	***0.00***	0.32	1.00
mi|Xelaev18022482m.g|	osgin1.L	-0.28	0.66	1.60	***0.01***	-0.56	0.41
mi|Xelaev18011823m.g|	mpo.L	-0.28	0.53	-1.75	***0.00***	-0.71	0.11
mi|Xelaev18045642m.g|	loc101733921.L	-0.27	0.63	-1.45	***0.00***	-0.82	0.10
mi|Xelaev18025588m.g|	rgs1.S	-0.27	0.68	-1.45	***0.01***	-0.56	0.26
mi|Xelaev18030023m.g|	ccl34.S	-0.26	0.57	-0.05	0.93	2.45	***0.00***
mi|Xelaev18036896m.g|	blnk.S	-0.26	1.00	-3.54	***0.00***	-0.45	0.54
mi|Xelaev18030479m.g|	loc100485553.L	-0.26	1.00	0.33	1.00	-2.13	***0.00***
mi|Xelaev18038229m.g|	fut7.L	-0.25	0.80	-2.98	***0.00***	-1.87	***0.01***
mi|Xelaev18024485m.g|	f2.S	-0.21	0.90	-1.80	***0.00***	-0.08	0.91
mi|Xelaev18046858m.g|	Xelaev18046858m.g	-0.20	0.68	1.60	***0.00***	0.94	0.06
mi|Xelaev18004385m.g|	Xelaev18004385m.g	-0.20	1.00	-1.72	0.02	-2.85	***0.01***
mi|Xelaev18015101m.g|	rcc1.S	-0.19	0.69	1.31	***0.00***	0.51	0.27
mi|Xelaev18005016m.g|	spon2.L	-0.17	0.73	0.09	0.85	1.53	***0.00***
mi|Xelaev18046293m.g|	Xelaev18046293m.g	-0.17	0.83	0.30	0.61	1.72	***0.00***
mi|Xelaev18018909m.g|	loc100489257.L	-0.17	0.81	-1.43	***0.00***	-0.88	0.06
mi|Xelaev18040736m.g|	Xelaev18040736m.g	-0.17	0.74	-1.31	***0.00***	-1.03	0.02
mi|Xelaev18034327m.g|	loc101732585.L	-0.17	1.00	-3.12	***0.00***	-0.47	0.46
mi|Xelaev18019574m.g|	Xelaev18019574m.g	-0.16	0.92	-0.26	0.70	2.00	***0.00***
mi|Xelaev18045686m.g|	itgax.L	-0.14	0.75	-1.40	***0.00***	-1.17	***0.01***
mi|Xelaev18014899m.g|	trem2.S	-0.14	0.77	-1.65	***0.00***	-1.41	***0.00***
mi|Xelaev18006478m.g|	crlf1.L	-0.13	0.87	2.95	***0.00***	-0.84	1.00
mi|Xelaev18040568m.g|	Xelaev18040568m.g	-0.13	1.00	-1.11	0.39	-3.26	***0.01***
mi|Xelaev18028195m.g|	unnamed.L	-0.13	0.81	-1.48	***0.00***	0.66	0.25
mi|Xelaev18019323m.g|	Xelaev18019323m.g	-0.11	1.00	-0.78	0.28	1.87	***0.01***
mi|Xelaev18000322m.g|	Xetrov90017301m.1	-0.11	1.00	0.31	0.85	6.52	***0.00***
mi|Xelaev18029617m.g|	loc101732807.S	-0.10	0.86	-1.35	***0.01***	-0.37	0.43
mi|Xelaev18018927m.g|	Xelaev18018927m.g	-0.10	0.94	-1.51	***0.00***	-0.93	0.05
mi|Xelaev18017925m.g|	lep.L	-0.10	0.83	1.99	***0.00***	2.54	***0.00***
mi|Xelaev18026469m.g|	otor.L	-0.09	0.91	-1.44	***0.00***	-0.31	0.48
mi|Xelaev18040078m.g|	chac1.L	-0.09	0.84	1.17	***0.01***	0.35	0.44
mi|Xelaev18001309m.g|	Xelaev18001309m.g	-0.09	1.00	-1.89	***0.00***	-0.13	0.91
mi|Xelaev18040645m.g|	Xelaev18040645m.g	-0.09	1.00	-2.34	***0.01***	-1.30	0.07
mi|Xelaev18017091m.g|	loc100492943.L	-0.08	0.94	-1.96	***0.00***	-1.16	0.01
mi|Xelaev18018912m.g|	Xelaev18018912m.g	-0.06	1.00	-2.71	***0.00***	-1.01	0.10
mi|Xelaev18029163m.g|	Xetrov90013833m.S	-0.05	0.93	-1.20	***0.01***	-0.90	0.03
mi|Xelaev18031052m.g|	Xelaev18031052m.g	-0.04	1.00	3.33	***0.00***	0.70	0.45
mi|Xelaev18041824m.g|	Xelaev18041824m.g	-0.03	1.00	0.14	0.84	-1.34	***0.01***
mi|Xelaev18029904m.g|	inpp5d.S	-0.02	1.00	-1.52	***0.00***	-0.37	0.44
mi|Xelaev18010287m.g|	loc100495012.S	-0.02	1.00	-3.73	***0.00***	-1.02	0.24
mi|Xelaev18012950m.g|	loc100491382.L	0.00	1.00	-1.40	***0.01***	-1.22	0.01

mi|Xelaev18011155m.g|	aqp3.S	0.00	0.99	2.48	***0.00***	1.11	0.03
mi|Xelaev18001974m.g|	Xelaev18001974m.g	0.00	1.00	-1.33	***0.00***	-0.01	1.00
mi|Xelaev18002279m.g|	Xelaev18002279m.g	0.00	1.00	-7.48	***0.00***	0.00	1.00
mi|Xelaev18012119m.g|	Xetrov90004776m.L	0.00	1.00	-0.27	0.85	-2.00	***0.01***
mi|Xelaev18047968m.g|	Xelaev18047968m.g	0.00	1.00	-0.55	0.65	-3.49	***0.00***
mi|Xelaev18014944m.g|	elf3.S	0.00	1.00	0.81	0.59	-2.18	***0.00***
mi|Xelaev18035501m.g|	Xelaev18035501m.g	0.00	1.00	3.18	1.00	-6.88	***0.00***
mi|Xelaev18002773m.g|	Xelaev18002773m.g	0.00	1.00	3.91	***0.00***	4.89	0.14
mi|Xelaev18042717m.g|	cers2.S	0.00	1.00	-0.98	0.14	2.06	***0.01***
mi|Xelaev18036478m.g|	loc101735028.L	0.01	1.00	-1.09	0.39	-2.89	***0.00***
mi|Xelaev18042614m.g|	was.S	0.02	1.00	-1.26	***0.01***	-1.22	***0.01***
mi|Xelaev18023601m.g|	selp.L	0.02	1.00	2.36	***0.00***	1.51	0.17
mi|Xelaev18019679m.g|	Xelaev18019679m.g	0.02	1.00	-0.83	0.08	-1.22	***0.01***
mi|Xelaev18024674m.g|	Xelaev18024674m.g	0.03	0.96	1.34	***0.01***	0.70	0.18
mi|Xelaev18042217m.g|	sowahd.S	0.05	1.00	-2.28	***0.00***	-0.75	0.27
mi|Xelaev18038242m.g|	Xelaev18038242m.g	0.05	1.00	-1.79	***0.00***	-0.39	0.66
mi|Xelaev18046526m.g|	mmp9.S	0.06	0.89	-1.36	***0.00***	-0.39	0.37
mi|Xelaev18047462m.g|	Xelaev18047462m.g	0.09	0.84	-2.43	***0.00***	-0.44	0.45
mi|Xelaev18046133m.g|	ada.S	0.10	0.93	-1.77	***0.00***	-1.60	***0.00***
mi|Xelaev18038283m.g|	slc2a6.L	0.10	0.94	-0.52	0.30	-1.62	***0.00***
mi|Xelaev18034351m.g|	ptpn6.L	0.10	0.82	-1.34	***0.00***	-0.79	0.06
mi|Xelaev18026124m.g|	epcam.L	0.11	1.00	0.49	0.50	-3.09	***0.00***
mi|Xelaev18047323m.g|	pttg1ip.1.S	0.11	0.93	-2.37	***0.00***	-1.62	***0.00***
mi|Xelaev18040481m.g|	Xetrov90022231m.L	0.12	1.00	-2.13	0.09	-3.32	***0.01***
mi|Xelaev18040971m.g|	arhgap30.L	0.12	0.86	-1.63	***0.00***	-0.80	0.10
mi|Xelaev18008130m.g|	loc100491157.L	0.13	1.00	0.04	1.00	-2.49	***0.00***
mi|Xelaev18012541m.g|	loc100216114.L	0.14	0.86	-1.75	***0.01***	-1.48	***0.01***
mi|Xelaev18001597m.g|	Xelaev18001597m.g	0.15	0.80	-1.37	***0.01***	-1.46	***0.00***
mi|Xelaev18039831m.g|	Xelaev18039831m.g	0.16	0.80	2.19	***0.00***	-1.25	0.03
mi|Xelaev18009031m.g|	corin.S	0.18	0.91	-2.06	***0.00***	-0.10	0.86
mi|Xelaev18037095m.g|	oit3.S	0.19	0.80	-2.38	0.02	4.41	***0.00***
mi|Xelaev18043850m.g|	tmc8.L	0.20	1.00	-2.11	0.02	-2.85	***0.01***
mi|Xelaev18025577m.g|	apold1.S	0.20	0.86	-0.82	0.29	-2.28	***0.01***
mi|Xelaev18012028m.g|	Xelaev18012028m.g	0.21	0.69	-1.19	***0.01***	-0.73	0.14
mi|Xelaev18035153m.g|	loc100493672.L	0.22	0.80	-3.00	***0.00***	-0.94	0.08
mi|Xelaev18024949m.g|	irf8.S	0.24	0.64	-1.31	***0.01***	-0.55	0.22
mi|Xelaev18043803m.g|	c17orf67.L	0.24	1.00	-1.19	0.05	-3.31	***0.00***
mi|Xelaev18022524m.g|	cetp.L	0.26	0.63	-1.31	***0.01***	0.90	0.09
mi|Xelaev18006268m.g|	misp.L	0.26	0.64	-1.31	***0.01***	-0.25	0.77
mi|Xelaev18036079m.g|	loc733245.L	0.27	0.73	-2.15	***0.00***	-1.27	0.02
mi|Xelaev18012841m.g|	loc100498345.L	0.27	1.00	6.21	***0.01***	-4.06	0.51
mi|Xelaev18030549m.g|	Xelaev18030549m.g	0.29	0.58	-1.61	***0.00***	-0.92	0.17
mi|Xelaev18019232m.g|	Xelaev18019232m.g	0.29	1.00	-3.27	***0.00***	-1.73	0.01

mi|Xelaev18045096m.g|	Xelaev18045096m.g	0.29	0.51	-1.19	***0.01***	-0.15	0.76
mi|Xelaev18040753m.g|	nr1i3.L	0.30	0.69	2.11	***0.00***	-0.82	0.18
mi|Xelaev18013599m.g|	itgb7.L	0.30	0.86	-1.99	***0.00***	-6.15	***0.01***
mi|Xelaev18002230m.g|	Xelaev18002230m.g	0.32	0.72	-0.12	0.84	2.10	***0.00***
mi|Xelaev18034835m.g|	oit3.L	0.32	0.48	1.16	0.01	1.27	***0.01***
mi|Xelaev18047336m.g|	cd28.S	0.32	0.73	-0.91	0.10	-1.44	***0.01***
mi|Xelaev18015722m.g|	Xelaev18015722m.g	0.33	0.61	2.01	***0.00***	0.42	0.46
mi|Xelaev18043551m.g|	Xetrov90029403m.L	0.34	0.55	1.39	***0.01***	-0.53	0.37
mi|Xelaev18002241m.g|	Xelaev18002241m.g	0.34	0.42	-1.64	***0.00***	0.84	0.05
mi|Xelaev18018616m.g|	nt5dc4.L	0.34	0.45	-1.36	***0.00***	0.30	0.56
mi|Xelaev18047455m.g|	Xelaev18047455m.g	0.35	0.39	-1.49	***0.00***	0.11	0.82
mi|Xelaev18039829m.g|	myh8-like.L	0.36	0.62	1.54	***0.00***	-1.11	0.04
mi|Xelaev18013483m.g|	Xelaev18013483m.g	0.39	0.62	-1.65	***0.01***	0.41	0.48
mi|Xelaev18012851m.g|	evi2b.L	0.40	0.58	-1.93	***0.00***	-0.77	0.15
mi|Xelaev18010114m.g|	Xelaev18010114m.g	0.41	0.73	0.33	0.70	-2.46	***0.00***
mi|Xelaev18047463m.g|	Xelaev18047463m.g	0.41	0.36	-2.08	***0.00***	-1.00	0.11
mi|Xelaev18026609m.g|	loc100491567.L	0.42	0.33	1.49	***0.00***	0.11	0.81
mi|Xelaev18035141m.g|	loc100491969.L	0.42	0.51	-1.01	0.07	1.88	***0.00***
mi|Xelaev18014009m.g|	Xelaev18014009m.g	0.44	0.41	-1.29	***0.01***	-0.16	0.81
mi|Xelaev18026314m.g|	capn1.L	0.44	1.00	-2.15	***0.00***	-3.04	***0.00***
mi|Xelaev18026634m.g|	mall.L	0.44	1.00	-0.95	0.54	-2.21	***0.01***
mi|Xelaev18047951m.g|	gbp7.S	0.44	1.00	-0.94	0.70	-2.18	***0.00***
mi|Xelaev18020541m.g|	ttll13.S	0.44	0.37	-0.57	0.26	1.62	***0.00***
mi|Xelaev18047454m.g|	Xelaev18047454m.g	0.44	0.30	-1.61	***0.00***	-0.48	0.45
mi|Xelaev18045098m.g|	Xelaev18045098m.g	0.45	0.28	-1.42	***0.00***	-0.25	0.54
mi|Xelaev18015636m.g|	itgbl1.S	0.45	1.00	2.63	***0.00***	0.07	1.00
mi|Xelaev18004858m.g|	loc100486585.L	0.46	0.45	-1.96	***0.00***	-1.21	***0.01***
mi|Xelaev18014832m.g|	Xelaev18014832m.g	0.46	0.82	-0.94	0.70	6.52	***0.00***
mi|Xelaev18020415m.g|	itga11.S	0.46	0.60	-0.03	1.00	2.34	***0.00***
mi|Xelaev18025053m.g|	dnase2b.S	0.47	0.47	-1.76	***0.00***	-1.21	***0.01***
mi|Xelaev18023577m.g|	rgs1.L	0.47	0.35	-1.53	***0.00***	-0.32	0.49
mi|Xelaev18008128m.g|	naip.L	0.48	0.83	0.08	1.00	-1.80	***0.01***
mi|Xelaev18009945m.g|	loc101731250.S	0.53	0.24	-1.22	***0.01***	-1.04	0.01
mi|Xelaev18032360m.g|	fam84b.L	0.54	0.25	0.68	0.17	1.58	***0.00***
mi|Xelaev18007448m.g|	sds.L	0.55	0.18	-1.11	***0.01***	0.15	0.72
mi|Xelaev18047451m.g|	hbd.S	0.56	0.17	-1.24	***0.00***	-0.03	0.95
mi|Xelaev18006310m.g|	acer1.L	0.57	0.54	-2.40	***0.00***	0.54	0.35
mi|Xelaev18013693m.g|	frem2.L	0.57	0.28	0.81	0.16	1.56	***0.00***
mi|Xelaev18019580m.g|	loc100496720.L	0.57	0.17	-1.23	***0.00***	-0.74	0.08
mi|Xelaev18046765m.g|	Xelaev18046765m.g	0.59	0.49	-1.89	***0.00***	-0.14	1.00
mi|Xelaev18008621m.g|	Xelaev18008621m.g	0.61	0.60	3.08	***0.00***	-0.04	1.00
mi|Xelaev18037666m.g|	loc100494342.S	0.61	0.34	-0.53	0.28	-1.58	***0.00***
mi|Xelaev18003937m.g|	Xelaev18003937m.g	0.63	0.68	-2.59	***0.00***	-0.87	0.16

mi|Xelaev18014389m.g|	cd86.S	0.63	0.68	-2.80	***0.00***	-1.02	0.12
mi|Xelaev18034532m.g|	Xelaev18034532m.g	0.63	0.68	-2.90	***0.01***	0.25	1.00
mi|Xelaev18013901m.g|	ctsc.L	0.64	0.13	-1.28	***0.00***	-0.44	0.29
mi|Xelaev18030640m.g|	Xetrov90028682m.L	0.65	0.65	2.85	***0.01***	-0.91	0.21
mi|Xelaev18018338m.g|	Xetrov90008914m.L	0.66	0.40	-2.21	***0.01***	-0.23	0.84
mi|Xelaev18029422m.g|	irf6.S	0.67	0.38	2.20	***0.00***	-0.65	0.35
mi|Xelaev18032923m.g|	slc39a12.S	0.68	0.31	-1.58	***0.01***	-0.17	0.76
mi|Xelaev18006518m.g|	loc100487203.L	0.68	0.62	-1.83	0.02	-1.78	***0.01***
mi|Xelaev18018928m.g|	loc101735287.L	0.69	0.25	-1.69	***0.01***	-1.12	0.05
mi|Xelaev18043661m.g|	socs3.L	0.71	0.09	0.75	0.08	1.22	***0.01***
mi|Xelaev18044327m.g|	loc101735213.L	0.74	0.31	-3.96	***0.00***	-0.04	1.00
mi|Xelaev18004366m.g|	Xelaev18004366m.g	0.74	0.14	-0.75	0.12	-1.46	***0.00***
mi|Xelaev18028344m.g|	loc100494572.L	0.76	0.38	2.07	0.04	6.96	***0.00***
mi|Xelaev18047036m.g|	fev.S	0.76	0.31	-2.52	***0.01***	-0.11	0.89
mi|Xelaev18003075m.g|	MGC108117.L	0.77	0.10	0.10	0.84	-1.20	***0.01***
mi|Xelaev18037390m.g|	tagln.S	0.77	0.09	2.31	***0.00***	0.36	0.70
mi|Xelaev18019764m.g|	Xelaev18019764m.g	0.77	0.54	1.55	0.33	-2.68	***0.00***
mi|Xelaev18009901m.g|	pram1.S	0.78	0.14	-1.33	***0.01***	-0.42	0.41
mi|Xelaev18036267m.g|	btg5.L	0.78	0.12	1.55	0.02	2.07	***0.00***
mi|Xelaev18012177m.g|	Xelaev18012177m.g	0.78	0.17	-2.13	***0.00***	-0.94	0.09
mi|Xelaev18043629m.g|	tnfrsf6b.L	0.78	0.35	3.56	***0.01***	-1.80	0.40
mi|Xelaev18046900m.g|	loc100492872.S	0.79	0.25	-2.05	***0.00***	1.08	0.06
mi|Xelaev18007824m.g|	morn3.L	0.80	0.14	-1.49	***0.00***	0.44	0.41
mi|Xelaev18001864m.g|	pparg.L	0.81	0.33	-2.85	***0.00***	0.59	0.43
mi|Xelaev18033828m.g|	fabp4.S	0.81	0.15	-1.31	0.01	1.53	***0.01***
mi|Xelaev18030909m.g|	vwde.L	0.83	0.13	-1.33	***0.01***	0.05	0.92
mi|Xelaev18018863m.g|	plekha2.L	0.84	0.06	-1.21	***0.01***	-0.36	0.40
mi|Xelaev18003860m.g|	Xelaev18003860m.g	0.85	0.48	0.71	0.47	-6.68	***0.00***
mi|Xelaev18045085m.g|	hbg2.L	0.86	0.04	-1.82	***0.00***	-0.06	0.88
mi|Xelaev18018730m.g|	adrb3.L	0.86	0.08	-1.29	***0.01***	-0.67	0.14
mi|Xelaev18032381m.g|	sla.L	0.87	0.06	-1.40	***0.00***	-0.88	0.05
mi|Xelaev18022778m.g|	loc100498398.L	0.87	0.48	-3.63	***0.00***	1.73	0.06
mi|Xelaev18023293m.g|	frrs1.L	0.87	0.11	-1.73	***0.00***	0.08	1.00
mi|Xelaev18009669m.g|	ebi3.S	0.88	0.25	-1.03	0.06	-1.70	***0.00***
mi|Xelaev18018337m.g|	cemip.L	0.90	0.04	-1.13	***0.01***	0.23	0.59
mi|Xelaev18023567m.g|	apold1.L	0.93	0.14	0.63	0.35	-1.65	***0.01***
mi|Xelaev18039832m.g|	Xelaev18039832m.g	0.93	1.00	2.06	***0.00***	0.11	1.00
mi|Xelaev18010932m.g|	Xelaev18010932m.g	0.94	0.44	-6.59	***0.00***	-2.59	0.09
mi|Xelaev18003995m.g|	Xelaev18003995m.g	0.94	0.44	6.61	***0.00***	-1.47	0.19
mi|Xelaev18002945m.g|	Xelaev18002945m.g	0.94	0.44	1.00	0.44	-3.77	***0.00***
mi|Xelaev18019026m.g|	loc100489278-like.L	1.00	0.09	-1.50	***0.01***	-0.44	0.40
mi|Xelaev18025458m.g|	loc100492113.S	1.00	1.00	-2.02	***0.01***	-1.19	0.48
mi|Xelaev18043641m.g|	Xelaev18043641m.g	1.00	1.00	-1.05	0.17	-3.37	***0.00***

mi|Xelaev18029975m.g|	Xelaev18029975m.g	1.00	0.02	0.44	0.29	1.21	***0.00***
mi|Xelaev18017462m.g|	epyc.L	1.02	0.10	0.06	1.00	-1.71	***0.00***
mi|Xelaev18047889m.g|	eln2.S	1.04	0.37	0.43	0.82	-5.47	***0.00***
mi|Xelaev18027040m.g|	tagap.L	1.04	0.19	-1.50	0.03	-2.18	***0.00***
mi|Xelaev18015840m.g|	prph.S	1.07	***0.01***	-0.54	0.19	-0.14	0.74
mi|Xelaev18003264m.g|	nppc.L	1.08	***0.01***	-0.26	0.53	0.09	0.83
mi|Xelaev18034566m.g|	bnip3.L	1.09	***0.01***	-0.11	0.79	-0.26	0.53
mi|Xelaev18034521m.g|	htra1.L	1.09	***0.01***	0.61	0.15	0.60	0.16
mi|Xelaev18002242m.g|	Xelaev18002242m.g	1.10	0.04	-1.74	***0.00***	-0.24	0.83
mi|Xelaev18040563m.g|	Xelaev18040563m.g	1.10	0.05	-1.99	***0.00***	-0.06	1.00
mi|Xelaev18007069m.g|	slc7a7.L	1.10	0.01	-1.10	0.01	-1.61	***0.00***
mi|Xelaev18038363m.g|	stom-like.L	1.11	***0.01***	-0.16	0.70	-0.06	0.89
mi|Xelaev18027826m.g|	mterf4.L	1.11	***0.01***	-0.37	0.38	-0.03	0.95
mi|Xelaev18012516m.g|	meaf6.L	1.12	***0.01***	-0.07	0.88	-0.37	0.39
mi|Xelaev18005777m.g|	ccser1.L	1.12	***0.01***	-0.17	0.68	-0.24	0.57
mi|Xelaev18000374m.g|	Xelaev18000374m.g	1.12	0.32	-6.34	***0.00***	-1.28	0.21
mi|Xelaev18043101m.g|	hap1.L	1.12	***0.01***	-0.35	0.40	-0.05	0.90
mi|Xelaev18019674m.g|	gps2.L	1.12	***0.01***	-0.13	0.76	0.15	0.73
mi|Xelaev18044932m.g|	dnah7.L	1.13	***0.01***	-0.56	0.18	0.21	0.61
mi|Xelaev18036025m.g|	apoc1-like.L	1.13	***0.01***	-1.12	***0.01***	-0.78	0.06
mi|Xelaev18038290m.g|	sardh.L	1.13	***0.01***	-0.79	0.06	0.05	0.90
mi|Xelaev18001086m.g|	gps2.S	1.13	***0.01***	-0.30	0.46	0.00	0.99
mi|Xelaev18037809m.g|	xilr2.S	1.14	0.12	-0.97	0.09	-1.84	***0.00***
mi|Xelaev18033922m.g|	klf10.S	1.14	***0.01***	-0.14	0.74	0.08	0.85
mi|Xelaev18012652m.g|	loc100490302.L	1.14	***0.01***	-0.26	0.55	0.24	0.57
mi|Xelaev18039412m.g|	zfp36.L	1.14	***0.01***	-0.78	0.07	-0.03	0.96
mi|Xelaev18041412m.g|	slc47a2.S	1.14	***0.01***	-0.48	0.26	0.21	0.62
mi|Xelaev18010477m.g|	pik3ip1.S	1.14	***0.01***	-0.51	0.23	-0.35	0.41
mi|Xelaev18014018m.g|	art5.L	1.14	0.01	-1.84	***0.00***	-0.91	0.03
mi|Xelaev18006700m.g|	svep1.L	1.14	***0.01***	0.05	0.91	0.12	0.77
mi|Xelaev18019862m.g|	cecr1.S	1.15	***0.01***	-0.67	0.11	-0.25	0.55
mi|Xelaev18046783m.g|	foxk2.S	1.15	***0.01***	-0.28	0.49	-0.14	0.73
mi|Xelaev18019047m.g|	Xelaev18019047m.g	1.15	0.12	-1.70	***0.01***	-0.64	0.34
mi|Xelaev18013275m.g|	lrrc63-like.L	1.16	***0.01***	-0.23	0.60	0.19	0.67
mi|Xelaev18042189m.g|	loc100485432.S	1.16	0.21	0.71	0.47	-2.81	***0.00***
mi|Xelaev18045318m.g|	aldob.L	1.16	***0.01***	-0.61	0.14	-0.05	0.90
mi|Xelaev18022884m.g|	wdr63.L	1.16	***0.01***	-0.94	0.03	0.68	0.11
mi|Xelaev18006882m.g|	klf9.L	1.16	***0.01***	0.00	1.00	0.42	0.32
mi|Xelaev18032810m.g|	wdr86.S	1.16	***0.01***	-0.21	0.64	0.57	0.20
mi|Xelaev18001487m.g|	unnamed.L	1.16	***0.01***	-0.16	0.70	0.01	0.98
mi|Xelaev18021845m.g|	cd44.L	1.17	***0.01***	-0.33	0.43	-0.04	0.92
mi|Xelaev18001657m.g|	Xetrov90004318m.1	1.17	***0.01***	0.00	1.00	0.00	1.00
mi|Xelaev18034594m.g|	cyp26a1.L	1.17	0.06	-0.42	0.64	1.70	***0.00***

mi|Xelaev18005623m.g|	cyp2u1.L	1.17	***0.01***	-0.37	0.38	-0.42	0.32
mi|Xelaev18038781m.g|	smarca1.L	1.17	***0.01***	-0.29	0.50	-0.34	0.43
mi|Xelaev18008992m.g|	klf3.S	1.18	***0.01***	-0.03	0.97	0.03	0.98
mi|Xelaev18026338m.g|	fosl2.L	1.18	***0.01***	0.01	1.00	1.16	***0.01***
mi|Xelaev18007986m.g|	cdo1.L	1.18	***0.00***	-0.88	0.03	-0.30	0.47
mi|Xelaev18005591m.g|	larp1b.L	1.18	***0.00***	-0.53	0.20	-0.12	0.77
mi|Xelaev18043400m.g|	Xelaev18043400m.g	1.18	***0.01***	-0.51	0.23	0.06	0.90
mi|Xelaev18041751m.g|	itih6.S	1.19	0.02	-1.22	***0.01***	0.28	0.55
mi|Xelaev18046734m.g|	Xelaev18046734m.g	1.19	***0.00***	0.00	1.00	-0.05	0.90
mi|Xelaev18011288m.g|	loc100490688.L	1.19	***0.01***	-0.24	0.57	-0.17	0.69
mi|Xelaev18017444m.g|	lrriq1.L	1.19	***0.01***	-0.45	0.30	0.02	0.98
mi|Xelaev18044604m.g|	dapl1.L	1.19	***0.00***	-0.94	0.02	-0.09	0.82
mi|Xelaev18025448m.g|	Xelaev18025448m.g	1.19	***0.01***	0.27	0.56	0.29	0.54
mi|Xelaev18016709m.g|	kdm7a.L	1.20	***0.00***	-0.54	0.20	-0.04	0.92
mi|Xelaev18045307m.g|	Xelaev18045307m.g	1.20	0.04	1.77	***0.00***	-0.63	0.33
mi|Xelaev18042589m.g|	zfp36.S	1.20	***0.00***	-0.11	0.79	0.05	0.91
mi|Xelaev18004106m.g|	caps2.L	1.20	***0.01***	-0.51	0.27	0.01	1.00
mi|Xelaev18028620m.g|	adck3.S	1.20	***0.00***	-0.63	0.13	-0.14	0.74
mi|Xelaev18016367m.g|	prcp.S	1.20	***0.00***	-0.26	0.53	-0.19	0.65
mi|Xelaev18031550m.g|	kdsr.L	1.21	***0.00***	-0.22	0.59	-0.10	0.81
mi|Xelaev18009120m.g|	snx25-like.S	1.21	0.10	-0.31	0.73	2.16	***0.01***
mi|Xelaev18037000m.g|	loc100493658.S	1.21	***0.01***	-0.80	0.08	-0.31	0.48
mi|Xelaev18038146m.g|	timp-1.L	1.21	***0.00***	1.56	***0.00***	1.21	***0.01***
mi|Xelaev18046174m.g|	cebpb.S	1.22	***0.01***	0.14	0.76	0.15	0.76
mi|Xelaev18027477m.g|	bcl6.L	1.22	***0.00***	-0.20	0.64	0.22	0.60
mi|Xelaev18023656m.g|	cbx7.L	1.22	***0.01***	-0.32	0.51	0.01	1.00
mi|Xelaev18017111m.g|	myot.L	1.22	***0.00***	0.20	0.63	-0.40	0.34
mi|Xelaev18023809m.g|	mst1.L	1.23	***0.00***	0.29	0.49	1.11	***0.01***
mi|Xelaev18040399m.g|	txnip.L	1.23	***0.00***	-0.63	0.13	-0.06	0.88
mi|Xelaev18012717m.g|	loc548774.L	1.23	***0.01***	-0.40	0.37	-0.15	0.73
mi|Xelaev18031383m.g|	dnah5.L	1.23	***0.00***	-0.45	0.28	0.19	0.65
mi|Xelaev18018457m.g|	iqch.L	1.23	***0.00***	-0.49	0.25	0.30	0.48
mi|Xelaev18046264m.g|	ctsz.S	1.24	***0.00***	-0.46	0.26	-0.35	0.40
mi|Xelaev18039809m.g|	lrrc9.L	1.24	***0.00***	-0.47	0.27	0.01	0.99
mi|Xelaev18021489m.g|	fnip1.S	1.24	***0.01***	-0.03	0.96	-0.10	0.84
mi|Xelaev18043673m.g|	dcxr.L	1.24	***0.00***	-0.44	0.29	0.01	0.98
mi|Xelaev18001795m.g|	loc407755.L	1.25	0.27	-2.08	0.09	-2.85	***0.01***
mi|Xelaev18029292m.g|	Xelaev18029292m.g	1.25	***0.01***	-0.32	0.51	-0.20	0.67
mi|Xelaev18017258m.g|	stk32a.L	1.25	***0.00***	-0.38	0.37	-0.42	0.32
mi|Xelaev18032213m.g|	snx31.L	1.25	***0.01***	-0.28	0.54	-0.11	0.84
mi|Xelaev18037836m.g|	ppp1r15a.S	1.26	***0.00***	-0.70	0.10	0.08	0.85
mi|Xelaev18022000m.g|	ifitm1.L	1.27	***0.00***	-0.56	0.19	-0.55	0.19
mi|Xelaev18017252m.g|	crebrf-like.L	1.27	***0.01***	-0.62	0.19	0.10	0.83

mi|Xelaev18017176m.g|	loc101731999.L	1.27	***0.01***	-0.39	0.41	-0.45	0.31
mi|Xelaev18006345m.g|	mknk2.L	1.27	***0.00***	-0.29	0.49	0.00	0.99
mi|Xelaev18038347m.g|	Xelaev18038347m.g	1.27	***0.00***	-0.88	0.04	0.97	0.02
mi|Xelaev18046379m.g|	loc100496782.S	1.27	***0.00***	-0.80	0.07	0.15	0.73
mi|Xelaev18042722m.g|	ctss.S	1.27	***0.00***	-0.57	0.17	-0.76	0.07
mi|Xelaev18030882m.g|	pdk4.L	1.27	***0.00***	-0.46	0.26	0.18	0.67
mi|Xelaev18041279m.g|	atg2b.S	1.28	***0.00***	-0.31	0.45	-0.02	0.95
mi|Xelaev18022531m.g|	Xelaev18022531m.g	1.28	***0.00***	-0.17	0.67	-0.25	0.55
mi|Xelaev18038035m.g|	tyrobp.S	1.28	0.02	-1.55	***0.00***	-0.40	0.39
mi|Xelaev18043867m.g|	tnnc2.L	1.28	0.02	1.97	***0.00***	-0.13	0.82
mi|Xelaev18045304m.g|	Xelaev18045304m.g	1.28	0.33	6.92	***0.00***	0.83	1.00
mi|Xelaev18032679m.g|	loc733184.S	1.28	***0.00***	-0.43	0.33	0.21	0.62
mi|Xelaev18040619m.g|	Xelaev18040619m.g	1.29	***0.01***	0.22	0.65	-0.62	0.17
mi|Xelaev18000342m.g|	Xelaev18000342m.g	1.29	0.10	-1.54	***0.00***	1.22	0.11
mi|Xelaev18032161m.g|	cpne3.L	1.29	***0.00***	-0.47	0.26	-0.02	0.97
mi|Xelaev18027995m.g|	fbxo25.L	1.29	***0.00***	-0.21	0.62	-0.20	0.64
mi|Xelaev18019135m.g|	loc101733102.L	1.29	0.24	3.45	***0.01***	0.72	0.55
mi|Xelaev18027237m.g|	lin28b.L	1.29	***0.00***	-0.23	0.62	-0.11	0.82
mi|Xelaev18040384m.g|	Xelaev18040384m.g	1.30	***0.00***	-0.66	0.12	-0.16	0.71
mi|Xelaev18007577m.g|	pik3ip1.L	1.30	***0.00***	-0.77	0.07	-0.34	0.41
mi|Xelaev18008362m.g|	ranbp3l-like.L	1.30	***0.00***	-0.02	0.97	0.25	0.57
mi|Xelaev18039910m.g|	sptb.L	1.30	***0.01***	-1.21	***0.01***	0.03	0.96
mi|Xelaev18037048m.g|	cpn1.S	1.31	0.07	-1.71	***0.00***	-0.37	0.56
mi|Xelaev18023478m.g|	Xelaev18023478m.g	1.31	***0.00***	0.59	0.18	0.20	0.66
mi|Xelaev18003184m.g|	loc100485662.1.L	1.31	***0.00***	-0.42	0.32	-0.07	0.86
mi|Xelaev18038577m.g|	renbp.L	1.31	***0.00***	-0.37	0.37	-0.29	0.48
mi|Xelaev18047609m.g|	Xelaev18047609m.g	1.31	0.03	1.54	***0.00***	-0.71	0.16
mi|Xelaev18013408m.g|	rdh7.L	1.31	***0.00***	-0.39	0.37	0.34	0.44
mi|Xelaev18045016m.g|	ankar.L	1.31	***0.01***	-0.56	0.29	0.73	0.15
mi|Xelaev18039999m.g|	actc1.L	1.32	***0.00***	0.40	0.37	-0.11	0.81
mi|Xelaev18032762m.g|	wnt9a.S	1.32	***0.01***	0.08	0.95	-0.39	0.45
mi|Xelaev18014029m.g|	prcp.L	1.33	***0.00***	-0.63	0.14	-0.48	0.26
mi|Xelaev18043284m.g|	slc4a1.L	1.33	***0.00***	-0.80	0.08	-0.50	0.32
mi|Xelaev18031061m.g|	nek10.L	1.33	***0.01***	-0.05	0.93	0.08	0.92
mi|Xelaev18018391m.g|	arrdc4.L	1.33	***0.00***	-0.77	0.08	-0.34	0.44
mi|Xelaev18039402m.g|	nccrp1.L	1.34	***0.00***	-0.10	0.84	0.02	1.00
mi|Xelaev18039849m.g|	lgals3.L	1.34	***0.00***	0.32	0.49	-0.26	0.56
mi|Xelaev18043510m.g|	loc101731830.L	1.34	***0.01***	-0.16	0.77	0.14	0.78
mi|Xelaev18022474m.g|	Xelaev18022474m.g	1.34	***0.00***	-0.13	0.81	-0.41	0.36
mi|Xelaev18023681m.g|	tef.L	1.34	***0.00***	0.00	1.00	-0.19	0.66
mi|Xelaev18038813m.g|	lhfpl1.L	1.34	***0.00***	0.10	0.81	-0.16	0.73
mi|Xelaev18036758m.g|	acta2.S	1.34	***0.00***	-0.51	0.24	0.82	0.08
mi|Xelaev18015277m.g|	Xelaev18015277m.g	1.35	***0.01***	-0.10	0.90	-0.12	0.80

mi|Xelaev18038046m.g|	Xelaev18038046m.g	1.35	***0.01***	-0.35	0.49	0.25	0.66
mi|Xelaev18005527m.g|	hhip.L	1.35	***0.00***	-0.49	0.24	-0.23	0.58
mi|Xelaev18046523m.g|	loc100494738.S	1.36	0.13	3.24	***0.00***	-0.24	0.84
mi|Xelaev18009396m.g|	npnt.S	1.36	***0.00***	-0.14	0.77	0.48	0.27
mi|Xelaev18042598m.g|	gata1.S	1.37	***0.01***	-0.50	0.30	-0.26	0.82
mi|Xelaev18047856m.g|	mylpf.S	1.38	***0.01***	0.83	0.08	-0.50	0.30
mi|Xelaev18028179m.g|	loc100490476.L	1.38	***0.00***	-1.14	0.01	-0.16	0.73
mi|Xelaev18020076m.g|	Xelaev18020076m.g	1.39	***0.01***	0.06	1.00	-0.83	0.15
mi|Xelaev18016717m.g|	loc100488229.L	1.39	***0.00***	0.37	0.41	0.37	0.42
mi|Xelaev18038251m.g|	Xelaev18038251m.g	1.39	0.02	1.20	0.16	6.23	***0.01***
mi|Xelaev18045645m.g|	mylpf.L	1.39	***0.01***	0.91	0.05	-0.60	0.20
mi|Xelaev18011171m.g|	vwa5a-like.S	1.39	***0.00***	-0.33	0.43	0.04	0.93
mi|Xelaev18044099m.g|	Xelaev18044099m.g	1.39	***0.00***	-0.12	0.77	0.13	0.76
mi|Xelaev18022528m.g|	Xelaev18022528m.g	1.39	***0.00***	0.48	0.25	-0.19	0.64
mi|Xelaev18011324m.g|	creg1.L	1.40	***0.00***	-1.02	0.02	-0.13	0.77
mi|Xelaev18018854m.g|	loc100490587.L	1.40	0.22	-2.14	***0.01***	-0.24	1.00
mi|Xelaev18000380m.g|	Xelaev18000380m.g	1.40	***0.00***	-0.06	0.94	0.50	0.27
mi|Xelaev18006949m.g|	ctsv.L	1.40	***0.00***	-0.67	0.10	0.05	0.90
mi|Xelaev18007970m.g|	lox.L	1.40	***0.00***	0.25	0.56	0.15	0.73
mi|Xelaev18032678m.g|	Xelaev18032678m.g	1.40	***0.01***	-0.68	0.18	0.39	0.40
mi|Xelaev18005528m.g|	Xetrov90000949m.L	1.40	***0.00***	-0.20	0.66	-0.34	0.43
mi|Xelaev18035724m.g|	loc101730276.L	1.40	***0.01***	-1.21	0.03	-0.80	0.11
mi|Xelaev18043994m.g|	tldc2.L	1.41	0.48	-2.33	***0.00***	-0.91	0.24
mi|Xelaev18010639m.g|	rhof.S	1.41	0.48	3.45	***0.01***	-2.18	0.06
mi|Xelaev18042112m.g|	Xelaev18042112m.g	1.41	***0.00***	-0.14	0.74	0.18	0.69
mi|Xelaev18019317m.g|	loc100494987-like.L	1.41	***0.00***	-0.30	0.51	-0.07	0.87
mi|Xelaev18021438m.g|	Xelaev18021438m.g	1.41	***0.00***	-0.42	0.34	-0.02	0.97
mi|Xelaev18010384m.g|	sds.S	1.41	***0.00***	-1.23	***0.00***	-0.09	0.84
mi|Xelaev18047615m.g|	rsph10b.S	1.41	***0.00***	-0.65	0.14	0.29	0.50
mi|Xelaev18013332m.g|	prph.L	1.41	***0.00***	-0.13	0.74	0.08	0.85
mi|Xelaev18016494m.g|	Xelaev18016494m.g	1.42	***0.00***	-0.49	0.24	0.02	0.97
mi|Xelaev18025951m.g|	cidec.S	1.42	***0.00***	-0.61	0.17	-0.13	0.78
mi|Xelaev18042946m.g|	srms.S	1.42	***0.00***	-0.53	0.21	0.20	0.64
mi|Xelaev18033400m.g|	loc100488932.S	1.43	0.22	1.21	0.39	-2.64	***0.00***
mi|Xelaev18029914m.g|	klhl24.S	1.43	***0.00***	-0.73	0.10	-0.22	0.62
mi|Xelaev18032260m.g|	klf10.L	1.43	***0.00***	-0.17	0.69	0.00	1.00
mi|Xelaev18009386m.g|	sgms2.S	1.44	***0.01***	-0.56	0.28	0.88	0.11
mi|Xelaev18031046m.g|	nr1d2.L	1.44	***0.00***	0.26	0.57	-0.61	0.18
mi|Xelaev18007471m.g|	ube3b.L	1.45	***0.00***	-0.68	0.16	0.44	0.34
mi|Xelaev18034344m.g|	c1s.L	1.45	***0.00***	0.61	0.17	0.28	0.53
mi|Xelaev18047510m.g|	myh11.S	1.45	***0.00***	-0.01	1.00	-0.42	0.39
mi|Xelaev18005035m.g|	loc100492954.L	1.45	***0.00***	0.16	0.74	-0.74	0.10
mi|Xelaev18043832m.g|	Xelaev18043832m.g	1.45	***0.00***	-0.59	0.15	0.14	0.73

mi|Xelaev18045100m.g|	Xelaev18045100m.g	1.46	0.04	-2.10	***0.00***	0.99	1.00
mi|Xelaev18010386m.g|	slc8b1.S	1.46	***0.00***	-0.53	0.28	-0.39	0.43
mi|Xelaev18036115m.g|	ppp1r15a.L	1.46	***0.00***	-0.75	0.08	-0.22	0.61
mi|Xelaev18017962m.g|	loc100498358.L	1.47	***0.01***	0.52	0.33	-0.02	1.00
mi|Xelaev18043345m.g|	march10.L	1.47	***0.00***	-0.08	0.90	0.37	0.49
mi|Xelaev18011164m.g|	ccl19.S	1.47	***0.00***	1.15	0.05	1.07	0.21
mi|Xelaev18022532m.g|	Xetrov90011361m.L	1.47	***0.00***	1.30	***0.00***	-0.25	0.60
mi|Xelaev18023481m.g|	Xelaev18023481m.g	1.47	***0.00***	0.91	0.03	0.44	0.29
mi|Xelaev18034005m.g|	trib1.S	1.47	***0.00***	0.68	0.17	0.13	0.82
mi|Xelaev18025655m.g|	cbx7.S	1.47	***0.00***	-0.04	0.94	0.08	0.87
mi|Xelaev18007501m.g|	hps4.L	1.47	***0.00***	0.05	0.91	-0.18	0.69
mi|Xelaev18021194m.g|	gns.S	1.49	***0.00***	-0.26	0.53	0.32	0.44
mi|Xelaev18008260m.g|	loc100496633-like.L	1.49	***0.00***	-0.34	0.48	0.10	0.84
mi|Xelaev18029762m.g|	ptx3.S	1.49	***0.00***	-0.85	0.08	-0.19	0.71
mi|Xelaev18015701m.g|	ednrb.S	1.50	***0.01***	0.04	1.00	0.43	0.45
mi|Xelaev18031468m.g|	fam134b.L	1.50	***0.00***	-0.66	0.12	-0.21	0.61
mi|Xelaev18022274m.g|	fosl1.L	1.50	***0.00***	1.50	***0.01***	0.81	0.40
mi|Xelaev18028160m.g|	clic4.L	1.51	0.01	-1.58	***0.00***	-0.50	0.65
mi|Xelaev18034029m.g|	sla.S	1.51	***0.00***	-1.53	***0.00***	-0.93	0.03
mi|Xelaev18045087m.g|	loc100485964-like.L	1.51	***0.00***	0.11	0.83	0.23	0.61
mi|Xelaev18032617m.g|	loc100491922-like.S	1.51	***0.01***	-0.74	0.13	-0.65	0.17
mi|Xelaev18016664m.g|	Xetrov90026715m.L	1.51	***0.00***	0.50	0.25	0.74	0.09
mi|Xelaev18018420m.g|	pcsk6.L	1.52	***0.00***	-0.14	0.74	0.38	0.37
mi|Xelaev18045301m.g|	Xelaev18045301m.g	1.52	***0.01***	1.31	***0.01***	-0.34	0.50
mi|Xelaev18027773m.g|	loc100145763.L	1.52	***0.00***	-0.29	0.49	-0.14	0.75
mi|Xelaev18027530m.g|	cpn2.L	1.53	***0.01***	-2.09	***0.00***	1.34	0.13
mi|Xelaev18009128m.g|	casp3.S	1.53	***0.00***	-0.24	0.58	-0.35	0.42
mi|Xelaev18043110m.g|	krt19.L	1.54	***0.00***	-0.28	0.49	0.19	0.65
mi|Xelaev18032342m.g|	fbxo32.L	1.55	***0.00***	-0.03	1.00	-0.29	0.55
mi|Xelaev18044792m.g|	Xetrov90024114m.L	1.55	***0.00***	-0.38	0.39	-0.09	0.84
mi|Xelaev18042743m.g|	txnip.S	1.56	***0.00***	-0.63	0.13	0.04	0.91
mi|Xelaev18016908m.g|	loc100491991-like.L	1.56	***0.00***	-2.35	***0.00***	-1.37	***0.00***
mi|Xelaev18002528m.g|	crebl2.L	1.56	***0.00***	-0.61	0.15	-0.48	0.26
mi|Xelaev18036380m.g|	Xetrov90013771m.L	1.57	***0.00***	-0.01	1.00	0.23	0.63
mi|Xelaev18004051m.g|	azin2.S	1.57	***0.00***	0.20	0.64	-0.11	0.79
mi|Xelaev18027866m.g|	klhl24.L	1.58	***0.00***	-0.45	0.29	-0.34	0.42
mi|Xelaev18023934m.g|	irak2.L	1.58	***0.00***	-0.91	0.04	-0.59	0.18
mi|Xelaev18022676m.g|	dusp22a.L	1.59	***0.00***	-0.53	0.30	-0.13	0.81
mi|Xelaev18020523m.g|	cemip.S	1.59	***0.00***	-0.73	0.10	0.55	0.21
mi|Xelaev18020469m.g|	arrdc4.S	1.59	***0.00***	-0.71	0.10	-0.28	0.53
mi|Xelaev18011859m.g|	Xetrov90028149m.L	1.59	0.03	6.42	***0.00***	0.48	0.52
mi|Xelaev18007618m.g|	Xelaev18007618m.g	1.60	***0.00***	0.50	0.24	-0.34	0.44
mi|Xelaev18034956m.g|	glud1.L	1.60	***0.00***	-0.50	0.23	-0.26	0.53

mi|Xelaev18017179m.g|	Xelaev18017179m.g	1.60	***0.00***	-0.14	0.78	-0.53	0.25
mi|Xelaev18038157m.g|	loc100488699-like.L	1.61	***0.00***	0.44	0.34	-0.08	0.90
mi|Xelaev18023824m.g|	mst1r.L	1.62	***0.00***	-0.42	0.48	0.40	0.51
mi|Xelaev18006177m.g|	tex15.L	1.62	***0.00***	-0.49	0.32	-0.16	0.76
mi|Xelaev18019543m.g|	asgr2.L	1.63	***0.00***	0.65	0.34	0.25	0.90
mi|Xelaev18013717m.g|	kl.L	1.63	***0.00***	0.24	0.74	-0.33	0.53
mi|Xelaev18016451m.g|	Xelaev18016451m.g	1.64	***0.00***	-0.99	0.02	0.19	0.64
mi|Xelaev18015543m.g|	lyg2.S	1.64	***0.00***	1.20	***0.00***	0.59	0.17
mi|Xelaev18019549m.g|	Xelaev18019549m.g	1.65	***0.01***	-1.52	***0.00***	-1.08	0.03
mi|Xelaev18017672m.g|	asb15.L	1.66	***0.00***	-1.07	0.04	-0.58	0.26
mi|Xelaev18044199m.g|	loc100493941-like.L	1.66	***0.00***	-0.49	0.40	-0.67	0.35
mi|Xelaev18008195m.g|	bhmt.L	1.67	***0.00***	-0.55	0.24	0.54	0.27
mi|Xelaev18021871m.g|	loc100497319-like.L	1.67	0.06	-2.33	***0.00***	-0.53	0.33
mi|Xelaev18014552m.g|	Xelaev18014552m.g	1.67	***0.00***	-0.33	0.58	-0.26	0.73
mi|Xelaev18035721m.g|	loc100490200.L	1.67	***0.00***	-0.57	0.21	-0.56	0.21
mi|Xelaev18047342m.g|	loc100495429.S	1.68	***0.00***	0.37	0.51	0.32	0.49
mi|Xelaev18033990m.g|	fbxo32.S	1.68	***0.00***	-0.42	0.35	-0.25	0.57
mi|Xelaev18039392m.g|	loc100486294.L	1.68	***0.00***	-0.84	0.12	0.37	0.49
mi|Xelaev18006954m.g|	Xelaev18006954m.g	1.68	***0.01***	-0.22	0.71	0.13	0.85
mi|Xelaev18008304m.g|	itga2.L	1.69	***0.00***	0.79	0.09	0.42	0.39
mi|Xelaev18002612m.g|	Xelaev18002612m.g	1.69	***0.00***	-0.66	0.18	-0.10	0.87
mi|Xelaev18020777m.g|	stra6.S	1.70	***0.01***	-0.92	0.15	0.16	0.81
mi|Xelaev18040562m.g|	loc100488729.L	1.70	0.06	-1.95	0.02	-1.79	***0.00***
mi|Xelaev18026662m.g|	cyp1b1.L	1.72	***0.00***	-0.15	0.77	0.03	0.95
mi|Xelaev18031303m.g|	loc100489129.L	1.72	0.33	2.19	***0.00***	-1.19	0.07
mi|Xelaev18019125m.g|	crb3.L	1.72	***0.01***	-0.06	1.00	-0.68	0.26
mi|Xelaev18021479m.g|	rad50.S	1.73	***0.01***	-0.08	0.93	-1.59	0.02
mi|Xelaev18011812m.g|	cldn3.L	1.73	***0.00***	0.34	0.70	0.75	0.20
mi|Xelaev18044524m.g|	slc11a1.L	1.74	***0.00***	-0.40	0.47	-0.21	0.84
mi|Xelaev18013919m.g|	Xelaev18013919m.g	1.74	***0.00***	0.98	0.04	0.35	0.52
mi|Xelaev18003910m.g|	Xelaev18003910m.g	1.75	***0.00***	-1.18	***0.01***	0.25	0.55
mi|Xelaev18042550m.g|	prss16.S	1.76	***0.00***	-0.90	0.04	-0.73	0.09
mi|Xelaev18033700m.g|	cebpd.S	1.76	***0.00***	-0.47	0.25	0.57	0.17
mi|Xelaev18011870m.g|	loc100497109.L	1.77	***0.00***	-0.70	0.18	1.48	0.01
mi|Xelaev18032982m.g|	pdk4.S	1.77	***0.00***	-0.87	0.04	0.05	0.90
mi|Xelaev18021413m.g|	crebrf-like.S	1.77	***0.00***	-0.66	0.13	-0.29	0.50
mi|Xelaev18011016m.g|	lmbrd2.S	1.78	***0.00***	-0.23	0.72	-1.16	0.02
mi|Xelaev18031843m.g|	rab31.L	1.78	***0.01***	0.02	1.00	-0.18	0.80
mi|Xelaev18042115m.g|	loc100492639-like.S	1.79	***0.00***	-0.96	0.03	-0.11	0.82
mi|Xelaev18031576m.g|	Xelaev18031576m.g	1.81	***0.01***	-0.62	0.25	-0.54	0.35
mi|Xelaev18035884m.g|	cd163l1.L	1.81	***0.01***	0.29	0.73	-0.81	0.23
mi|Xelaev18035923m.g|	tmc4.L	1.81	***0.01***	-0.25	0.70	0.33	0.60
mi|Xelaev18031490m.g|	Xelaev18031490m.g	1.81	***0.01***	0.69	0.34	0.24	0.80

mi|Xelaev18004020m.g|	Xelaev18004020m.g	1.82	0.64	-0.25	1.00	-6.48	***0.00***
mi|Xelaev18040482m.g|	Xelaev18040482m.g	1.82	***0.00***	-0.86	0.07	-0.54	0.26
mi|Xelaev18001304m.g|	Xelaev18001304m.g	1.82	***0.01***	-1.19	0.08	-0.40	1.00
mi|Xelaev18046859m.g|	cacng4.S	1.83	***0.01***	-0.94	0.12	-0.92	0.13
mi|Xelaev18000330m.g|	Xelaev18000330m.g	1.83	***0.00***	-0.66	0.13	-0.01	0.98
mi|Xelaev18025760m.g|	mst1.S	1.84	***0.00***	-0.31	0.48	1.27	***0.00***
mi|Xelaev18029453m.g|	Xelaev18029453m.g	1.84	0.03	2.31	***0.01***	-0.91	0.21
mi|Xelaev18031280m.g|	aoah.L	1.84	***0.00***	-0.78	0.10	-0.36	0.43
mi|Xelaev18011456m.g|	Xelaev18011456m.g	1.85	***0.00***	-1.33	0.03	-1.87	***0.00***
mi|Xelaev18029940m.g|	rbp2.S	1.86	0.03	-3.41	***0.01***	0.31	0.72
mi|Xelaev18022529m.g|	Xelaev18022529m.g	1.88	***0.00***	-0.39	0.35	-0.57	0.17
mi|Xelaev18028378m.g|	scara3.L	1.88	***0.00***	-0.38	0.38	0.05	0.91
mi|Xelaev18036272m.g|	Xelaev18036272m.g	1.89	***0.00***	-0.08	0.88	-0.96	0.06
mi|Xelaev18025422m.g|	clca3p-like.S	1.89	***0.00***	0.98	0.03	0.54	0.26
mi|Xelaev18037808m.g|	Xelaev18037808m.g	1.89	0.04	-1.36	0.27	-2.61	***0.00***
mi|Xelaev18014604m.g|	loc100494383.S	1.89	***0.00***	0.29	0.66	-0.30	0.58
mi|Xelaev18047126m.g|	cd302.S	1.90	***0.00***	-0.13	0.82	0.25	0.69
mi|Xelaev18046185m.g|	nfatc2.S	1.90	***0.00***	-0.54	0.28	0.70	0.15
mi|Xelaev18047606m.g|	Xelaev18047606m.g	1.91	***0.01***	1.25	0.02	-0.20	0.78
mi|Xelaev18043069m.g|	loc101732252.L	1.91	***0.01***	0.93	0.26	0.33	1.00
mi|Xelaev18036024m.g|	apoe.L	1.91	***0.00***	-1.13	***0.01***	-1.39	***0.00***
mi|Xelaev18035274m.g|	Xelaev18035274m.g	1.92	***0.00***	-0.39	0.43	0.35	0.48
mi|Xelaev18002752m.g|	rnf182.S	1.93	***0.01***	-0.67	0.35	-1.08	0.10
mi|Xelaev18046048m.g|	slc4a1.S	1.93	***0.00***	-0.45	0.37	-0.18	0.81
mi|Xelaev18014838m.g|	mybph.S	1.94	***0.00***	0.12	0.90	-0.83	0.22
mi|Xelaev18031989m.g|	cebpd.L	1.95	***0.00***	-0.59	0.15	0.77	0.06
mi|Xelaev18039459m.g|	fos.L	1.95	***0.00***	0.67	0.23	-1.30	***0.00***
mi|Xelaev18010102m.g|	Xelaev18010102m.g	1.96	***0.00***	-0.56	0.33	0.03	1.00
mi|Xelaev18044446m.g|	Xelaev18044446m.g	1.98	***0.00***	-0.95	0.08	-1.39	0.02
mi|Xelaev18039005m.g|	loc100127779.L	1.98	***0.00***	-0.67	0.11	-0.31	0.46
mi|Xelaev18002404m.g|	Xelaev18002404m.g	1.99	***0.00***	-1.26	0.04	-0.18	0.85
mi|Xelaev18036829m.g|	htra1.S	1.99	***0.00***	1.33	***0.00***	0.91	0.03
mi|Xelaev18035813m.g|	Xelaev18035813m.g	2.01	***0.00***	0.39	0.73	-0.20	0.76
mi|Xelaev18004158m.g|	Xelaev18004158m.g	2.02	***0.00***	-0.74	0.11	-0.69	0.11
mi|Xelaev18025526m.g|	Xelaev18025526m.g	2.02	***0.00***	0.16	0.84	-0.10	1.00
mi|Xelaev18038264m.g|	Xelaev18038264m.g	2.03	***0.00***	-0.58	0.16	-0.19	0.65
mi|Xelaev18045569m.g|	rasd1.L	2.03	***0.00***	-0.32	0.49	-0.20	0.67
mi|Xelaev18010391m.g|	selplg.S	2.03	***0.00***	-0.65	0.29	0.08	0.91
mi|Xelaev18045021m.g|	mstn.1.L	2.03	***0.00***	0.37	0.75	1.62	0.19
mi|Xelaev18005146m.g|	cd38.L	2.05	***0.00***	-0.48	0.26	0.25	0.56
mi|Xelaev18021265m.g|	ptprq.S	2.08	***0.00***	-0.35	0.55	-0.78	0.14
mi|Xelaev18034760m.g|	ankrd1.L	2.10	***0.00***	0.43	0.61	0.14	0.91
mi|Xelaev18036353m.g|	Xelaev18036353m.g	2.10	***0.00***	-0.68	0.17	0.26	0.61

mi|Xelaev18032917m.g|	Xelaev18032917m.g	2.10	***0.01***	4.82	0.26	0.57	0.78
mi|Xelaev18018360m.g|	rlbp1.L	2.14	***0.00***	-0.55	0.29	0.72	0.19
mi|Xelaev18041485m.g|	Xelaev18041485m.g	2.14	***0.00***	-0.64	0.12	-0.19	0.65
mi|Xelaev18030844m.g|	loc100145516.L	2.15	***0.00***	0.73	0.32	1.70	0.02
mi|Xelaev18020261m.g|	loc100496891.S	2.16	***0.00***	-0.62	0.29	-0.76	0.17
mi|Xelaev18037324m.g|	adamts15.S	2.18	***0.00***	-0.10	0.92	0.40	0.62
mi|Xelaev18010923m.g|	cmya5.S	2.18	***0.00***	-0.16	0.84	-0.05	0.95
mi|Xelaev18038973m.g|	il13ra2.L	2.19	***0.00***	-1.27	0.04	0.52	0.64
mi|Xelaev18045355m.g|	pdilt.L	2.20	***0.00***	-0.65	0.25	0.37	0.46
mi|Xelaev18013909m.g|	loc100490389-like.L	2.20	0.14	-3.41	***0.01***	0.08	1.00
mi|Xelaev18002953m.g|	Xelaev18002953m.g	2.20	0.14	1.96	0.14	3.50	***0.01***
mi|Xelaev18008062m.g|	rfesd.L	2.22	***0.00***	-0.54	0.21	0.06	0.89
mi|Xelaev18025007m.g|	loc100497464.S	2.22	***0.00***	1.13	0.07	0.61	0.31
mi|Xelaev18009050m.g|	lrrc66.S	2.24	***0.00***	0.59	0.24	-0.02	1.00
mi|Xelaev18012630m.g|	Xelaev18012630m.g	2.25	***0.00***	1.12	0.04	0.49	0.53
mi|Xelaev18030326m.g|	Xelaev18030326m.g	2.25	***0.01***	0.92	0.18	0.25	0.90
mi|Xelaev18036286m.g|	loc100488841.L	2.26	***0.00***	-0.60	0.31	0.28	0.88
mi|Xelaev18027313m.g|	loc100487665-like.L	2.26	***0.00***	0.14	0.78	-0.06	0.90
mi|Xelaev18003389m.g|	atp2a1.L	2.26	***0.00***	1.88	***0.00***	1.09	0.05
mi|Xelaev18009358m.g|	loc100493381.S	2.27	***0.00***	1.27	0.06	-0.96	0.13
mi|Xelaev18000853m.g|	serpine1.S	2.29	***0.00***	0.14	0.92	0.45	0.73
mi|Xelaev18045310m.g|	loc100170419.L	2.31	0.25	2.08	***0.00***	0.38	0.77
mi|Xelaev18000041m.g|	Xelaev18000041m.g	2.32	***0.00***	0.69	0.62	0.40	0.84
mi|Xelaev18009995m.g|	Xelaev18009995m.g	2.33	***0.00***	-1.05	0.01	0.01	0.98
mi|Xelaev18017497m.g|	amdhd1.L	2.33	***0.00***	-0.51	0.31	1.74	***0.00***
mi|Xelaev18031194m.g|	inhba.L	2.34	***0.00***	0.92	0.18	-0.24	0.84
mi|Xelaev18030932m.g|	gpnmb.L	2.36	***0.00***	-0.11	0.80	-0.98	0.02
mi|Xelaev18004278m.g|	Xelaev18004278m.g	2.36	***0.00***	-0.38	1.00	-0.69	0.44
mi|Xelaev18012471m.g|	htr1d.L	2.36	***0.00***	-0.48	0.56	-1.28	0.05
mi|Xelaev18017011m.g|	loc101734840.L	2.36	***0.00***	3.56	***0.01***	-0.53	0.46
mi|Xelaev18016518m.g|	Xelaev18016518m.g	2.40	***0.00***	0.33	0.61	0.45	0.43
mi|Xelaev18030843m.g|	steap4.L	2.42	***0.00***	0.26	0.58	0.34	0.47
mi|Xelaev18042591m.g|	unnamed.S	2.43	***0.01***	0.26	0.87	-0.46	0.49
mi|Xelaev18038926m.g|	tmem182-like.2.L	2.44	***0.00***	-0.56	0.47	-0.50	0.47
mi|Xelaev18034150m.g|	slc39a4.S	2.45	***0.00***	1.11	***0.01***	0.10	0.82
mi|Xelaev18027311m.g|	Xelaev18027311m.g	2.45	***0.00***	-0.03	0.95	0.26	0.58
mi|Xelaev18003789m.g|	Xelaev18003789m.g	2.45	***0.00***	-0.81	0.07	0.16	0.70
mi|Xelaev18024125m.g|	Xelaev18024125m.g	2.51	***0.00***	-0.97	0.27	0.27	1.00
mi|Xelaev18024546m.g|	adm.S	2.52	***0.00***	0.02	1.00	-0.26	0.79
mi|Xelaev18004252m.g|	Xelaev18004252m.g	2.53	***0.00***	0.79	0.07	0.93	0.05
mi|Xelaev18003557m.g|	Xelaev18003557m.g	2.53	***0.01***	1.37	0.10	0.96	0.35
mi|Xelaev18034942m.g|	ldb3-like.1.L	2.54	***0.00***	-0.53	0.35	-0.65	0.28
mi|Xelaev18018897m.g|	loc100489110.L	2.57	***0.00***	0.60	0.27	0.63	0.20

mi|Xelaev18020464m.g|	Xelaev18020464m.g	2.57	***0.00***	-0.77	0.59	-0.18	0.86
mi|Xelaev18010995m.g|	oxct1.S	2.60	***0.00***	-1.17	0.39	-2.68	***0.00***
mi|Xelaev18034736m.g|	rbp4.L	2.61	***0.00***	-1.02	0.02	-0.19	0.67
mi|Xelaev18039176m.g|	cfb.L	2.62	***0.00***	0.46	0.27	1.58	***0.00***
mi|Xelaev18029882m.g|	Xelaev18029882m.g	2.63	0.15	-3.48	***0.00***	-1.10	0.16
mi|Xelaev18043321m.g|	mylk.L	2.63	0.15	-3.41	***0.01***	0.08	1.00
mi|Xelaev18041128m.g|	dhrs7.S	2.63	0.15	0.69	0.62	-1.91	***0.00***
mi|Xelaev18019918m.g|	ano2.S	2.63	***0.01***	1.00	0.44	-0.06	1.00
mi|Xelaev18022081m.g|	Xelaev18022081m.g	2.63	***0.01***	1.40	0.27	-0.07	1.00
mi|Xelaev18009049m.g|	Xelaev18009049m.g	2.64	***0.00***	0.95	0.11	0.15	0.82
mi|Xelaev18005354m.g|	slc25a4.L	2.64	***0.00***	0.95	0.21	-1.20	0.11
mi|Xelaev18014352m.g|	fam3b.S	2.66	***0.00***	0.35	0.46	-0.91	0.05
mi|Xelaev18037722m.g|	xa-1-like.S	2.67	***0.00***	0.21	1.00	1.06	0.37
mi|Xelaev18026176m.g|	Xelaev18026176m.g	2.70	***0.00***	0.16	0.90	-0.21	0.80
mi|Xelaev18014488m.g|	mab21l3.S	2.71	***0.00***	-1.14	0.06	1.91	***0.00***
mi|Xelaev18039339m.g|	olfm4.L	2.73	***0.00***	1.34	***0.00***	1.16	***0.01***
mi|Xelaev18025859m.g|	irak2.S	2.77	***0.00***	-2.83	0.06	0.63	1.00
mi|Xelaev18009501m.g|	Xelaev18009501m.g	2.78	***0.00***	0.31	0.64	0.59	0.31
mi|Xelaev18036931m.g|	Xelaev18036931m.g	2.84	***0.01***	-5.90	0.02	-1.63	0.04
mi|Xelaev18007437m.g|	MGC75582.L	2.84	***0.01***	0.64	0.47	-1.13	0.18
mi|Xelaev18026247m.g|	marc1-like.L	2.84	***0.01***	-0.30	0.84	0.65	0.65
mi|Xelaev18041860m.g|	lhx6.S	2.86	***0.00***	-0.76	0.28	0.65	0.42
mi|Xelaev18019129m.g|	c3.L	2.93	***0.00***	0.84	0.04	2.40	***0.00***
mi|Xelaev18027022m.g|	Xelaev18027022m.g	2.97	***0.00***	-0.94	0.70	0.08	1.00
mi|Xelaev18041151m.g|	gpr132.S	2.97	***0.00***	-0.15	0.90	0.26	0.89
mi|Xelaev18009500m.g|	cxcl8.S	2.97	***0.00***	2.87	0.06	0.87	0.59
mi|Xelaev18043989m.g|	Xelaev18043989m.g	2.99	***0.01***	-4.34	0.51	-0.39	1.00
mi|Xelaev18008772m.g|	cyp26b1.S	3.19	***0.00***	-0.34	0.83	-0.63	0.38
mi|Xelaev18021416m.g|	stc2.S	3.19	***0.00***	-0.29	1.00	-0.63	0.38
mi|Xelaev18011936m.g|	cideb.L	3.27	***0.00***	0.00	1.00	0.63	1.00
mi|Xelaev18006826m.g|	rflb.L	3.29	0.03	3.03	***0.00***	0.08	1.00
mi|Xelaev18043836m.g|	g6pc-like.1.L	3.32	***0.00***	-1.59	***0.00***	0.29	0.50
mi|Xelaev18035519m.g|	Xelaev18035519m.g	3.33	***0.00***	6.35	***0.00***	0.00	1.00
mi|Xelaev18009504m.g|	Xelaev18009504m.g	3.36	***0.00***	1.41	0.03	0.20	1.00
mi|Xelaev18034628m.g|	col17a1.L	3.38	***0.00***	1.17	0.04	-0.75	0.27
mi|Xelaev18013488m.g|	loc496972.L	3.40	1.00	-2.93	***0.00***	-0.76	0.29
mi|Xelaev18007073m.g|	ighx.L	3.40	1.00	-6.01	0.01	-6.56	***0.00***
mi|Xelaev18046507m.g|	g6pc-like.1.S	3.47	***0.00***	-1.99	***0.00***	0.05	1.00
mi|Xelaev18038397m.g|	c9orf9.L	3.55	***0.00***	0.02	1.00	2.01	0.14
mi|Xelaev18002519m.g|	Xelaev18002519m.g	3.65	***0.00***	-0.54	0.65	0.96	0.30
mi|Xelaev18001530m.g|	prss53.L	3.70	***0.00***	-1.53	0.19	-1.98	0.02
mi|Xelaev18046620m.g|	Xelaev18046620m.g	3.75	***0.01***	-0.50	0.60	0.53	0.70
mi|Xelaev18009482m.g|	loc100271753.S	3.85	***0.00***	2.15	***0.00***	0.80	0.09

mi|Xelaev18016263m.g|	mmp1.S	3.95	***0.00***	3.18	***0.00***	1.00	0.24
mi|Xelaev18028931m.g|	loc100490133.S	3.99	***0.00***	-1.41	0.17	4.68	0.26
mi|Xelaev18034823m.g|	Xelaev18034823m.g	4.09	***0.00***	2.31	***0.01***	-0.55	0.49
mi|Xelaev18013990m.g|	hpx.L	4.18	***0.00***	4.26	***0.00***	5.65	0.04
mi|Xelaev18043858m.g|	loc100494738.L	4.19	***0.00***	1.77	***0.00***	0.49	0.82
mi|Xelaev18013829m.g|	mmp13l.L	4.26	***0.00***	2.03	***0.00***	1.80	***0.00***
mi|Xelaev18045368m.g|	Xelaev18045368m.g	4.28	***0.00***	5.10	***0.00***	2.26	0.25
mi|Xelaev18013823m.g|	mmp8.L	4.39	***0.00***	4.52	***0.00***	0.55	0.80
mi|Xelaev18032949m.g|	loc100145516.S	4.40	***0.00***	0.02	1.00	2.04	0.03
mi|Xelaev18036097m.g|	loc100489674.L	4.80	***0.00***	0.94	1.00	0.07	1.00
mi|Xelaev18027359m.g|	loc100497103.L	4.89	0.26	-1.36	0.27	-3.30	***0.00***
mi|Xelaev18021463m.g|	Xelaev18021463m.g	5.29	0.08	-3.41	***0.01***	-0.33	0.82
mi|Xelaev18018930m.g|	loc100489908.L	5.33	0.08	-5.47	0.08	-6.52	***0.00***
mi|Xelaev18037094m.g|	pla2g12b.S	5.35	0.08	6.40	***0.00***	-1.23	0.09
mi|Xelaev18011861m.g|	loc100496785.L	6.27	***0.01***	0.02	1.00	0.08	1.00
mi|Xelaev18016883m.g|	Xelaev18016883m.g	6.44	***0.00***	-5.60	0.04	-0.43	0.68
mi|Xelaev18005827m.g|	cxcl8.L	6.44	***0.00***	1.66	0.05	1.24	0.15
mi|Xelaev18043108m.g|	krt17.L	6.44	***0.00***	3.18	1.00	0.07	1.00
mi|Xelaev18010739m.g|	gramd3.S	6.59	***0.00***	0.60	0.57	-0.27	0.75
mi|Xelaev18006861m.g|	Xelaev18006861m.g	6.85	***0.00***	3.18	1.00	-0.71	0.59

Supplementary Table S8 - Antibodies

Target	#Cat		WB	IFI	Methanol (IFI)
p-S6 (S235/236)	Cell-Signaling	2211	1:1000	1:200	Yes
S6	Cell-Signaling	2217	1:1000	1:200	
p-4E-BP1 (T37/46)	Cell-Signaling	2855	1:1000	1:100	Yes
4E-BP1	Cell-Signaling	9644	1:1000	1:100	
SOX2	Cell-Signaling	4900S	1:1000	1:200	
PCNA	Sigma-Aldrich	P8825		1:100	
Neurofilament-200	Sigma-Aldrich	N4142		1:1000	
α-Tubulina	Abcam	ab7291	1:10000		
Alexa 488	Invitrogen	A11001		1:500	
Alexa 555	Invitrogen	A21422		1:500	
Alexa 635	Invitrogen	A31577		1:500	
IgG-Mouse, HRP	Invitrogen	32430	1:2000		
IgG-Rabbit, HRP	Invitrogen	31460	1:2000		

Uncropped images of blots included in main figures

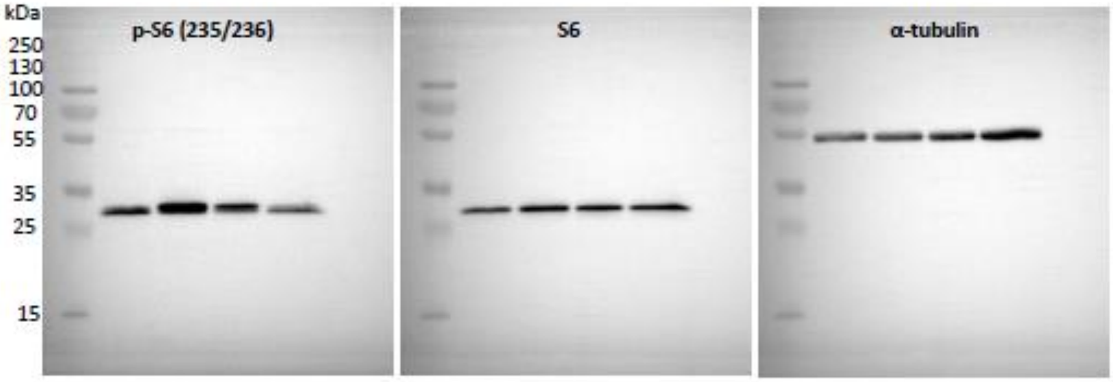

The images correspond to the western blot included in figure 4f.

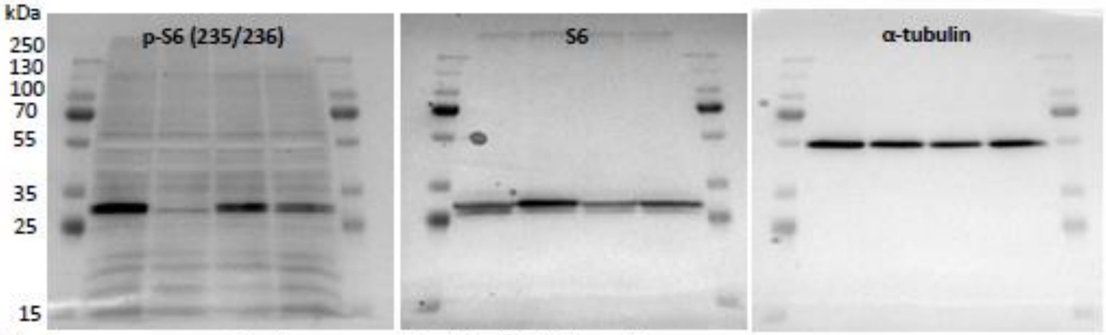

The images correspond to the western blot included in figure 5b.

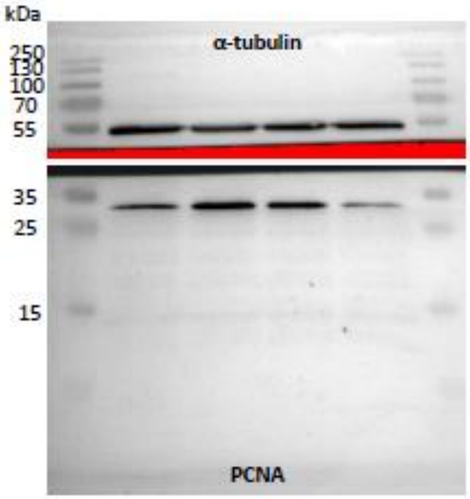

Both images are part of a unique membrane divided in two, each section of the membrane was incubated with different antibodies, as specified. These images correspond to the western blot included in figure 7q.

Uncropped images of blots included in supplementary information

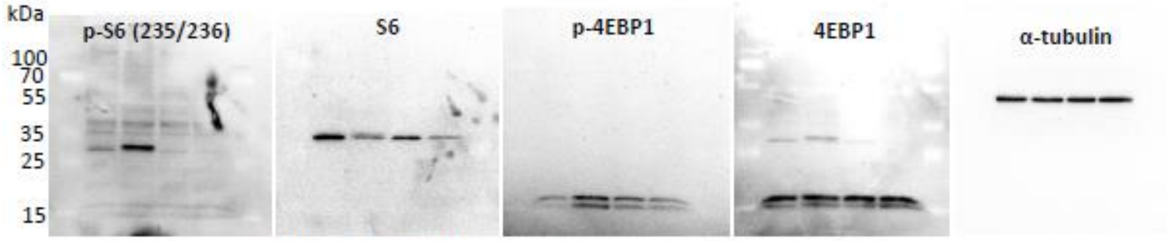

The images correspond to the western blot included in figure S3b.

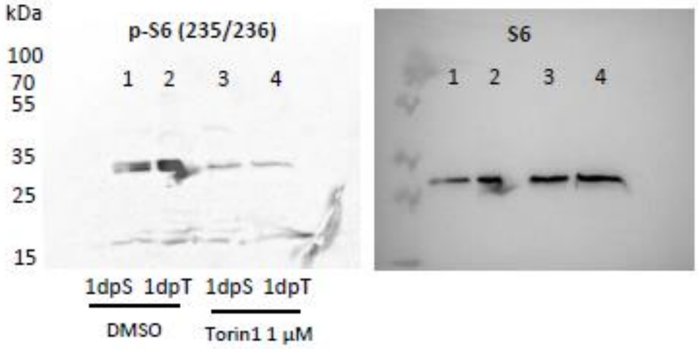

Lanes 1 and 3 correspond to sham animals, and are the ones included in figure S3e.

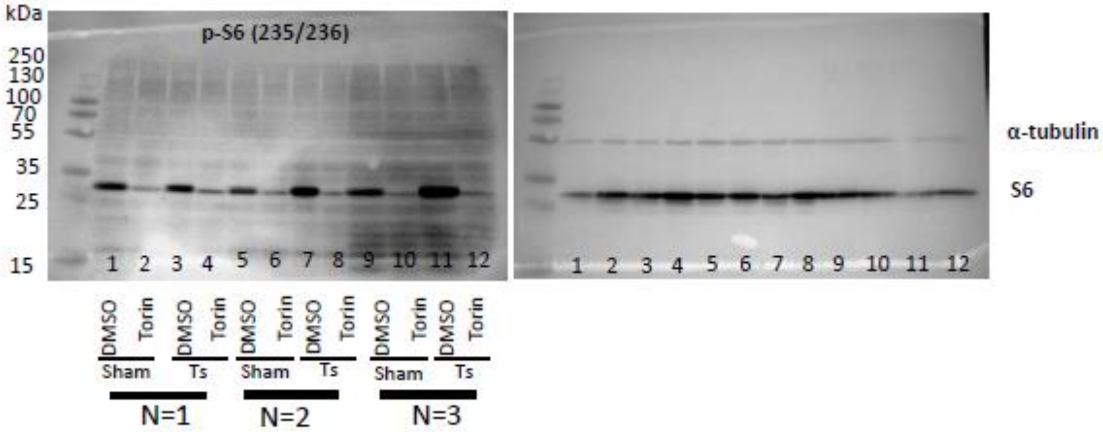

Lanes 7 and 8 included in figure S4b. The figure includes three independent experiments (N=1, 2 and 3).

1
