# Supplementary material for: Analysis of the early response to spinal cord injury identified a key role for mTORC1 signaling in the activation of neural stem progenitor cells
Source: NPJ Regen Med. 2021 Oct 22;6:68. doi: 10.1038/s41536-021-00179-3 (PMC8536777; doi:10.1038/s41536-021-00179-3)
Supplement: Supplementary file 2 — Reporting Summary [file 41536_2021_179_MOESM2_ESM.pdf]

nature portfolio

Corresponding author(s): Juan Larraín

Last updated by author(s): Aug 25, 2021

Reporting Summary

Nature Portfolio wishes to improve the reproducibility of the work that we publish. This form provides structure for consistency and transparency in reporting. For further information on Nature Portfolio policies, see our Editorial Policies and the Editorial Policy Checklist.

Statistics

For all statistical analyses, confirm that the following items are present in the figure legend, table legend, main text, or Methods section.

n/a	Confirmed	
☐	☒	The exact sample size (n) for each experimental group/condition, given as a discrete number and unit of measurement
☐	☒	A statement on whether measurements were taken from distinct samples or whether the same sample was measured repeatedly
☐	☒	The statistical test(s) used AND whether they are one- or two-sided<br>*Only common tests should be described solely by name; describe more complex techniques in the Methods section.*
☒	☐	A description of all covariates tested
☐	☒	A description of any assumptions or corrections, such as tests of normality and adjustment for multiple comparisons
☐	☒	A full description of the statistical parameters including central tendency (e.g. means) or other basic estimates (e.g. regression coefficient) AND variation (e.g. standard deviation) or associated estimates of uncertainty (e.g. confidence intervals)
☐	☒	For null hypothesis testing, the test statistic (e.g. F, t, r) with confidence intervals, effect sizes, degrees of freedom and P value noted<br>*Give P values as exact values whenever suitable.*
☒	☐	For Bayesian analysis, information on the choice of priors and Markov chain Monte Carlo settings
☐	☒	For hierarchical and complex designs, identification of the appropriate level for tests and full reporting of outcomes
☒	☐	Estimates of effect sizes (e.g. Cohen's d, Pearson's r), indicating how they were calculated

Our web collection on statistics for biologists contains articles on many of the points above.

Software and code

Policy information about availability of computer code

Data collection	n/a
Data analysis	FASTQC software; Bowtie-RSEM; Bowtie2-RSEM; ComBat function in SVA package; BlastP (-evalue e-5 -max_target_seqs 5); InterProScan, Blast2GO; GPy (Phython)

For manuscripts utilizing custom algorithms or software that are central to the research but not yet described in published literature, software must be made available to editors and reviewers. We strongly encourage code deposition in a community repository (e.g. GitHub). See the Nature Portfolio guidelines for submitting code & software for further information.

Data

Policy information about availability of data

All manuscripts must include a data availability statement. This statement should provide the following information, where applicable:

- Accession codes, unique identifiers, or web links for publicly available datasets
- A description of any restrictions on data availability
- For clinical datasets or third party data, please ensure that the statement adheres to our policy

mRNA-seq data is available on NCBI Gene Expression Omnibus (GEO) database repository (Accession number: GSE165343; Token: ktwxsgagnrcpdkr). All data generated or analyzed during this study are included in this published article (and its supplementary information files). Other data, coding or reagents are available upon request from the corresponding author.

Field-specific reporting

Please select the one below that is the best fit for your research. If you are not sure, read the appropriate sections before making your selection.

☒ Life sciences ☐ Behavioural & social sciences ☐ Ecological, evolutionary & environmental sciences

For a reference copy of the document with all sections, see nature.com/documents/nr-reporting-summary-flat.pdf

Life sciences study design

All studies must disclose on these points even when the disclosure is negative.

Sample size	Sample size was determined considering previous studies performed in the lab, for which the effect of sampling size have already been calculated (Lee-Liu et al., 2014). In addition, we have in consideration the bioethical constrains.
Data exclusions	One data set from the TUNEL assay was excluded before quantification due to abnormal behaviour of the tissues obtained (morphological changes never observed before).
Replication	All data presented in this study have been obtained in at least 3 independent experiment, thus, at least 3 biological replicates.
Randomization	n/a
Blinding	The analyses were not blinded because most of them were automated or semi-automatized assays.

Reporting for specific materials, systems and methods

We require information from authors about some types of materials, experimental systems and methods used in many studies. Here, indicate whether each material, system or method listed is relevant to your study. If you are not sure if a list item applies to your research, read the appropriate section before selecting a response.

Materials & experimental systems

n/a	Involved in the study	
☐	☒	Antibodies
☒	☐	Eukaryotic cell lines
☒	☐	Palaeontology and archaeology
☐	☒	Animals and other organisms
☒	☐	Human research participants
☒	☐	Clinical data
☒	☐	Dual use research of concern

Methods

n/a	Involved in the study	
☒	☐	ChIP-seq
☒	☐	Flow cytometry
☒	☐	MRI-based neuroimaging

Antibodies

Antibodies used	p-S6 (S235/236) cell signaling 2211; S6 cell signaling 2217; p-4E-BP1 cell signaling 2855; 4E-BP1 cell signaling 9644; SOX2 cell siganling 4900S, PCNA sigma-Aldrich P8825; alpha-tubulin Abcam ab7291; Neurofilament-200 Sigma-Aldrich N4142
Validation	Antibodies against Sox2, PCNA, alpha-tubulin and Neurofilament-200 were previously validated and used in published papers from the lab (Muñoz et al., 2015; Edwards-Faret et al., 2018; Méndez-Olivos et al., 2017). Antibodies against p-S6, S6, p-4E-BP1, 4E-BP1 were validated for the present work, briefely, a battery of antibodies were obteined and they eficacy for detecting the specific proteins in Xenopus was determined.

Animals and other organisms

Policy information about studies involving animals; ARRIVE guidelines recommended for reporting animal research

Laboratory animals	Xenopus leavis
Wild animals	n/a
Field-collected samples	n/a
Ethics oversight	Animal procedures were approved by the Scientific Ethics Committee for the Care of Animals and Environment of the Pontificia Universidad Católica de Chile

Note that full information on the approval of the study protocol must also be provided in the manuscript.
